# Supplementary material for: Review of the land snails of the genus Kora from Brazil, with description of eight new species and a new related genus Koltrora, including comparison with two Andean Neopetraeus species (Gastropoda, Eupulmonata, Orthalicoidea)
Source: PLoS One. 2024 Dec 19;19(12):e0315272. doi: 10.1371/journal.pone.0315272 (PMC13052136; doi:10.1371/journal.pone.0315272)
Supplement: S1 Appendix — (DOCX) [file pone.0315272.s001.docx]

**Appendix 1 –list of characters used in the phylogenetic analysis**

1. Color: 0= uniform pale beige; 1= mosaic of color (Olympus, Drymaeus, Sanniostracus, Anctus, Neopetraeus); 2= brown with subsutural pale band (Kora); 3= uniform brown (Rhinus).
2. General form: 0= globose; 1= discoid (Olympus); 2= turriform (Lavajatus); 3= elliptic (remaining).
3. Size rank: 0= ~30 mm (Olympus, Lavajatus, Drymaeus, Bulimulus, Sanniostracus, Rhinus, Anctus, K nigra, K aetheria, Koltrora); 1= ~45 mm (K corallina, K rupestris, K jimenezi, K uhlei, N tesselatus); 2= ~55 mm (K tupan, K itacarambi, N lobbi).
4. Range of length versus width: 0= ~2.0 (Drymaeus, Bulimulus, Sanniostracus, Anctus, K corallina, K rupestris. K tupan, K aetheria, K jimenezi, K uhlei, Koltrora, N lobbi; 1= ~1.5 (Rhinus, K nigra, K itacarambi, N tesselatus); 2= ~1 (Olympus); 3= ~7 (Lavajatus).
5. Dorso-ventral weak flattening: 0= absent; 1= present (K tupan, K aetheria, Koltrora)
6. Sculpture: 0= absent; 1= axial undulations (Kora, Drymaeus, Bulimulus, Anctus).
7. Spiral micro-stripes: 0= absent; 1= present (Rhinus, Kora).
8. Aperture: 0=indeterminate (not deflected); 1= deflected (determinate), but thin (Olympus, Drymaeus, Sanniostracus, Rhinus, Anctus); 2= deflected and thick (Kora) (additive?)
9. Aperture: 0= uniform with growth: 1= weakly dislocated to right (Kora, Koltrora, Neopetraeus).
10. Outer lip with horizontal portion in superior implantation: 0= absent; 1= present (Rhinus, K nigra, K tupan, K itacarambi, K jimenezi,
11. Columellar middle wide fold in peristome inner lip: 0= absent; 1= present (Kora, Neopetraeus).
12. Umbilicus: 0=simply open; 1=covered ventrally by inner lip (Drymaeus, Kora, Bulimulus, Sanniostracus, Rhinus, Anctus. Koltrora, Neopetraeus)
13. Umbilicus: 0= restricted to peristome; 1= going further along columella (Kora, Neopetraeus).
14. Umbilicus: 0= closed to narrow; 1= widely opened (Anctus, K corallina, K nigra, K itacarambi, K aetheria, K jimenezi, Neopetraeus).
15. Protoconch sculpture: 0= absent; 1= reticulate (Drymaeus, Bulimulus, Sanniostracus, Rhinus); 2= smooth followed by axial cords (Kora).
16. Calcified epiphragm: 0= absent; 1= present (Kora, Koltrora) (? In Neopetraeus).
17. Pair of secondary columellar muscles: 0= absent (Olympus, Lavajatus); 1= present.
18. Secondary pair of columellar muscles with medial pair of bundles: 0= absent; 1= present (Kora).
19. Number of anterior insertions of left secondary pair of columellar muscles: 0= 2; 1= 3-4 (Anctus, K jimenezi, K uhlei, Koltrora, Neopetraeus); 2= 5 (K itacarambi, K aetheria); 3= 7-8 (K nigra, K rupestris, K tupan); 4= +10 (K corallina).
20. Number of anterior insertions of right secondary pair of columellar muscles: 0= 2-4; 1= 6-7 (K corallina, K, nigra, K rupestris, K tupan, K aetheria).
21. Secondary pair of columellar muscles with medial branch differentiated: 0= absent; 1= present (K nigra, K rupestris, K tupan, K itacarambi, K aetheria, K uhlei).
22. Mantle edge: 0= lacking secondary folds; 1= with large, triangular fold (Bulimulus, Sanniostracus, Anctus); 2= with rounded secondary folds (K corallina, K aetheria, K jimenezi, K uhlei, Koltrora); 3= with pointed secondary folds (K nigra, K rupestris, K tupan, K itacarambi).
23. Intercalated pair of vessels at left from pulmonary vessel: 0= absent; 1= present (Kora **except** K jimenezi, Koltrora).
24. Anterior end of pulmonary vessel (cv): 0= simple; 1= branched (K corallina, K tupan, K itacarambi, K aetheria, K uhlei, Neopetraeus).
25. Ureter aperture: 0= 100-30%; 1= ~15% (Drymaeus); 2= 5% (Olympus); 3= 0% (Lavajatus, Kora, Koltrora, Bulimulus, Sanniostracus, Rhinus, Anctus).
26. % of reno-pericardial area in pulmonary cavity: 0= ~1/4; 1= ~1/10 (Kora, Sanniostracus).
27. Kidney: 0= mostly solid; 1= hollow inner space surrounded by tall glandular folds (Kora); 2= hollow but extremely elongated (Olympus).
28. Jaw plate: 0= single plate; 1= strong central notch (Drymaeus, K corallina, K rupestris, K aetheria, Neopetraeus); 2= narrow horseshoe-shaped (Bulimulus, Rhinus, K jimenezi); 3= slightly rectangular (K nigra, K tupan, K itacarambi, K uhlei, Koltrora); 4= absent (Lavajatus).
29. Jaw plate sculpture: 0= fold-like; 1= rib-like (Kora).
30. Radula: clear separation between rachidian and lateral teeth: 0= present; 1= slightly different (Drymaeus, Bulimulus, Sanniostracus, Rhinus, Anctus, Neopetraeus); 2= absent (Kora, Koltrora).
31. Radula: clear separation between lateral and marginal teeth: 0= present; 1= slightly different (Drymaeus, Bulimulus, Sanniostracus, Rhinus, Anctus, Neopetraeus); 2= absent (Kora, Koltrora).
32. Radular teeth: 0= curved inwards; 1= curved outwards (Drymaeus, Bulimulus, Neopetraeus, Kora, Koltrora, Sanniostracus, Rhinus, Anctus).
33. Radular lateral teeth cusps: 0= single, blunt; 1= bifid, asymmetric (Olympus, Lavajatus, Drymaeus, Bulimulus, Sanniostracus, Rhinus, Anctus, Neopetraeus); 2= single, very long (Kora, Koltrora).
34. Radular marginal teeth cusps: 0= similar to lateral teeth; 1= multicuspid (Drymaeus, Sanniostracus, Rhinus).
35. Radular teeth width: 0=relatively wide; 1= very narrow (Kora, Koltrora).
36. Radular sac widely bulging posteriorly in buccal mass: 0= absent; 1= present (Kora).
37. Radular length: 0= slightly longer than buccal mass; 1= about twice buccal mass length (Kora).
38. Pair m1l: 0= absent; 1= present (Lavajatus, K rupestris, K tupan, K aetheria, K jimenezi, K uhlei, Koltrora, Neopetraeus).
39. Pair m1v duplicated: 0= absent; 1= present (Anctus, K corallina, K nigra).
40. M2 insertion: 0= as 2 separated bundles; 1= practically as single bundle (Drymaeus, Kora); 2= in anterior region (Lavajatus).
41. Pair m2a: 0= absent; 1= present (Anctus, K itacarambi, K jimenezi).
42. M3: 0= absent; 1= pair of transverse muscles in ventral region of esophageal origin (Sanniostracus, Rhinus, K. corallina); 2= pair dorso-ventral (Olympus, Anctus, Kora **except** K jimenezi).
43. M3: 0= absent; 1= as latero-lateral muscle in esophageal origin (Rhinus); 2= strong ventral pair (m3p) (Olympus).
44. M5: 0= separated from m4; 1= covering m4 (Bulimulus, Drymaeus, Sanniostracus, Anctus, Koltrora, Kora **except** following); 2= as continuation of m4 (K tupan, K uhlei); 3= as single mass (Olympus, Neopetraeus).
45. M6: 0= present; 1= absent (Lavajatus, Anctus, Kora).
46. M7: 0= absent (Anctus, Neopetraeus); 1= as 2 separated muscles (Olympus, Lavajatus, Bulimulus, Drymaeus, Sanniostracus, Koltrora); 2= as single narrow bundle (K corallina, K nigra, K rupestris, K itacarambi, K aetheria); 3= with 3 bundles (K tupan); 4= broad, thick (K jimenezi); 5= filiform (K uhlei).
47. M8: 0= absent; 1= present (Kora).
48. M10: 0= posterior inserted; 1= anterior inserted (Kora).
49. M10: 0= broad; 1= narrow (Kora **except** K tupan & K aetheria).
50. M11: 0= well-developed; 1= not detected (Kora, Koltrora, Drymaeus).
51. Mj forming ventral muscular platform: 0= absent; 1= present (Kora, Koltrora).
52. % of fusion between odontophore cartilages: 0= 0%; 1= ~20% (Olympus, Sanniostracus, Rhinus); 2= ~50% (Lavajatus, Drymaeus, N lobbii, K nigra); 3= ~80%; (K. corallina, K rupestris, K tupan, K itacarambi, K aetheria, K jimenezi, N tesselatus); 4= ~90% (K uhlei); 5= 100% (Anctus, Koltrora). (add)
53. Salivary gland: 0= small, as 2 separated masses; 1= large, fused in single mass (Drymaeus, Kora).
54. Salivary gland aperture in dorsal wall of buccal cavity: 0= in middle level; 1= in posterior region (Rhinus, Anctus, K tupan, K jimenezi, Koltrora, Neopetraeus).
55. Salivary gland aperture on a papilla: 0= absent; 1= present (K jimenezi, K uhlei).
56. Stomach: 0= bulbed; 1= as simple curve (Drymaeus, Sanniostracus, Rhinus, Kora, Anctus).
57. Duct to anterior lobe of digestive gland: 0= in esophagus (*D. currais)*; 1= in stomach (Drymaeus (except *D. currais*), Sanniostracus, Rhinus, Kora, Anctus).
58. Anterior duct to digestive gland: 0= branched to both sides; 1-= branched only to right side (K nigra, K tupan, K itacarambi, K jimenezi, K uhlei, Neopetraeus).
59. Posterior duct to digestive gland: 0= simple branched tube; 1= tube with right branches only (Kora **except** K nigra).
60. Anus: 0= opening inside lung; 1= opening directly outside (Olympus, Lavajatus, Bulimulus, Drymaeus, Sanniostracus, Rhinus, Kora, Anctus, Neopetraeus, Koltrora).
61. Hermaphrodite duct (hd) insertion: 0= simple; 1= strongly curved (Kora **except** K nigra, N lobbii).
62. Form of seminal receptacle: 0= balloon-like; 1= digitiform-cylindric (Bulimulus, Drymaeus, Sanniostracus, Rhinus, Anctus); 2= digitiform-flattened (Kora), 3= multilobed (Lavajatus).
63. Seminal receptacle: 0= straight; 1= curved (Anctus, K jimenezi).
64. Insertion of hermaphrodite duct in carrefour: 0= in its base; 1= in its tip (Olympus, Lavajatus).
65. Carrefour form: 0= filiform; 1= conic (Drymaeus, Sanniostracus, Rhinus, Anctus, K corallina, K tupan, K itacarambi, K aetheria, K jimenezi, K uhlei, Neopetraeus), 2= entirely narrow (Bulimulus sula, K rupestris, Koltrora); 3= elongated (K nigra).
66. Carrefour duct: 0= absent; 1= narrow-long (Olympus, Lavajatus, Anctus, Drymaeus, Bulimulus, Sanniostracus, K nigra, K rupestris, K tupan, K aetheria; K uhlei, Koltrora, Neopetraeus); 2= narrow-short (K itacarambi, K jimenezi); 3= short-wide (Rhinus, K corallina)
67. Carrefour: bulged distal region by side of receptacle: 0= absent; 1= present (Anctus, K tupan, K jimenezi, K uhlei, N lobbii).
68. Insertion of carrefour: 0= in spermoviduct (Olympus, Lavajatus, D. currais, K rupestris, Koltrora); 1= in duct of albumen gland (Anctus, Drymaeus castilhensis, D. micropyrus, K corallina, Neopetraeus); 2= between albumen gland duct and accessory albumen chamber (K nigra, K tupan, K uhlei); 3= in albumen chamber (K itacarambi, K jimenezi); 4= between albumen duct and spermoviduct (K aetheria).
69. Albumen chamber: 0= as separated sac; 1= as simple curve preceding spermoviduct (Anctus, K corallina, K itacarambi, K jimenezi, K uhlei, Neopetraeus); 2= double (Sanniostracus); 3= in intersection of both (Rhinus); 4= absent (Olympus, Lavajatus).
70. Accessory albumen chamber (as): 0= absent; 1= present (Kora, Neopetraeus); 2= a diverticulum (Sanniostracus).
71. % of prostate in spermoviduct: 0= ~50%; 1= ~35% (Drymaeus, K corallina, K nira, K aetheria, Koltrora); 2= ~20% (Neopetraeus).
72. Number of sperm folds in spermoviduct: 0= 1; 1= 2 (K nigra); 2= 0 (Bulimulus, Neopetraeus).
73. Uterus wall: 0= thin; 2= thick glandular (Drymaeus, Sanniostracus).
74. Vas deferens origin: 0= simple hole; 1= in confluence of pair of folds (Kora).
75. Duct of bursa copulatrix: 0= weakly muscular; 1= strongly muscular (Kora **except** K jimenezi).
76. Duct of bursa length: 0= considerable % of spermoviduct; 1= shorter than 25% of spermoviduct (Olympus, Lavajatus).
77. Vas deferens insertion in penis: 0= simple; 1= strongly curved (K nigra, K tupan, K itacarambi, K uhlei).
78. Penis form: 0= stubby-claviform; 1= slightly filiform (Lavajatus, Drymaeus, Sanniostracus, Kora, Anctus).
79. Penis length: 0= ~1/2 of spermoviduct; 1= ~75% of spermoviduct (Drymaeus, Sanniostracus, Anctus, K corallina, K aetheria); 2= 85-90% (K tupan, K itacarambi, N tesselatus); 3= ~100% (K nigra, K jimenezi).
80. Penis shield: 0= absent (Rhinus); 1= only in penis base (Bulimulus, Sanniostracus, Anctus, Kora); 2= ~1/3 pf penis (Drymaeus). (add)
81. Penis muscle insertion: 0= at tip of penis; 1= at tip of epiphallus (Drymaeus, Sanniostracus, Anctus, K corallina, K nigra, K tupan, K aetheria, K jimenezi, Neopetraeus); 2= base of epiphallus (K rupestris, K itacarambi, K uhlei); 3= subterminal (Olympus, Koltrora).
82. Penis distinct muscular basal wall: 0= absent; 1= present (kora **except** K jimenezi).
83. Penis inner organization: 0= single chamber with large fold; 1= 2 chambers separated by transverse fold (Bulimulus, Sanniostracus); 2= elongated chamber with special mosaic of low folds (Drymaeus, Rhinus, Anctus); 3= divided into compartments (Kora); 4= 2 chambers separated by middle narrow duct (Olympus).
84. Pair of longitudinal strong folds in proximal penial region: 0= absent; 1= present (Kora, Koltrora).
85. Type of pair of longitudinal folds: 0= simple (K nigra, K rupestris, K itacarambi, K jimenezi); 1= imbricated (K corallina, K aetheria, K uhlei); 2= forming wing-like folds (K tupan); 3= fused distally (Koltrora) (- in folds-lacking taxa).
86. Pair of longitudinal penial folds – imbricated projections: 0= entire (K corallina); 1= distal region only (K aetheria, K uhlei) (- for lacking).
87. Penis umbrella-like transverse fold: 0= absent; 1= present (K rupestris, K tupan, K itacarambi, K aetheria, K uhlei).
88. Number of rods in umbrella-like transverse fold: 0= 6 (K aetheria); 1= 5 (K itacarambi, K rupestris); 2= 3 (K tupan, K uhlei) (- in folds-lacking taxa).
89. Epiphallus length: 0= ~50% of penis; 1= absent (Olympus, Bulimulus); 2= ~10-15% of penis (Lavajatus, Drymaeus, Sanniostracus, Rhinus, K rupestris); 3= ~35% of penis (Anctus, K corallina, K aetheria, N lobbii); 4- 20-25% (K nigra, K tupan, K itacarambi, K jimenezi, K uhlei, Koltrora, N tesselatus).
90. Epiphallus inner large longitudinal fold: 0= absent; 1= present (Drymaeus, Sanniostracus, Rhinus, K corallina, K tupan, K itacarambi, Neopetraeus).
91. Folds converging to vas deferens aperture: 0= from penis; 1= from epiphallus (Kora **except** K jimenezi, K rupestris).
92. Nerve ring with pleural ganglia close to pedal ganglia: 0= absent; 1= present (Drymaeus, Sanniostracus, Rhinus, Anctus, Kora, Koltrora, Neopetraeus).
93. Pleural commissure: 0= absent; 1= present (Drymaeus, Sanniostracus, Rhinus, Anctus, Kora, Koltrora, Neopetraeus).
94. Cerebral commissure: 0= wide; 1= short (Drymaeus, Sanniostracus, Kora).
