# Supplementary material for: Review of the land snails of the genus Kora from Brazil, with description of eight new species and a new related genus Koltrora, including comparison with two Andean Neopetraeus species (Gastropoda, Eupulmonata, Orthalicoidea)
Source: PLoS One. 2024 Dec 19;19(12):e0315272. doi: 10.1371/journal.pone.0315272 (PMC13052136; doi:10.1371/journal.pone.0315272)
Supplement: S2 Appendix — (DOCX) [file pone.0315272.s002.docx]

**Appendix 2 –matrix of characters used in the phylogenetic**

**analysis**

| *Sp\character* | **1 2 3 4**  **1234567890 1234567890 1234567890 1234567890 12345** |
| --- | --- |
| *Kora corallina* | 2310011210 1111211141 0211311112 2120111011 01011 |
| *Kora nigra* | 2301011211 1111211131 1310311312 2120111011 02011 |
| *Kora rupestris* | 2310011210 1110211131 1310311112 2120111101 02011 |
| *Kora tupan* | 2320111211 1110211131 1311311312 2120111101 02021 |
| *Kora ajar* | 2321011211 1111211120 1311311312 2120111001 12011 |
| *Kora aetheria* | 2300111210 1111211121 1211311112 2120111101 02011 |
| *Kora jimenezi* | 2310011211 1111211110 0200311212 2120111101 10011 |
| *Kora uhlei* | 2310011210 1110211110 1211311312 2120111101 02021 |
| *Koltrora pyrostoma* | 2300100110 0100011010 0210300302 2120111100 00010 |
| *Neopetraeus lobbi* | 1320000110 11110?1010 0001000101 1110000100 00030 |
| *Neopetr. tesselatus* | 1311000110 11110?1010 0001000101 1110000100 00030 |
| *Drymaeus castilhensis* | 1300010100 0100101000 0000100001 1111000001 00010 |
| *Drymaeus micropyrus* | 1300010100 0100101000 0000100001 1111000001 00010 |
| *Drymaeus currais* | 1300010100 0100101000 0000100001 1111000001 00010 |
| *Bulimulus sula* | 0300010100 0100101000 0100300201 1110000000 00010 |
| *Sanniost. carnavalescus* | 1300000100 0100101000 0100310001 1111000000 01010 |
| *Rhinus botocudus* | 3301001101 0100101000 0000300201 1111000000 01100 |
| *Anctus angiostomus* | 1300010100 0101001010 0100300001 1110000010 12011 |
| *Olympus nimbus* | 1102000100 0000000000 0000202000 0010000000 02230 |
| *Lavajatus moroi* | 0203000000 0000000000 0000300400 0010000102 00001 |
| *Strophocheilidae* | 0000000000 0000000000 0000000000 0000000000 00000 |

| *Sp\character* | **4 5 6 7 8 9**  **67890 1234567890 1234567890 1234567890 1234567890 1234** |
| --- | --- |
| *Kora corallina* | 21111 1310011011 1200130111 1001100111 1131100-31 1111 |
| *Kora nigra* | 21111 1210011101 0200310201 1101101131 11310-0-40 1111 |
| *Kora rupestris* | 21111 1310011011 1200210001 0001100101 21310-1120 0111 |
| *Kora tupan* | 31101 1311011111 1200111201 0001101121 11312-1241 1111 |
| *Kora ajar* | 21111 1310011111 1200120311 0001101121 21310-1141 1111 |
| *Kora aetheria* | 21101 1310011011 1200110401 1001100111 1131111030 1111 |
| *Kora jimenezi* | 41111 1311111111 1210121311 0001000131 10310-0-40 0111 |
| *Kora uhlei* | 51111 1410111111 1200111211 0001101101 2031111240 1111 |
| *Koltrora pyrostoma* | 10001 1501000001 0000210000 1000000000 30013-0-40 0110 |
| *Neopetraeus lobbi* | 00000 0201000101 1000111111 2200000001 1000--0-31 0110 |
| *Neopetr. tesselatus* | 00000 0301000101 0000110111 2200000021 1000--0-41 0110 |
| *Drymaeus castilhensis* | 10001 0210011001 0100110100 1010000112 1020--0-21 0111 |
| *Drymaeus micropyrus* | 10001 0210011001 0100110100 1010000112 1020--0-21 0111 |
| *Drymaeus currais* | 10001 0210010001 0100110000 1010000112 1020--0-21 0111 |
| *Bulimulus sula* | 10000 0000000001 0100210000 0200000001 0010--0-10 0000 |
| *Sanniost. carnavalescus* | 10000 0100011001 0100110022 0010000111 1010--0-21 0111 |
| *Rhinus botocudus* | 00000 0101011001 0100130030 0000000000 0020--0-21 0110 |
| *Anctus angiostomus* | 00000 0501011001 0110111110 0000000111 1020--0-30 0110 |
| *Olympus nimbus* | 10000 0100000001 0001010040 0000010000 3040--0-10 0000 |
| *Lavajatus moroi* | 10000 0200000001 0301010040 0000010100 0000--0-20 ?000 |
| *Strophocheilidae* | 00000 0000000000 0000000000 0000000000 0000--0-00 0000 |
