# Supplementary material for: Review of the land snails of the genus Kora from Brazil, with description of eight new species and a new related genus Koltrora, including comparison with two Andean Neopetraeus species (Gastropoda, Eupulmonata, Orthalicoidea)
Source: PLoS One. 2024 Dec 19;19(12):e0315272. doi: 10.1371/journal.pone.0315272 (PMC13052136; doi:10.1371/journal.pone.0315272)

PLOS ONE

Review of the genus Kora from Brazil, with description of eight new species and a new related genus Koltrora, including comparison with two Andean Neopetraeus species (Gastropoda, Eupulmonata, Orthalicoidea).

--Manuscript Draft--

Manuscript Number: PONE-D-23-41760

Article Type: Research Article

Full Title: Review of the genus Kora from Brazil, with description of eight new species and a new related genus Koltrora, including comparison with two Andean Neopetraeus species (Gastropoda, Eupulmonata, Orthalicoidea).

Short Title: Review of orthalicoidean Kora from Brazil

Corresponding Author: Luiz Ricardo L. Simone, Dr.
Universidade de São Paulo: Universidade de Sao Paulo
São Paulo, São Paulo BRAZIL

Keywords: Molluca; Phylogeny; Anatomy; Morphology; Phenotypy; Systematics; Taxonomic review; molecular counterpoint

Abstract: The orthalicoidean genus Kora Simone, 2012, is reviewed. The three known species are redescribed, including their anatomy. These species are K. corallina (the type species), K. nigra, and K. rupestris. Eight new species are introduced, all of which occur in the region of the São Francisco River, from the north of Minas Gerais to the south of Bahia, Brazil. They are K. tupan, K. ajar, K. aetheria, K. jimenezi, K. uhlei, K. kremerorum, K. vania, and K. curumim. All of them are described, including anatomical features, except the last three, which are based solely on shell characters. Another related genus is also described, Koltrora, with a single new species, K. pyrostoma. They are compared to ~~two sample~~[illegible]an Andean genus, Neopetraeus (N. lobbii, N. tesselatus), which also exhibit sim[illegible]es. [illegible] detailed phenotypic study [illegible]erformed [illegible]everal comparative ways, including a p[illegible]ogenetic approach, using o[illegible] [illegible]alicoideans with the same level of phenotypic details known. The single objective is to better [illegible]ablish the currently suggested taxonomy and to provide a brief comparison with rece[illegible]esults based on molecular approaches. According to these preliminary results, the triad Neopetraeus-Koltrora-Kora is monophyletic, supported by 10 synapomorphies. Koltrora and Kora are sister taxa, supported by 11 synapomorphies. Kora is monophyletic, with strong support from 25 synapomorphies. Discussions on classification, phylogeny, anatomy, and comparison with other recent orthalicoidean literature are also included. [illegible]he new structures are discovered, such as the odontophore pair of muscle[illegible] the accessory albumen chamber, a different kind of [illegible]rmatophore, the exclusiv[illegible]d of radula of Kora and Koltrora, and a calcified [illegible]phragm, unique in South American snails. Register ZooBank: urn:lsid:zoobank.org:pub:2F13D53C-0A36-42FB-B936-D80E7C958259.

Order of Authors: Luiz Ricardo L. Simone, Dr.

Additional Information:

Question	Response
**Financial Disclosure**<br><br>Enter a financial disclosure statement that describes the sources of funding for the work included in this submission. Review the submission guidelines for detailed requirements. View published research articles from *PLOS ONE* for specific examples.	The author(s) received no specific funding for this work.

Powered by Editorial Manager® and ProduXion Manager® from Aries Systems Corporation

This statement is required for submission and **will appear in the published article** if the submission is accepted. Please make sure it is accurate.<br><br>**Funded studies**<br>Enter a statement with the following details:<br>• Initials of the authors who received each award<br>• Grant numbers awarded to each author<br>• The full name of each funder<br>• URL of each funder website<br>• Did the sponsors or funders play any role in the study design, data collection and analysis, decision to publish, or preparation of the manuscript?<br><br>Did you receive funding for this work?	
**Competing Interests**<br><br>Use the instructions below to enter a competing interest statement for this submission. On behalf of all authors, disclose any competing interests that could be perceived to bias this work—acknowledging all financial support and any other relevant financial or non-financial competing interests.<br><br>This statement is required for submission and **will appear in the published article** if the submission is accepted. Please make sure it is accurate and that any funding sources listed in your Funding Information later in the submission form are also declared in your Financial Disclosure statement.<br><br>View published research articles from *PLOS ONE* for specific examples.	NO authors have competing interests

Powered by Editorial Manager® and ProduXion Manager® from Aries Systems Corporation

**NO authors have competing interests**<br><br>Enter: *The authors have declared that no competing interests exist.*<br><br>**Authors with competing interests**<br><br>Enter competing interest details beginning with this statement:<br><br>*I have read the journal's policy and the authors of this manuscript have the following competing interests: [insert competing interests here]*<br><br>* typeset	
**Ethics Statement**<br><br>Enter an ethics statement for this submission. This statement is required if the study involved:<br><br>• Human participants<br>• Human specimens or tissue<br>• Vertebrate animals or cephalopods<br>• Vertebrate embryos or tissues<br>• Field research<br><br>Write "N/A" if the submission does not require an ethics statement.<br><br>General guidance is provided below. Consult the submission guidelines for detailed instructions. **Make sure that all information entered here is included in the Methods section of the manuscript.**	N/A

**Format for specific study types**<br><br>**Human Subject Research (involving human participants and/or tissue)**<br>• Give the name of the institutional review board or ethics committee that approved the study<br>• Include the approval number and/or a statement indicating approval of this research<br>• Indicate the form of consent obtained (written/oral) or the reason that consent was not obtained (e.g. the data were analyzed anonymously)<br><br>**Animal Research (involving vertebrate animals, embryos or tissues)**<br>• Provide the name of the Institutional Animal Care and Use Committee (IACUC) or other relevant ethics board that reviewed the study protocol, and indicate whether they approved this research or granted a formal waiver of ethical approval<br>• Include an approval number if one was obtained<br>• If the study involved *non-human primates*, add *additional details* about animal welfare and steps taken to ameliorate suffering<br>• If anesthesia, euthanasia, or any kind of animal sacrifice is part of the study, include briefly which substances and/or methods were applied<br><br>**Field Research**<br><br>Include the following details if this study involves the collection of plant, animal, or other materials from a natural setting:<br>• Field permit number<br>• Name of the institution or relevant body that granted permission	
**Data Availability**<br><br>Authors are required to make all data underlying the findings described fully available, without restriction, and from the time of publication. PLOS allows rare exceptions to address legal and ethical concerns. See the PLOS Data Policy and FAQ for detailed information.	Yes - all data are fully available without restriction

A Data Availability Statement describing where the data can be found is required at submission. Your answers to this question constitute the Data Availability Statement and **will be published in the article**, if accepted.<br><br>**Important:** Stating 'data available on request from the author' is not sufficient. If your data are only available upon request, select 'No' for the first question and explain your exceptional situation in the text box.<br><br>Do the authors confirm that all data underlying the findings described in their manuscript are fully available without restriction?	
**Describe where the data may be found in full sentences. If you are copying our sample text, replace any instances of XXX with the appropriate details.**<br><br>• If the data are **held or will be held in a public repository**, include URLs, accession numbers or DOIs. If this information will only be available after acceptance, indicate this by ticking the box below. For example: *All XXX files are available from the XXX database (accession number(s) XXX, XXX.*).<br>• If the data are all contained **within the manuscript and/or Supporting Information files**, enter the following: *All relevant data are within the manuscript and its Supporting Information files.*<br>• If neither of these applies but you are able to provide **details of access elsewhere**, with or without limitations, please do so. For example:<br><br>*Data cannot be shared publicly because of [XXX]. Data are available from the XXX Institutional Data Access / Ethics Committee (contact via XXX) for researchers who meet the criteria for access to confidential data.*<br><br>*The data underlying the results presented in the study are available from (include the name of the third party*	All relevant data are within the manuscript and its Supporting Information files.

and contact information or URL).<br>• This text is appropriate if the data are owned by a third party and authors do not have permission to share the data.<br><br>* typeset	
Additional data availability information:	Tick here if your circumstances are not covered by the questions above and you need the journal's help to make your data available.

Powered by Editorial Manager® and ProduXion Manager® from Aries Systems Corporation

Manuscript
Click here to access/download;Manuscript;Kora Review.docx

Review of the genus *Kora* from Brazil, with description of eight new species and a new related genus *Koltrora*, including comparison with two Andean *Neopetraeus* species (Gastropoda, Eupulmonata, Orthalicoidea).

Luiz Ricardo L. Simone

Museu de Zoologia da Universidade de São Paulo, Cx. Postal 42391; 04218-970 São Paulo, SP, Brazil; lrsimone@usp.br; lrlsimone@gmail.com; OrcID: 0000-0002-1397-9823

Abstract

The orthalicoidean genus *Kora* Simone, 2012, is reviewed. The three known species are redescribed, including their anatomy. These species are *K. corallina* (the type species), *K. nigra*, and *K. rupestris*. Eight new species are introduced, all of which occur in the region of the São Francisco River, from the north of Minas Gerais to the south of Bahia, Brazil. They are *K. tupan*, *K. ajar, K. aetheria, K. jimenezi, K. uhlei, K. kremerorum, K. vania*, and *K. curumim*. All of them are described, including anatomical features, except the last three, which are based solely on shell characters. Another related genus is also described, *Koltrora*, with a single new species, *K. pyrostoma*. They are compared to two samples of an Andean genus, *Neopetraeus* (*N. lobbii, N. tesselatus*), which also exhibit similarities. The detailed phenotypic study is performed in several comparative ways, including a phylogenetic approach, using other orthalicoideans with the same level of phenotypic details known. The single objective is to better establish the currently suggested taxonomy and to provide a brief comparison with recent results based on molecular approaches. According to these preliminary results, the triad *Neopetraeus-Koltrora-Kora* is monophyletic, supported by 10 synapomorphies. *Koltrora* and *Kora* are sister taxa, supported by 11 synapomorphies. *Kora* is monophyletic, with strong support from 25 synapomorphies. Discussions on classification, phylogeny, anatomy, and comparison with other recent orthalicoidean literature are also included. Some new structures are discovered, such as the odontophore pair of muscles m8, the accessory albumen chamber, a different kind of spermatophore, the exclusive kind of radula of *Kora* and *Koltrora*, and a calcified epiphragm, unique in South American snails. Register ZooBank: urn:lsid:zoobank.org:pub:2F13D53C-0A36-42FB-B936-D80E7C958259.

Introduction

The Orthalicoidea Brazilian genus *Kora* Simone, 2012 [1] has as a short, recent and relatively endogenous history. It was introduced in 2012 to shelter a single species, *K. corallina* Simone, 2012 ([1]: 4[illegible]), described only based on shell characteristics in samples from Santa Maria da Vitória, Bahia, being, thus, the type species. Three years later, three new species were described [2], including *K. nigra* Simone, 2015 (Carinhanha, Bahia). The other two species were afterwards transferred to another genus in a paper [3] that also described another species, *K. rupestris* Salvador & Simone, 2016 (also from Carinhanha region, Bahia) ar[illegible]formed an emended diagnosis of the genus.

The genus *Kora* is, thus, so far attributed [illegible]above mentioned three species that [illegible]bits semidry regions of Bahia, Brazil. Morphologically, [illegible] mainly characterized [1, 3] by a medium-sized, relatively monochromatic shell, with color[illegible]ating from brown to beige-orange, with [illegible]er areas. The protoconch has about two whor[illegible] initially smooth, gradually having sinuous [illegible]al ribs. Teleoconch sculptured from growth lines to axial ribs. Peristome expanded laterally, [illegible]ay from the shell axis. Columellar lamella oblique. Umbilicus well-marked. No anatomical information was so far available.

[illegible]he recent years, several new samples of land snails have been brought to study by the tea[illegible]f collectors of the naturalist José Coltro Jr., in a project studying malacofauna from Bahia and Minas Gerais regions in which the soil is calcareous. A truly profusion of forms of several lands nails has been collected. This includes some new K[illegible] samples from the known and unknown species and, [illegible]n better, mostly with soft parts, permitting a anatomic investigations. Similar rece[illegible]esults on eupulmonates in the same project include the urocoptid *Habeas* Sim[illegible] 2012, now with eight species [4, 5], and the stophocheilid *Anthinus* Albers, 1850, now with eight species and the new *Catracca* Simone, 2022 [6].

This paper has, thus, the main objective o[illegible]reviewing the genus *Kora*, [illegible]uding the redescription of the three know[illegible] (and the type) species, this time including a [illegible]nplete anatomical description of topotypes and other samples. [illegible]litionally, [illegible] new species are introduced, four of which also anatomically described. A correlated n[illegible] species, which has [illegible]e similarity with *Kora*, is also described in the new genus *Koltrora*, including anatomical [illegible]ects. [illegible]veying the literature, it was detected that some [illegible]ples of *Kora* were already studied [illegible]viously, misidentified as *Neopetraeus* Martens, 1[illegible] [7]. Unfortunately, that materia[illegible] no longer available, destroyed by the fire at the National Museum of Rio de Janeiro in 2018, and both authors are deceased. However, [illegible]member that 2003 presentation and can confirm that those samples belonged to what is no[illegible]nown as *Kora*. Aiming to [illegible]front that *Neopetraeus* and *Kora* are different taxa, samples of two species of the former [illegible] here also included. *Neopetraeus* is an Andean genus, which includes 15 valid species [8]. It has some resemblance with *Kora* in having a deep umbilicus, and a projected peristome relatively away from shell axis. However, usually its characteristic protoconch is differently sculptured a[illegible] it has a [illegible]gher myriad of shell colors [9], [illegible]at set both genera apart. Trying to perform the taxonomic argumentation on a more w[illegible]-based scenario, beyond the usual comparative discussion, an initial phylogenetic analysis on a sample of Bulimulidae and allies are also [illegible]form[illegible] This has this single objective, as it is recognized that the examined assembly is not represe[illegible]ive in a so diverse family.

Material and methods

Material

A complete list of material examined follows each species' description. Material [illegible]nprised of dry shells or shells and specimens preserved in 70% EtOH. All listed specimens w[illegible] used t[illegible] compose the descriptions; those that are only shells (sh) were examined at a conchological level; all complete specimens (shell and soft parts) (spm) were extracted and dissected. The Table 1 summarizes the total number of specimens in each studied species being shells, dissected individuals and measured shells, emphasizing that no statistical study [illegible]esently provided, the intention is only giving a notion of size.

Table 1. Summary of number of specimens

[illegible]cies	Shells examined	Specimens dissected	Shells measured*
[illegible]a corallina	106	6	5
Kora nigra	27	4	4
Kora rupestris	70	5	2
Kora tupan	5	4	3
Kora ajar	58	15	4
Kora aetheria	30	5	3
Kora jimenezi	115	2	5
Kora uhlei	23	8	2
Kora kremerorum	22	--	4
Kora vania	3	--	3
Kora curumim	1	--	1
Koltrora pyrostoma	55	11	5
Neopetraeus lobbii	2	1	2
Neopetr. tesselatus	2	2	2

*Just to give an idea of size for taxonomy, no statistical intentions.

Methods and scope

Color photos were obtained by digital cameras, either hand-held (Canon Digital Rebel XTi with macro-lenz) or attached to the dissecting microscope (Zeiss AxioCam ERc 5s). Shell measurements were obtained with a digital caliper, for a minimum of 10 adult shells when available. The specimens were dissected by standard techniques [10] under dissecting stereomi[illegible]copes, with the specimen immersed under the fixative. All drawings were obtained with the aid of a camera lucida; initially penciled, afterwards inked; usually drawings produced for each species include data derived from several specimens, as the intraspecific variation has [illegible]wn insignificant. Thus, the anatomical drawings are a composite of all examined sp[illegible]ens. [illegible] scales were obtained putting a ruler by side of the specimen. The type and voucher ma[illegible]l are mainly deposited in Museu de Zoologia da Universidade de São Paulo (MZSP) malacological collection, with some duplicates to other indicated museums. The [illegible]cimens usually are relatively easily extracted from their shells, except for few cases (e.g., [illegible]otype of *Koltrora pyrostoma* and *Neopetraeus cremnobates*), in those cases a 'cesarean' (a small window) needed to be[illegible]e in the [illegible] whorl. SEM [illegible]k on the material is standard, with [illegible]ces metalized by gold, an[illegible]amined [illegible]e Laborator[illegible] Electron Microscopy of the MZSP. [illegible]atomical terminology, particularly of the odontophore muscles, follows Simone [10], which has been [illegible]dually explained in Malacopedia project http://w[illegible]moluscos.org/malacopedia_previous.html. [illegible]resent descriptions the anterior genital structures [illegible] penis and vagina, are examined and described in retracted condition, and thus the structures present in internal lumen is called "internal", despite it is recognized [illegible]t all of them are exteriorized, [illegible] become external, during the copula. Th[illegible]aterial studied [illegible] described in this work was collected by a team working for Femorale, a private company [www.femorale.com; http://www.femorale.com/femorale/index.asp]. The places of collection are not within protected areas, and as such collection activity did not require special permits. Nonetheless, the collections were made under general/permanent license IBAMA-Sisbio #10560-2, which permits extraction of wildlife samples for scientific purposes. As most of the studied material was collected by non-scientific expeditions, no further data beyond coordinates and place names were available. Thus, details on vegetation, climate, soil, rainfall, etc., were not available, but, when possible and relevant, these data were extracted from the literature, digital online resources, or official websites. For comparison of the presently studied

species with those already known, the large MZSP collection was consulted. Collections of other European and American museums were also consulted [illegible]~~ny trips~~ seeking type [illegible]cimens, some of them illustrated in a catalogue [11]. The [illegible]nario, style, model and [illegible]posal of items are entirely based on a previous similar paper in this journal [6].

Phylogenetic analysis

The phylogenetic methodology is the same as reported by Simone [6, 10, 12], that basically consists of [illegible] matrix ~~mounted in Nexus,~~ analyzed by programs [illegible]T and PAUP. All [illegible]hem resulted in a single cladogram [illegible]wn below. ~~Thus, beyond the s~~[illegible]~~ies herein stud~~[illegible], the present preliminary phylogeny [illegible]based upon [illegible]eady published [illegible]a of 9 species listed below, as well as additional examination of their voucher material dep[illegible]ed in MZSP [illegible]~~ome~~ unstudied structures, such as~~, e.g.,~~ the odontophore. The list of characters is i[illegible]**ppendix 1**; and respective matrix in **Appendix 2**. ~~As reported above,~~ it is important to emphasize that the shown phylogeny is not to be interpreted as "the phylogeny of the Bulimulidae". It has only the intention of demonstrating that the description of the new genus – *Koltrora* – is necessary, [illegible]~~well to further base~~ the genus *Kora*, helping [illegible] discussion on their placements [illegible]~~der the~~ [illegible]t of recent molecular scenarios reported [illegible]he Discussion. In the literature, a small set of orthalicoideans has their anatomy known in sufficient details for an initial phylogenetic inference. They are:

(1) *Drymaeus castilhensis* Simone & Amaral, 2018 [13];
(2) *D. micropyrus* Simone & Amaral, 2018 [13];
(3) *D. currais* Simone, Belz & Gernet, 2020 [14];
(4) *Bulimulus sula* Simone & Amaral, 2018 [13];
(5) *Sanniostracus carnavalescus* (Simone & Salvador, 2016) [15];
(6) *Rhinus botocudus* Simone & Salvador, 2016 [15];
(7) *Anctus angiostomus* (Wagner, 1827) [16];

As a remote outgroup, the [illegible]und plan of Strophocheilidae, as obtained in Simone's phylogeny (2022 [6], fig. 27, node 2), [illegible]es as the basis for rooting. The seven species mentioned above are considered close outg[illegible]ps but are operationally analyzed as part of the ingroup, which consists of 11 anatomically studied species ~~in this research~~. This methodology has been previously applied in other studies [6, 10, 12]. In the case of [illegible]*tus angiostomus*, the anatomical description provided [illegible] [16] contains some miss[illegible] data, which are addressed later in this paper. Additionally, two o[illegible] species, taxonomically distant from Orthalicoidea, are included as far outgroups (listed below) but are also operationally analyzed as part of the ingroup. This approach is adopted to test the monophyly of the orthalicoidean families, at least within the current assembly of species. It's important to note that ~~it is recognized that~~ this assembly does not encompass the full diversity of Orthalicoidea, but, as mentioned earlier, ~~it~~ represents a preliminary step, primarily aimed at understanding the taxa studied in this paper, especially in light of ~~the new insights from~~ the new classification based on molecular approaches [17], which will be examined in the context of the current morphological analysis in the Discussion ~~item~~. As far outgroups, the following taxa are included:

(8) *Olympus nimbus* Simone 2010 (Solaropsidae) [18]
(9) *Lavajatus moroi* Simone 2018 (Achatinidae/Subulininae) [19]
[illegible])Strophocheilidae ground plan [6] rooting.

Nomenclatural acts

The electronic edition of this article conforms to the requirements of the amended International Code of Zoological Nomenclature, and hence the new names contained herein are available under that Code from the electronic edition of this article. This published work and the nomenclatural acts it contains have been registered in ZooBank, ~~online registration system for the ICZN~~. ~~The~~ ZooBank LSIDs (Life Science Identifi[illegible], can be resolved, and the associated information viewed through any standard web browser by appending the LSID to the prefix "http://zoobank.org/". The LSID for this publication is: urn:lsid:zoobank.org:pub: FC4DD323-EF6A-404B-9755-F124F9DBB6D4. The electronic edition of this work was published in a journal with an ISSN, and has been archived and is available from the following digital repositories: PubMed Central, LOCKSS, ResearchGate. It is important to state that funders had no role in study design, data collection and analysis, decision to publish, or preparation of the manuscript. The author received no specific funding for this work.

Abbreviations in ~~the~~ Figures: **aa**, anterior aorta; ac, albumen chamber; ad, albumen g[illegible]nd duct; **ag**, albumen gland; **an**, anus; **as**, accessory albumen chamber; **au**, auricle; **bc**, bursa copulatrix; **bd**, bursa copulatrix duct; **bg**, buccal ganglion; **bm**, buccal mass; **br**, subradular membrane; **bu**, muscular wall of bursa duct; **bv**, blood vessel; **ca,** carrefour; **cc,** cerebral commissure; **ce**, cerebral ganglion; **cd**, cerebral node; **cl**, left secondary columellar muscle; **cm**, columellar muscle; **cn**, cerebro-pedal and cerebro-pleural connectives; **co**, collar vessel; **cr**, right secondary columellar muscle; **cv**, pulmonary (efferent) vein; **da**, digestive gland anterior lobe; **dc**, dorsal chamber of buccal cavity; **dd**, anterior gastric duct to digestive gland; **df**, dorsal folds of buccal mass; **dg**, digestive gland posterior lobe; **di**, diaphragm or pallial floor; **dp**, posterior gastric duct to digestive gland; **ed**, esophageal dilatation; **ef,** esophageal fold; **eh**, epiphallus; **ei**, epiphallus inner longitudinal fold; **eo**, spermoviduct; **es**, esophagus; **ey**, eye; **fe**, female right lateral sulcus; **fg**, fecal groove; **fo**, free oviduct; **fp**, genital pore; **fs**, foot sole; **ft**, foot; **gm**, genital muscle; **go**, gonad; **gp**, pallial gland; **hd**, hermaphrodite duct; **if**, inner fold of pneumostome; **in**, intestine; **ir**, insertion of m4 in tissue on radula (to) and m7a; **iv**, intestinal transverse fold; **jw**, jaw; **ki**, kidney; **kl**, kidney lobe; **m1–m10**, extrinsic and intrinsic odontophore muscles; **mb**, mantle border (edge); **me**, ommatophore muscle; **mf**, mantle fold; **mi**, micro muscular pallial longitudinal fibers; **mj**, jaw and peribuccal muscles; **ml**, pallial muscle; **mo**, mouth; **mp**, muscular wall of penis; **mr**, membrane surrounding radular sac; **mt**, mantle; **mu**, prerectal muscle; **ne**, nephropore; **nr**, nerve ring; **oc**, odontophore cartilage; **od**, odontophore; **om**, ommatophore; **on**, optical nerve; **ou**, ommatophore muscle; **pb**, penis bulged portion; **pc**, pericardium; **pe**, penis; **pf**, penis inner fold(s); **pg** pedal gland; **pl**, pleural ganglia bridge; **pm**, penis muscle; **pn**, pneumostome; **pp**, pedal ganglion; **pr**, penis aperture; **ps**, penis shield; **pt**, prostate; **pu**, pulmonary cavity; **pv**, penis inner transverse fold; **ra**, radula; **rn**, radular nucleus; **rs**, radular sac; **rt**, rectum; **sa**, salivary gland aperture; **sd**, salivary gland duct; **se**, septum between esophagus and odontophore; **sg**, salivary gland; **sp**, sperm inner longitudinal fold; **sr**, seminal receptacle; **st**, stomach; **su**, anal sulcus; **sy**, statocyst; **tg**, integument; **tm**, tentacle muscle; **to**, tissue on radula et end of radular sac; **ty**, typhlosole; **ua**, urinary aperture; **ug**, external urinary gutter in head-foot; **um**, umbrella-like transverse penis fold; **un**, union of mantle border with nuchal surface; **up**, primary ureter; **ur**, urinary gutter; **us**, secondary ureter; **ut**, uterus; **va**, vagina; **vd**, vas deferens; **ve**, ventricle; **vf**, vaginal fold; **vg**, vagina; **vm**, visceral mass; **wo**, parasite worm.

Additionally in the text, the following abbreviations are used: **L**, length; **sh**, empty dry shell; **spm**, complete specimen (shell and soft parts); **W**, width. Institutions: **MNRJ**: Museu Nacional da Universidade Federal do Rio de Janeiro; **MZSP**: Museu de Zoologia da

[illegible]versidade de São Paulo; **NHMUK**, Natural History Museum, London; **USNM**: National [illegible]seum of Natural History, Smithsonian Institution.

Results

Comparative conchology and anatomy

Systematics. Genus *Kora* Simone, 2012

[illegible]petraeus: Salgado & Coelho, 2003 [7]: 134 (non Martens, 1885).

Kora Simone, 2012 [1]: 432; 2015 [2]: 51; Salvador & Simone, 2016 [3]: 2.

Diagnosis. Shell fusiform to obese; with brownish, relatively uniform color, subsutural pale band. Sculpture axial undulations only, with minute spiral aligned pits in some areas. Protoconch of ~2 smooth whorls, some axial sculpture in last whorl sometimes present. Umbilicus usually well-developed, resulted of columellar hollow area, producing inner lip usually with middle region bulged. Secondary pair of columellar muscles with medial differentiated branch. White pallial gland. Ureter is totally closed (tubular). Radular sac bulging posteriorly in buccal mass, covered by translucent membrane (mr). Radula as numerous hook-like teeth, with blunt tip, almost no difference among rachidian, lateral and marginal teeth. Odontophore pair m8 present; ventral tensor muscle of radula lost. Base of bursa copulatrix and penis thick muscular. Accessory albumen chamber present. Penis divided into compartments, with pair of longitudinal inner folds. Spermatophore with chitinous basal tube. Calcified epiphragm.

List of included species. *Kora corallina* Simone, 2012 (type species by M & OD); *K. nigra* Simone, 2015; *K. rupestris* Salvador & Simone, 2016; *K. tupan* new species; *K. ajar* new species; *K. aetheria* new species; *K. jimenezi* new species; *K. kremerorum* new species; *K. curumim* new species; *K. vania* new species; *K. uhlei* new species.

Taxonomic discussion. see Discussion ~~item~~.

***Kora corallina* Simone, 2012** Figs 1–6

Kora corallina Simone, 2012 [1]: 432–433 (figs 1–8); 2015 [2]: 51, 53–55 (fig. 14); Salvador & Simone, 2016 [3]: 2–6 (fig. 2); Cavallari et al., 2016 [20]: 15 (fig. 5); MolluscaBase, 2023 [9].

Types. Holotype MZSP 103910; Paratypes: MZSP 103911, 1 shell; MZSP 103912, 1 shell, USNM, 2 shells; MNRJ, 2 shells; NHMUK, 2 shells; MZSP 103913, 32 shells; all from type locality (all examined).

Type locality. BRAZIL. Bahia; Santa Maria da Vitória, ~13°24'S 44°12'W, ~460 m of elevation (Coltro col., i/2012).

Diagnosis. Size about 45 mm, ~2.3 times longer than wide; lacking dorso-ventral compression. Apex with light color. Subsutural lighter band present. Peristome white. Delicate spiral striae present. Aperture occupying ~44% of length and ~70% width. Implantation of outer lip slightly vertical. Inner lip with high middle fold. Umbilicus wide. Secondary columellar muscles with 13 insertions in left and 7 in right. Two pairs of [illegible]v. Jaw with central notch. Odontophore

cartilages ~75% fused. Pair m10 narrow. Carrefour duct wide and short, inserted in duct of albumen gland. Albumen chamber in curve. Penis ~70% of spermoviduct length, lacking umbrella-like fold; epiphallus ~13% of penis length; penis muscle at epiphallus tip.

Redescription. Shell. (Figs 1, 2A–J) Proper description in [1]. Complement: length up to 45 mm, outline fusiform, elongated, ~2.3 longer than wide. Color yellowish-white (Figs 1A–B), beige (Figs 1F–H, J–N), or brown (Figs 2A–E) in first whorls, gradually becoming darker towards past whorl, with brown pigment, particularly dark in last whorl (Figs 1G, K, M); peristome white. Protoconch (Figs 1H, 2F) with ~2 whorls, bluntly pointed; first whorl smooth, axial riblets gradually appearing in second whorl. Callus low, weak (Figs 1A, C, F, J, L, 2A). Aperture with inner lip bearing strong oblique, middle, wide fold (Figs 1D, N, 2C), almost forming stubby plica. Umbilicus broadly (Fig. 1I) to narrow (Fig. 2D) opened, partially covered by inferior half of inner lip.

Epiphragm. Present in few specimens (Figs 1C–E, 2J) calcified, thick, occluding entire aperture; dislocated posteriorly from peristome.

Head-foot. (Fig. 3B) of normal shape. Color uniformly pale beige. Columellar muscle thick, 1.5 whorls in length. Inner arrangement of columellar annexed muscles relatively complex. Main columellar bundle (cm) occupying ventral floor of haemocoel, relatively flattened, wide ~3/4 of foot width. Pair of secondary columellar/cephalic muscles, each of which with ~half of main columellar bundle (cm) width. Both with multiple, aligned, similar-sized origins, flanking floor of haemocoel, edging at short distance pedal gland (pg) and mouth (mo); away from each other anteriorly, approaching from each other posteriorly, at ~1/3 of columellar muscle length. Anterior most branch short, as ommatophore retractor muscle (me); second small branch of right secondary muscle as genital muscle (gm); left secondary muscle (cl) with 13 branches, being posterior branch slightly broader; right secondary muscle (cr) with 6–7 branches, being posterior branch very broader. Both secondary columellar muscles attaching to main bundle only in their posterior region, jointed to radular muscle (m2) attaching externally. Pedal gland (pg) weakly protruding in posterior region of buccal area.

Mantle organs. (Fig. 3A) Mantle border (mb) thick, lacking pigments. Pneumostome (pn) protected by simple right ventral flap (if), width ~1/5 of aperture length. Folds of mantle border weakly developed. Pneumostome (pn) ~1/8 of shell aperture length, bearing exclusively air entrance and urinary aperture (ua); flanked in internal edge by narrow urinary gutter. Anus (an) separate aperture located at right, adjacent to pneumostome. Lung of ~1.5 whorls in length, ~twice long than wide, possessing minute longitudinal muscle fibers in its wall seen by translucency; right side from pulmonary vein ~twice wider than left side. Pulmonary venation well-developed, especially in region preceding pneumostome; posterior region of pulmonary vein (cv) protruded; left 2/3 only pair of intercalated longitudinal vessels; right 1/3 mostly having perpendicular vessels rather uniformly distributed, weak posterior, becoming taller anteriorly; in region preceding pneumostome pulmonary vessel bifurcating, bearing radial arrangement of secondary vessels. Pulmonary vein (cv) running longitudinally across pallial cavity roof. Reno-pericardial area of beige color, slightly triangular, located posteriorly within pallial cavity, its posterior abutting wall of visceral cavity, occupying ~20% of cavity length and ~55% of its width (details below). Rectum (rt) wide. Primary (up) and secondary (us) ureters entirely closed (tubular), relatively narrow, aperture (ua) simple, directly outside at right in pneumostome region.

Visceral mass. (Fig. 3C) ~2.5 whorls in length. Both digestive gland lobes greenish beige in color; anterior lobe (da) flattened, occupying ~1/5 of visceral volume, located just posteriorly to pallial cavity, continuous to kidney. Posterior lobe (dg) larger, extending 2 spiral whorls, occupying ~50% of visceral volume. Stomach (st) ~1/10 of visceral volume, located between both digestive gland lobes, ~3/4 whorl posterior to pallial cavity. Digestive tubes (described below) proportionally large. Gonad multi-lobed, cream color, encased between posterior lobe of digestive gland and columella, occupying ~1/3 of last whorl, ~1/15 of visceral volume.

Circulatory and excretory systems. (Fig. 3A) Pericardium (pc) ~twice as long as wide, located obliquely between middle and left thirds of posterior end of pallial roof, appressed against right lateral side of kidney; occupying ~5% of lung area. Auricle (au) located anteriorly, as continuation from pulmonary vein (cv); ventricle (ve) located posteriorly, larger. Kidney (ki) simple, weakly dorso-ventrally flattened; size reported above; slightly triangular, width ~1/2 of length; internally organized as successive tall glandular folds (kl), being taller in dorsal wall, and shorter in wall with visceral mass; central region hollow. Nephropore small, longitudinal slit at anterior-left corner of kidney, directed towards right, inside anterior end of primary ureter.

Digestive system. (Figs 3C, 4A–C) Mouth (mo) and oral tube (mj) wide, short, thick muscular. Jaw plate (Figs 2G–H) thick, yellow; cutting edge convex, notched at middle or chevron-like; sculptured by successive, rather uniform, transverse, wide folds. Buccal mass elliptic, occupying~1/5 of haemocoel volume; being ~1/4 of its posterior bulged chamber, sheltering coiled radular sac (rs), covered by transparent membrane (mr). Dorsal surface of oral cavity with well-developed pair of dorsal folds (Fig. 4A: df), width of each ~1/2 of dorsal wall width; touching with each other in median line. Odontophore (Fig. 3C: od) with ~50% of buccal mass volume. Odontophore muscles (Figs 4A–C): **mj**, jaw and peribuccal muscles originating in outer-ventral surface of odontophore cartilages (Fig. 4B), running towards ventral making platform covering ventral surface of cartilages (oc), afterwards splaying in dorsal wall of oral tube; **m1**, jugal muscles covering entirely haemocoelic structures, more concentrated close to mouth; **m1v**, two pairs of barrow ventral protractors jugal muscles (Fig. 3C), originating in ventral surface of haemocoel close to mouth, running towards posterior, inserting in ventral-posterior region of odontophore close to m2 insertion; **m2**, radular muscle, or strong pair of retractor muscles of buccal mass, originating as single bundle in columellar muscle posterior end (Fig. 3B), running anteriorly close to median line along ~60% of haemocoel length, becoming broader, inserting as two different bundles (Fig. 4C) connected medially (Fig. 4B) in ventro-posterior edge of odontophore, surrounding at some distance radular nucleus; **m3**, pair of thin longitudinal fibers immersed in posterior wall of odontophore (mr), between esophageal origin and radular nucleus; **m4**, main pair of dorsal tensor muscles of radula, very thick, originating in postero-medial region of odontophore cartilages, surrounding outside and medially cartilages, inserting in subradular membrane in its region correspondent to buccal cavity; **m5**, pair of auxiliary dorsal tensor muscles of radula, also thick, originating on postero-ventral region of odontophore cartilages, running towards anterior covering cartilages, inserting in subradular membrane along radular exposed (in buccal cavity) region; **m6**, horizontal muscle minute, only detectable in short portion (~10% of cartilage length) posterior to fusion between both cartilages; **m7**, narrow and slender, originated splayed in posterior region of fusion between both cartilages, run inside dorsal region od radular sac (Fig. 4C); **m7a**, thick muscular bundle inside ventral stored portion of radula in radular sac, in which part of pair m4 inserts (Fig. 2I: ir); **m8**, pair of wide, superficial muscles in lateral edge of cartilages, longer laterally, shorter in internal dorsal (Fig. 4C) and ventral (Fig. 4B) ends; **m10**, pair of narrow ventral odontophore protractor muscles, originating in ventro-

anterior region of haemocoel, just ventral to mouth, running towards posterior covered by m1v, inserting in latero-posterior surface of odontophore close to m2 insertions; **m11**, pair of narrow ventral tensor muscles of radula absent. Odontophore non-muscular structures: oc, pair of odontophore cartilages flattened, rather elliptical, slightly rectangular, ~1.5 times longer than wide, fused with each other along ~75% in their anterior-medial edge, posterior end roughly rounded; **sc**, subradular cartilage, with expanding region in buccal cavity protecting subradular membrane. Radular sac (rs) long, performing loop inside translucent membrane (mr) bulded posteriorly from odontophore (Fig. 3C).

Radula. (Figs 2I, 5) ~1.5 times longer than odontophore. Composed of uniform, similar kind of tooth, with no clear differentiation among rachidian, lateral or marginal teeth, ~250 ~~pairs of~~ teeth per row (Fig. 5A). Each tooth with elongated base, 6–7 times longer than wide, placed longitudinally, very close to each other; proximal end rounded, slightly elevated (Figs 5B, C, D); distal end bearing long, curved cusp, slightly longer than base; base reinforced by central fold gradually tapering up to ~70% of cusp length (Fig. 5C); cusp tip flattened, slightly broader, rounded-barely spoon-like, possessing shallow subterminal, longitudinal, short furrow (Figs 5C, E). All teeth straight aligned per row, except for ~20 more marginal teeth, slightly arched aligned (Figs 5C, F).

Salivary glands small, covering ~1/10 of esophagus length, located between anterior and second quarter of esophageal length (Fig. 3C: sg), forming two elliptic, white, thin masses. Each salivary duct differentiable in middle and anterior side of glands, with ~1/12 of esophageal width (sd). Salivary duct running in both sides of esophageal origin, penetrating buccal mass wall in region close to buccal ganglia (bg), running immersed in buccal dorsal wall along ~1/3 its length (Fig. 4A). Salivary ducts opening as small pores (sa), located in middle level of posterior region of lateral edges of wide dorsal folds. Esophagus ~1-whorl long, with firm walls (Fig. 3C: es); anterior region with narrow, tall longitudinal, uniform folds; posterior third weakly broader, smooth surface. Stomach (st) narrow, curved, weakly bulging; position and size described above (visceral mass); gastric walls thin, weakly muscular; inner surface mostly smooth, lacking folds. Esophageal insertion on right side, intestinal origin on left side, both close to columella. Duct to anterior lobe of digestive gland at short distance from esophagus and intestine intersection (dd) broad, running towards anterior, possessing secondary successive branches along both sides along ~1/3 whorl immersed in digestive gland. Duct to posterior lobe of digestive gland located short distance from intestinal origin, slightly posterior and at right to above-described duct, directed towards opposite side (dp), slightly narrower and possessing only branches in right side. Intestine (Fig. 3C: in) initially narrower than esophageal insertion, maintaining this caliber along its entire length; performing its usual wide sigmoid loop in anterior lobe of digestive gland; its anterior region having pair of narrow typhlosoles close from each other, from stomach level, up to pericardium level (ty). Rectum and anus position described above (pallial cavity) (rt, an). Anus sessile, as slit in right end of mantle edge directly turned outside.

Reproductive system. (Figs 4E, D, 6A–C) Gonad position described above (visceral mass), composed of 5–6 lobes with minute digitiform acini. Hermaphroditic duct (Fig. 4E: hd) narrow; coiled portions occupying middle 2/3, with narrow coils; insertion preceded by straight region, and strongly curve (Fig. 5B: hd). Seminal receptacle (Figs 4E, 6B: sr) small, straight, long, tapering gradually, ~10 times longer than wide, flattened. Fertilization complex or carrefour (Fig. 6B: ca) simple, as wide region in receptacle base, ~1/3 of its length; totally immersed in albumen gland, tapering abruptly up to very narrow duct inserting in posterior end of

spermoviduct, at side of tip of wide albumen gland duct. Albumen gland (ag) solid, white, elliptical, ~5 times larger than gonad (~1/3 whorl). Albumen gland duct subterminal, connected to distal end of spermoviduct (Fig. 6B: ad), continuing as small, curved albumen chamber (Fig. 6B: ac); narrowly connected to distal end of spermoviduct (eo). Spermoviduct (Figs 4E, 6B: eo) of ~1.5 whorl in length, slightly narrower than albumen gland, ~20 times longer than wide. Secondary albumen chamber (as) ~4-times larger than primary chamber (described above), connected to spermoviduct some distance anterior to it by narrow duct (Fig. 6B). Prostate wide (pt), ~1/3 of spermoviduct diameter (Fig. 4D); uterus with weakly glandular walls, highly, transversally, and relatively uniformly folded (Figs 4D–E, 5A–B: ut). Sperm inner longitudinal fold (Figs 4D, 6A: sp) as simple, tall, thick fold, a second small fold gradually appearing in basal third; both folds fusing with each other, originating vas deferens, slightly anterior to end of uterine level (Fig. 6A: vd). Vas deferens uniformly narrow, uncoiled (Figs 4E, 6A: vd). Genital muscle in intersection vagina and penis (Figs 4E, 6A: gm). Bursa copulatrix (bc) and its duct (bd) of usual position, with ~90% of spermoviduct length (Fig. 4); bursa duct with basal third with walls extraordinarily thick muscular (Fig. 6A: bu). Free oviduct (fo) and vagina (vg) simple, possessing respectively 2 and 4 wide, low, longitudinal, simple folds (Fig. 6A). Penis slightly coiled, ~70% of spermoviduct length if straightened (Fig. 4E: pe); epiphallus as continuation of penis, penis muscle inserted at epiphallus' tip (Figs 4E, 6C: pm), short, simple. Penis shield (Figs 4E, 6A, C: ps) with transverse muscle fibers, with ~1/7 penis length. Penis wall relatively muscular, especially in region adjacent to penis shield (mp). Epiphallus (eh) ~1/8 of penis' length, amply opened to penis; only vas deferens insertion marking its limit (Fig. 6C: vd). Epiphallus inner surface with single high longitudinal fold (ei) and 8–10 secondary small, parallel folds (Fig. 6C: eh). Internal penial arrangement of folds clearly with three regions (Fig. 6C): (1) basal third, possessing only 3–4 longitudinal, broad, low, simple folds; (2) middle third, with strong pair of tall, longitudinal folds in a side, each one with simple external margin, and successive small secondary branches in internal margin, imbricating with its counterpart, this strong pair basal end fading, distal end rounded; mosaic of low, oblique, rather irregular folds flanking both strong folds; (3) distal third with basal region smooth, and 8–10 longitudinal folds similar to those from epiphallus gradually appearing; some of them, including one of them slightly larger, converge to vas deferens aperture (Fig. 6C: vd).

Central nervous system. (Fig. 6D) Nerve ring located in anterior half of buccal mass. Pair of cerebral ganglia located very close with each other, commissure extremely short; each cerebral ganglion elliptic, with ~1/10 buccal area's size. Pair of cerebral nodes, or glands (cd), with ~1/8 each ganglion's size. Pair of cerebro-pedal and cerebro-pleural connectives (cn) slender, long (~2.5 times longer than each cerebral ganglion), similar-sized, running close from each other. Pair of pleural ganglia (pl) connected with each other by short commissure slightly dislocated to left, region possessing narrow blood vessel (bv); both pleural ganglia broadly connected to pedal ganglia in their outer region. Pair of pedal ganglia (pp) located close from each other, commissure extremely short; each pedal ganglion rather spheric, ~1.5 times larger than each cerebral ganglion. Pair of statocysts (sy) located in ventral surface of pedal ganglia, each one with ~1/15 of each pedal ganglion's size; internally with several rounded, crystalline statoconia.

Distribution. *K. corallina* so far has been only found in a Bahia region west from São Francisco River, more or less defined by the quadrilateral Santa Maria da Vitória – São Félix do Coribe – Serra do Ramalho – Taquarinópolis.

Habitat. Under rocks, limestone areas.

Measurements. (in mm) MZSP 132078 (Figs 1C–E): 44.8 by 21.2; MZSP 151952#1 (Figs 1F–G) 35.5 by 19.6; MZSP 151952#2 (Figs 1J–K): 44.8 by 20.1; MZSP 151952#3 (Figs 2A–D): 40.0 by 19.3; MZSP 125175 (Figs 1L–N): 37.0 by 17.1.

Material examined. All types. BRAZIL (W Vailant-Mattos col.). **Bahia**. Santa Maria da Vitória (topotypes), 13°28'S 44°13'W, MZSP 132078, 5 spm (vi.2016); São Félix do Coribe, 13°26'12"S 44°12'21"W, MZSP 151952, 19 shells (4.iv.2020); Serra do Ramalho, Toca, 13°38'13"S 43°50'10"W, MZSP 152175, 25 shells, 152223, 1 shell (v.2019); Taquarinópolis, 13°32'44"S 43°50'28"S, MZSP 151808, 9 shells (v.2019).

Taxonomic remarks. See Discussion ~~item~~.

***Kora nigra* Simone, 2015** Figs 7–11

Kora nigra Simone, 2015 [2]: 3 (figs 6–13, 21); Salvador & Simone, 2016 [3]: 1–6 (fig. 3), 2021 [21]: 396–397 (fig. 2A–B); Cavallari et al., 2016 [20]: 15 (fig. 6); Salvador et al., 2022 [22]; 5 (fig. 18); MolluscaBase, 2023 [9].

Types. Holotype MZSP 106232; Paratypes: MZSP 106241, 1 shell; 106250, 2 shells, 104831, 5 shells; all from type locality (all examined).

Type locality. BRAZIL. **Bahia**. Carinhanha, Serra do Ramalho, Gruna do Cesário, 14°19'S 43°47'W (Bichuette col., 12.ix.2008).

Diagnosis. Size about 30 mm, ~1.6 times longer than wide; lacking dorso-ventral compression. Apex with same color as remaining shell. Subsutural lighter band present. Peristome with brown spots. Delicate spiral striae present. Aperture occupying ~48% of length and ~60% width. Implantation of outer lip slightly horizontal. Inner lip with high middle fold. Umbilicus wide. Secondary columellar muscles with 7 insertions in left and right. Two pairs of m1v. gastric posterior duct Y-shaped. Jaw rectangular. Odontophore cartilages ~60% fused. Pair m10 narrow. Carrefour duct narrow and long, inserted between albumen gland and accessory albumen chamber. Albumen chamber sac-like. Two sperm folds in spermoviduct. Penis ~100% of spermoviduct length, lacking umbrella-like fold; epiphallus ~20% of penis length; penis muscle at epiphallus tip.

Distinctive redescription. Shell. (Figs 7, 8A–C) Proper description in [2]. Complement: maximum length up to 43 mm, vast majority ~30-35 mm; outline fusiform-obese, ~1.8x longer than wide. Color dark (Figs 7A–O) to light brown (Figs 8A–C), with narrow white subsutural band (Figs 7B, I, K, N, 8B); peristome white with some brown tinting in different degrees (high: Fig. 7A, lower: Figs 7C, J, M, 8A). Apical view rounded (Fig. 7G). Protoconch (Fig. 7F) with ~2 whorls, wide; first whorl smooth, weak axial riblets gradually appearing in second whorl. Callus weak (Figs 7D, E, L, O). Aperture with inner lip bearing strong oblique, middle, slightly narrow fold (Figs 7E, L, O, 8C), almost forming stubby plica. Umbilicus broadly opened (Fig. 7H), partially covered by inferior half of inner lip.

Head-foot. (Fig. 8D, 9B) similar character as preceding species, remarks and distinctions following. Integument with clear (urinary?) sulcus from pneumostome up to genital aperture (Fig. 8D: ug). Columellar muscle also divided into three bundles. Main columellar bundle (cm) slightly narrower, with ~1/2 foot width. Left secondary columellar/cephalic muscle (cl) with ~half of main columellar bundle (cm) width; with 7 transversely aligned narrow anterior

branched, medial one larger, inserted more posteriorly in haemocoelic floor, remaining aligned perpendicularly from floor to left wall, being 2 dorsal ones longer than remaining, and ommatophore muscle as last one (me). Right secondary columellar/cephalic muscle (cr) similar to left one, but ~half its width; anteriorly only 5 branches, medial branch wide and more posteriorly inserted, remaining branches more anteriorly inserted on right wall, 2 more dorsal branches as ommatophore (me) and genital (gm) muscles. Pedal gland (pg) weakly protruding in posterior region of buccal area.

Mantle organs. (Fig. 9A) characters similar to those of preceding species, interesting features following. Mantle border (mb) with pair of small left and right folds (mf), with middle end pointed. Pulmonary venation well-developed, especially in region preceding pneumostome at along almost half of pulmonary cavity; left 2/3 with 3 longitudinal vessels draining to pericardial region; right 1/3 mostly having perpendicular weak vessels rather uniformly distributed, becoming taller only in close region preceding pneumostome; left region of pulmonary vein (cv) with set of oblique, successively larger vessels, located very close from each other, interspaces bearing transverse secondary vessels. Pulmonary vein (cv) broad, particularly in both ends. Reno-pericardial broadly triangular, occupying ~15% of cavity length and ~60% of its width (details below). Rectum (rt) wide. Urinary aperture (ua) simple, directly outside at right in pneumostome region, almost in continuation to anus (an).

Visceral mass. General features similar to preceding species.

Circulatory and excretory systems. (Fig. 9A) Features similar to preceding species (check proportions above), except for slightly narrower heart and pericardium (pc), broader kidney (ki), and by kidney lobe being single, U-shaped glandular mass (check detail of transverse section on Fig. 9A).

Digestive system. Most characteristics as those described for *K. corallina*, remarks and distinctions following. Jaw plate (Fig. 8E) slightly thinner, yellow; cutting edge weakly convex, with small notch at middle. Buccal mass also with similar attributes; oral cavity with dorsal folds slightly taller, especially in their posterior region; aperture of salivary glands equally positioned, but wider. Odontophore also with same characters, except for having minor fusion degree between both odontophore cartilages – ~60% fused. Radular sac also sheltered inside bulged translucent sac. **Radula.** (Fig. 10) ~2 times longer than odontophore. Structure and characters as those described for *K. corallina*. Except in having slightly more pairs of teeth, ~300 pairs per row (Figs 10A, D), and each teeth slightly more robust, being weakly broader, with shorter cusp (Figs 10B, C, E, F). Stomach (st) (Fig. 9C) also with similar features, including position of ducts to both lobes of digestive gland; anterior duct (dd) much shorter, possessing only 4 strong branches; posterior duct (dp) also shorter, bifurcated in its base.

Reproductive system. (Figs 9D, 11) general organization similar to preceding species, remarks and distinctions following. Gonad proportionally larger (~1/2 whorl), composed of 8 lobes. Hermaphroditic duct (Fig. 11A: hd) intensely coiled, mainly in region at some distance from its insertion, having some broader loops (Figs 11A, C); short portion very narrow preceding its insertion (Fig. 11C). Seminal receptacle (Fig. 11C: sr) very small, straight, with uniform width along its length, ~5 times longer than wide, flattened. Fertilization complex or carrefour (Fig. 11C: ca) simple, wide, ~twice longer than receptacle and 3-times its width; receptacle and hermaphroditic duct inserting in its distal tip; bluntly tapering in basal end up to its insertion in beginning of albumen chamber (Fig 11C: ac). Duct of albumen gland (Fig 11C: ad) narrow, inserted in albumen chamber by side of that of carrefour. Albumen chamber

relatively large, as bifid chamber (Fig. 11C: ac); narrowly connected to distal end of spermoviduct (eo). Spermoviduct (Figs 11A, C: eo) ~25 times longer than wide. Secondary albumen chamber (as) weakly larger than primary chamber (described above), connected to spermoviduct some distance anterior to it by narrow duct (Fig. 11C). Prostate wide (pt), ~1/3 of spermoviduct diameter (Fig. 9D); uterus with weakly glandular walls, highly, transversally, and uniformly folded (Fig. 11A: ut). Sperm inner longitudinal fold (Fig. 9D: sp) with simple, tall, thick primary fold, and second small fold all along spermoviduct length. Remaining features of basal region of genital structures similar to preceding species, except for uterus having more uniform and narrow folds, and by duct of bursa copulatrix having taller and more irregular folds in its base. Penis proportionally longer, almost as long as spermoviduct if straightened (Fig. 11A: pe). Penis shield (ps) with ~1/10 penis length. Penis with especial thick muscular region in its basal third. Epiphallus (eh) ~1/4 of penis' length, amply opened to penis; vas deferens insertion and abrupt narrowing marking its limit (Fig. 11A: vd). Epiphallus inner surface with ~8 uniform, narrow, parallel folds (Fig. 11B: eh); its basal end bulging inside penis distal end, papilla-like, partially covering vas deferens aperture (Fig. 11B: vd). Internal penial arrangement of folds also clearly with three regions (Fig. 11B): (1) basal third, corresponding to strongly muscular region, with smooth surface; (2) middle third, with strong pair of tall, simple, longitudinal folds in a side, smooth area between both; small, narrow, longitudinal secondary folds surrounding both main folds; these folds suddenly appearing just after basal muscular portion, fading in region preceding distal third; (3) distal third region almost smooth, only divergent/coalescent, weak, narrow wrinkles.

Spermatophore. (Figs 8F–G) found inside duct of bursa copulatrix. Chitinous walls, color greenish-yellow. Having 2 portions: (1) basal portion, ~70% of its length, slender hollow stem; walls longitudinally folded, translucent, relatively hard; basal aperture irregular; superior region gradually broadening, with smooth walls; (2) distal portion, ~30% of its length, bulged, elongated sperm chamber; tip flaccid, sharp pointed, irregular; base connected to stem in a side, with irregular, disform aperture in other side; walls relatively firm, weakly flattened.

Central nervous system. Same characters as preceding species.

Distribution. *K. nigra* has so far been found both, in Bahia and Minas Gerais, but in regions close from each other, close to their border and very close to São Francisco River, in the region of Serra do Ramalho, Bahia, and in region of Itacarambi, Minas Gerais.

Habitat. Under rocks, limestone areas.

Measurements (in mm): MZSP 106230#1 (Figs 6J–K): 40.6 by 23.2; 106230#2 (Figs 6C–E): 41.8 by 22.4; MZSP 151827 (Figs 6M–O): 43.3 by 24.5; MZSP 151830 (Figs 6aA–C): 39.9 by 22.5.

Material examined. All types. BRAZIL (W Vailant-Mattos col.). **Minas Gerais**. Itacarambi, Serra de Itacarambi, 15°01'42"S 44°13'15"W, MZSP 151830, 10 shells (i.2020), Fazenda ICIL, 15°00'39"S 44°03'24"W, MZSP 151827, 4 shells, MZSP 151828, 4 spm (i.2020).

Taxonomic remarks. See Discussion item.

***Kora rupestris* Salvador & Simone, 2016** Figs 12–15

Kora rupestris Salvador & Simone, 2016 [3]: 1–6 (figs 4–11, 15–17), 2021 [20]: 396–397 (figs 2A–B); Simone, 2022 [23]: 44 (fig. 7); MolluscaBase, 2023 [9].

Kora sp: Cavallari et al., 2016 [20]: 15 (fig. 7);

Types. Holotype MZSP 121416; Paratypes: MZSP 121441, 2 shells from type locality (all examined).

Type locality. BRAZIL. Bahia; Carinhanha, Canabrava Hill, ~14°18'18"S 43°45'54"W (A. Bianchi col., viii/2012).

Diagnosis. Size about 45 mm, ~2.3 times longer than wide; lacking dorso-ventral compression. Apex with light color. Subsutural lighter band present. Peristome white. Delicate spiral striae present. Aperture occupying ~50% of length and ~66% width. Implantation of outer lip slightly vertical. Inner lip with high middle fold. Umbilicus wide. Secondary columellar muscles with 7 insertions in left and in right. Two pairs of m1v; additional pair m3 close to m2 insertion. Jaw with central notch. Odontophore cartilages ~75% fused. Pair m10 narrow. Carrefour duct narrow and long, inserted in tip of spermoviduct. Albumen chamber sac-like. Penis ~60% of spermoviduct length, umbrella-like fold present, with 5 rods; epiphallus ~15% of penis length; penis muscle at epiphallus base.

Distinctive redescription. Shell. (Figs 12A–K) Proper description in [3]. Complement: length up to 46.5 mm, outline rather conic, elongated, ~2.2x longer than wide. Transverse section circular (Fig. 12G). Color orange (Figs 12A–B) to brown (Figs 12C–H), subsutural pale band (Figs 12C, F, I–K); peristome completely white (Figs 12A–B) or with outer lip brown (Figs 12C, F, I, K). last whorl having only narrow axial undulations and growth lines (Fig. 12B) or associating minute spiral striae more (Figs 12D, E) or less (Fig. 12J) dense. Callus low, weak (Figs 12A, C, F, I, K). Aperture with inner lip bearing strong oblique, relatively tall, narrow fold (Figs 12F, K), almost forming stubby plica. Outer lip implantation more vertical shaped (Fig. 12A), or more horizontal implanted (Fig, 12I), with intermediaries (Fig. 12C). Umbilicus narrowly opened (Fig. 2H), partially covered by inferior half of inner lip.

Head-foot. With similar features as *K. nigra*. Except for left secondary columellar muscle slightly broader; and by pedal gland deeper and more developed.

Mantle organs. (Figs 12M, 13A) most features similar to *K. corallina*, distinctions and remarks following. Mantle border (mb) with pair of secondary folds (mf) small and pointed, located in opposed sides. Pulmonary venation weakly poorly developed, including in region preceding pneumostome, possessing only transverse, low vessels. Pulmonary vein (cv) running longitudinally across pallial cavity roof. Pallial wall in entire pulmonary cavity with uniform cover of minute longitudinal muscle fibers (Fig. 12M: mi). Primary (up) and secondary (us) ureters entirely slightly broader.

Visceral mass. With same characters as *K. corallina*.

Circulatory and excretory systems. (Fig. 13A) general characteristics as those described for *K. corallina*, except for pericardium (pc) slightly narrower if compared to kidney; and for arrangement of kidney lobe, as 4 successive, transverse, tall folds.

Digestive system. (Fig. 13B) overall morphology similar to that of *K. corallina*. Differences and remarks following. Peribuccal circular muscles (mj) longer and thicker. Jaw plate (Figs 12L) strongly notched at middle; sculptured by successive, rather uniform, transverse, wide folds. Buccal cavity with esophageal folds (ef) slightly taller and dark pigmented, having salivary apertures (sa) in their outer-anterior edges. Odontophore intrinsic, extrinsic muscles, and other structures similar to *K. corallina*, except for **m1v**, pair single and wider; **m1l**, narrow pair

of jugal lateral protractor muscles, originating in lateral region of mouth, running towards posterior, inserting in lateral region of buccal mass posterior end; **m3**, pair short and wide, originating in inner insertion of m2, shortly inserting along membrane surrounding radular sac; **m10**, broader and more visible. **Radula** (Fig. 14) with same attributes as *K. corallina*, except for tip of teeth slightly more rounded, with subterminal furrow shorter (Figs. 14B, D); and marginal teeth slightly more arched (Fig. 14E). Stomach with anterior duct to digestive gland slightly narrower.

Reproductive system. (Figs 12N–O, 13C–D, 15) general structures similar to preceding species, remarks and distinctions following. Gonad (go) proportionally larger, not clearly divided in lobes (Fig. 12P). Hermaphroditic duct (Figs 13C, 15A: hd) intensely coiled, particularly in its half close to its insertion; except for short narrow region preceding its insertion (Fig. 15A). Seminal receptacle minuscule (Figs 15A: sr), straight, long, tapering gradually, ~8 times longer than wide, tip rounded. Fertilization complex or carrefour (Fig. 15A: ca) as simple, long, relatively wide duct, lacking bulged regions; inserting directly in spermoviduct beginning. Albumen gland (ag) ~5 twice larger than gonad. Albumen gland duct (Fig. 15A: ad) subterminal, connected to spermoviduct as narrow, separated duct. Albumen chamber (ac) as blind sac, ~twice longer than wide, connected to spermoviduct beginning by narrow region, jointed to duct of albumen gland. Secondary albumen chamber (as) ~3-times larger than primary chamber, with its same length, broad, abruptly tapering in its insertion at short distance from albumen chamber insertion. Spermoviduct (eo) slightly narrower, slender ~30 times longer than wide. Prostate wide (pt), ~1/2 of spermoviduct diameter (Fig. 12D); uterus with glandular walls, highly, transversally, and relatively uniformly folded (Fig. 13C: ut). Sperm inner longitudinal fold (Fig 13D: sp) simple, low, thick fold, a second small fold gradually appearing in basal third; both folds fusing with each other, originating vas deferens, slightly anterior to end of uterine level (Fig. 12N: vd). Genital muscle in two bundles attached to vaginal outer wall (Fig. 13C: gm). Bursa copulatrix (bc) and its duct (bd) almost as long as spermoviduct length (Fig. 13C); bursa duct with basal 2/3 with walls extraordinarily thick muscular (Figs 12N: bu, 13C). Basal region of duct of bursa copulatrix with special arrangement of irregular inner folds as shown in Fig. 12B: bd. Penis slightly mostly straight, ~60% of spermoviduct length if straightened (Fig. 12C: pe). Penis muscle inserted along lateral walls of epiphallus, leaving tip free (Figs 15B–C: pm), long, simple. Penis walls weakly muscular, except for region adjacent to penis shield (Figs 13C, 15C:mp). Epiphallus (eh) ~1/7 of penis' length, amply opened to penis; only vas deferens insertion marking its limit (Fig. 17C: vd) and weak sudden change of folds. Epiphallus inner surface only with 8–10 narrow, small, parallel folds (Fig. 15C: eh). Internal penial arrangement of folds clearly with 4 regions (Fig. 15C): (1) basal 1/4, highly muscular region (pm), possessing mosaic of low, uniform, longitudinal folds; (2) sub-basal 1/4, with strong pair of tall, longitudinal folds in a side, located close from each other, each one with simple margins; mosaic of low, longitudinal, rather irregular folds flanking both strong folds; (3) sub-distal 1/4, with basal region almost smooth, only having 4–5 oblique, low folds, separated from each other; **umbrella-like fold** (Fig. 15B: um) located distally to this region, tall and narrow, possessing 5 rods exceeding fold's edge, septum-like, inserted transversally in inner penis wall; (4) distal region, possessing only longitudinal folds, those more basal as continuation of umbrella-like fold rods, gradually fading posteriorly, becoming lower and more numerous; some of them converging to aperture of vas deferens (Fig. 15B: vd), other continuous with epiphallus inner folds.

Central nervous system. Same characters as *K. corallina*, the single detected difference is slightly shorter connectives.

Distribution. *K. rupestris* is the only species with a wide range. It occurs from Serra do Ramalho, Bahia (~13.5°S) up to Januária, Minas Gerais (~15°S), a range of ~250 km. It is also, the single species that occurs in both sides of the São Fransisco River, with a sample collected in Iuiú, a city in east side from São Francisco River (the others are in the west side). As the species' shell is very characteristic, and very distinct from the other species, and the access to complete specimens was very scanty (Table 1) (only samples from Januária, Minas Gerais were complete), it was not possible to change the co-specificity of all these populations. A conservative approach was so far adopted, based on the conchology only.

Habitat. Under rocks, limestone areas.

Measurements. (in mm) MZSP 121416#3 (Figs 9C–H): 46.1 by 20.2; MZSP 151891 (Figs 9I–K): 44.1 by 21.8.

Material examined. All types. BRAZIL (W Vailant-Mattos col.). **Bahia**. Cocos, Itaguari, 14°37'06"S 45°31'13"W, altitude 730 m, MZSP 151920, 8 shells (ii.2020); Iuiu, 14°25'50"S 43°33'50"W, MZSP 151765, 5 shells (i.2022); Serra do Ramalho, Pedreira, 13°32'44"S 43°50'28"S, MZSP 151956, 26 shells (v.2019). **Minas Gerais**. Januária, border of National Park Paruaçu, 14°57'55"S 44°04'21"W, MZSP 151890, 5 spm, MZSP 151891, 9 shells (i.2020), Barreiros, 15°28'47"S 44°22'03"W, MZSP 152132, 5 shells (iv.2020); Cônego Marinho, 15°19'15"S 44°27'52"W, MZSP 152163, 4 shells (iv.2020), Vale do Gala, 15°19'15"S 44°27'52"W, MZSP 152062, 5 shells (iv.2020)

Taxonomic remarks. See Discussion ~~item~~.

***Kora tupan* new species** Figs 16–20

ZooBznk: urn:lsid:zoobank.org:act:A8261BE4-E22E-41FF-B845-2C21DCCB273B.

Types. Holotype MZSP 161200, complete spm; paratypes: MZSP 151817, 3 spm, MZSP 151823, 1 shell, all from type locality.

Type locality. BRAZIL. **Minas Gerais**; São João da Ponte, Gruta do Índio, 15°50'44"S 44°00'03"W (W. Vailant-Mattos col., i.2020).

Diagnosis. Size about 55 mm, ~1.9 times longer than wide; dorso-ventrally slightly compressed. Apex with same color as remaining shell. Subsutural lighter band present. Peristome white. Delicate spiral striae present. Aperture occupying ~54% of length and ~70% width. Implantation of outer lip slightly horizontal. Inner lip with high middle fold. Umbilicus wide. Secondary columellar muscles with 8 insertions in left and 6 in right. pairs of m1v lacking. Jaw rectangular. Odontophore cartilages ~75% fused. Pair m10 broad, m7 as 3 bundles. Carrefour bearing bulged portion, duct narrow and long, inserted between duct of albumen gland and albumen chamber. Albumen chamber sac-like. Penis ~85% of spermoviduct length, umbrella-like fold present, with 3 rods; epiphallus ~20% of penis length; penis muscle at epiphallus tip.

Description (distinctive in anatomy). Shell. Length up to 56 mm, outline fusiform-globose, ~1.9 longer than wide. Color pale (Figs 16K, M) to middle brown (Figs 16A–D, H–J) in spire, gradually becoming dark brown in last whorl (Figs 16B, F, I, M); subsutural pale band in all whorls well-developed (Figs 16A, C, I, M). Protoconch (Figs 16G, L, 17B) with 2 whorls, bluntly pointed; length ~5% of shell length, and ~11% of shell width; mostly smooth, barely sculptured

by axial riblets in last whorl. Limit between protoconch and teleoconch weakly visible, weakly prosocline. Teleoconch of ~4.5 whorls successively and uniformly increasing; whorls weakly concave; suture weakly deep; sculpture absent, except for growth lines and delicate axial, uniform undulations, ~60 in penultimate whorl. Dorso-ventrally very softly flattened (Fig. 16E). Peristome weakly dislocated to right; deflected, except for region of callus. Callus weak (Figs 16A, D, H, J, K). Aperture wide, somewhat dislocated from spire longitudinal axis; length ~54% of shell length, ~70% of shell width. Outer lip inserted distantly from adjacent suture, simple, arched. Inner lip concave, superior half weakly convex, mostly showing outer surface of last whorl; inferior half weakly convex, concave only inferiorly; bearing oblique fold in limit with superior half, having weak elevation preceding its end in inner lip (Figs 16C, D, J); tooth length ~35% of peristome length. Umbilicus opened, narrow, partially covered by inferior half of inner lip (Fig. 16F).

Head-foot. (Fig. 18B) with similar features as *K. corallina*. Except for both secondary columellar muscles slightly narrower, and with fewer basal insertions, 6 in right (cr), 8 in left (cl); additionally pair of central-medial longer insertions slightly symmetrical sized.

Mantle organs. (Figs 17E, F, 18A) most features similar to *K. corallina*, distinctions and remarks following. Mantle border (mb) with large secondary fold (mf) at left from pneumostome (pn) with pointed left end. Pulmonary venation strongly developed, especially in region preceding pneumostome, where vessels touch each other. Pulmonary vein (cv) entirely broad; its anterior end with ramifications; those at right from it basically straight, more developed anteriorly and in region adjacent to kidney; those at left from it longer and more complex, with anastomosis with next main longitudinal secondary vessel at left from it (Fig. 18A). Region at left from pulmonary vein (cv) with single pair of long, longitudinal, intercalated vessels; collar vessel (co) also broad. Pallial wall in entire pulmonary cavity with uniform cover of minute longitudinal muscle fibers (Fig. 17E: mi).

Visceral mass. (Fig. 18C) with same characters as *K. corallina*. Except for digestive gland, with both lobes (anterior and posterior) connected with each other, and portion posterior to stomach slightly longer.

Circulatory and excretory systems. (Fig. 18A) general characteristics as those described for *K. corallina*, except for pericardium (pc) with about half its width; and for arrangement of kidney lobe, as only 2 broad, tall folds.

Digestive system. (Fig. 18C) overall morphology similar to that of *K. corallina*. Differences and remarks following. Peribuccal circular muscles (mj) longer and thicker. Jaw plate (Figs 17A, C) thick, rather rectangular, cutting edge only weakly concave; sculptured by successive, rather uniform, transverse, narrow folds. Buccal cavity (Fig. 17C) with esophageal folds (ef) taller and subfolded, having salivary apertures (sa) immersed in posterior end of deep furrow. Odontophore intrinsic, extrinsic muscles, and other structures similar to *K. corallina*, except for **m1v**, absent; **m1l**, narrow pair of jugal lateral protractor muscles, originating in lateral region of mouth, running towards posterior, inserting in lateral region of buccal mass posterior end; **m3**, similar, but slightly wider; **m7**, originated in same region in cartilages, but divided into 3 distinct and equidistant bundles; **m4-m5**, of difficult separation, being continuation from each other; **m10**, broader and more visible. **Radula.** (Fig. 19) with same characters as *K. corallina*, except in having more pairs per row (~350); and in having proportionally smaller and slightly more arched teeth. Esophagus slightly broader, mainly in its anterior region. Salivary glands narrow (Fig. 18A: sg). Stomach (Fig. 18C: st) bulbed, with anterior (dd) and posterior (dp) ducts

to digestive gland more elongated; anterior duct with only right branches. Middle portion of intestine, in its region after crossing aorta (aa) much wider, running obliquely, direct to right-posterior region of visceral mass (in).

Reproductive system. (Fig. 20) general structures similar to preceding species, remarks and distinctions following. Gonad (go) proportionally longer, ~1.5 whorl, not clearly divided in lobes (Fig. 17G), with well-developed digital acini. Hermaphroditic duct (Figs 20A, E: hd) gradually more intensely coiled towards anterior; except for short narrow region preceding its insertion (Fig. 20E). Seminal receptacle small (Figs 20E: sr), straight, long, cylindric, ~10 times longer than wide, tip rounded; inserted between two bulged terminal regions, being one of them insertion of hermaphroditic duct (Fig. 20E). Fertilization complex or carrefour (Fig. 20E: ca) elongated, triangular, tapering gradually up to becoming narrow duct inserted in end of albumen gland duct. Albumen gland (Fig. 20A: ag) ~twice larger than gonad. Albumen gland duct (Fig. 20E: ad) subterminal, connected to tip of spermoviduct as narrow, separated duct. Albumen chamber (Figs 20A, C: ac) as flattened, rather triangular blind sac, ~as long as wide, connected to beginning of spermoviduct by narrow region, jointed to duct of albumen gland. Secondary albumen chamber (as) ~with same size and form of primary chamber, insertion at some distance from albumen chamber insertion. Spermoviduct (Fig. 20A: eo) slightly narrower, slender, ~30 times longer than wide. Prostate wide (Fig. 20C: pt), almost 1/2 of spermoviduct diameter; uterus lacking glandular walls, highly, transversally, and relatively uniformly folded (Figs 20A–C: ut). Sperm inner longitudinal fold (Fig. 20C: sp) simple, tall, narrow fold, second small fold gradually appearing in basal third; both folds fusing with each other, originating vas deferens, slightly anterior to end of uterine level (Fig. 20B: vd). Genital muscle wide, attached to vaginal outer wall (Fig. 20A: gm). Bursa copulatrix (bc) and its duct (bd) as long as spermoviduct length (Fig. 20A); bursa duct with basal 3/5 with walls thick muscular (Fig. 20B). Basal region of duct of bursa copulatrix with smooth inner surface (Fig. 20B). Free oviduct and vagina with thick muscular walls (Fig. 20B: fo, vg). Penis slightly mostly straight, weakly coiled, ~85% of spermoviduct length if straightened (Fig. 20A: pe). Penis muscle inserted terminally in epiphallus tip (Figs 20A, D: pm), very short, simple. Penis walls weakly muscular, except for region adjacent to penis shield (Fig. 20D: mp), region with very thick muscular walls. Epiphallus (eh) ~1/5 of penis' length, amply opened to penis; only vas deferens insertion marking its limit (Fig. 20D: vd). Epiphallus inner surface only with ~8 narrow, small, parallel folds, being one of them larger, converging in vas deferens aperture (Fig. 20D: eh). Internal penial arrangement of folds clearly with 3 regions (Fig. 20D): (1) basal 1/5, highly muscular region (pm), lumen narrow, possessing only 4–5 low, uniform, longitudinal folds; (2) following basal 3/5, with strong pair of wide, rounded in section, longitudinal folds in a side, located close from each other, each one with simple margins in their basal 2/3, apical 1/3 possessing successive oblique branches in side turned to its counterpart, each main fold, after this, becoming oblique and successively branching; mosaic of low, longitudinal, regular folds flanking both strong folds; smooth space between both folds; **umbrella-like fold** (Fig. 20D: um) located distally to this region, tall and narrow, possessing 3 rods exceeding fold's edge, septum-like, inserted transversally in inner penis wall; (3) distal region, possessing only 5–6 separated longitudinal, uniform folds; some of them converging to aperture of vas deferens (Fig. 20D: vd), other continuous with epiphallus inner folds.

Central nervous system. (Fig. 18C: nr) Same characters as *K. corallina*.

Distribution. So far restricted to type locality.

Habitat. Under rocks, limestone areas.

Etymology. The specific epithet is in apposition, and refers to the native Tupi-Guarani godhead Tupã, the Thunder-God, creator of the earth, heaven, and seas. This is an allusion to the species being the largest of the genus.

Measurements. (in mm) MZSP 161200 (holotype, Figs 11A–F): 55.8 by 29.2; MZSP 151817 (paratype, Figs 11H–J): 47.0 by 24.4; MZSP 151823 (paratype, Figs 11K–M): 53.1 by 29.6.

Material examined. The types.

Taxonomic remarks. See Discussion ~~item~~.

***Kora ajar* new species** Figs 21–23

ZooBank: urn:lsid:zoobank.org:act:D72F5CEE-AF7B-4B6A-BE94-C8D48DD53DA2.

Types. Holotype MZSP 163700, 1 complete spm; paratypes: MZSP 151867, 7 spm, MZSP 151866, 39 shells, USNM, 2 shells, MNRJ, 2 shells, all from type locality. BRAZIL. **Minas Gerais**; Itacarambi, Serra de Itacarambi, 15°01'42"S 44°13'15"W, 740 m altitude, MZSP 151829, 7 spm (W. Vailant-Mattos col., i.2020).

Type locality. BRAZIL. **Minas Gerais**; Itacarambi, Vargem Grande, 466 m altitude, 15°00'50"S 44°04'34"W (W. Vailant-Mattos col., 7.ii.2020).

Diagnosis. Size about 55 mm, ~1.7 times longer than wide; lacking dorso-ventral compression. Apex with same color as remaining shell. Subsutural lighter band present. Delicate spiral striae present. Peristome with brown spots. Aperture occupying ~54% of length and ~71% width. Implantation of outer lip slightly horizontal. Inner lip with high middle fold. Umbilicus wide. Radula with narrow teeth, tip of cusps pointed, lacking expansion and subterminal furrow. Secondary columellar muscles with 5 insertions in left and 4 in right. Lacking m1v. Jaw rectangular. Odontophore cartilages ~75% fused. Pair m10 narrow, pair m2a present. Carrefour duct narrow and short, inserted in albumen chamber. Albumen chamber in curve. Penis ~90% of spermoviduct length, umbrella-like fold present, with 5 rods; epiphallus ~25% of penis length; penis muscle at epiphallus base.

Description (distinctive in anatomy). Shell. Length up to 50 mm, outline fusiform-globose, ~1.7x longer than wide. Color brown (Fig. 21), slightly lighter in tip, subsutural pale band in all whorls well-developed (Figs 21B, C, I, J, L). Protoconch (Figs 21G, 22A) with 2 whorls, bluntly pointed; length ~4% of shell length, and ~9.5% of shell width; mostly smooth, barely sculptured by axial riblets in last whorl. Limit between protoconch and teleoconch weakly visible, weakly prosocline. Teleoconch of ~4.2 whorls successively and uniformly increasing; whorls weakly concave; suture weakly deep; sculpture absent, except for growth lines and delicate axial, uniform undulations, ~60 in penultimate whorl; weak spiral striae gradually appearing in last whorls, more visible in last whorl (Figs 21B, C, F, I, L), distribution relatively uniform from suture up to inferior region of last whorl. Transverse section rounded (Fig. 21E). Peristome weakly dislocated to right; deflected, except for region of callus. Callus weak (Figs 21A, D, H, J, K, L). Aperture wide, weakly dislocated from spire longitudinal axis; length ~54% of shell length, ~71% of shell width. Outer lip inserted distantly from adjacent suture, simple, arched. Inner lip concave, superior half weakly convex, mostly showing outer surface of last whorl; inferior half weakly convex, concave only inferiorly; bearing oblique, broad fold in limit with superior half, having weak elevation preceding its end in inner lip (Figs 21 D, J, L); tooth

length ~30% of peristome length. Umbilicus opened, relatively wide, partially covered by inferior half of inner lip (Fig. 21F).

Head-foot. (Fig. 23A) with similar features as *K. corallina*. Except for both secondary columellar muscles slightly narrower, and with fewer basal insertions, 4 in right (cr), 5 in left (cl); additionally pair of central-medial longer insertions very asymmetric, left one almost as small as remaining basal branches; right one very big, making right secondary columellar muscle (cr) almost bifid in its insertion. Also, insertion of secondary columellar muscles more medially positioned.

Mantle organs. (Figs 22C, F) most features similar to *K. corallina*, distinctions and remarks following. Mantle border (mb) with large secondary fold (Fig. 22F: superior mf) at left from pneumostome (pn) with tall pointed left end. Pallial edge gland in left-dorsal region of mantle border (Fig. 22F: gp), white, claviform. Pulmonary venation strongly developed, especially in region preceding pneumostome up to almost half whorl posterior to it, with vessels touching each other. Pulmonary vein (cv) entirely broad; its anterior end bifid close to pneumostome (pn); those at right from it basically straight, more developed anteriorly and in region adjacent to kidney; those at left from it longer and more complex, obliquely crowding in region preceding pneumostome, with anastomosis with next main longitudinal secondary vessel at left from it (Fig. 22F). Region at left from pulmonary vein (cv) with single pair of long, longitudinal, intercalated vessels (bv).

Visceral mass. (Fig. 23F) with same characters as *K. corallina*. Except for stomach positioned more posteriorly, and by presence of pallial pre-rectal muscle (ml), originating in columellar region of shell in level of pericardium, running slightly flattened, fan-like, towards ventral, through digestive gland, very close to anterior aorta and adjacent portion of intestine, inserting splaying in pallial floor posterior end.

Circulatory and excretory systems. (Figs 22C, F, 23F) general characteristics as those described for *K. corallina*, except for pericardium (pc) with 2/3 its width; and for arrangement of kidney lobe, as 3 main narrow, tall folds (Figs 22C: kl, 23F: ki).

Digestive system. (Fig. 23F) overall morphology similar to that of *K. corallina*. Differences and remarks following. Peribuccal muscles and oral tube relatively narrow. Jaw plate (Figs 22B, 23F: jw) thick, rather rectangular, cutting edge straight; sculptured by successive, uniform, transverse, narrow folds. Odontophore intrinsic, extrinsic muscles, and other structures similar to *K. corallina*, except for **m1v**, **m1l,** both absent; **m2a**, narrow, working as pair of ventral protractors of buccal mass, originating in lateral region of mouth, running towards anterior, inserting in edge of m2, in its both lateral insertions (replacing m1v); **m3**, similar, but as single wider, weak bundle covering dorsal end of membrane surrounding radular sac (mr); **m7**, with single, fan-like origin; **m10**, pair narrow. **Radula** (Fig. 24) most attributes similar to those of *K. corallina*, except in having teeth much slenderer, from base to cusp; tip of cusp sharp pointed, lacking expansion and subterminal furrow; marginal teeth slightly more arched (Fig. 24C). Esophagus (es) slightly broader, mainly in its anterior region. Salivary glands (sg) with shorter ducts (sd). Stomach (st) bulbed, with anterior (dd) and posterior (dp) ducts to digestive gland narrower and more elongated; anterior duct with only right branches, each one very long.

Reproductive system. (Figs 23B–E) general structures similar to preceding species, remarks and distinctions following. Gonad (Fig. 23C: go) proportionally longer, ~1.4 whorl, not clearly divided in lobes, with well-developed, aligned digital acini. Hermaphroditic duct (hd) coiled only in its middle region; narrow and straight in both ends (Fig. 23C), always with tight curve

preceding its insertion in carrefour (Fig. 23E: bd). Seminal receptacle elongated (Figs 23C, E: sr), straight, weakly flattened, ~15 times longer than wide, tip rounded, base slightly broader by side insertion of hermaphroditic duct (Fig. 23E). Fertilization complex or carrefour (Fig. 23E: ca) elongated, narrowly triangular, tapering gradually up to narrow duct inserted in albumen gland chamber-spermoviduct limit. Albumen gland (Fig. 23C: ag) ~twice larger than gonad. Albumen gland duct (Fig. 23E: ad) subterminal, narrow, connected to tip of albumen chamber. Albumen chamber (Figs 23C, E: ac) flattened, curved blind sac, ~twice long than wide, connected to beginning of spermoviduct by narrow region. Secondary albumen chamber (as) ~1.5 time larger than primary chamber, slightly triangular, insertion at some distance from albumen chamber insertion. Spermoviduct (Fig. 23C: eo) slightly narrower, slender, ~30 times longer than wide. Prostate wide (Fig. 23C, D: pt), ~1/2 of spermoviduct diameter; uterus lacking glandular walls, highly, transversally, and relatively uniformly folded (Figs 20C–E: ut). Sperm inner longitudinal fold (Fig. 23D: sp). Anterior end of spermoviduct, entire free oviduct, vagina and ¾ of duct of bursa copulatrix with walls very thick muscular; their inner lumen tightly narrow, with thin longitudinal folds. Bursa copulatrix (bc) and its duct (bd) slightly shorter than spermoviduct length (Fig. 23C); bursa duct with basal 3/4 with walls thick muscular. Penis slightly mostly straight, weakly coiled, ~90% of spermoviduct length if straightened (Fig. 23C: pe). Penis muscle inserted subterminally in epiphallus, with fibers inserted since its base, in region of vas deferens insertion, running attached to epiphallus side (Figs 23B, C: pm). Penis walls weakly muscular, except for basal ¼, region adjacent to penis shield (Fig. 23C: mp), region with very thick muscular walls twice longer than penis shield. Epiphallus (eh) ~1/4 of penis' length, amply opened to penis; only vas deferens insertion marking its limit (Fig. 23B: vd). Epiphallus inner surface only with ~8 narrow, small, parallel folds, being one of them larger, converging in vas deferens aperture. Internal penial arrangement of folds clearly with 3 regions (Fig. 23B): (1) basal 1/4, highly muscular region (pm), lumen narrow, possessing only 4–5 low, uniform, longitudinal folds; (2) following basal 1/2, with strong pair of narrow, rounded in section, longitudinal folds in a side, located close from each other, each one with simple margins in their basal 2/3, apical 1/3 composed of successive small nodes, 6–7 in number, positioned turned to each other; mosaic of 4 low, longitudinal, regular folds flanking both strong folds; smooth space between both folds; distal third of area between both main folds, secondary folds abruptly disappearing, with only irregular surface produced by nodes of main folds; **umbrella-like fold** (Fig. 23B: um) located distally to this region, between middle and distal peins thirds, low and narrow, possessing 5–6 small rods exceeding fold's edge, septum-like, inserted transversally in inner penis wall; (3) distal ¼ region, possessing only 7–8 separated, longitudinal, uniform folds; some of them converging to aperture of vas deferens (Fig. 23B: vd), other continuous with epiphallus inner folds.

Spermatophore. (Figs 22D–E) found inside duct of bursa copulatrix. Virtually similar to that described for *K. nigra*, except for stem slightly shorter; and distal, bulged portion having blunt (instead in having sharp) tip.

Central nervous system. Same characters as *K. corallina*.

Distribution. So far restricted to type locality.

Habitat. Cerrado biome, altitude of 466 to 740 m.

Etymology. The specific epithet is from the Latin [illegible]rks *ajar*, also applied in English, correspondent to the umbilicus aperture.

Measurements. (in mm). holotype MZSP 163700 (Figs 13A–G): 47.4 by 28.8; MZSP 151866#1 (Figs 13H–J): 43.0 by 26.4; #2 (Figs 13K–L): 49.1 by 27.9.

Material examined. The types.

Taxonomic remarks. See [illegible]cussion item.

***Kora aetheria* new species** Figs 25–27

ZooBank: urn:lsid:zoobank.org:act:D19B73B4-F62D-4753-8595-54E92445E5E2.

Types. Holotype MZSP 163400, 1 complete spm; paratypes: MZSP 153856, 4 spm, from type locality. BRAZIL. **Bahia**; Serra do Ramalho, Pedreira, 13°26'40"S 43°49'05"W, MZSP 152249, 23 shells, USNM, 1 shell, MNRJ, 1 shell (W. Vailant-Mattos col., v.2019).

Type locality. BRAZIL. **Bahia**; Serra do Ramalho, Toca, 13°38'13"S 43°50'10"W (W. Vailant-Mattos col., v.2019).

Diagnosis. Size about 30 mm, ~2 times longer than wide; dorso-ventrally weakly compressed. Apex with same color as remaining shell. Subsutural lighter band absent. Peristome white. Delicate spiral striae absent. Aperture occupying ~45% of length and ~60% width. Implantation of outer lip slightly vertical. Inner lip lacking high middle fold. Umbilicus narrow. Secondary columellar muscles with 5 insertions in left and 7 in right. One pair of m1v. Jaw with central notch. Odontophore cartilages ~75% fused. Pair m10 broad. Carrefour duct narrow and long, inserted between duct of albumen gland and spermoviduct. Albumen chamber sac-like. Penis ~65% of spermoviduct length, umbrella-like fold present, with 6 rods; epiphallus ~33% of penis length; penis muscle at epiphallus tip.

Description (distinctive in anatomy). Shell. (Figs 25, 26B[illegible]ngth up to 33 mm, outline fusiform-elongated, ~1.9–2.0x longer than wide. Color unifo[illegible]beige (Figs 25A–I) to light brown (Figs 25J–L) with paler apex, gradually becoming dark brown in last whorl (Fig. 25K); subsutural pale band in all whorls well-developed in darker specimens (Figs 25 J–L). Protoconch (Figs 25G, 26B) with 2.2 whorls, bluntly pointed; length ~4% of shell length, and ~14% of shell width (Fig. 25E); almost entirely sculptured by uniform, axial riblets. Limit between protoconch and teleoconch weakly visible, weakly prosocline. Teleoconch of ~4.5 whorls successively and uniformly increasing; whorls weakly concave; suture weakly deep; sculpture absent, except for growth lines and delicate axial, uniform undulations, ~50 in penultimate whorl. Dorso-ventrally very softly flattened (Fig. 25E). Peristome very weakly dislocated to right; deflected, except for region of callus. Callus weak (Figs 25J, L) to relatively thick (Figs 25A, C, D, H). Aperture wide; length ~44–47% of shell length, ~60% of shell width. Outer lip inserted distantly from adjacent suture, simple, arched. Inner lip concave, superior half weakly convex, mostly showing callus or outer surface of last whorl; inferior half straight or weakly convex, concave only inferiorly; bearing low, oblique, uniform fold in limit with superior half (Figs 25C, D, L); tooth length ~35% of peristome length. Umbilicus opened, narrow, partially covered by inferior half of inner lip (Fig. 25F).

Head-foot. (Fig. 27A) with similar features as *K. corallina*. Except for details of both secondary columellar muscles, with fewer basal insertions, 7 in right (cr), 5 in left (cl); additionally, pair of central-medial longer insertions slightly symmetrical sized and similar to remaining basal insertions. Pedal gland (pg) wider and shorter.

Mantle organs. (Figs 26A, C) most features similar to *K. corallina*, distinctions and remarks following. Mantle border (mb) with large secondary fold (mf) at left from pneumostome (pn) with rounded, projected left end. Pulmonary venation strongly developed, especially in region preceding pneumostome, vessels not touching each other, showing secondary transverse venation in interspaces. Pulmonary vein (cv) relatively narrow; its anterior end with ramifications, bifid at end; those at right from it basically straight, more developed anteriorly; those at left from it longer and more complex, with anastomosis with next main longitudinal secondary vessel at left from it (Fig. 26A: bv). Region at left from pulmonary vein (cv) with single pair of long, longitudinal, intercalated vessels; right vessel Y-shaped, with strong bifurcation anterior, covering collar vessel (Fig. 26A: bv).

Visceral mass. (Fig. 27B) with similar characters as *K. corallina*.

Circulatory and excretory systems. (Fig. 26C) general characteristics as those described for *K. corallina*. Arrangement of kidney lobe (kl), as 4 successive larger, tall folds, with ventral lobe well-developed.

Digestive system. (Fig. 27B) overall morphology similar to that of *K. corallina*. Differences and remarks following. Peribuccal circular muscles (mj) longer and thicker. Jaw plate (Fig. 26D) thin, curved, cutting edge only concave, notched at middle; sculptured by successive, rather uniform, transverse, narrow folds. Buccal cavity also similar, except for pair of salivary ducts having subterminal bulging. Odontophore intrinsic, extrinsic muscles, and other structures similar to *K. corallina*, **m1l**, present; **m2**, slightly narrower, inserted in buccal mass in 3 separated, isometric bundles; **m3**, similar, but slightly wider; **m10**, broader and more visible. **Radula** (Figs 26E–G) with similar characteristics as *K. corallina*, except for teeth slightly more arched, mainly marginal teeth (Fig. 26G). Esophagus (es) narrow, walls thicker, with internal 7–8 longitudinal, separated folds. Salivary glands narrow (sg). Stomach (st) narrow, as simple curve, with anterior (dd) and posterior (dp) ducts to digestive gland slender; anterior duct strongly bifurcated.

Reproductive system. (Fig. 27: C–F) general structures similar to preceding species, remarks and distinctions following. Hermaphroditic duct (Figs 27C, E: hd) entirely, uniformly and delicately coiled. Seminal receptacle small (sr), very elongated, weakly flattened, ~15 times longer than wide, tip rounded; inserted by side of insertion of hermaphroditic duct (Fig. 27C). Fertilization complex or carrefour (Fig. 27E: ca) elongated, initially (1/4) bulged, abruptly tapering, becoming narrow, long duct, equivalent to ¾ its length; inserting at tip of spermoviduct. Albumen gland (Fig. 27C: ag) ~4x larger than gonad. Albumen gland duct (Fig. 27E: ad) subterminal, very narrow, connected to tip of spermoviduct in same region of carrefour duct. Albumen chamber (Figs 27C, E: ac) bulged blind sac, ~twice longer than wide, widely connected to beginning of spermoviduct. Secondary albumen chamber (as) ~double of primary chamber, balloon-like, duct narrow, insertion at some distance from albumen chamber insertion. Spermoviduct (Fig. 27C: eo) slightly shorter, ~10 times longer than wide. Prostate wide but low (Fig. 27F: pt), ~30% of spermoviduct diameter; uterus lacking glandular walls, highly, transversally, and relatively uniformly folded (Figs 27C, F: ut). Sperm inner longitudinal fold (Fig. 27F: sp) simple, tall, narrow fold. Anterior end of spermoviduct, entire free oviduct, vagina and ¾ of duct of bursa copulatrix with walls very thick muscular; their inner lumen tightly narrow, with thin longitudinal folds. Genital muscle very narrow, attached to vaginal outer wall (Fig. 26C: gm). Bursa copulatrix (bc) and its duct (bd) ~80% of spermoviduct length (Fig. 27C); bursa duct with basal 1/3 with walls thick muscular. Basal region of duct of bursa copulatrix with 4–5 longitudinal, tall folds. Free oviduct and vagina with

thick muscular walls; inner lumen of both narrow, bearing only 4–5 longitudinal, tall folds. Penis slightly straight, ~65% of spermoviduct length (Fig. 27C: pe). Penis muscle inserted terminally in epiphallus tip (Figs 27C, D: pm), very short, simple. Penis walls weakly muscular, except for region adjacent to penis shield (Fig. 27D: mp), region with very thick muscular walls equivalent to ~1/3 of its length. Epiphallus (eh) ~1/3 of penis' length, amply opened to penis; only vas deferens insertion marking its limit (Fig. 27D: vd). Epiphallus inner surface only with ~8–10 narrow, low, parallel folds, being one of them slightly larger (Fig. 27D: eh). Internal penial arrangement of folds clearly with 3 regions (Fig. 27D): (1) basal ~1/3, highly muscular region (pm), lumen very narrow, possessing only 4–5 low, uniform, longitudinal folds; (2) following basal ~1/3, with strong pair of wide, rounded in section, longitudinal-slightly curved folds in a side, located close from each other, each one with simple margins in their basal half; apical half possessing 6–7 successive, uniform, oblique branches in side turned to its counterpart, each secondary branch almost touching its pair of other fold; smooth space between both folds in both sides, including wide distance between them and umbrella-like fold; **umbrella-like fold** (Fig. 27D: um) located distally to this region, low and narrow, possessing 6 rods exceeding fold's edge, septum-like, inserted transversally in inner penis wall; (3) distal region, possessing only 56 separated longitudinal, uniform folds; some of them converging to aperture of vas deferens (Fig. 27D: vd), other continuous with epiphallus inner folds.

Central nervous system. (Fig. 27B: nr) Same characters as *K. corallina*.

Distribution. So far restricted to Serra do Ramalho region, Bahia.

Habitat. Under rocks, limestone areas.

Etymology. The specific epithet is a Latin word meaning ethereal, heavenly, divine, [illegible]estial, an allusion to the simplicity of the shell shape.

Measurements. (in mm) MZSP 163400 (holotype, Figs 25A–G): 29.4 by 15.3; MZSP 153856 (Figs 25H–I): 30.3 by 15.1; MZSP 152249 (Figs 25J–L): 32.6 by 16.6.

Material examined. The types.

Taxonomic remarks. See Discussion item.

***Kora jimenezi* new species** Figs 28–33

ZooBank: urn:lsid:zoobank.org:act:105DB150-42A9-4569-952A-2C130DB634FB.

Types. Holotype MZSP 151907; paratypes: MZSP 151906, 58 shells, MNRJ, 2 shells, USNM, 2 shells, all from type locality. BRAZIL. **Minas Gerais**; Itacarambi (W. Vailant-Mattos col.), 15°10'30"S 44°13'24"W, altitude, 530 m, MZSP 152191, 28 shells (ii.2019), border of National Park of Peruaçu, 15°06'35"S 44°10'13"W, MZSP 152190, 18 shells (i.2020); Between Januária and Barreiros, 15°28'47"S 44°22'03"W, MZSP 152162, 4 shells, 152139, 2 spm (iv.2020).

Type locality. BRAZIL. **Minas Gerais**; Itacarambi, west downtown, 15°00'38"S 44°07'10"W, altitude 540–550 m (W. Vailant-Mattos col., ii.2019).

Diagnosis. Size about 45 mm, ~2 times longer than wide; lacking dorso-ventral compression. Apex with same color as remaining shell. Subsutural lighter band present. Delicate spiral striae present. Peristome white. Delicate spiral striae present. Aperture

occupying ~45% of length and ~70% width. Implantation of outer lip slightly horizontal. Inner lip with high middle fold. Umbilicus narrow. Kidney elongated, narrow. Secondary columellar muscles with 3 insertions in left and in right. Lacking pairs m1v. Jaw arched. Odontophore cartilages ~75% fused. Pair m10 filiform, pair m2a present. Carrefour duct narrow and short, inserted in duct of albumen chamber; bulged region present. Albumen chamber in curve. Penis ~100% of spermoviduct length, lacking umbrella-like fold; epiphallus ~25% of penis length; penis muscle at epiphallus tip.

Description (distinctive in anatomy). Shell. Length up to 44 mm, outline fusiform-elongated, 1.9–2.2x longer than wide. Color brown, lighter in spire, gradually becoming dark brown in last whorl (Figs 28B, F, I, K, 28B, F); subsutural pale band in all whorls well-developed (Figs 28D, F, 29B, F, G). Protoconch (Figs 28G, 29D, J, I) with 2 whorls, bluntly pointed; length ~10% of shell length, and ~13% of shell width (Fig. 28E); mostly smooth, barely sculptured by axial riblets in last whorl. Limit between protoconch and teleoconch weakly visible, weakly prosocline. Teleoconch of ~4.2 whorls successively and uniformly increasing; whorls weakly concave; suture weakly deep. Sculpture growth lines and delicate axial, uniform undulations, ~60 in penultimate whorl; also weak, uniform spiral striae, clearer in last whorl, 55–60 in last whorl, each stria composed of minute aligned pits, separated from each other by equivalent distance of their width. Transverse section rounded (Fig. 28E). Peristome weakly dislocated to right; deflected, except for region of callus. Callus weak (Figs 29A, H, J, 30A, E, G, H). Aperture wide, somewhat dislocated from spire longitudinal axis; length ~45% of shell length, ~70% of shell width. Outer lip inserted distantly from adjacent suture, simple, arched. Inner lip concave, superior half weakly convex, mostly showing outer surface of last whorl; inferior half weakly convex, concave only inferiorly; bearing oblique fold in limit with superior half, having weak elevation preceding its end in inner lip (Figs 28D, 29C); tooth length ~31% of peristome length. Umbilicus opened, relatively wide, partially covered by inferior half of inner lip (Fig. 28F).

Head-foot. (Fig. 30A) with similar features as *K. corallina*. Except for details of both secondary columellar muscles, with fewer and broader basal insertions, 3 in each side (cr, cl); additionally, pair of central-medial longer insertions slightly as medial part of remaining basal insertions. Pedal gland (pg) much shorter.

Mantle organs. (Figs 31A–D) most features similar to *K. corallina*, distinctions and remarks following. Mantle border (mb) with large secondary fold (mf) at left from pneumostome (pn) with rounded, projected left end (Figs 31A, B). Pulmonary venation not so developed, composed mainly of pulmonary vein (cv) and perpendicular set of narrow secondary vessels in both sides (Figs 31A, C) especially in region preceding pneumostome. Pulmonary vein (cv) relatively wide; its anterior end lacking ramifications, ending in edge of pneumostome (Fig. 31C: cv). Region at left from pulmonary vein (cv) lacking visible large vessels (Fig. 31A). Mante with well-developed longitudinal muscular micro-fibers (Fig. 31D: mi).

Visceral mass. (Figs 30B, 31F) with similar characters as *K. corallina*. Gonad (go) located more anterior, ~2.5 whorls anterior to posterior end.

Circulatory and excretory systems. (Figs 31A, G, H) general characteristics as those described for *K. corallina*; except for kidney shape, very antero-posteriorly elongated (ki); kidney ~8-times longer than wide; nephropore (ne) in anterior end, at beginning of primary ureter (up). Arrangement of kidney lobe (Figs 30B, 31H: kl), as 6-7 tall folds, fulfilling internal space almost completely.

Digestive system. (Figs 30B–D) overall morphology similar to that of *K. corallina*. Differences and remarks following. Jaw plate (Fig. 31E) thin, narrow, arched; sculptured by successive, rather uniform, transverse, broad folds. Buccal cavity also similar, except for pair of dorsal folds relatively narrow, producing wide dorsal chamber (dc); salivary ducts aperture in tip of papilla (sa), located in medial-posterior side of dorsal folds medial edge. Odontophore intrinsic, extrinsic muscles, and other structures similar to those of *K. corallina*, except for: **m1l**, present, very narrow (Fig. 30C); **m2**, very thick, with single bundle; **m2a**, present, and broad, possessing small nerve inside (Fig. 30C); **m3**, not visible; **m5**, part originated in m4; **m7**, thick, as single bundle (Fig. 30D); **m8** and **m10**, both small. **Radula** (Figs 32B–D) with same features of *K corallina*, except in having slightly more erected cusp, keeping base more exposed. Esophagus (Figs 30, 32A: es) initially narrow, becoming broad, being like that up to gastric insertion; internally well-developed longitudinal folds (Fig. 32A: ef). Salivary (sg) glands as 2 separated, narrow masses. Stomach (Fig. 30B: st) narrow, as simple curve, with anterior (dd) and posterior (dp) ducts to digestive gland relatively broad; anterior duct strongly bifurcated at base, having branches only in its right side.

Reproductive system. (Fig. 31) general structures similar to preceding species, remarks and distinctions following. Gonad as 4–5 well-separated, rounded masses (Figs 30B, 31F: go), united by narrow branches of gonoducts. Hermaphroditic duct (hd), initially narrow and uncoiled (Figs 30B, 31F), gradually becoming intensely coiled, with thick and irregular coils (Figs 33B, E). Seminal receptacle small (Figs 33B, E: sr), elongated, weakly flattened, ~6 times longer than wide, tip performing 1 whorl (Fig. 33E); inserted between curved insertion of hermaphroditic duct and wide projection of carrefour (ca), about as wide as receptacle, ~1/3 its length. Fertilization complex or carrefour (Fig. 33E: ca) (except posterior projection) conic, abruptly tapering, becoming narrow, short duct; inserting at tip of spermoviduct, in intersection of albumen gland duct (ad) and its chamber (ac). Albumen gland (ag) of ~1/3 whorl in length. Albumen gland duct (Fig. 33E: ad) subterminal, narrow, connected to tip of spermoviduct by side of carrefour duct. Albumen chamber (Figs 33B, E: ac) bulged curve, ~3-times longer than wide, widely connected with its proximal end continuous with spermoviduct (eo). Secondary albumen chamber (as) slightly larger than of primary chamber, balloon-like, duct broad, insertion at some distance from albumen chamber insertion. Spermoviduct (Fig. 33B: eo) long and narrow, ~25 times longer than wide. Prostate wide but low (Fig. 33A: pt), almost 1/2 of spermoviduct diameter; uterus lacking glandular walls, highly, transversally, and relatively uniformly folded (Figs 33A–C: ut). Sperm inner longitudinal fold (Figs 33A, C: sp) simple, tall, narrow fold, with distal edge sharp. Anterior regions of genital tubes with walls weakly muscular (Fig. 33C); free oviduct (fo) and vagina (va) with inner surface smooth. Bursa copulatrix (bc) and its duct (bd) ~80% of spermoviduct length (Fig. 33B); bursa duct with basal half slightly thicker than distal half. Basal region of duct of bursa copulatrix with 4–5 longitudinal, long, rather irregular folds (Fig. 33C: bd). Genital muscle (Fig. 33B: gm) small, narrow. Penis relatively broad, ~as long as spermoviduct length (Fig. 33B: pe). Penis muscle inserted terminally in epiphallus tip (Figs 33B, D: pm), short, simple. Penis walls entirely weakly muscular (Fig. 33D). Epiphallus (eh) ~1/4 of penis' length, amply opened to penis; only vas deferens insertion and end of special inner fold (pf) marking its limit (Fig. 33D: vd). Epiphallus inner surface only with ~4 narrow, low, parallel folds (Fig. 33D: eh). Internal penial arrangement of folds clearly with 3 regions (Fig. 33D): (1) basal ~2/3, after short basal region having only longitudinal wrinkles, pair of strong folds abruptly appearing (pf), with rounded end, running close from each other, interspace smooth, equivalent to their width, all along this region, narrowing gradually; surrounding both folds smooth surface; (2) following ~1/6 as

bulged region, preceded by sphincter-like constriction, internally having only 7–8 longitudinal, uniform, separated, narrow folds; (3) distal ~1/3 as narrow region, internally having only single, tall fold (pf), fold basal half only planar, gradually increasing, becoming Y-shaped in section, its distal end tapering and converging to aperture of vas deferens (vd).

Central nervous system. (Fig. 30B: nr) Same characters as *K. corallina*.

Distribution. So far restricted to Itacarambi region, Minas Gerais.

Habitat. Under rocks or rock crevices.

Etymology. The specific epithet is in honor of ~~the~~ David Jimenez, an expedition sponsor and remarkable contributor to the malacological [illegible]a.

Measurements. (in mm): MZSP 151907 (holotype, Figs 28A–G): 43.8 by 24.2; MZSP 151906#1 (Figs 28H–I): 41.3 by 20.1; #3 (Figs 29A–D): 38.6 by 18.7; #4 (Figs 29E–F): 41.4 by 19.6; paratype MZSP 151906#5 (Fig. 29G): 42.5 by 18.9.

Material examined. The types.

Taxonomic remarks. See Discussion item.

***Kora uhlei* new species** Figs 34–38

ZooBank: urn:lsid:zoobank.org:act:B8283016-B72B-41A6-A1B3-A9CADB1D757F.

Types. Holotype MZSP 165720, spm; paratypes: MZSP 164889, 7 spm, MZSP 164888, 15 shells, all from type locality.

Type locality. BRAZIL. **Minas Gerais**; Matias Cardoso, near Lagoa do Cajueiro State Park, near São Francisco River, 14°53’10”S 43°54’51”W (Wesley Vailant-Mattos col., vi.2023).

Diagnosis. Size about 45 mm, ~2 times longer than wide; lacking dorso-ventral compression. Apex with same color as remaining shell. Subsutural lighter band present. Peristome with brown spots. Delicate spiral striae absent. Aperture occupying ~50% of length and ~65% width. Implantation of outer lip slightly vertical. Inner lip with high middle fold. Umbilicus wide. Secondary columellar muscles with 3 insertions in left and in right. Absence of pairs of m1v. Jaw rectangular. Odontophore cartilages ~90% fused. Pair m10 narrow, pair m7 filiform. Carrefour duct narrow and long, inserted between duct of albumen gland and albumen chamber; bulged region present. Albumen chamber in curve. Penis ~60% of spermoviduct length, umbrella-like fold present, with 3 rods; epiphallus ~25% of penis length; penis muscle at epiphallus base.

Description (distinctive in anatomy). Shell. (Fig. 34) length up to 42 mm, outline fusiform-globose, ~2x longer than wide. Color brown, lighter in spire, gradually becoming dark brown in last whorl (Figs 34B, K); subsutural pale band developed (Figs 34B, D, J, K). Protoconch (Figs 34F, I) with 2 whorls, bluntly pointed; length ~5.5% of shell length, ~17% of shell width (Figs 34G, H); mostly smooth, barely sculptured by axial riblets in last half whorl. Limit between protoconch and teleoconch visible, weakly prosocline. Teleoconch of ~4.2 whorls successively and uniformly increasing; whorls weakly concave, profile almost straight; suture shallow. Sculpture growth lines and delicate axial, uniform undulations, ~60 in penultimate whorl; spiral striae absent. Transverse section rounded (Figs 34G, H). Peristome not dislocated; deflected,

except for region of callus. Callus weak (Figs 34A, C, E, J, L). Aperture wide, not dislocated from spire longitudinal axis; length ~50% of shell length, ~65% of shell width. Outer lip inserted distantly from adjacent suture, simple, arched. Inner lip concave, superior half weakly convex, mostly showing outer surface of last whorl covered by thin callus; inferior half almost straight, concave only inferiorly; bearing oblique fold in limit with superior half (Figs 34C, E, L); tooth length ~35% of peristome length. Umbilicus opened, relatively wide, partially covered by inferior half of inner lip (Figs 34D, M, N).

Head-foot. (Fig. 35A) with similar features as *K. corallina*. Except for details of both secondary columellar muscles, with fewer and broader basal insertions, 3 in left side (cl), and single, wide bundle in right side (cr), having a single additional narrow anterior branch adjacent to genital (gm) and ommatophore (me) muscles; additionally, pair of central-medial longer insertions well-developed, but turned backwards (instead of anteriorly).

Mantle organs. (Figs 36A, B) most features similar to *K. corallina*, distinctions and remarks following. Mantle border (mb) with large secondary fold (mf) at left from pneumostome (pn) with blunt, projected left end (Fig. 36A: mf). Mantle edge gland well-developed, white (gp). Pulmonary venation strong in region of pneumostome, very branched in anterior end of pulmonary vein (cv); those at left of pulmonary vein intercalated with its branches and branches from collar vessel. Vessels at right from pulmonary vein, perpendicular positioned, well-developed all along pulmonary length, also intercalated and slightly more developed in region preceding pneumostome. Pulmonary vein (cv) relatively narrow. Region at left from pulmonary vein (cv) with visible large, intercalated vessels (Fig. 36A). Mante with well-developed longitudinal muscular micro-fibers (Fig. 36B: mi).

Visceral mass. (Figs 35B, 36D) with similar characters as *K. corallina*. Gonad (go) located more ~1 whorl anterior to posterior end.

Circulatory and excretory systems. (Figs 36F, G) general characteristics as those described for *K. corallina*; except for kidney shape, relatively antero-posteriorly elongated (ki); kidney ~3-times longer than wide, triangular. Reno-pericardial area occupying ~1/15 of pulmonary roof. Arrangement of kidney lobe (Fig. 36G: kl), as 6–7 tall folds, fulfilling internal space almost completely.

Digestive system. (Figs 35B–D) overall morphology similar to that of *K. corallina*. Differences and remarks following. Jaw plate (Fig. 36C) thin, wide (~2.5 times wider than long), almost straight; sculptured by successive, rather uniform, transverse, narrow folds; cutting edge chevron-like. Buccal cavity also similar, except for pair of dorsal folds (Fig. 35C: df) relatively narrow, producing wide dorsal chamber between them; salivary ducts aperture in tip of papilla (sa), located in medial-middle side of dorsal folds medial edge. Odontophore intrinsic, extrinsic muscles, and other structures similar to those of *K. corallina*, differences and notes following (Figs 35B, D, 36E) **m1l**, present, narrow and long; **m2**, narrow, inserted as 2 bundles; **m3**, longitudinal pair, in esophageal insertion area; **m5**, mostly by side of m4, originated from cartilages; **m7**, extremely narrow, each one originated in 2 different points (Fig. 35D); **m8**, broad. Pair of cartilages fused with each other in higher degree, ~90%. **Radula** (Figs 37) with same attributes as *K corallina*, except in having more pairs of teeth per row (~300) (Fig. 37A); cusp more elongated, touching adjacent row (Figs. 37B, C), with tip slightly more arched; 7–8 more marginal teeth with great diminishment of size (Fig. 37D). Salivary (sg) glands as 2 rounded masses, partially united in medial region (Fig. 35B: sg). Stomach (Fig. 35B, 36D: st) bulged; internally with 4–5 transverse folds, with anterior (dd) and posterior (dp)

ducts to digestive gland relatively broad; anterior duct large bifurcation at base, having branches only in its right side. Posterior duct relatively short, also having only branches in right side. Intestine (in) with pair of low parallel folds initiating in stomach, ending in region adjacent to pericardium (Fig. 35B).

Reproductive system. (Figs 35F, 38) general structures similar to preceding species, remarks and distinctions following. Gonad as single block of digitiform acini (Figs 35F, 36D: go). Hermaphroditic duct (hd), initially narrow and uncoiled (Fig. 36D), abruptly becoming intensely coiled, with very thick, irregular coils (Figs 35F, 36D: hd). Seminal receptacle small (Figs 35F, 38A: sr), elongated, weakly flattened, ~6 times longer than wide, tip bluntly pointed (Fig. 38A); inserted between curved insertion of hermaphroditic duct and narrow projection of carrefour (ca), about as wide as receptacle, ~1/3 its length. Fertilization complex or carrefour (Fig. 38A: ca) (except posterior projection) conic, tapering, becoming narrow, long duct, as long as carrefour itself; inserting in intersection of albumen gland duct (ad) and its chamber (ac). Albumen gland (ag) of ~1/3 whorl in length. Albumen gland duct (Fig. 38A: ad) subterminal, narrow, connected to superior side of albumen chamber (ac), by side of carrefour duct. Albumen chamber (Figs 35F, 38A: ac) as small blind-sac, ~twice longer than wide, connected to tip of spermoviduct by narrow, short duct located also in its posterior region, opposite to insertion of carrefour. Secondary albumen chamber (as) ~3x larger than of primary chamber, bulged, balloon-like, duct narrow, insertion at some distance from albumen chamber insertion. Spermoviduct (Fig. 35F: eo) long and narrow, ~20 times longer than wide. Prostate wide, tick (Fig. 35E: pt), almost 1/2 of spermoviduct diameter and thickness; uterus lacking glandular walls, highly, transversally, and relatively uniformly folded (Figs 35F, 38B: ut). Sperm inner longitudinal fold (Figs 35E, 38B: sp) simple, posteriorly short, gradually becoming taller, broad, fulfilled by prostate gland in anterior region (Fig. 38B). Anterior regions of genital tubes with muscular walls (Fig. 35B); free oviduct (fo) and vagina (va) with inner surface possessing longitudinal, wide, low folds. Bursa copulatrix (bc) and its duct (bd) ~80% of spermoviduct length (Fig. 35F); bursa duct with basal third with walls thick muscular (Fig. 38B: bu). Basal region of duct of bursa copulatrix with 4–5 longitudinal, long, wide folds (Fig. 38B: bd). Genital muscle (Figs 35F, 38B: gm) large, single. Penis relatively broad, ~60% of spermoviduct length (Fig. 35F: pe). Penis shield (ps) short, ~10% of penis length. Penis muscle inserted subterminally, along epiphallus lateral side (Figs 35F, 38C: pm), long, with epiphallus zigzagging it. Penis walls entirely weakly muscular, except for its base, with ~1/5 thick muscular (Fig. 38C: mp). Epiphallus (eh) ~1/4 of penis' length if straightened, amply opened to penis; only vas deferens insertion marking its limit (Fig. 38C: vd). Epiphallus inner surface only with ~8–10 narrow, low, parallel folds (Fig. 38C: eh). Internal penial arrangement of folds clearly with 3 regions (Fig. 38C): (1) basal ~1/4, thick muscular region, with inner lumen with 10–12 longitudinal, simple, uniform folds; (2) previous region suddenly opening to wide, bulged area possessing wide transverse fold, encircling almost entire local circumference, except for short region, in which edges turn perpendicularly, originating pair of strong, longitudinal folds running along ~1/2 of penis length; circling these pair of main folds 4–5 secondary longitudinal folds, running parallel to main folds, with interspaces equivalent to their width; pair of main folds with smooth interspace, equivalent to their width, their distal third possessing 5–6 short, uniform, intercalated branches, turned to its pair, up to sudden folds end, smooth ~1/4 of this region's length preceding umbrella-like transverse fold; umbrella-like transverse fold (um) very narrow, possessing only 3 rods; (3) distal ~1/3, slightly narrow, from umbrella-like fold, continuing to epiphallus, internally with 6–7 longitudinal, narrow, simple, separated, uniform folds, 2 of them converging in aperture of vas deferens (vd).

Central nervous system. (Fig. 35B: nr) Same characters as *K. corallina*.

Distribution. So far restricted to type locality.

Habitat. Under rocks or rock crevices.

Etymology. The specific epithet is in honor of ~~the~~ Mauricio Uhle, São Paulo, shell collector, sponsor of expeditions, and enthusiastic contributor to the malacological area.

Measurements. (in mm): holotype (Figs 34A–G): 41.4 by 20.6; paratype MZSP 164889# (Figs 34H–G): 40.0 by 20.1.

Material examined. The types.

Taxonomic remarks. See Discussion item.

***Kora kremerorum* new species** Figs 39–40

ZooBank: urn:lsid:zoobank.org:act:D9F0CAAC-68E9-40AD-8440-7C62765AEF82.

Types. Holotype MZSP 151809; paratypes: MZSP 151810, 19 shells, MNRJ, 1 shell, USNM, 1 shell, all from type locality.

Type locality. BRAZIL. **Minas Gerais**; São João da Ponte, near Olímpio Campos, 15°50’44”S 44°00’03”W, altitude 759 m (W. Vailant-Mattos col., i.2020).

Diagnosis. Size about 45 mm, ~1.9 times longer than wide; dorso-ventral weakly compressed. Apex with same color as remaining shell. Subsutural lighter band present. Peristome white. Delicate spiral striae present. Aperture occupying ~49% of length and ~70% width. Implantation of outer lip slightly horizontal. Inner lip with high middle fold. Umbilicus wide.

Description. Shell. Length up to 44 mm, outline fusiform-globose, 1.9x longer than wide. Color brown, lighter in spire, gradually becoming dark brown in last whorl (Figs 39B, 40B, E, G); subsutural pale band in all whorls well-developed (Figs 39C, D, 40A, E, G, J). Protoconch (Figs 39G, 40I, K) with 2.2 whorls, bluntly pointed; length ~4% of shell length, and ~13% of shell width (Fig. 39F); first whorl smooth, second whorl sculptured by spaced axial riblets. Limit between protoconch and teleoconch weakly visible, weakly prosocline. Teleoconch of ~4.2 whorls successively and uniformly increasing; whorls weakly concave; suture weakly deep. Sculpture well-developed, uniform, delicate axial undulations, forming axial riblets, ~60 in penultimate whorl; also weak, uniform spiral striae, clearer in last whorl, 45–50 in last whorl, each stria composed of minute aligned pits, separated from each other by equivalent distance of their width. Dorso-ventrally slightly flattened (Fig. 39E). Peristome weakly dislocated to right; deflected, except for region of callus. Callus weak (Figs 39A, D, 40A, C, D, F, H). Aperture wide, somewhat dislocated from spire longitudinal axis; length ~49% of shell length, ~70% of shell width. Outer lip inserted distantly from adjacent suture, simple, arched. Inner lip concave, superior half weakly convex, mostly showing outer surface of last whorl; inferior half almost straight (Figs 39A, 40D, F) to weakly convex, concave only inferiorly (Fig. 40A); bearing oblique fold in limit with superior half, having weak elevation preceding its end in inner lip (Figs 39D, 40C, H); tooth length ~31% of peristome length. Umbilicus opened, narrow, partially covered by inferior half of inner lip (Fig. 39E).

Distribution. So far restricted to type locality.

Habitat. Under rocks or rock crevices.

Etymology. The specific epithet is in honor of ~~the~~ Lee and Jan Kremer, shell collectors, expedition sponsors and enthusiastic of the malacological area.

Measurements. (in mm) MZSP 151809 (holotype, Figs 39A–E): 40.8 by 21.8; paratypes MZSP 151810 #1 (Figs 40A–C): 48.6 by 26.7; #2 (Figs 40D–E): 41.9 by 21.2; #3 (Figs 40F–H): 43.5 by 23.6.

Material examined. The types.

Taxonomic remarks. See Discussion item.

***Kora vania* new species** Fig. 41

ZooBank: urn:lsid:zoobank.org:act:F49EC154-BBD7-4FEF-A84F-7CED97C2E780.

Types. Holotype MZSP 165500. Paratypes: MZSP 163776, 2 shells, MZSP 164899, 8 shells, from type locality.

Type locality. BRAZIL. **Minas Gerais**; Montalvânia, E of, 14°25’25”S 44°21’43”W (W. Vailant-Mattos col., vi.2023).

Diagnosis. Size about 30 mm, ~1.9 times longer than wide; lacking dorso-ventral compression. Apex with same color as remaining shell. Subsutural lighter band present. Peristome white. Delicate spiral striae scanty. Aperture occupying ~46% of length and ~65% width. Implantation of outer lip slightly horizontal. Inner lip with high middle fold. Umbilicus narrow.

Description. Shell. Length ~37 mm, outline fusiform-elongate, ~1.9x longer than wide. Color light brown, with darker region half whorl preceding aperture (Figs 41D, C); light subsutural band present (Figs 41C, E). Protoconch (Figs 41G, H) width of 2.25 mm, of ~2 convex whorls, first whorl smooth, axial narrow ribs gradually appearing in second whorl; transition with teleoconch unclear, weakly prosocline; occupying 4.6% of shell length, 15.5% of shell width in holotype. Teleoconch of ~5 whorls successively and uniformly increasing; whorls slightly concave; suture well-marked; spire angle ~45°. Sculpture absent, except for growth lines; surface slightly glossy; spiral striae very weak and sparse, 6–7 in last whorl (Figs 41B, J, M). Transverse section circular (Fig. 41F). Peristome slightly dislocated to right (Figs 41A, I, L); slightly prosocline, ~15° in relation to longitudinal shell axis (Figs 41C, K). Callus thin (Figs 41C, D, K). Aperture wide; length ~46% of shell length, ~65% of shell width. Outer lip inserted very distantly from adjacent suture, in inferior slope; simple, arched. Inner lip concave, superior half weakly convex, constituted by weak callus; inferior half also convex due to strong middle fold (Figs 41A, C, D, I, L); bearing oblique, low fold in limit with superior half (Fig. 41D); tooth length ~40% of peristome length. Umbilicus opened, covered by inferior half of inner lip (Fig. 41E).

Distribution. So far restricted to type locality.

Habitat. Cerrado region.

Etymology. The specific epithet is in apposition, and is a Latinization of the final part of the local of occurrence, the city of Montalvânia.

Measurements. holotype MZSP 165500 (Figs 41A–H): 38.0 by 20.3; paratype MZSP 163776#1 (Figs 41I–K): 34.7 by 17.8; #2 (Figs 41L–M): 38.8 by 20.8.

Material examined. The types.

Taxonomic remarks. See Discussion item.

***Kora curumim* new species** Fig. 42

ZooBank: urn:lsid:zoobank.org:act:CD86562F-7B7D-4149-AE23-E420378975EB.

Types. Holotype MZSP 152077.

Type locality. BRAZIL. **Minas Gerais**; Inaí, Pedra da Fartura, 16°31'37"S 46°49'18"W (W. Vailant-Mattos col., iv.2020).

Diagnosis. Size about 30 mm, ~1.7 times longer than wide; lacking dorso-ventral compression. Apex with same color as remaining shell. Subsutural lighter band absent. Peristome white. Delicate spiral striae absent. Aperture occupying ~50% of length and ~65% width. Implantation of outer lip slightly vertical. Inner lip lacking high middle fold. Umbilicus almost closed. Umbilicus wide.

Description. Shell. Length ~28 mm, outline fusiform-globose, 1.7x longer than wide. Color light brown, intercalating spiral wide darker and lighter bands along whorls (Figs 42A, B, C). Protoconch not seen. Teleoconch of ~4.5 whorls successively and uniformly increasing; whorls weakly concave; suture weakly deep, canaliculated. Sculpture absent, except for growth lines; surface slightly glossy. Transverse section circular. Peristome not dislocated. Callus relatively thick (Figs 42A, D). Aperture wide, not dislocated from spire longitudinal axis; length ~50% of shell length, ~65% of shell width. Outer lip inserted distantly from adjacent suture, simple, arched. Inner lip concave, superior half weakly convex, constituted by callus; inferior half almost straight (Figs 42A, D); bearing oblique, low fold in limit with superior half (Fig. 42D); tooth length ~31% of peristome length. Umbilicus tightly opened, very narrow, covered by inferior half of inner lip (Fig. 42E).

Distribution. So far restricted to type locality.

Habitat. Cerrado region.

Etymology. The specific epithet is derived from Tupy word *curumim*, meaning child, small, allusion to the small size of the specimen.

Measurements. (in mm) MZSP 152077 (holotype, Fig. 42): 27.7 by 16.1.

Material examined. The type.

Taxonomic remarks. See Discussion item.

Genus *Koltrora* new genus

ZooBank: urn:lsid:zoobank.org:act:ABA4B542-3282-4916-B2B0-132ADEBDE415.

Diagnosis. Shell ~~with~~ thin, translucent ~~walls~~. Protoconch with 2 whorls, smooth, with weak axial riblets in last whorl. Teleoconch sculpture only axial, uniform undulations. Umbilicus open~~ed~~. Peristome deflected, wide, weakly dislocated. Ureter totally closed (tubular). Odontophore pair m8 absent; ventral tensor muscle of radula lost. Odontophore cartilages totally fused with each other. Two ducts to anterior lobe of digestive gland. Accessory albumen chamber absent. Uterus with glandular walls. Epiphallus widely opened to penis, with penis muscle subterminal. Penis with transverse, simple inner fold at middle. Calcified epiphragm present.

List of included taxa. Monotypic so far, only *K. pyrostoma* n. sp. the type species.

Etymology. The genus name is in apposition, a contraction of Coltro – in honor to the Coltro brothers (José and Marcus, who have contributed considerably with Brazilian Malacology, with several collects and donation of material) and *Kora*, the genus in which the new one has some similarity.

Gender. Feminine.

Taxonomic discussion. see Discussion item

***Koltrora pyrostoma* new species** Figs 43–48

ZooBank: urn:lsid:zoobank.org:act:E6EC25FB-9685-45F4-BA76-560201653CC5.

Types. Holotype MZSP 163500, 1 complete spm; paratypes: MZSP 151789, 10 spm, MZSP 151791, 2 shells, MZSP 151790, 19 shells, MNRJ, 1 shell, USNM, 1 shell, all from type locality. BRAZIL. **Bahia**; Bom Jesus da Lapa (W. Vailant-Mattos col.), near Pirâmide Luxor Hotel, 13°15’35”S 43°25’12”W, altitude 500–520 m, MZSP 152017, 21 shells (17.iv.2019)

Type locality. BRAZIL. **Bahia**; Bom Jesus da Lapa, way to Morrão, 13°09’45”S 43°18’33”W, altitude 440–460 m (W. Vailant-Mattos col., 17.iv.2019).

Diagnosis. Size about 30 mm, ~1.8 times longer than wide; dorso-ventrally weakly compressed. Apex with same color as remaining shell. Subsutural lighter band absent. Peristome white. Delicate spiral striae absent. Aperture occupying ~50% of length and ~57% width. Implantation of outer lip slightly vertical. Inner lip lacking high middle fold. Umbilicus narrow. Secondary columellar muscles with 4 insertions in left and 2 in right. Absence of pairs of m1v. Jaw rectangular. Odontophore cartilages ~100% fused. Pair m10 broad, absence of m8. Double anterior duct to digestive gland. Carrefour duct narrow and long, inserted in tip of spermoviduct. Albumen chamber sac-like. Penis ~50% of spermoviduct length, lacking umbrella-like fold; epiphallus ~25% of penis length; penis muscle subterminal in epiphallus.

Description [illegible]stinctive in anatomy). Shell. (Figs 43, 44A–E, 45A–D, G–H) length up to 32 mm, outline fu[illegible]rm-elongate, ~1.8 longer than wide. Color uniform pale beige to cream, walls translucent. Protoconch (Figs 43H, I, 44D, E, 45C) with 2 whorls, bluntly pointed; length ~7% of shell length, and ~6.5% of shell width (Fig. 43G); mostly smooth, barely sculptured by axial riblets in last whorl. Limit between protoconch and teleoconch weakly visible, weakly prosocline. Teleoconch of ~4 whorls successively and uniformly increasing; whorls weakly concave; suture weakly deep; sculpture absent, except for growth lines and delicate axial, uniform undulations, ~60 in penultimate whorl. Dorso-ventrally softly flattened (Figs 43F, G). Peristome weakly dislocated to right, slightly oblique; deflected. Callus plane, relatively well-

developed in adult (Figs 43A, D, E, 42A), weak in young (Figs 44D, 45A, D). Aperture wide, somewhat dislocated from spire longitudinal axis; length ~50% of shell length, ~57% of shell width. Outer lip inserted distantly from adjacent suture, simple, arched. Inner lip concave, superior half weakly convex, mostly composed of plane callus; inferior half weakly straight to weakly convex; bearing very low oblique fold in limit with superior half (Figs 43E, 44B). Umbilicus opened, narrow, partially covered by inferior half of inner lip (Fig. 43F).

Epiphragm. Present in few specimens (Fig. 45D) calcified, thin, occluding entire aperture; dislocated posteriorly from peristome.

Head-foot. (Figs 45J, 44B) of normal shape, resembling *K corallina*. Color bluish beige with some red pigment in furrows of mosaic of dorsal foot integument (Figs 45A, G: ft, H), becoming uniformly bluish beige in preserved specimens (Fig. 46J). Clear oblique furrow (Fig. 45J: fe) running at right in integument from pneumostome (pn) up to very anteriorized genital pore (Fig. 45E: fp). Columellar muscle thick, 1.2 whorls in length. Main columellar bundle (cm) ~3/4 of foot width. Each secondary columellar/cephalic muscles with ~1/3 of main columellar bundle (cm) width. Right cephalic muscle (Fig. 46B: cr) with 2 broad insertions, being medial insertion broader, with also broad tentacular, ommatophore muscles and small genital muscles as more lateral branches; left cephalic muscle (cl) similarly organized, but with 4 slightly narrow aligned insertions. Pedal gland (pg) short, weakly protruding in posterior region of buccal area (mo).

Mantle organs. (Figs 45J, K, 46A) with similarities to *K. corallina*, distinctions and remarks following. Mantle edge (mb) thick, strongly red pigmented (Figs 45A, G: mb, H); seen through shell translucency (Figs 43C, 45B, D); red pigment disappearing in preserved specimens (Figs 45E, J, K). Right pallial fold of mantle edge well-developed (Fig. 46A: mf), with projected, bluntly pointed left end. Pneumostome (Fig. 46A: pn) bearing exclusively air entrance and urinary aperture (ua); anus (an) as separate aperture located at right, adjacent to pneumostome. Lung of ~1.5 whorls in length, ~twice long than wide; scarcely possessing minute longitudinal muscle fibers in its wall seen by translucency, more concentrated in region near pulmonary vein. Pulmonary venation well-developed, especially in region preceding pneumostome; posterior region of pulmonary vein (cv) protruded, relatively straight; left 2/3 with only pair of narrow intercalated longitudinal vessels; right 1/3 mostly having perpendicular vessels rather uniformly distributed, weak in middle, becoming taller in region adjacent to kidney and anteriorly; pulmonary vessel bifurcating very close to pneumostome (Figs 45K, 46A: cv), with other subterminal branches overlapping right vessels producing strange anastomoses (Figs 45K, 46A). Collar vessel (co) also well-developed, running slightly away from mantle edge. Reno-pericardial area of light brown color, slightly triangular with slender anterior prolongation (Figs 45F, 46A: ki), occupying ~35% of cavity length and ~65% of its width (details below). Rectum (rt) wide. Primary (Figs 45F, 46A: up) and secondary (us) ureters entirely closed (tubular), relatively narrow, aperture (ua) simple, directly outside at right in pneumostome region.

Visceral mass. (Fig. 46C) ~3 whorls in length, with similar attributes as *K corallina*. Except for stomach smaller and slightly more anterior localized.

Circulatory and excretory systems. (Figs 45F, 46A) general Bauplan similar to *K. corallina*, with following remarks. Pericardium (pc) slightly broader. Kidney (ki) size reported above; slightly triangular, as long as wide, with anterior slender sharp projection. Nephrostome at

anterior tip of this projection, inside primary ureter beginning. Internally organized as successive tall glandular folds (Fig. 45F: kl), of relative similar height.

Digestive system. (Fig. 44C) general organization resembling that of *K. corallina*, distinctions and remarks following. Jaw plate (Fig. 45I) thin, yellow, translucent, ~1.5x broader than long; cutting edge softly convex, slightly notched at middle; sculptured by successive, rather uniform, transverse, wide folds. Buccal mass with radular sac large, bulging ~1/3 of its posterior side, sheltering coiled radular sac (rs), covered by transparent membrane (mr). Dorsal surface of oral cavity with broad and low pair of dorsal folds (Fig. 47A: df), width of each almost 1/2 of dorsal wall width; not touching with each other in median line, keeping shallow dorsal chamber. Odontophore (Fig. 46C: od) with ~50% of buccal mass volume. Odontophore muscles (Fig. 47B) with overall features of *K., corallina*, with following remarks: **m1v**, absent; **m1l**, relatively wide pair of dorso-lateral protractor muscles, originating in lateral region of mouth, running short distance towards anterior, inserting in latero-dorsal region of buccal mass surface (Fig. 46C); **m3**, not detected; **m4**, with separated branch connected to radular sac and m7a (Fig. 47B: ir); **m5**, originating only median portion on postero-ventral region of odontophore cartilages, ~80% on m4; **m6**, absent; **m7**, narrow and slender, originated as 2 branches in posterior region of fusion between both cartilages, run inside dorsal region od radular sac; **m8**, absent; **m10**, broad. Pair of odontophore cartilages almost entirely fused with each other in their anterior-medial edge (Fig. 47B: **oc**). Radular sac (Fig. 46C: rs) long, performing loop inside translucent membrane (mr) bulded posteriorly from odontophore. **Radula.** (Figs 44F–H) ~2.5 times longer than odontophore. Composed of uniform, similar kind of tooth, with no clear differentiation among rachidian, lateral or marginal teeth, ~100 pairs of teeth per row. Each tooth with elongated base, 6–7 times longer than wide, placed longitudinally, very close to each other; proximal end rounded; distal end bearing long, curved cusp, slightly longer than base; cusp slightly flattened, base reinforced by central fold gradually tapering up to ~70% of cusp length; cusp tip flattened, slightly broader, rounded-barely spoon-like, possessing shallow subterminal, longitudinal, short furrow. All teeth straight aligned per row, except for 8–10 more marginal teeth, slightly arched aligned (Figs 44G, H).

Salivary glands covering ~1/20 of esophagus length, located between anterior and second quarter of esophageal length (Fig. 46C: sg), forming two elliptic, white, thin masses. Each salivary duct differentiable in anterior side of glands (sd). Salivary duct running in both sides of esophageal origin, penetrating buccal mass wall in region close to buccal ganglia (Fig. 47A: bg), running immersed in buccal dorsal wall along ~1/6 its length (Fig. 47A). Salivary ducts opening as small pores (sa), located medially in posterior region of oral cavity, at anterior tip of pair of short longitudinal, broad folds (ef). Esophagus (Fig. 46C: es) entirely narrow, simple; inner surface with narrow, separated longitudinal folds. Esophageal duct to digestive gland (Fig. 46C: dd inferior) long, located at short distance from stomach, connected to anterior lobe of digestive gland. Stomach (st) small, narrow, curved, not bulging; position and size described above (visceral mass); gastric walls thin, not muscular; inner surface mostly smooth, lacking folds. Duct to anterior lobe of digestive gland at short distance from intestine intersection (dd superior) narrow; strongly bifid, right branch running perpendicularly to right, left branch long, running towards anterior covered ventrally by esophageal duct. Duct to posterior lobe of digestive gland located short distance from intestinal origin, directed towards opposite side (dp), as wide as other ducts, bifurcating only after long distance. Intestine (in) entirely narrow, performing its usual wide sigmoid loop in anterior lobe of digestive gland. Rectum and anus position described above (pallial cavity) (rt, an). Anus sessile, as slit in right end of mantle edge directly turned outside (Fig. 46A: an).

Reproductive system. (Figs 47C–F, 48B, C) general structures similar to preceding species, remarks and distinctions following. Gonad composed of 5–6 lobes with minute digitiform acini. Hermaphroditic duct (Fig. 47D: hd) narrow; coiled portions occupying middle 2/3, with narrow coils; insertion preceded by straight region, and strongly curve (Fig. 47C: hd). Seminal receptacle (Figs 47C, D: sr) small, curved, long, tip pointed, ~6 times longer than wide, flattened. Fertilization complex or carrefour (Fig. 47C: ca) simple, as narrow, duct-like region in receptacle base, ~1/2 of its length; totally immersed in albumen gland, inserting in posterior end of spermoviduct, at side of tip of wide albumen gland duct. Albumen gland (Fig. 47D: ag) ~6 times larger than gonad (~1/3 whorl). Albumen gland duct subterminal, conic, connected laterally to distal end of spermoviduct (Fig. 47C: ad). Albumen chamber (Fig. 47D: ac) as wide sac in spermoviduct initial portion, widely connected to distal end of spermoviduct (eo). Spermoviduct (Figs 47D, 48B: eo) of ~1 whorl in length, slightly narrower than albumen gland, ~10 times longer than wide. Secondary albumen chamber absent. Prostate narrow (pt), ~1/4 of spermoviduct diameter (Figs 47E, F); uterus with thick glandular walls posteriorly, gradually becoming thinner glandular walled anteriorly; broadly, transversally, relatively uniformly folded (Figs 47D, 48B: ut). Sperm inner longitudinal fold as simple, low, very thick fold (Figs 47E, F: sp); a second small fold gradually appearing only in basal end; both folds fusing with each other, originating vas deferens, slightly anterior to end of uterine level (Fig. 48B: vd). Vas deferens uniformly narrow, uncoiled (Figs 47D, 48B: vd). Genital muscle in intersection vagina and penis (Figs 47D, 48B: gm), wide and long. Bursa copulatrix (bc) and its duct (bd) of usual position, with ~70% of spermoviduct length (Fig. 47D); bursa duct weakly muscular (Fig. 48B: bd). Free oviduct (fo) and vagina (vg) simple, possessing 2 very wide, low, longitudinal, simple folds (Fig. 48B). Penis almost straight, ~50% of spermoviduct length (Fig. 47D: pe); epiphallus as continuation of penis, penis muscle inserted subterminally in epiphallus (Figs 47D, 48C: pm), short, simple. Penis shield (Fig. 47D: ps) with transverse muscle fibers, with ~1/10 penis length. Penis wall weakly muscular, except for thick muscular walls in region adjacent to penis shield (Fig. 48C). Epiphallus (eh) ~1/4 of penis' length, amply opened to penis; only vas deferens insertion marking its limit (Fig. 48C: vd). Epiphallus inner surface with 8–10 small, low, parallel folds. Internal penial arrangement of folds clearly with three regions (Fig. 48C): (1) basal 1/4, possessing only 3–4 longitudinal, broad, low, simple folds, correspondent to penial muscular portion; (2–3) remaining ¾ portion, divided at middle by transverse fold, of rounded profile, surrounding entirely penis wall; inferior half having pair of longitudinal folds united with each other in distal end, close to transverse fold, in opposed end both folds finishing abruptly, between both folds almost smooth space, surrounding them 5 secondary longitudinal folds parallel to them; distal half with similar arrangement than inferior half, but with fusion between both folds more ample, and both folds running longitudinally very close from each other, surrounding them 6–7 low, wide, longitudinal secondary folds, all them continuing to epiphallus, with some converging to vas deferens aperture.

Central nervous system. (Figs 48A, D) characters of ganglia and statocysts virtually similar to those described for *K. corallina*. Except for cerebral (cc) and pedal commissures slightly longer; and by pleural ganglia (pl) slightly proportionally larger.

Distribution. So far know for region of Bom Jesus da Lapa, Bahia.

Habitat. Under rocks, limestone areas.

Measurements (in mm): MZSP 163500 (holotype, Figs 43 A–E): 29.1 by 16.3; MZSP 191791 (Figs 43A): 31.9 by 17.2.

Material examined. All types.

Etymology. The specific epithet is a junction of the [illegible]tin words *pyro*, meaning fire, and Greek *stoma*, meaning mouth, in allusion to the red c[illegible] of the aperture when the animal is alive (Figs 43A, G, H), [illegible]ecting outside by shell translucency (Fig. 45B: mb).

Taxonomic remarks. See Discussion item.

Genus *Neopetraeus* Martens, 1885

Complemented diagnosis. Shell elongated to obese; with mosaic of spots and bands on light basal color; higher variation of sculptures. Protoconch of ~2 smooth whorls, variating from smooth up to reticulated, from rounded to carinated. Umbilicus usually well-developed, resulted of columellar hollow area, producing inner lip usually with middle region bulged. Ureter 50–30% opened (as groove). Both pairs of dorsal tensor muscles of radula (m4 and m5) as indistinct single mass; odontophore pair m8 absent; ventral tensor muscle of radula present. Accessory albumen chamber present.

***Neopetraeus lobbii* (Reeve, 1849)** Figs 49–53

Bulimus lobbii Reeve, 1849[24]: pl 71 (fig. 516).

Neopetraeus lobbii: Pilsbry, 1897[25]: 177–178 (pl. 29 figs 24–26) (+ ancient synonymy); Breure & Araujo, 2017[26]: 82–83 (fig. 31F); MolluscaBase, 2023[8] (fig).

Types. Lectotype NHMUK 1975431, 1 shell (Figs 49J–M) (examined)

Type locality. Balsas, Banks of the Amazon near Balsas, Peru [24].

[illegible]tinctive description.

Shell. (Figs 49A–G, J–M) length up to 44 mm, outline fusiform-elongate, ~2.2 longer than wide. Color uniform white to very light beige as base, plus axial bands randomly variating from dark, middle and light brown, from uniformly (Figs 49J–M) to randomly (Figs 49A–D) distributed, bands from suture to suture, in last whorl entering in columellar area and umbilicus (Figs 49A, D, J), concentrated in region preceding peristome (Figs 49C, D, K). Protoconch described as having 2.25 whorls with delicate reticulate [25] (only axial riblets detected in damaged specimen – Figs 49F, G). Teleoconch of ~5 whorls successively and uniformly increasing; whorls weakly concave; suture weakly deep; sculpture absent, almost smooth and shining, except for growth lines and delicate axial, uniform undulations in region preceding aperture. Transverse section circular (Figs 49F, M). Peristome weakly dislocated to right, ample; deflected. Callus weak, almost absent (Figs 49A, D, J). Aperture wide, somewhat dislocated from spire longitudinal axis; length 41–50% of shell length, 57–70% of shell width. Outer lip inserted distantly from adjacent suture, simply arched. Inner lip concave, superior half weakly convex, mostly composed of exposed last whorl; inferior half weakly straight; bearing very low oblique fold in limit with superior half (Figs 49C, D). Umbilicus opened, narrow, partially covered by inferior half of inner lip (Fig. 49F). More details in [24, 25].

Head-foot. (Fig. 50B) of normal shape, resembling *K corallina*. Distinctions and remarks following. Columellar muscle (cm) and its secondary units (cl, cr) narrower. Right cephalic

muscle (Fig. 50B: cr) particularly narrow, with 4 slim, aligned, isometric insertions, plus ommatophore and small genital muscles as more lateral branches; left cephalic muscle (cl) similarly organized, but ~3-times wider, with only 3 insertions, 2 of them broad insertions, being middle insertion larger, plus ommatophore muscle as more lateral branch, medial-most insertion very narrow and more posteriorly inserted. Pedal gland (pg) long and narrow, weakly protruding in posterior region of buccal area (mo).

Mantle organs. (Figs 50A, 51A) with similarities to *K. corallina*, distinctions and remarks following. Mantle edge (mb) very thick, lacking secondary folds or glands. Pneumostome (pn) bearing exclusively air entrance and urinary groove (ur); anus (an) as separate aperture located at right, adjacent to pneumostome. Lung of ~1.5 whorls in length, ~3x longer than wide; lacking longitudinal muscle fibers. Pulmonary venation well-developed only in region preceding pneumostome; entire pulmonary vein (cv) protruded, relatively broad; left 2/3 lacking longitudinal vessels, bearing only sparse transverse minute vessels; right 1/3 mostly having perpendicular vessels rather uniformly distributed, weak in middle and posterior regions, becoming taller anteriorly; pulmonary vessel bifurcating very close to pneumostome, anterior quarter of pulmonary vessel with relatively symmetric set of oblique, wide, bifurcating vessels. Reno-pericardial area of light brown color, slightly triangular (ki), occupying ~20% of cavity length and ~50% of its width (details below). Rectum (rt) wide. Primary (Fig. 50A: up) and most of secondary (us) ureters entirely closed (tubular), relatively narrow; ~20% of anterior region of ureter opened (as groove) (ur), running like this up to pneumostome, in urinary furrow flanking perpendicularly inner edge of pneumostome (ur).

Visceral mass. (Fig. 51A) ~3 whorls in length, with similar attributes as *K corallina*. Except for stomach smaller and slightly more anterior localized.

Circulatory and excretory systems. (Figs 50A, 51A) general Bauplan similar to *K. corallina*, with following remarks. Pericardium (pc) slightly narrower. Kidney (ki) size reported above; triangular, 1.5x longer than wide. Internally organized as 5 successive tall glandular folds (Fig. 50A: ki), of relative similar height.

Digestive system. (Figs 50C, D, 51A–C) general organization resembling that of *K. corallina*, distinctions and remarks following. Jaw plate (Fig. 49I) thick, yellow, curved, ~1.5x broader than long; cutting edge chevron-like; sculptured by successive, rather uniform, oblique, wide folds, convergent at middle. Buccal mass with radular sac small, weakly bulging beyond buccal mass (Fig. 50C: rn). Dorsal surface of oral cavity (Fig. 51B) with broad and low pair of dorsal folds (df), width of each ~1/3 of dorsal wall width; shallow dorsal chamber between them. Odontophore (Fig. 50C: od) ~70% of buccal mass volume. Odontophore muscles (Figs 50C, D, 51B, C) with overall features of *K. corallina*, with following remarks: **m1v**, broad and long; **m1l**, relatively wide pair of dorso-lateral protractor muscles, originating in lateral region of mouth, running short distance towards anterior, inserting in latero-dorsal region of buccal mass surface (Fig. 50C); **m1d**, small pair of dorsal protractor muscles of buccal mass, originating in dorsal region of mouth, running towards posterior covering dorsal surface of buccal mass, inserting in 2/3 of buccal mass length lateral surface (Fig. 50C); **m3**, as cover of transverse fibers covering entire posterior surface of odontophore; **m3l**, pair of dorso-ventral muscles located each one in each side of esophageal insertion, covering m3, running up to m2 insertion; **m4**, very wide, composed of several separated V-shaped layers (Figs 50D, 51C); **m5**, incorporated to m4, not individualized; **m6**, absent; **m7**, absent; **m8**, absent; **m10**, broad; **m11**, pair of ventral tensor muscles of radula, narrow, originating in posterior edge of ventral surface of cartilages, running between cartilages and mj, inserting splaying in subradular

membrane in its region adjacent of tip of cartilages; **mj**, inserted in latero-ventral edged of cartilages, forming ventral platform (Fig. 51C). Pair of odontophore cartilages ~50% fused with each other in their anterior-medial edge (Fig. 50D: **oc**). Radular sac (Fig. 50D: rs) short, slightly longer than odontophore. **Radula** (Fig. 52) Composed of uniform, similar kind of tooth, with weak differentiation among rachidian, lateral or marginal teeth, ~50 pairs of teeth per row; each row strongly arched in margins, widely V-shaped in center (Figs 52A, B). Rachidian with rectangular base, length ~twice width; cusp stubby, triangular, located in distal end of base, with ~half of base length; situated perpendicularly to base, slightly arched inwards; tip bluntly pointed. First lateral teeth similar to rachidian, but with ~double cusp, with rounded tip, and appearance of minuscule subterminal inner secondary cusp (Fig. 52D); remaining lateral teeth with cusp as large as base, and secondary cusp gradually increasing towards external up to becoming ~half main cusp's size (Fig. 52C). No clear border between lateral and marginal teeth. Marginal teeth similar to lateral teeth, narrowing gradually towards margins, where row arching; more marginal teeth very narrow (Fig. 52E). Salivary glands covering ~1/2 of esophagus length, located between anterior and second quarter of esophageal length (Fig. 51A: sg), forming single multilobed, flattened, yellow mass. Each salivary duct differentiable in anterior side of glands (sd). Salivary duct running in both sides of esophageal origin, penetrating buccal mass wall in region close to buccal ganglia (Fig. 51A: sd), running immersed in buccal dorsal wall along ~1/6 its length (Fig. 51B). Salivary ducts opening as small pores (Fig. 51B: sa), located medially in zigzag portion of posterior region of dorsal folds. Esophagus (Fig. 51A: es) with irregular width along its length; inner surface with narrow, separated longitudinal folds. Stomach (Fig. 51A: st) small, narrow, curved, weakly bulging; position and size described above (visceral mass); gastric walls thin, not muscular; inner surface mostly smooth, lacking folds. Duct to anterior lobe of digestive gland at short distance from intestine and esophageal intersections (dd) broad (as broad as intestine); bifid after some distance, with branches to both regions of anterior lobe of digestive gland (da). Duct to posterior lobe of digestive gland small, located in middle region of gastric ventral wall (dp). Intestine (Fig. 51A: in) relatively narrow, performing its usual wide sigmoid loop in anterior lobe of digestive gland. Rectum and anus position described above (pallial cavity) (rt, an). Anus sessile, as slit in right end of mantle edge directly turned outside (Fig. 51A: an).

Reproductive system. (Figs 51D, 53) general structures similar to preceding species, remarks and distinctions following. Gonad not clearly divided in lobes, acini not digitiform. Hermaphroditic duct (Fig. 53B: hd) broad; dark pigmented, strongly coiled, mainly its middle region; insertion preceded by rather straight region (Fig. 53D: hd). Seminal receptacle (Fig. 53D: sr) small, straight, tip curved, ~4 times longer than wide, cylindric. Fertilization complex or carrefour (Fig. 53D: ca) simple, tapering region in receptacle base, slightly longer than it, still possessing bulging region opposed to hermaphrodite duct insertion; totally immersed in albumen gland; insertion very narrow, in posterior end of spermoviduct, at tip of narrow albumen gland duct (ad) and intersection of albumen chamber (ac). Albumen gland (Fig 53B: ag) ~as large as gonad (~1/4 whorl). Albumen gland duct subterminal, narrow (Fig. 53D: ad), connected laterally to distal end of spermoviduct/albumen chamber. Albumen chamber (Fig. 53D: ac) as simple, bulged curve connected to distal end of spermoviduct (eo). Spermoviduct (Figs 53B–D: eo) of ~1.5 whorl in length, slightly narrower than albumen gland, ~20 times longer than wide. Secondary albumen chamber large (~half of albumen gland), balloon-like, with wide chamber, narrow duct connected just anterior to primary albumen gland chamber (Figs 53B, D: as). Prostate narrow (pt), ~1/6 of spermoviduct diameter (Figs 51D, 53B); uterus with walls not glandular (Figs 51D, 53C: ut); folds performing irregular zigzag (Fig. 53B: ut).

Sperm inner longitudinal fold double, tall, narrow posteriorly (Fig. 51D: sp); one of them becoming still taller, curving covering other, becoming duct like (Fig. 53C: sp), covering vas deferens origin, slightly anterior to end of uterine level (Fig. 53C: vd). Vas deferens uniformly narrow, uncoiled (Fig. 53B: vd). Genital muscle small, in vaginal base (Fig. 53B: gm). Bursa copulatrix (bc) and its duct (bd) of usual position, with ~70% of spermoviduct length (Fig. 53B); bursa duct weakly muscular (Fig. 53C: bd). Free oviduct (fo) and vagina (vg) simple, possessing 2 very wide, low, longitudinal, simple folds (Fig. 53C). Penis almost straight, ~50% of spermoviduct length (Fig. 53D: pe), base wider, gradually tapering up to pointed epiphallus; epiphallus as continuation of penis, penis muscle inserted terminally at epiphallus tip (Figs 53A, B: pm), very short, simple. Penis shield (ps) with ~1/4 penis length. Penis wall weakly muscular, all along its length (Fig. 53A). Epiphallus (eh) ~1/3 of penis' length, amply opened to penis; only vas deferens insertion marking its limit (Fig. 53A: vd). Epiphallus (eh) smooth, except for single, narrow, longitudinal fold (Fig. 53A: ei), ending by side of vas deferens aperture. Internal penial arrangement of folds clearly with 2 regions (Fig. 53A): (1) basal 1/3, possessing only 4 longitudinal, narrow, low, simple folds, with broad interspaces; (2) separated by transverse, low fold (pf) in which longitudinal folds end, occupying remaining 2/3 of penis length, bearing only 6–7 longitudinal, narrow folds, initially more evident and separated from each other, gradually becoming close from each other and weaker towards posterior, up to disappearing at some distance from epiphallus insertion.

Central nervous system. (Fig. 51E) characters of ganglia and statocysts virtually similar to those described for *K. corallina*. Except in being apparently more concentrated, and with shorter connectives. Pair of pleural ganglia (pl) located closer to pedal ganglia. Nerve ring located posteriorly to buccal mass (Fig. 51A: nr).

Distribution. Amazon region of Peru.

Habitat. Branches of *Jatropha* sp [24], Pata trees and cacti [25].

Measurements (in mm): MZSP 158045 (Figs 49A–E): 40.2 by 18.1; Lectotype NHMUK 1975431 (Figs 49J–L): 44.4 by 20.0.

Material examined. PERU. **Amazonas**; Balsas, Chacanto, 6°50'36.45"S 78°01'43.92"W, 880 m altitude, MZSP 158045, 1 spm (Valentin Mogollón col., ii.2016; Frederico Gutierres leg.).

Taxonomic remarks. See Discussion item.

***Neopetraeus tessellatus* (Shuttleworth, 1852)** Figs 54–57

Bulimus tessellatus Shuttleworth, 1852[27]: 200.

Neopetraeus tessellatus: Breure, 1978[28]: 217 (plus additional synonymy); Breure & Araujo, 217[26]: 83 (fig. 31G) (plus additional synonymy).

Type locality: Peruvia.

Distinctive description. Shell. (Fig. 54) length ~40 mm, outline fusiform-globose, ~1.5 longer than wide. Color uniform white to light beige as base, plus mosaic of dark to light brown spots, more evident in 2 last whorls, randomly organized, but successively repeated, modifying gradually along whorl; sometimes spirally organized [26: fig. 31G] sometimes axially (Figs 54H). Protoconch with 2.5 whorls, slightly shouldered; sculptured by strong axial, uniform riblets,

with interspaces with twice their width, fulfilled by narrow axial lines (Figs 54J, K), these axial lines very close from each other, from suture to suture (Fig. 54K); limit with teleoconch clear, slightly prosocline (Fig. 54J). Teleoconch of ~5 whorls successively and uniformly increasing; whorls weakly concave; suture weakly deep; sculpture absent, almost smooth, opaque, except for growth lines and delicate axial, uniform undulations in region preceding aperture. Transverse section circular (Figs 54F, G). Peristome weakly dislocated to right, ample; deflected. Callus weak, almost absent (Figs 54A, B, E, H). Aperture relatively narrow, somewhat dislocated from spire longitudinal axis; length ~57% of shell length, ~65% of shell width. Outer lip inserted not so distant from adjacent suture, simply arched. Inner lip concave, superior half weakly convex, mostly composed of exposed last whorl and thin callus; inferior half weakly straight; fold formed by umbilicus occupying entire inferior half (Figs 54E, F). Umbilicus widely opened, narrow, ventrally covered by inner half of inner lip (Fig. 54F). More details in [27, 28].

Head-foot. Same characters of *N. lobbii*, except for having a columellar muscle ~25% shorter, and left secondary columellar muscle ~20% broader.

Mantle organs. (Fig. 56A) with similarities to *N. lobbii*, distinctions and remarks following. Mantle edge (mb) also very thick and lacking secondary folds or glands. Lung of only ~1 whorl in length, almost as long as wide; lacking longitudinal muscle fibers. Pulmonary venation much less-developed, more visible in region preceding pneumostome; entire pulmonary vein (cv) protruded, relatively broad; left 2/3 lacking longitudinal vessels or any visible vessels, except for some few more developed in region of pneumostome; right 1/3 mostly having perpendicular, narrow vessels rather uniformly distributed, almost absent posteriorly, weak in middle and anterior regions; pulmonary vessel trifurcating very close to pneumostome. Reno-pericardial area of pale beige color, slightly triangular (ki), occupying ~40% of cavity length and ~50% of its width (details below). Rectum (rt) wide. Primary (up) and ~40% of secondary (us) ureters closed (tubular), relatively narrow; ~60% of anterior region of ureter opened, as widely opened groove (ur), with elevated edges, smooth inside, running like this up to pneumostome, in urinary furrow flanking anus (an) left edge.

Visceral mass. with similar attributes as *N. lobbii*.

Circulatory and excretory systems. (Figs 55D, 56A) general Bauplan similar to *N. lobbii*, except in being proportionally larger, and in having kidney lobe almost entirely solid (not organized in folds), with single, central, tight lumen (Fig. 55D).

Digestive system. (Figs 56B, C) general organization resembling that of *N. lobbii*, distinctions and remarks following. Jaw plate (Fig. 55F) thin, yellow, strongly curved, ~2x broader than long; cutting edge deeply concave; sculptured by successive, fewer, wide, oblique, wide folds, convergent at middle. Buccal mass with radular sac small, weakly bulging beyond buccal mass (Figs 55B, 56B, C: rn). Dorsal surface of oral cavity (Fig. 55A) similar, but lacking zigzag posterior portion, with dorsal folds (df) broad up to their posterior region, overlapping posteriorly. Odontophore (Fig. 56B: od) ~50% of buccal mass volume. Odontophore muscles (Figs 56B, C) with overall features of *N. lobbii*, with following remarks: **m1v** and **m1l**, similar, elongated; **m1d**, double; **m3** and **m3l**, similarly organized; **m4**-**m5**, also composed of several separated V-shaped layers; **m7**, pair present and broad, originated close to fusion of both cartilages (Fig. 56C). Pair of odontophore cartilages ~70% fused with each other in their anterior-medial edge (Fig. 56C: **oc**). **Radula** (Figs 55G–I) composed of rachidian plus ~40 pairs per row, with similar features as *N. lobbii*, except for: rachidian ~30% wider (Fig. 55G), with concave posterior edge; lateral and marginal teeth lacking secondary cusps and

more inclined externally (Figs. 55H, I). Salivary glands covering ~1/3 of esophagus length, located at middle of esophageal length (Figs 55B, 56B: sg), thick, forming single multilobed, yellowish-cream mass. Each salivary duct very broad, differentiable in anterior edge of glands (sd); left duct bifid. Esophagus (Figs 55B, 56B: es) initially narrow; posterior half with large crop, as enormous dilatation (Figs 55B, 56B: ed) bulged anteriorly, tapering posteriorly; internally bearing longitudinal, relatively regular folds (ef). Remaining gastric and intestinal features similar to those of *N. lobbii*, except for intestine having clear transverse wide fold in region adjacent to pericardium (Fig. 55C: iv) and to anterior aorta (aa).

Reproductive system. (Figs 55E, 57) general structures similar to *N. lobbii*, remarks and distinctions following. Gonad not clearly divided in lobes, acini not digitiform; located close to end of visceral whorls (Fig. 57A: go). Hermaphroditic duct (Fig. 57A: hd) narrow, slightly coiled along almost its entire length; insertion preceded by rather straight region (Fig. 57B: hd). Seminal receptacle (Fig. 57A, B: sr) small, straight, tip rounded, ~6 times longer than wide, cylindric. Fertilization complex or carrefour (Fig. 57B: ca) simple, small conic region in receptacle base, succeeded by elongated, narrow duct as long as receptacle slightly; insertion very narrow, by side of posterior end of spermoviduct, at tip of narrow albumen gland duct (ad). Albumen gland (Fig 57A: ag) ~2x larger than gonad (~1/3 whorl). Albumen gland duct subterminal, narrow (Fig. 57B: ad), connected frontally to distal end of spermoviduct and carrefour insertion. Albumen chamber (Figs 57A, B: ac) as simple, bulged curve connected to distal end of spermoviduct (eo). Spermoviduct (Figs 57A, C: eo) of ~1.5 whorl in length, ~3x narrower than albumen gland, ~15 times longer than wide. Secondary albumen chamber present, ~15% of albumen gland size, balloon-like, with wide chamber, narrow duct connected just anterior to primary albumen gland chamber (Figs 57A: as). Basic composition of spermoviduct and genital muscle similar to those of *N. lobbii*, except in lacking closure of sperm groove (sp). Bursa copulatrix (bc) and its duct (bd) with ~90% of spermoviduct length (Fig. 57A); bursa duct mostly weakly muscular, except for its thicker walls of anterior region (Fig. 57C: bd). Penis general attributes similar to those of *N. lobbii*, except in being longer (~90% of spermoviduct length); in having uniform width along its length (not tapering posteriorly); epiphallus (eh) proportionally shorter and broader, internally having strong longitudinal fold (Fig. 55E: ei), flanked by secondary, small oblique folds; wall uniformly more muscular; and in lacking bulged basal region with transverse fold (transverse fold absent).

Central nervous system. Same characteristics of *N. lobbii*.

Distribution. Lima region of Peru.

Habitat. No data.

Measurements (in mm): MZSP 158044: #1 (Figs 54A–E): 36.9 by 23.7.

Material examined. PERU. **Lima**; Cañón del Pato, Kiman Aullu, 8°46’25.85”S 77°52’26.28”W, 840–870 m altitude, MZSP 158044, 2 spm (Valentin Mogollón col., 18.vi.2010; Frederico Gutierres leg.).

Taxonomic remarks. See Discussion item.

Genus *Anctus* Martens, 1860

***Anctus angiostomus* (Wagner, 1827)** Fig. 59

The taxonomic treatment and anatomical description by Simone (1998) [16] are particularly complete. Some missing data of that, necessary to this species be included in the phylogeny of the present paper, are here completed.

Shell. (Figs 59A–E) with very elongated peristome (Figs 59A, C), wide umbilicus (Figs 59A, D), delicate undulations if sculpture (Fig. 59B). Protoconch dark brown, smooth, of 2 whorls (Fig. 59E).

Head-foot. Secondary columellar muscles with 2 wide insertions in right component, and 4 wide insertions in left component.

Digestive system. (Figs 59G, H, J, K) jaw plate wide horseshoe-shaped, with broad transverse folds (Fig. 59H). Buccal mass with well-developed pair m1l and 2 pairs of m1v (Fig. 59G), also pair m2a and pair m3 dorso-ventral in esophageal-odontophore limit. Odontophore cartilages (oc) almost 100% fused with each other (Fig. 59J), with pair m5 covering medial region of m4; m7 wanting. Salivary ducts (Fig. 59K: sd) broad; aperture of salivary glands (sa) very posteriorized on dorsal folds (df).

Reproductive system. (Fig. 59I) presence of bulged region by side of base of seminal receptacle (sr). Carrefour (ca) narrow and elongated duct inserted in albumen gland duct (ad). Albumen chamber (ac) as curve preceding spermoviduct.

Central nervous system. (Fig. 59F) similar to preceding species, cerebral commissure relatively wide. Pleural ganglia (pl) close to pedal ganglia (pp), bearing commissure.

Measuremens (in mm): MZSP 27937: 28.1 by 14.0.

Discussion

Taxonomic discussion

The present paper is almost entirely performed in a comparative context. The descriptions ~~themselves, as mentioned above, are mostly in a distinctive style,~~ [illegible]npar[illegible] each taxon with the first description, highlighting the differences, and only showca[illegible]g sor[illegible]nore important similarities. The set of characters that defines each species is reported in the diagnoses. Furthermore, the main differences among the studied species (new or not) are synthetically exposed in Table 2, concerning conchology, and in Table 3, concerning anatomy. Moreover, the phylogenetic analysis, exposed and discussed below, is also another ~~scenario of a~~ comparative approach. As explained above, its main concern is the comparison of the species studied herein with some others studied at the same level of anatomical details, rather than aiming to be the "phylogeny of the orthalicoideans."

This section, therefore, presents a formal taxonomic comparison of the genera and species studied here. However, it necessarily needs to be complemented by the above-mentioned texts and tables. [illegible]ording to the obtained results, no exclusive diagnostic character was obtained, neithe[illegible] the genus nor at the species levels. Actually, what is diagnostic is always a set of characters, and this set is reported in the respective diagnoses.

At the [illegible]us level, *Kora* exhibits much more uniformity of characters than *Neopetraeus*. Its shell char[illegible]ers are relatively monotonous, with almost monochromatic color, except for a small paler subsutural band, and sometimes a darker pre-apertural region. The general shape

is relatively similar, with some species slightly more elongated (e.g., *K. rupestris*), while others are more globose (e.g., *K. ajar*). *Neopetraeus*, on the other hand, displays a much wider range of features in its 15 species [8, 9], varying in color, shape, profile, and even sculpture. *Koltrora* is notable for its translucent, fragile shell in its so far single species. *Neopetraeus* occurs in the Andean region of South America, well separated from the other two genera, which occur in the mid-east region of Brazil, in the area of the São Francisco River. On the other hand, the three genera share interesting shell features that bring them closer and set them apart from other orthalicoideans, including the smooth protoconch with ~2 whorls, possessing some axial sculpture in the last half whorl in some species (some *Neopetraeus* are exceptions, with a reticulate protoconch – Fig. 54J); and a well-developed umbilicus resulting from a hollow tube zigzagging along the columella. This feature confused the initial identification of *Kora* samples, which were initially attributed to *Neopetraeus* [7], despite the geographic distance.

[illegible]espect to the anatomy, the three genera have the following more interesting dis[illegible]tions ~~and remarks~~: *Kora* is [illegible]gle in having a medial differentiated branch in secondary columellar muscles ~~in~~ both sides [illegible].., Fig. 18B), absent in the other known orthalicoideans; it also has a characteristic white pallial gland in mantle edge (e.g., Fig. 36A: gp); the radular sac bulging in posterior end of odontophore, covered by a transparent membrane (e.g., Fig. 3C: mr); the odontophore pair of muscles m[illegible]g., Fig. 4C); a very muscular basal region of bursa copulatrix (e.g., Fig. 6A: bu); a pair of longitudinal large folds along the inner penis surface (e.g., Fig. 38D: pf); and an especially elaborated spermatophore (e.g., Fig. 17D). *Koltrora* is the only known orthalicoidean that possesses two ducts to anterior lobe of digestive gland (Fig. 46C: dd). *Neopetraeus* is [illegible]orious in having both pairs of dorsal tensor muscles of radula (m4 and m5) as an indistinct [illegible]le mass (Fig. 56C). *Kora* and *Neopetraeus* share the accessory [illegible]umen chamber (e.g., Fig. 6B: as), which was not found in *Koltrora*. The ureter is totally [illegible]sed (tubular) in *Kora* and *Koltrora*, as apparently is the usual pattern of orthalicoideans, but *Neopetraeus* has the ureter opened in a larger degree (~50% – Fig. 56A); both genera also lost the pair of ventral tensor muscles of the radula (m11), which is present in *Neopetraeus* (Fig. 51C); they also share the calcified epiphragm (Figs 2J, 45D), which was not confirmed in *Neopetraeus*, neither in other orthalicoideans; both genera also have a thick muscular basal [illegible]ion in the penis wall, which is absent in *Neopetraeus*. ~~Other interesting argumentation is [illegible]sent in phylogenetic analysis below.~~

At the **species** level, all 11 *Kora* species have their shells naturally similar (Fig. 58), ~~only~~ small details can distinguish the species, which [illegible]e more important are exposed be[illegible] (Table 2). A relatively intraspecific uniformity, however, was observed, what is important in a [illegible]eatureless shelled genus. The species can be separated into size categories, those considered large, in the range of the 55 mm (*K. tupan, K. ajar*), those medium-sized, in the range of the 45 mm (*K. corallina, K. rupestris, K. jimenezi, K. uhlei, K. kremerorum*), and the small ones, in the range of the 30 mm (*K. nigra, K. aetheria, K. vania, K. curumim*). Related to the elongation degree, there is the more elongated, with tax length/width ~2.3 (*K. corallina, K. rupestris*), and those more globose, with tax ~1.6–1.7 (*K. nigra, K. ajar, K. curumim*), with remaining species with the tax ~1.9–2.0. Three species have a weak dorso-ventral shell compression: *K. tupan, K. aetheria* and *K. kremerorum*; the remaining species have a rounded transverse section. The superior implantation of the outer lip has a horizontal portion in *K. nigra, K. tupan, K. ajar, K. jimenezi, K. kremerorum,* and *K. vania*. The inner lip has usually a middle prominence in most species, almost making a fold, but some species this is reduced, as in *K. aetheria*, and weakly present in *K. nigra, K. kremerorum* and *K. curumim*. Related to the color, *K. corallina* and *K. rupestris* have their apical region light yellow to white, darkening in

middle level of spire; the remaining species have their apex with uniform color as remaining areas. The subsutural lighter band is not present only in *K. aetheria* and *K. curumim*. The pigmentation of the peristome is usually white, but *K. nigra, K. ajar* and *K. uhlei* commonly have brown pigment in it. The sculpture, beyond the usual axial narrow undulations, delicate spiral striae is also usually found, but they are absent in *K. aetheria, K. uhlei*, and *K. curumim*; and they are very scanty in *K. vania*. The umbilicus is usually widely opened, but *K. curumim* has it tightly occluded by inner lip; while *K. aetheria, K. vania* and *K. jimenezi* have the umbilicus narrowly opened.

Concerning anatomy, the differences between species become more pronounced. Table 3 presents synthesized information on the anatomical features of the species studied here. This discussion focuses only on those considered more interesting. The data presented in Table 3 is consistent across all examined specimens (Table 1), showing minimal intraspecific variation and providing a compelling basis for their use in taxonomy and phylogeny. Due to space limitations, the interpretation of Table 3 should be correlated with the list of abbreviations and, in some instances, with the information provided in the respective description. Notably, features such as folds in the mantle edge, the type of venation in the lung, and the organization of the kidney lobe serve as intriguing sources of taxonomic data, differing among the species. Similarly, variation was observed in the number of insertions of the secondary columellar muscles, with each species having its arrangement, and they are mostly asymmetrical. This observation extends to the buccal mass and odontophore muscles. As expected, the jugal muscles (m1l, m1v), and the m3 have proven useful in differentiating closely related species [29, 30]. On the other hand, the discovery of m2a in the present context is intriguing (Fig. 23F). This pair of muscles is exclusive to *K. ajar* and *K. jimenezi*, exhibiting an extraordinary convergence with *Anctus* (see below). Notably, this muscle pair, inserted into the m2 basal edge, contains a blood vessel (Fig. 30C). The jaw plate exhibits sets of different shapes; some have a central notch in the cutting edge (*K. corallina, K. rupestris, K. aetheria*), others are simply rectangular (*K. nigra, K. tupan, K. ajar, K. uhlei, Koltrora*), while *K. jimenezi* is narrow and arched. The localization of the salivary gland apertures also varies among the species, with some positioned in the middle and others more posterior. Additionally, *K. jimenezi* and *K. uhlei* have these apertures on papillae.

Concerning the odontophore, all species exhibit fused cartilages along the ventral edge, but the degree of fusion varies. *Koltrora* has approximately 100%, while *K. uhlei* has around 90%; *K. nigra* has the smallest fusion value of approximately 60%, and the remaining species are about 75% fused. The intrinsic muscles m4, m5, m7, and m10 also show distinctions among the species (Table 3), while the exclusive *Kora* pair m8 is relatively uniform, except in *K. jimenezi*, where it is relatively reduced. The function of this muscle m8 is enigmatic, as it only longitudinally covers the dorsal edge of the cartilages (e.g., Figs 4B, C).

Genital structures contain a wealth of comparative information. The carrefour region, for example, has a unique arrangement in each species (Figs 6B, 11C, 15A, 20E, 23E, 27E, 33E, 38A, 47C, 53D, 57B). This region, like many others (e.g., odontophore), has been neglected in descriptions and comparative studies, despite being highly informative. The carrefour (fertilization complex) form itself varies, as does its duct; its insertion can be in the duct of the albumen gland (ad) (K. *corallina, Neopetraeus*), between this duct and the albumen chamber (*K. nigra, K. tupan, K. uhlei*), at the tip of the spermoviduct (eo) (*K. rupestris, Koltrora*), directly in the albumen chamber (ac) (*K. ajar, K. jimenezi*), and between the duct of the albumen gland and spermoviduct (*K. aetheria*). The albumen chamber is a blind sac, in the usual fashion (e.g.,

Fig. 11C: ac), in *K. nigra, K. rupestris, K. tupan, K. aetheria*, and *Koltrora*; however, in the remaining species, *Neopetraeus* included, it has the strange form of a wide, bulged curve (e.g., Fig. 57B: ac). A second diverticulum-like projection, usually balloon-like, also filled with albumen and interpreted as an accessory albumen chamber (as), is a novelty in the presently studied species, being absent only in *Koltrora*. Another interesting feature is the presence of a thick muscular region in the basal portion of the duct of the bursa copulatrix (e.g., Fig. 6A: bu). It is present only in *Kora*, with the notable exception of *K. jimenezi*.

The characteristics of the penis also exhibit exclusivities for each species, showing weak intraspecific variation in adult forms. This renders the structure relatively reliable for species identification. The relative size (compared to the spermoviduct), the proportion of the epiphallus, and the internal (external during copula) arrangement of folds and structures are all important sources of comparative data (see Table 3). All *Kora* species (except for *K. jimenezi*) and *Koltrora* have the basal region of the penis with very muscular walls (e.g., Fig. 20D: mp). Additionally, all *Kora* species have the internal organization of the penis divided into regions, but a pair of main longitudinal folds is always present (e.g., Fig. 20D: pf). This is possibly linked to the formation of the long rod of the spermatophore, a characteristic feature of the genus (e.g., Fig. 17D). However, details of this pair of main folds (pf) and the arrangement of the remaining regions are exclusive to each species (Table 3). A very interesting feature of some *Kora* species is the transverse umbrella-like fold (e.g., Fig. 20: um). This enigmatic fold is only found in *K. rupestris*, *K. tupan, K. ajar, K. aetheria*, and *K. uhlei*. Each of these species also has a different number of rods sustaining the thin fold (Table 3). As no *Kora* mating behavior has been observed so far, the function and even the appearance of this umbrella-like fold during copulation can only be imagined. Another interesting distinctive feature is the insertion of the penis muscle (pm); in most species, it is at the tip of the epiphallus (terminal) (e.g., Fig. 20A). However, in *K. rupestris, K. ajar*, and *K. uhlei*, the insertion is at the base of the epiphallus, with additional insertions along the lateral wall of the epiphallus (e.g., Fig. 23B: pm). The insertion is subterminal in *Koltrora* (Fig. 48C).

Geographic analysis

As can be seen in Fig. 60, all Brazilian species studied in the present paper were collected in areas surrounding the São Francisco River, as it crosses through the states of Minas Gerais and Bahia. This region, primarily north of Minas Gerais up to South Bahia, is characterized by calcareous soil, abundant caves, and a remarkable richness of malacofauna. As depicted on the map (Fig. 60), species of *Kora* exhibit a high degree of endemicity, often separated by distances of less than 100 km. They are found on both sides of the river, with an equal prevalence of species on each side. The only species with a broader distribution is *K. rupestris* (indicated by red dots), occurring in an area of approximately 300 km and present on both sides of the river. All species also inhabit regions near the river; the farthest species is *K. curumim*, located approximately 250 km from the river.

Other pulmonates, such as *Habeas* [4, 5], and northern species of *Anthinus* [6], exhibit similar geographical patterns to those observed in *Kora*. The intricacies of the Brazilian region, both in biological and geological terms, are still under investigation—a project to which this paper contributes. It is anticipated that a more comprehensive explanation for the distribution patterns will emerge in the near future. Notably, *K. rupestris* stands out as an exception, in which anthropic influence is not excluded.

Phylogenetic analysis

As informed above, a phylogenetic analysis was performed with the single objective of comparing the species studied herein in a more orthodox scenario, as well as to take advantage of already available data in the literature on other orthalicoideans to analyze the taxonomic/phylogenetic position of them in a wider context. Also, a secondary objective is to compare the present result based on a morphologic-based dataset with others based on molecular ones.

The matrix of characters (Appendix 2), which includes ingroup and outgroups, was built based on the 94 characters (251 states) listed in the Appendix 1. The processing of this dataset as reported above resulted in a single cladogram (Fig. 61), which is analyzed herein. Its statistics are length: 391; consistency index: 54, retention index: 76. For the obtention of a single cladogram, the method by [31] was applied.

The three genera studied in this paper resulted in a monophyletic branch (node 10), supported by 10 synapomorphies. The more interesting are shell with slightly dislocated peristome (character 9) and strong umbilicus going further along columella (ch. 11, 13), higher fusion of odontophore cartilages (ch. 52), the accessory albumen chamber (ch. 70), and the elongation of the epiphallus (ch. 89).

Both *Neopetraeus* species resulted as first branch (node 11), also supported by 10 synapomorphies. The more remarkable are: the ureter ~50% opened (as a groove) (character 25), which, in the present context, resulted as a reversion; the odontophore m4 and 5 pairs as a single bundle (ch. 44); the insertion of the carrefour in the duct of albumen gland (ch. 68); the prostate fulfilling only ~20% of the spermoviduct (ch. 71); and the oviduct lacking spermoduct folds (ch. 72).

The other branch (node 12) gathers *Koltrora* and a monophyletic *Kora*. The branch is supported by 11 synapomorphies. The more interesting are the shell monochromatic color (character 1); the calcified epiphragm (ch. 16); the folds of the mantle edge (ch. 22); the intercalated pair of vessels ate left from pulmonary vein in lung (ch. 23); the similarities in the radula (ch. 31, 32, 33, 35); the loss of the odontophore pair m11 (ch. 50); the jaw and peribuccal muscles (mj) forming a ventral platform (ch. 51); and the pair of longitudinal folds at least in the basal region of the penis (ch. 84). Interestingly, *Koltrora pyrostoma* has nothing less than 13 homoplastic autapomorphies, being 4 of them reversions in the present context; two of them are noteworthy: the loss of the accessory albumen chamber (ch. 70) and of the penis shield (ch. 80). This set of autapomorphies, and the lack of the *Kora* synapomorphies, are sufficient reasons for the generic separations of both taxa, despite they share more characters than *Neopetraeus*. This genus is geographically more distant, but it has the overall shape more similar to *Kora* than *Koltrora* does. But some details of the shell (e.g., the smooth protoconch) and of the anatomy showed that *Koltrora* is closer to *Kora*, but sufficiently different to deserve its own genus. The nearly identical radula between *Koltrora* and *Kora*, in contrast to *Neopetraeus* which exhibits the typical radula patterns of most orthalicoideans (Figs. 52, 55G–I), appears to be the primary factor influencing the phylogenetic proximity between both genera. Despite *Kora* and *Neopetraeus* sharing an overall shell shape and the auxiliary/secondary albumen chamber, the loss of this latter character is attributed to *Koltrora* in this study.

The *Kora* branch (node 13) has the extraordinary support of 25 synapomorphies. The more interesting are: the shell sculpture of axial undulations and spiral micro-stripes (characters 6, 7); the axial cords in end of protoconch (ch. 15); the medial detached branch of secondary

columellar muscles (ch. 18); the robustness of the jaw folds (ch. 29); the radular sac widely bulging posteriorly in buccal mass covered by special membrane (ch. 36, 37); the loss of the odontophore horizontal muscle (m6) (ch. 45) and the presence of the pair m8 (ch. 47); the duct to anterior lobe of the digestive gland inserted in stomach, having only right branches (ch. 57, 59); the digitiform seminal receptacle (ch. 62); a confluence of inner folds originating the vas deferens in spermoviduct (ch. 74); the filiform penis internally divided into compartments (ch. 78, 83); and the short cerebral commissure (ch. 94).

Kora jimenezi, with its 14 homoplastic autapomorphies, is the first *Kora* branch. It is separated from the other congeners (**node 14**) by 9 synapomorphies. The noteworthiest are: 7–8 insertions of the left, and 6–7 of the right secondary columellar muscles (characters 19, 20); the pair of buccal mass dorso-ventral m3 (ch. 42); the odontophore pair m7 as a single, narrow bundle (ch. 46); the strongly muscular basal region of the walls of the bursa copulatrix duct (ch. 75), and of the penis (ch. 82). The node 14 is divided into *K. rupestris* and **node 15**, which is supported by 4 synapomorphies: pulmonary vein anteriorly branched (ch. 24); the carrefour insertion in duct of albumen gland (ch. 68); the imbricated pair of penial longitudinal folds (ch. 85); and converging folds from vas deferens coming from epiphallus (ch. 91). The node 15 is a dichotomy of 2 branches: nodes 16 and 17. **Node 16** gathers *K. corallina* and *K aetheria*, and is supported by 4 synapomorphies: shell umbilicus widely opened (ch. 14); prostate occupying ~35% of the spermoviduct (ch. 71); penis ~75% of spermoviduct length (ch. 79); and epiphallus ~35% of penis length (ch. 89). **Node 17** has the remaining *Kora*, being supported by 6 synapomorphies, such as jaw plate rectangular (ch. 28); odontophore pair m5 as continuation of the pair m4 (ch. 44); a bulger projection in the carrefour (ch. 67); insertion of carrefour between albumen gland duct and accessory albumen chamber (ch. 68); and vas deferens inserted in penis strongly curved (ch. 77). This node is divided into *K. uhlei* and **node 18**, which is supported by 6 synapomorphies, such as shell peristome with outer lip superior insertion rather horizontal (ch. 10); mantle edge with pointed secondary folds (ch. 22); penis 85–90% of spermoviduct length (ch. 79); large longitudinal fold in epiphallus (ch. 90). Node 18 includes *K. tupan* and **node 19**, supported by 5 synapomorphies, like loss of the pair m1v (ch. 38); the odontophore pair m5 covering the pair m4 (ch. 44); and the loss of the bulges portion of the carrefour (ch. 67). It is remarkable to observe that all *Kora* species have their considerable set of autapomorphies. Almost all of them are homoplastic, i.e., reversions or convergences with other branches. These set of characters double-reinforce the species distinctions, some of them are included in their diagnoses.

Going more amply, it is interesting to check that what up to recently was Bulimulidae (**node 3**), resulted monophyletic, supported by 9 synapomorphies. The more interesting are a narrow umbilicus in the shell covered by inner lip (character 12); the pair of secondary columellar muscles (ch. 17); the similarity between the radular rachidian, lateral and marginal teeth (ch. 30, 31, 32); the odontophore pair m5 covering the pair m4, inserted in it instead of in the cartilage (ch. 44); the digitiform-cylindric form of seminal receptacle (ch. 62); and the penis shield restricted to the penis base (ch. 80). More recently, based on molecular studies, what was only Bulimulidae now is a set of several families. The present assembly, for example, there is Simpulopsidae (*Rhinus*), Megaspiridae (*Kora*), and truly Bulimulidae (*Bulimulus, Anctus, Sanniostracus, Drymaeus, Neopetraeus*) [8, 17]. The presently shown arrangement does not agree with this recent taxonomy, as will be discussed below. All these genera are gathered in the superfamily Orthalicoidea.

Node 3 is divided into *Bulimulus sula* and **node 4**, supported by 9 synapomorfies, such as colorful shell (character 1); 3–4 insertions of the left secondary columellar muscle (ch. 19); posterior location of the salivary aperture in oral cavity (ch. 54); the conic form of the carrefour (ch. 65); insertion of penis muscle at tip of epiphallus (ch. 81); epiphallus ~10–15% of penis length (ch. 89); and nerve ring with pleural ganglia close to pedal pair, and bearing pleural commissure (ch. 92, 93).

Node 4 is a dichotomy, one of its branches is the node 10 already discussed above. The other is the **node 5**, which is supported by 6 synapomorphies. Those more remarkable are: the buccal mass pair of muscles m3 close to esophagus (ch. 42); the stomach as a simple curve, with anterior duct inserted in it (ch. 56, 57); the slightly filiform penis (ch. 78); and the inner penial organization with a special mosaic of low folds (ch. 83). Node 5 gathers *Anctus angiostomus* plus **node 6**, supported by 4 synapomorphies: reticulated protoconch (ch. 15); left secondary columellar muscle with 2 insertions (ch. 19); multicuspid radular marginal teeth (ch. 34); and epiphallus with large longitudinal inner fold (ch. 90). This node, in turn, is divided into *Rhinus botocudus* and **node 7**, having 3 synapomorphies, such as odontophore pair m7 as 2 separated muscles (ch. 46); and uterus wall thick glandular (ch. 73). Node 7 has *Sanniostracus carnavalescus* as a branch, and **node 8** as the other, representing the genus *Drymaeus*, supported by 10 synapomorphies. The more notable are: ureter opened ~15% inside pulmonary cavity (ch. 25); buccal mass pair m2 as a single bundle (ch. 40); loss of m3 and m11 (ch. 42, 50); odontophore cartilages ~50% fused (ch. 52); pair of salivary glands forming a single mass (ch. 53); prostate occupying ~35% of the spermoviduct (ch. 71); penis shield with ~1/3 of the penis length (ch. 80); and a short cerebral commissure (ch. 94). The 3 species of *Drymaeus* (node 8) have an internal arrangement, with *D. castilhensis* closer to *D. micropyrus*, in the **node 9**, supported by a single synapomorphy (insertion of the carrefour in the duct of the albumen gland – ch. 68), separating them from *D. currais*.

Going further ampler, two more remote outgroups resulted, as expected, outside the orthalicoideans, in the **node 2**. This node gathers together the Solaropsidae *Olympus nimbus* (superfamily Sagdoidea) [8], and the Achatinidae/Subulininae *Lavajatus moroi* (Achatinoidea). The node is supported by 3 synapomorphies: Insertion of hermaphrodite duct in the tip of seminal receptacle (character 64); the lack of the albumen chamber (ch. 69); and the bursa copulatrix shorter than 25% of the spermoviduct (ch. 76). Of course, it is important to keep in mind that, as these taxa are not the focus of the present paper, the survey of characteristics is not as detailed as in the orthalicoideans. Anyway, both being reunited in a branch (node 2) is a surprise. This arrangement serves at least as first step of an investigation to define Orthalicoidea phylogenetically based on phenotypic features, what is still missing.

The genus *Olympus* Simone, 2010, has been considered synonym with *Solaropsis* Beck, 1837 [8]. It is appropriate to clarify a taxonomic misconception in this case, which is based on a molecular approach [32]. In the referenced paper, *Olympus* is placed in a final polytomy in the main cladogram of that paper (see [32] fig. 1), mixed with species of *Solaropsis* and *Psadara* Miller, 1878. At the base of this polytomy, two species of Solaropsis precede it. The authors, based on this result, synonymized all genera, rendering Solaropsidae monogeneric. However, this overlooks considerable phenotypic differences between *Solaropsis* and, at least, *Psadara* and *Olympus* [11, 18]. The premature consideration of all these genera as synonyms resulted from the low resolution of the cladogram, the non-inclusion of the type species of *Psadara* and *Solaropsis*, and a disregard for the important set of morphologic differences among the three genera [18]. The synonymy can be easily challenged after a total resolution of the polytomies,

with the inclusion of the type species, and a supraspecific taxonomic rearrangement of the species. Therefore, for taxonomic stability, a more conservative approach should be taken in maintaining the validity of all three genera until a better resolution is achieved, along with a deeper analysis of the generic differences, which is still lacking. Possibly, after a better resolution, more species can be added to *Olympus*, instead of being an invalid genus belonging to a large monogeneric family. Confirming that, a recently described species, *Solaropsis penthesileae* Salvador, 2021 [33] has the shell very similar to *O. nimbus*, and the molecular analysis also resulted both species as sister taxa [33: fig. 1]. Certainly *S. penthesileae* is an *Olympus*. Applying these justifications, *Olympus* is maintained valid in this paper.

Brief comparison with molecular approaches

While the primary focus of this study pertains to the current assembly of studied taxa, the broader range of included taxa in the phenotypic phylogenetic analysis allows for some inferences and comparisons with molecular studies. Molecular approaches have become much more prevalent in recent times, nearly rendering other methodologies obsolete. Interestingly, these approaches seem to evolve without apparent concern for anchoring the resulting taxa with recognizable morphological attributes. There is little regard for prior arrangements, whether based on morphological or molecular datasets, as the results of each published study rarely align fully with those of previous ones.

For instance and example, within the orthalicoideans, the positioning of *Drymaeus* in relation to *Bulimulus* and *Bostryx* is subject to variation. In some studies, *Drymaeus* is found to be closer to *Bulimulus* than *Bostryx* [34]; in others, *Bostryx* is closer to *Bulimulus* than *Drymaeus* [35]. At times, *Drymaeus* is identified as the sister group to a combined cluster of *Bostryx* and *Bulimulus* species [17]. With such fluidity, maintaining taxonomic stability becomes challenging, and it poses a significant obstacle to achieving mental coherence in the classification trustworthiness.

A recent study [17] marks a significant milestone in the field of orthalicoideans, both in terms of the extensive range of studied taxa and the profound taxonomic changes it introduces. That paper provides a comprehensive history and analysis of previous molecular studies, many of which were conducted by the authors themselves. Among the species examined in that study is *Kora rupestris*, which emerged as a monophyletic branch with *Thaumastus* Martens, 1860 positioned at the base of a larger branch containing other orthalicoideans. The authors connected this branch with a preceding one that encompasses *Megaspira* Lea, 1836, and coined the term 'Megaspiridae' to describe this recognized paraphyletic group. Interestingly, 'Megaspiridae' appears to be a notably heterogeneous taxon, as the three genera share little beyond being snails. *Megaspira* was previously associated with Achatinidae/Subulininae due to its turriform shell until a recent reevaluation, with its transfer to Orthalicoidea being solely based on molecular evidence [34], as its anatomy remains unknown.

Neopetraeus tessellatus is also featured in the aforementioned study [17: fig 6]. It emerged as the first branch of *Drymaeus*, alongside *D. expansus*. In that study, the authors proposed placing both genera, along with three others, in the new subfamily Peltellinae within Bulimulidae. Therefore, the proximity observed between *Kora* and *Neopetraeus* in this analysis, supported by 10 synapomorphies (Fig. 61: node 10), is not corroborated by the findings in that particular paper. In this study, the representative of *Bulimulus* was identified as the first branch in the orthalicoidean lineage (node 3), while *Drymaeus* emerged as the last

branch (node 8). In contrast, [17: fig. 6] depicts a very different scenario, wherein both genera are practically sister taxa in an almost terminal branch.

In the present paper, between *Bulimulus* and *Drymaeus*, the genera *Anctus, Rhinus* and *Sanniostracus* are successively allocated (Fig. 61: nodes 5–7). In that paper [17], *Bulimulus, Sanniostracus, Drymaeus* and *Anctus* are Bulimulidae, while *Rhinus* is Simpulopsidae. That arrangement is not compatible with the present one, as a simpulopsid is allocated between bulimulids, in a paraphyletic arrangement.

The discrepancies observed in phenotypic and molecular arrangements, as well as among different molecular studies, could potentially be attributed to the limited morphological framework in Mollusca when compared to other zoological groups such as vertebrates and arthropods [31]. This highlights the urgent need to promote research in comparative morphology, parallel to the progress seen in molecular studies.

Main evolutive processes

Orthalicoideans and strophocheiloideans constitute the most speciose groups among South American land snails [12]. However, they may not be closely related, as they are separated at the infraorder level [8], with the former belonging to Orthalicoidei and the latter to Rhytidoidei. According to a previous study [6], the phylogeny revealed a clear trend among strophocheilids to increase in body size, consume a wide range of vegetal matter, and reproduce through a strategy of few and large eggs, interpreted as adaptations of the digestive and genital systems.

In contrast, Orthalicoideans maintain a plesiomorphic pattern in terms of size and egg-laying, but different genera and species exhibit significant meal specializations. They are generally challenging to maintain in captivity and display a wide range of digestive conformations, particularly in buccal structures and radula. Nevertheless, node 3 (Fig. 61) presents, for the first time, a set of synapomorphies for Orthalicoidea. While this is preliminary, given that several important subgroups are still missing, it serves as an initial step and provides a framework for future studies at a similar level of detail.

The first orthalicoidean branch is *Bulimulus*, characterized by a fragile, featureless shell. This contrasts with its sister branch (node 4), where shells are predominantly colorful and thick-walled. Additionally, there is an increment in the epiphallus, with the penis muscle connected to it rather than being inserted into the penis body, along with the appearance of a commissure of the pair of pleural ganglia.

This branch (node 4) exhibits a dichotomy, giving rise to nodes 5 and 10. Node 10 encompasses all species focused on in this paper, predominantly characterized by large, thick-walled shells, a robust peristome, and a notable umbilicus that forms the tip of a hollow fold running along the columella. Notably, the internal structure reveals the presence of an accessory albumen chamber, although the mechanism of egg laying in these animals remains elusive.

On the other branch (node 5), a mix of bulimulids and simpulopsid is observed. This branch demonstrates a trend towards an increase in midgut size, with specializations in the gastric region, and a simplistically organized yet highly elongated penis.

Despite being preliminary, this marks the first attempt to construct an orthalicoid phylogeny based solely on phenotypic features—a crucial initial step toward a broader

understanding. This framework will serve as the foundation for ongoing studies encompassing other branches within this superfamily. Remarkably, this paper introduces newly discovered structures, including the odontophore pair of muscles (m8), resembling cartilage tensors, so far exclusive to *Kora*. The accessory albumen chamber, shared by *Kora* and *Neopetraeus*, is noteworthy, with a similar feature found in *Sanniostracus carnavalescus* [15]. Additionally, a distinct model of spermatophore (e.g., Fig. 8F, G) with a multifolded tubular basal rod has been identified in certain *Kora* species. The calcified epiphragm, commonly found in non-South American snails (e.g., helicinids), is practically absent on this mainland, except for instances in *Kora* and *Koltrora*. The unusual radula conformation observed in these two genera further contributes to the morphologic and taxonomic novelties presented. These examples underscore the gaps in our knowledge of South American land snails, emphasizing the need for further comprehensive studies.

Acknowledgements

I am especially grateful to the people that sponsored the collect expedition and donated the samples to MZSP, particularly José Coltro Jr, Mauricio Uhle, Weslley Vailant-Mattos, David Jimenez. ~~To~~ Frederico Gutierrez ~~by~~ the kind donation of both Peruvian *Neopetraeus* samples studied herein. To Andrea Salvador, NHMUK, for permission to publish the photo of *N. lobbi* lectotype. For the SEM examination, I thank to Lara Guimarães, Natan Carvalho Pedro, and Fernanda Santos Silva (MZSP).

Author Contributions

Conceptualization: Luiz Ricardo L. Simone.
Data curation: Luiz Ricardo L. Simone.
Formal analysis: Luiz Ricardo L. Simone.
Investigation: Luiz Ricardo L. Simone.
Methodology: Luiz Ricardo L. Simone.
Project administration: Luiz Ricardo L. Simone.
Resources: Luiz Ricardo L. Simone.
Software: Luiz Ricardo L. Simone.
Supervision: Luiz Ricardo L. Simone.
Validation: Luiz Ricardo L. Simone.
Visualization: Luiz Ricardo L. Simone.
Writing – original draft: Luiz Ricardo L. Simone.
Writing – review & editing: Luiz Ricardo L. Simone.

References

1. Simone LRL. Taxonomical study on a sample of pulmonates from Santa Maria da Vitória, Bahia, Brazil, with description of a new genus and four new species (Mollusca: Orthalicidae and Megalobulimidae). Pap Avul Zool. 2012; 52(36): 431–439.
2. Simone LRL. Three new species of *Kora* (Pulmonata, Orthalicidae) from Bahia and Minas Gerais, Brazil. J Conch. 2015; 42(1): 51–56.
3. Salvador RB, Simone LRL. A new species of *Kora* from Bahia, Brazil (Gastropoda: Pulmonata: Orthalicoidea), with an emended diagnosis of the genus. Stuttg. Beitr. Naturk. A, n.s. 2016; 9: 1–7. DOI: 10.18476/sbna.v9.a1

4. Simone LRL, Review of the genus *Habeas* from Brazil, with description of four new species and in which anatomical features revealed that they belong to Urocoptidae (Eupulmonata, Stylommatophora). Malacologia, 2022; 64(2): 269–286.
5. Simone LRL. A new *Habeas* (Eupulmonata, Urocoptidae) from Canarana region, Bahia, Brazil. Pap. Avuls. Zool. 2022; 62: e202262060. Doi: 10.11606/1807-0205/2022.62.060
6. Simone LRL. Additions to the genus *Anthinus* occurring in Minas Gerais and Goiás regions, Brazil, with description of five new species, one of the in the new related genus *Catracca* (Gastropoda, Eupulmonata, Strophocheilidae). PlosOne 17(8): e0273067. Doi: 10.1371/journal.pone.0273067
7. Salgado N; Coelho ACS. Ocorrência de espécie de *Neopetraeus* Martens, 1885 no Brasil. IN: Encontro Brasileiro de Malacologia Pesquisas e Resumos: XVIII EBRAM. Universidade do Estado do Rio de Janeiro. Rio de Janeiro, pg. 134; 2003.
8. MolluscaBase eds. MolluscaBase. *Neopetraeus* Martens, 1885. Accessed at: https://www.molluscabase.org/aphia.php?p=taxdetails&id=995860 on 2023-06-13.
9. Pilsbry H. Manual of Conchology. Second series, 1901–1902; 14: 1–302.
10. Simone LRL. Phylogeny of the Caenogastropoda (Mollusca), based on comparative morphology. Arq. Zool., 2011; 42: 161–323.
11. Simone LRL. Land and freshwater Molluscs of Brazil. São Paulo, EGB/FAPESP; 2006.
12. Simone LRL. Bosquejos de Filogenia. São Paulo. Clube de Autores; 2020. https://clubedeautores.com.br/livro/bosquejos-de-filogenia
13. Simone LRL; Amaral VS. Insular life: new endemic species from São Paulo oceanic islands, Brazil (Pulmonata, Bulimulidae), as example of endemicity. J. Conch. 2018; 43(2): 167–187.
14. Simone LRL; Belz CE; Gernet MV. A new species of *Drymaeus* endemic from Currais Archipelago, Paraná, Brazil (Pulmonata, Bulimulidae). Pap Avul Zool. 2020; 60: e20206057. DOI 10.11606/1807-0205/2020.60.57
15. Simone LRL; Salvador RB. Taxonomical study on a sample of land snails from Nanuque (Minas Gerais, Brazil), with descriptions of three new species. Stuttgarter Beit. Z. Naturk. A, n. s. 2016; 9: 9–30.
16. Simone LRL. Anatomical description of *Anctus angiostomus* (Wagner, 1827) from Northeastrn Bahia, Brazil (Gastropoda, Pulmonata, Bulimulidae. Stud. Neotrop. Fauna & Environm., 1998; 33: 170–177.
17. Salvador RB; Silva FS; Cavallari DC; Köhler F; Slapcinsky J; Breure ASH. Molecular phylogeny of the Orthalicoidea land snails: Further support and surprises. Plos One, 2023, 18(7): e0288533. https://doi.org/10.1371/journal.pone.0288533.
18. Simone LRL. A new genus and species of camaenid from the Amazon rainforest, Brazil (Pulmonata, Helicoidea). J Conch, 2010, 40(2): 149–161.
19. Simone LRL. *Lavajatus moroi*, new cavernicolous Subulininae from Ceará, Brazil. Spixiana, 2018, 41(2): 173–187.
20. Cavallari DC; Salvador RB; Simone LRL. A possible land snail diversity hotspot in Bahia state, Brazil. Tentacle, 2016; 24: 14–16. Era 17
21. Salvador RB; Simone LRL. New records of Orthalicoidea land snail species for the state of Minas Gerais, southeastern Brazil (Gastropoda, Stylommatophora). Check List, 2021; 17(2): 395–399.
22. Salvador RB; Silva FS; Cavallari DC; Cunha CM; Bichuette ME. Cave-dwelling gastropods (Mollusca: Gastropoda) of Brazil: state of the art and conservation. Zoologia, 2022; 39: e21033.
23. Simone LRL. The molluscan jugal muscles – m1. Malacopedia, 2022; 5(5): 43–48. http://www.moluscos.org/trabalhos/Malacopedia/05-05Simone%202022%20Malacopedia-jugal%20muscle%20m1.pdf

24. Reeve L. Monograph of the genus *Bulimus*. Conchologia Iconica, 1849; 5: pl 1–89.
25. Pilsbry HA. American Bulimulidae. Manual Conch., 1897–1898; 11: 1–339, 51 pls.
26. Breure ASH; Araujo R. The Neotropical land snails (Mollusca, Gastropoda) collected by the 'Comisión Científica del Pacífico'. PeerJ, 2017; 5: e3065; DOI 10.7717/peerj.3065
27. Shuttleworth RJ. Diagnosen neuer Mollusken. Mittheilungen Naturf. Gesell. in Bern. 1852; 248–249: 193–208.
28. Breure ASH. Notes on and descriptions of Bulimulidae (Mollusca, Gastropoda). Zool. Verhandelingen, 1978; 164: 3–255, pls. 1–22.
29. Simone LRL. The molluscan jugal muscles – m1. Malacopedia, 2022; 5(5): 43–48. http://www.moluscos.org/trabalhos/Malacopedia/05-05Simone%202022%20Malacopedia-jugal%20muscle%20m1.pdf
30. Simone LRL. The unnamed odontophore muscles m3 and m9. Malacopedia, 2023; 6(5): 29–34. http://www.moluscos.org/trabalhos/Malacopedia/06-052023%20Malacopedia%20m3-m9.pdf
31. Simone LRL. Reflections: A biological method for diminishing the number of trees in phylogenetic studies. Malacopedia, 2023; 6(4): 25–28. http://www.moluscos.org/trabalhos/Malacopedia/06-042023%20Malacopedia%20less%20trees.pdf
32. Calcutt J; Cuezzo MG; Jackson M; Salvador RB. Phylogenetic relationships and classification of Solaropsidae (Gastropoda: Stylommatophora). Arch. Moll. 2020; 149(2): 181–193.
33. Salvador RB. A new species of *Solaropsis* Back, 1837 (Gastropoda: Stylommatophora: Solaropsidae) from the Brazilian Amazon. Folia Malacologica 2021; 29(3): 178–185. https://doi.org/10.12657/folmal.029.021
34. Breure ASH, Romero PE. Support and surprises: molecular phylogeny of the land snail superfamily Orthalicoidea using a three-locus gene analysis with a divergence time analysis and ancestral area reconstruction (Gastropoda: Stylommatophora). Arch. Mollusk., 2012; 141(1): 1–20.
35. Breure ASH, Groenenberg DSJ, Schilthuizen M. New insights in the phylogenetic relations within the Orthalicoidea (Mollusca: Gastropoda), based on 28S sequence data. Basteria 2010; 74: 25–31.

Legends

Table 1. Summary of number of specimens. (In Material and methods)

Table 2. Synoptic table of main conchological differences among studied species.

sp| charact	shell size range mm	longer than wide	dorso-ventral flattened	aperture % length	aperture % width	horizontal end in outer lip	middle fold in inner lip
Kora corallina	45	2.3	no	44	70	no	yes
Kora nigra	30	1.6	no	48	60	yes	yes
Kora rupestris	45	2.3	no	50	66	no	yes
Kora tupan	55	1.9	yes	54	70	yes	yes
Kora ajar	55	1.7	no	54	71	yes	yes
Kora aetheria	30	2.0	yes	45	60	no	no
Kora jimenezi	45	2.0	no	45	70	yes	yes
Kora uhlei	45	2.0	no	50	65	no	yes

Kora kremerorum	45	1.9	yes	49	70	yes	yes
Kora vania	30	1.9	no	46	65	yes	yes
Kora curumim	30	1.7	no	50	65	no	no
Koltrora pyrostoma	30	1.8	yes	50	57	no	no
Neopetraeus lobbii	55	2.2	no	45	60	no	no
Neopetraeus tesselatus	45	1.5	no	57	65	no	no

Table 3. Synoptic table of main anatomical differences among studied species.

sp| charact	mb folds	venation L from cv	anterior end of cv	strong venation L of cv	kidney lobe	insertions cl	insertions cr
Kora corallina	low	pair intercal	branched	near PN	several entire	13	7
Kora nigra	narrow pointed	pair post	simple	up to 1/2 py	single broad surround	7	7
Kora rupestris	narrow pointed	pair intercal	simple	almost absent	4 anterior	7	7
Kora tupan	wide point	pair intercal	branched	up to 1/2 py	single broad surround	8	6
Kora ajar	wide point	pair intercal	branched	up to 1/2 py	several entire	5	4
Kora aetheria	wide projetct	pair intercal +Y	branched	up to 1/2 py	several entire	5	7
Kora jimenezi	wide round	no	simple	near PN	several entire	3	3
Kora uhlei	wide round	pair intercal	branched	up to 1/2 py	several entire	3	3
Koltrora pyrostoma	wide round	pair intercal	simple	up to 1/2 py	several entire	4	2
Neopetraeus lobbii	no	no	branched	up to 1/2 py	4 anterior	3	4
Neopetraeus tesselatus	no	no	branched	near PN	5 anterior	3	4

sp| charact	medial branch differentiated	m1l	m1v pairs	m3	m2a	dp post duct dg	dd ant duct
Kora corallina	no	no	2	pair esoph	no	R branches	both branch
Kora nigra	yes	no	2	pair esoph	no	Y-shaped	R branch

Kora rupestris	yes	yes	2	pair m2	no	R branches	both branch
Kora tupan	yes	yes	0	pair esoph	no	R branches	R branch
Kora ajar	yes, asymm	no	0	pair esoph	yes	R branches	R branch
Kora aetheria	yes	yes	1	pair esoph	no	R branches	both branch
Kora jimenezi	no	yes	0	0	yes	R branches	R branch
Kora uhlei	yes, post	yes	0	pair esoph	no	R branches	R branch
Koltrora pyrostoma	no	yes	0	0	no	single	double*
Neopetraeus lobbii	no	yes	1	0	no	single	R branch
Neopetraeus tesselatus	no	yes	1	2 pairs lomgit	no	single	R branch

sp| charact	jaw	sa localiz	sa form	oc fusion %	m4-m5	m7	m8
Kora corallina	central notch	middle 1/3	0	75	m4 covering m5	narrow, single origin	wide
Kora nigra	rectangular	middle 1/3	0	60	m4 covering m5	narrow, single origin	wide
Kora rupestris	central notch	middle 1/3	0	75	m4 covering m5	narrow, single origin	wide
Kora tupan	rectangular	post 1/3	0	75	m4 as continuation of m5	3 bundles	wide
Kora ajar	rectangular	middle 1/3	0	75	m4 covering m5	narrow, single origin	wide
Kora aetheria	central notch	middle 1/3	0	75	m4 covering m6	narrow, single origin	wide
Kora jimenezi	arched	post 1/3	papilla	75	m5 covering m4	broad, thick	narrow
Kora uhlei	rectangular	middle 1/3	papilla	90	m4 as continuation of m5	filiform	wide
Koltrora pyrostoma	rectangular	post 1/3	0	100	m5 covering m4	2 separated stripes	0
Neopetraeus lobbii	central notch	post 1/3	zigzag fold	50	single mass	0	0

Neopetraeus tesselatus	central notch	post 1/3	0	70	single mass	0	0

sp| charact	m10	m11	hd curved at end	ca-carrefour	ca-duct	ca bulged portion	ca insertion
Kora corallina	narrow	absent	yes	conic	wide-short	0	in ad
Kora nigra	narrow	absent	no	elongated	narrow-long	0	betw ad-ac
Kora rupestris	narrow	absent	yes	entire narrow	narrow-long	0	tip eo
Kora tupan	broad	absent	yes	conic	narrow-long	yes-narrow	betw ad-ac
Kora ajar	narrow	absent	yes	conic	narrow-short	0	in ac
Kora aetheria	broad	absent	yes	conic	narrow-long	0	betw ad-eo
Kora jimenezi	filiform	absent	yes	conic	narrow-short	yes-wide	in ac
Kora uhlei	narrow	absent	yes	conic	narrow-long	yes-narrow	betw ad-ac
Koltrora pyrostoma	broad	absent	no	entire narrow	narrow-long	0	tip eo
Neopetraeus lobbii	broad	present	yes	conic	narrow-long	yes-small	in ad
Neopetraeus tesselatus	broad	present	no	conic	narrow-long	0	in ad

sp| charact	ac-alb chamb	as - accessory alb chamb	numb of sp	% of pt in eo	bu- musc bursa duct	% pe of eo	% bd of eo
Kora corallina	curve	present	1	35	yes	70	90
Kora nigra	sac	present	2	35	yes	100	90
Kora rupestris	sac	present	1	50	yes	60	100
Kora tupan	sac	present	1	45	yes	85	100
Kora ajar	curve	present	1	45	yes	90	90
Kora aetheria	sac	present	1	35	yes	65	80
Kora jimenezi	curve	present	1	50	no	100	80
Kora uhlei	curve	present	1	45	yes	60	80
Koltrora pyrostoma	sac	0	1	25	no	50	70
Neopetraeus lobbii	curve	present	2	20	no	50	70
Neopetraeus tesselatus	curve	present	2	20	no	90	90

sp| charact	vd terminal curve	mp basal musc wall	pf pair longit	um umbrella fold	um # rods	% eh of pe	ei-epiph fold	pm insertion
Kora corallina	0	yes	yes-imbricated	0	0	13	yes	terminal
Kora nigra	yes	yes	yes-simple	0	0	25	0	terminal
Kora rupestris	0	yes	yes-simple	yes	5	15	0	base
Kora tupan	yes	yes	yes-wings	yes	3	20	yes	terminal
Kora ajar	yes	yes	yes-simple	yes	5	25	yes	base
Kora aetheria	0	yes	yes-imbricated	yes	6	33	0	terminal
Kora jimenezi	0	0	yes-simple	0	0	25	0	terminal
Kora uhlei	yes	yes	yes-imbricated	yes	3	25	0	base
Koltrora pyrostoma	0	yes	yes-fused	0	0	25	0	subterminal
Neopetraeus lobbii	0	0	0	0	0	33	yes	terminal
Neopetraeus tesselatus	0	0	0	0	0	20	yes	terminal

Legends

Fig. 1. *Kora corallina* shell characters. (A–B) holotype MZSP 103910 (L 43.4 mm), frontal and dorsal views. (C–E) dissected specimen MZSP 132078, frontal, right and dorsal views, epiphragm preserved, hole artificially done for specimen extraction (L 44.8 mm). (F) MZSP 151952#1, shell slightly deformed (L 35.5 mm). (G) same, dorsal view. (H) same, detail of apex, profile-slightly apical view, arrow showing transition protoconch-teleoconch, scale= 2 mm. (I) same, detail of last whorl, left-slightly anterior view showing umbilicus, scale= 2 mm. (J–K) MZSP 151952#2, frontal and dorsal views (L 44.8 mm). (L–N) MZSP 125175, frontal, dorsal and right views (L 37.0 mm).

Fig. 2. *Kora corallina* shell and anatomical characters, light photos. (A) MZSP 151952#3 shell, frontal view, naturally broken (L 40.0 mm). (B) same, dorsal view. (C) same, right view. (D) same, left-slightly anterior view showing umbilicus. (E) same, apical view. (F), same, detail of apex, profile-slightly apical view, arrow indicating transition protoconch-teleoconch. (G) jaw in situ, specimen MZSP 132078#3, ventral view, adjacent tissues removed. (H) same for specimen MZSP 132078#2, younger one. (I) radula, isolated, extended and opened longitudinally, dorsal view, specimen MZSP 132078#1. (J) detail of shell aperture of specimen MZSP 132078#1 occluded by epiphragm. Scales= 1 mm.

Fig. 3. *Kora corallina* anatomical drawings. (A) extended pallial (pulmonary) cavity, ventral-inner view, inner edge of pneumostome sectioned and deflected upwards, transverse section of indicated region of kidney also shown. (B) head-foot, dorsal view, head, dorsal integument and internal organs removed, remaining muscles expanded. (C) foregut and midgut, mostly ventral view as in situ, topology of some adjacent structures also shown, 2 transverse sections

of indicated regions of esophagus and intestine also shown, small ventral portion of intestine adjacent to pericardium removed to show inner surface. Scales = 2 mm.

Fig. 4. *Kora corallina* anatomical drawings. (A) buccal mass, right view, dorsal wall sectioned and deflected to right, in inner-ventral view. (B) odontophore, right-anterior view, cartilages (oc) strongly curved inwards, with right m4 and peribuccal muscles (mj) deflected. (C) same, dorsal view, radula removed and deflected downwards (m7a, located inside radular sac, slightly deflected), left muscles as in situ, right muscles deflected externally. (D) spermoviduct, middle region, short portion in transverse view. (E) genital structures, dorsal view, mostly uncoiled. Scales = 1 mm.

Fig. 5. *Kora corallina* radulae in SEM. (A) whole view of a middle part, scale= 500 µm. (B) detail of marginal region, scale= 30 µm. (C) same, other level, scale= 30 µm. (D) detail of central region, scale= 50 µm. (E) detail of lateral region, scale= 30 µm. (F) detail of marginal region, another specimen, scale= 30 µm.

Fig. 6. *Kora corallina* anatomical drawings. (A) genital tubes portion preceding pore, dorsal view, all of them longitudinally opened. (B) genital structures in albumen gland level if it was transparent, ventral view. (C) penis, ventral view, longitudinally opened. (D) central nervous system (nerve ring), ventral view. Scales = 1 mm.

Fig. 7. *Kora nigra* shell characters. (A–B) Holotype MZSP 106230, frontal and dorsal views (L 30.1 mm). (C) shell of dissected specimen MZSP 151828#2, frontal view (L 41.8 mm); (D) same, right view. (E) same, right-slightly ventral view. (F) same, detail of apex, profile-slightly apical view, arrow indicating transition protoconch-teleoconch, scale= 1 mm. (G) same, apical view. (H) same, anterior-slightly left view. (I) same, dorsal view. (J–L) shell of dissected specimen MZSP 151828#1, frontal, dorsal and right views (L 40.6 mm). (M–O) MZSP 151827, frontal, dorsal and right views (L 43.3 mm).

Fig. 8. *Kora nigra* shell and anatomical characters, light photos. (A–C) shell MZSP 151830, frontal, dorsal and right views (L 39.9 mm). (D) contracted MZSP 151828#1 specimen just removed from shell, detail of mantle edge and part of head-foot in pneumostome region, frontal view. (E) jaw, ventral view, MZSP 151828#1. (F) spermatophore extracted from duct of bursa copulatrix of specimen MZSP 151828#2. (G) same, detail of its stem middle portion. Scales= 1 mm.

Fig. 9. *Kora nigra* anatomical drawings. (A) extended pallial (pulmonary) cavity, ventral-inner view, inner edge of pneumostome sectioned and deflected upwards, transverse section of indicated region of kidney also shown. (B) head-foot, dorsal view, head, dorsal integument and internal organs removed, remaining muscles expanded. (C) midgut, ventral view, esophagus slightly displaced ventrally. (D) spermoviduct, transverse section of middle region. Scales = 2 mm.

Fig. 10. *Kora nigra* radulae in SEM. (A) whole view, scale= 500 µm. (B) detail of central region, scale= 30 µm. (C), detail of lateral region, scale= 20 µm. (D) detail of central region, scale= 200 µm. (E) detail of lateral region, scale= 20 µm. (F) detail of marginal region, scale= 30 µm.

Fig. 11. *Kora nigra* anatomical drawings. (A) genital structures, dorsal view, mostly uncoiled. (B) penis, ventral view, longitudinally opened, transverse section in its middle level also done. (C) genital structures in albumen gland level if it was transparent, ventral view. Scales = 2 mm.

Fig. 12. *Kora rupestris* shell and anatomical characters, light photos. (A–B) Holotype shell MZSP 121416, frontal and dorsal views (L 37.7 mm). (C) shell of dissected specimen MZSP 151890#3, frontal view (L 46.1 mm). (D) same, dorsal view. (E) same, right view. (F) same, right-slightly ventral view. (G) same, apical view. (H) same, left-slightly anterior view, showing umbilicus. (I–K) shell MZSP 151891, frontal, dorsal and right views (L 44.1 mm). (L) mouth, ventral view, MZSP 151890#2 with jaw exposed. (M) pallial (pulmonary) cavity, detail of anterior-left region, ventral view, MZSP 151890#1. (N) basal region of genital system, dorsal view, both left tubes (right in Fig.) opened longitudinally with inner surface exposed, only penis base shown, MZSP 151890#1. (O) spermatophore found inside duct of bursa copulatrix of MZSP 151890#1 (some mucus still covering it) (P) gonad in situ, ventral view, #2. Scales= 1 mm.

Fig. 13. *Kora rupestris* anatomical drawings. (A) extended pallial (pulmonary) cavity, ventral-inner view, inner edge of pneumostome sectioned and deflected upwards, transverse section of indicated region of kidney also shown. (B) foregut, right view. (C) genital structures, dorsal view, mostly uncoiled. (D) spermoviduct, transverse section of middle region. Scales = 2 mm.

Fig. 14. *Kora rupestris* radulae in SEM. (A) central view, scale= 100 µm. (B) same, detail, scale= 20 µm. (C) lateral region, scale= 50 µm. (D) same, detail, scale= 10 µm. (E) marginal region, scale= 30 µm.

Fig. 15. *Kora rupestris* anatomical drawings. (A) genital structures in albumen gland level if it was transparent, ventral view. (B) penis, uncoiled distal end. (C) penis, ventral view, longitudinally opened. Scales = 2 mm.

Fig. 16. *Kora tupan* shell characters. (A) holotype MZSP 161200, frontal view (L 55.8 mm). (B) same, dorsal view. (C) same, right view. (D) same, right-slightly ventral and anterior view. (E) same, apical view. (F) same, left-slightly anterior view showing umbilicus. (G) apex, profile, arrow indicating transition protoconch-teleoconch. (H–J) paratype MZSP 151817, frontal, dorsal and right views (L 47.0 mm). (K) paratype MZSP 151823, frontal view (L 53.1 mm). (L) same, detail of apex, profile-slightly apical view, arrow indicating transition protoconch-teleoconch. (M) same, dorsal view. Scales= 1 mm.

Fig. 17. *Kora tupan* shell and anatomical characters, light photos. (A) jaw in situ, ventral view, MZSP 151817#1. (2) protoconch of holotype (MZSP 161200) and first teleoconch whorl, profile, arrow indicating transition protoconch-teleoconch. (C) buccal mass, ventral view, odontophore sectioned along left edge and deflected to right, inner dorsal surface exposed, MZSP 151817#1. (D) spermatophore found inside duct of bursa copulatrix of MZSP 151817#1 (broken at end). (E) pallial hoof (lung), ventral view, detail of antero-left region showing venation and pallial micro musculature, MZSP 151817#2. (F) region of pneumostome, ventral view, its inner edge sectioned and deflected upwards to show inner structures, MZSP 151817#1. (G) gonad in situ, ventral view, MZSP 151817#2. Scales= 1 mm.

Fig. 18. *Kora tupan* anatomical drawings. (A) extended pallial (pulmonary) cavity, ventral-inner view, inner edge of pneumostome sectioned and deflected upwards, transverse section of indicated region of kidney also shown. (B) head-foot, dorsal view, head, dorsal integument and internal organs removed, remaining muscles expanded. C) foregut and midgut, mostly ventral view as in situ, topology of some adjacent structures (gonad included) also shown, distal esophageal region shown if transparent. Scales = 5 mm.

Fig. 19. *Kora tupan* radulae in SEM. (A) detail of central region. (B) detail of lateral-marginal regions. Scales= 200 µm.

Fig. 20. ***Kora tupan*** **anatomical drawings**. (A) genital structures, dorsal view, mostly uncoiled. (B) genital tubes portion preceding pore, dorsal view, 2 of them longitudinally opened. (C) spermoviduct, middle region, short portion in transverse view. (D) Penis longitudinally opened, transverse section of indicated region of epiphallus also shown, short portion of adjacent regions of genital tubes also shown. (E) genital structures in albumen gland level if it was transparent, ventral view. Scales = 2 mm.

Fig. 21. ***Kora ajar*** **shell characters**. (A) holotype MZSP 163700, frontal view (L 47.4 mm). (B) same, dorsal view. (C) same, right view. (D) same, right-slightly ventral view. (E) same, apical view. (F) same, left view showing umbilicus. (G) same, detail of apex, arrow showing transition protoconch-teleoconch, scale= 1 mm. (H–J) paratype MZSP 151866#1, frontal, dorsal and right views (L 43.0 mm). (K–L) paratype MZSP 151866#2, frontal and right views (L 49.1 mm).

Fig. 22. ***Kora ajar*** **shell and anatomical characters, light photos**. (A) apex of MZSP 151866#1, profile-slightly apical view, arrow showing transition protoconch-teleoconch. (B) jaw in situ, ventral view, MZSP 151829#1. (C) reno-pericardial area, ventral view, kidney ventral wall opened along left edge and deflected upwards, ventral pericardial wall and head-foot removed, MZSP 151829#1. (D) spermatophore found inside duct of bursa copulatrix of MZSP 151829#2, stem digitally restored (it is broken in 3 levels). (E) same, detail of stem middle region. (F) extended pallial (pulmonary) cavity, ventral-inner view, inner edge of pneumostome sectioned and deflected upwards. Scales= 1 mm.

Fig. 23. ***Kora ajar*** **anatomical drawings**. (A) head-foot, dorsal view, head, dorsal integument and internal organs removed, remaining muscles expanded. (B) penis, ventral view, longitudinally opened, transverse section of indicated region of epiphallus also shown. (C) genital structures, dorsal view, mostly uncoiled. (D) spermoviduct, transverse section of middle region. (E) genital structures in albumen gland level if it was transparent, ventral view. (F) Posterior end of pallial cavity and foregut-midgut, mostly ventral view as in situ, topology of some adjacent structures also shown, distal esophageal region shown if transparent, transverse section of indicated region of kidney also shown. Scales = 2 mm.

Fig. 24. ***Kora ajar*** **radulae in SEM**. (A) detail of central region. (B) detail or lateral region. (C) detail of marginal region. Scales= 100 µm.

Fig. 25. ***Kora aetheria*** **shell characters**. (A) holotype MZSP 163400, frontal view (L 29.4 mm). (B) same, dorsal view. (C), same, right view. (D) same, right-slightly antero-ventral view. (E) same, apical view. (F) same, left view showing umbilicus. (G) apex, profile-slightly apical view, scale= 1 mm. (H–I) paratype MZSP 153856, frontal and dorsal views (L 30.3 mm). (J–L) paratype 152249, frontal, dorsal and right views (L 32.6 mm).

Fig. 26. ***Kora aetheria*** **shell and anatomical characters, light photos, SEM of radula**. (A) extended pallial (pulmonary) cavity, ventral-inner view, inner edge of pneumostome sectioned and deflected upwards, MZSP 153856#1. (B) shell apex, profile-slightly apical view, MZSP 153856#2. (C) reno-pericardial area, ventral view, kidney ventral wall opened along left edge and deflected upwards, ventral pericardial wall removed, MZSP 153856#1. (D) jaw in situ, ventral view, MZSP 153856#1. Scales = 1 mm. (E–G) Radulae in SEM, scales= 100 µm. (E) detail of central region. (F) detail of lateral region. (G) detail of marginal region.

Fig. 27. ***Kora aetheria*** **anatomical drawings**. (A) head-foot, dorsal view, head, dorsal integument and internal organs removed, remaining muscles expanded. (B) foregut and midgut, mostly ventral view as in situ, topology of some adjacent structures also shown (gonad

included), 3 transverse sections of indicated regions of esophagus also shown, distal portion of esophagus represented if it was transparent. (C) genital structures, dorsal view, mostly uncoiled. (D) penis, ventral view, longitudinally opened. (E) genital structures in albumen gland level if it was transparent, ventral view. (F) spermoviduct, transverse section of middle region. Scales = 2 mm.

Fig. 28. ***Kora jimenezi* shell characters**. (A) holotype MZSP 151907, frontal view (L 43.8 mm). (B) same, dorsal view. (C) same, right view. (D) same, right-slightly ventral view. (E) same, apical view. (F) same, left-slightly anterior view, showing umbilicus. (G) same, detail of apex, profile, scale= 1 mm. (H–I) paratype MZSP 151906#1, frontal and dorsal views (L 41.3 mm). (J–K) paratype MZSP 151906#2, frontal and dorsal views (L 37.6 mm).

Fig. 29. ***Kora jimenezi* shell characters**. (A–C) paratype MZSP 151906#3 (L 38.6 mm), frontal, dorsal, and right views. (D) same, detail of apex in profile, scale= 1 mm. (E–F) paratype MZSP 151906#4 (L 41.4 mm), frontal and dorsal views. (G) paratype MZSP 151906#5 (L 42.5 mm), frontal view. (H–I) paratype MZSP 151906#6 (L 23.8 mm), young specimen, frontal and dorsal views. (J) same, detail of apex, profile-slightly apical view, scale= 1 mm.

Fig.30. ***Kora jimenezi* anatomical drawings**. (A) head-foot, dorsal view, head, dorsal integument and internal organs removed, remaining muscles expanded. (B) foregut and midgut, mostly ventral view as in situ, topology of some adjacent structures also shown (gonad included), transverse section of indicated region of kidney also shown. (C) buccal mass, right view, esophagus and m2 only partially shown. (D) odontophore, dorsal view, superficial layer of membranes and muscles removed, both cartilages deflected, left muscles as in situ, right muscled expanded. Scales= 2 mm.

Fig.31. ***Kora jimenezi* anatomical photos**. (A) extended pallial (pulmonary) cavity, ventral-inner view, inner edge of pneumostome sectioned and deflected upwards, scale= 5 mm. (B) mantle edge, frontal view as in situ, inner region not shown, scale= 2 mm. (C) same as Fig. A, detail of pneumostome region, scale= 2 mm. (D) same, detail of middle region showing longitudinal micro muscular fibers (mi), scale= 1 mm. (E) jaw in situ, ventral view, scale= 1 mm. (F) visceral mass, detail of posterior region partially uncoiled, right view, mantle opened longitudinally and deflected, scale= 2 mm. (G) reno-pericardial region, ventral view, scale= 2 mm. (H) same, posterior region of kidney opened longitudinally in its left edge and deflected upwards.

Fig. 32. ***Kora jimenezi* anatomical photos and radula in SEM**. (A) foregut, ventral view, odontophore removed, esophagus opened longitudinally, inner surface exposed, scale= 1 mm. (B) Radula in SEM, detail of central region, scale= 100 µm. (C) same, detail of lateral and marginal regions, scale= 100 µm. (D) same, wide view of marginal region, scale= 200 µm.

Fig. 33. ***Kora jimenezi* anatomical drawings**. (A) spermoviduct, transverse section in its middle region. (B) genital structures, dorsal view, gonad extracted (see Fig. 30B, 31F). (C) same, detail of anterior region, dorsal view, female portions opened longitudinally. (D) penis, opened longitudinally, with transverse section of indicated region of epiphallus also shown. (E) genital structures in albumen gland level if it was transparent, ventral view. Scales= 2 mm.

Fig. 34. ***Kora uhlei* shell of types**. (A–G) holotype MZSP 165720 (L 41.4 mm). (A) frontal view. (B) dorsal view. (C) right view. (D) left-slightly anterior view. (E) right-slightly anterior view; (F) detail of apex in profile. (G) apical view. (H–N) paratype MZSP 164889#2 (L 40 mm). (H) apical view. (I) detail of apex in profile. (J) frontal view, (K) dorsal view. (L) right view. (M) left-slightly anterior view. (N) detail of umbilicus, left-slightly anterior view. Scales= 1 mm.

Fig. 35. *Kora uhlei* anatomical drawings. (A) head-foot, dorsal view, head, dorsal integument and internal organs removed, remaining muscles expanded, left ommatophore only partially shown. (B) foregut and midgut, mostly ventral view as in situ, topology of some adjacent structures also shown, portion of indicated portion of intestine shown opened. (C) buccal mass, ventral view, odontophore removed. Esophagus opened longitudinally. (D) odontophore, dorsal view, superficial layer of membranes and muscles removed, both cartilages deflected, left muscles as in situ, right muscled expanded. (E) spermoviduct, transverse section in its middle region. (F) genital structures, dorsal view. Scales= 2 mm.

Fig. 36. *Kora uhlei* anatomical fotos of holotype MZSP 165720. (A) extended pallial (pulmonary) cavity, ventral-inner view, inner edge of pneumostome sectioned and deflected upwards, scale= 5 mm. (B) same, detail of its mid region, showing some vessels and longitudinal micro-muscular fibers, scale= 2 mm. (C) jaw plate in situ, ventral view, scale= 1 mm. (D) posterior end of visceral mass, uncoiled and with part of superficial mantle removed, scale= 3 mm. (E) Buccal mass, left view, scale= 2 mm. (F) reno-pericardial region od pallial roof, ventral view, scale= 2 mm. (G) same, kidney ventral wall opened along its left edge and deflected upwards, exposing renal lobe, scale= 2 mm.

Fig. 37. *Kora uhlei* radulae in SEM. (A) panoramic view, scale= 50 µm. (B) detail of central region, scale= 100 µm. (C) detail of lateral and marginal region, scale= 100 µm. (D) detail of marginal region, scale= 50 µm.

Fig. 38. *Kora uhlei* anatomical drawings. (A) genital structures in albumen gland level if it was transparent, ventral view. (B) genital structures, detail of anterior region, dorsal view, female portions opened longitudinally. (C) penis, opened longitudinally, epiphallus with only small window-like orifice. Scales= 2 mm.

Fig. 39. *Kora kremerorum* holotype MZSP 151809 shell characters. (A) frontal view (L 40.8 mm). (B) dorsal view. (C) right view. (D) right-slightly ventral view. (E) left-slightly anterior view showing umbilicus. (F) apical view. (G) detail of apex, profile-slightly apical view, scale= 1 mm.

Fig. 40. *Kora kremerorum* paratypes MZSP 151810 shell characters. (A–C) #1 (L 48.6 mm), frontal, dorsal and right views. (D–E) #2 (L 41.9 mm), frontal and dorsal views. (F–H) #3 (L 43.5 mm). (I–J) #4 young specimen (L 30.9 mm), frontal and dorsal views. (K) same, detail of apex, profile-slightly apical view, scale= 1 mm.

Fig. 41. *Kora vania* types. (A–H) holotype MZSP 165500 (L 38.0 mm). (A), frontal view. (B) dorsal view. (C) right view. (D) right-slightly ventral view. (E) left-slightly anterior view. (F) apical view. (G) apex in profile, scale= 2 mm. (H) same, apical view. (I–K) paratype MZSP 163776#1 (L 34.7 mm), frontal, dorsal and right views. (L–M) paratype MZSP 163776#2 (L 38.8 mm), frontal and dorsal views.

Fig. 42. *Kora curumim* holotype MZSP 152077 shell characters. (A) frontal view (L 27.7 mm). (B) dorsal view. (C) right view. (D) right-slightly ventral view. (E) detail of anterior region, left-slightly ventral view, scale= 5 mm.

Fig. 43. *Koltrora pyrostoma* holotype MZSP 163500 shell characters. (A) frontal view (L 29.1 mm), specimen alive retracted inside. (B) same, right view. (C) same, dorsal view. (D) present state after extraction of specimen, frontal view. (E) right-slightly ventral view. (F) anterior-left view, showing umbilicus. (G) apical view. (H) apex, profile. (I) protoconch, profile. Scales= 1 mm.

Fig.44. *Koltrora pyrostoma* paratypes shells and radula. (A–C) MZSP 191791, frontal, right and dorsal views (L 31.9 mm). (D) MZSP 151789, frontal view (L 27.0 mm). (E) same, detail of apex, profile-slightly apical view, scale= 1 mm. (F–G) radula in SEM, central region, scales= 50 µm. (H) same, lateral region, scale= 50 µm.

Fig. 45. *Koltrora pyrostoma* anatomical features in light photos of paratypes MZSP 151789. (A–B) alive retracted specimen #7, frontal and dorsal views (L 26.3 mm). (C) same, detail of apex, profile-slightly apical view. (D) specimen #6, frontal view, with epiphragm (L 24.2 mm). (E) head-foot anterior view, forceps deflecting integument upwards to show genital aperture (fp). (F) reno-pericardial region, ventral view, ventral wall of kidney cut along left edge and deflected upwards. (G–H) alive semi-retracted specimens showing red mantle edge (average L 36 mm). (J) extracted specimen, anterior region, right view. (I) jaw in situ, ventral view. (K) pulmonary cavity, anterior-right region, ventral edge of pneumostome sectioned and deflected upwards. Scales= 1 mm.

Fig. 46. *Koltrora pyrostoma* anatomical drawings. (A) extended pallial (pulmonary) cavity, ventral-inner view, inner edge of pneumostome sectioned and deflected upwards, transverse section of indicated region of kidney also shown. (B) head-foot, dorsal view, head, dorsal integument and internal organs removed, remaining muscles expanded. (C) foregut and midgut, mostly ventral view as in situ, topology of some adjacent structures also shown. Scales = 2 mm.

Fig.47. *Koltrora pyrostoma* anatomical drawings. (A) buccal mass, isolated dorsal wall, ventral view, with adjacent portion of esophagus. (B) odontophore, dorsal view, superficial layer of muscles and membranes removed, both cartilages deflected, left muscles as in situ, right muscles deflected outside. (C) genital structures in albumen gland level if it was transparent, ventral view. (D) genital structures, dorsal view, mostly uncoiled. (E–F) spermoviduct, transverse sections of indicated regions. Scales = 1 mm.

Fig. 48. *Koltrora pyrostoma* anatomical drawings. (A) central nervous system (nerve ring), dorsal view. (B) basal end of spermoviduct, dorsal view, mostly opened longitudinally, transverse section in subterminal region of sperm inner longitudinal fold (sp) artificially done, some adjacent structures also shown. (C) penis, ventral view, longitudinally opened, some adjacent structures also shown. (D) nerve ring, ventral view. Scales = 1 mm.

Fig. 49. *Neopetraeus lobbi* shell and anatomical characters, specimen MZSP 158045. (A) frontal view (L 40.2 mm). (B) dorsal view. (C) right view. (D) right-slightly ventral view. (E) left-anterior view. (F) apical view. (G) apex, profile. (H) nerve ring, detail of pair of pedal ganglia, ventral view, with detail of statocyst enlarged. (I) jaw in situ, ventral view. Scales= 1 mm. (J–M) Lectotype NHMUK 1975431, frontal, right, dorsal and apical views (L 44.4 mm) (copyright © The Trustees of the Natural History Museum, London, published under permission).

Fig. 50. *Neopetraeus lobbi* anatomical drawings. (A) extended pallial (pulmonary) cavity, ventral-inner view, inner edge of pneumostome sectioned and deflected upwards, transverse section of indicated region of kidney also shown. (B) head-foot, dorsal view, head, dorsal integument and internal organs removed, remaining muscles expanded. (C) foregut, right view. (D) odontophore, dorsal view, cartilages deflected, radula removed and deflected to left, right muscles expanded, left muscles as in situ. Scales = 2 mm.

Fig. 51. *Neopetraeus lobbi* anatomical drawings. (A) foregut and midgut, mostly ventral view as in situ, topology of some adjacent structures also shown, with detail of indicated stretch of

intestine longitudinally opened. (B) buccal mass, ventral view, odontophore sectioned along its right and posterior edges and deflected to right, esophagus partially opened longitudinally. (C) odontophore, anterior view, cartilages (oc) curved ventrally, peribuccal muscles (mj) sectioned transversally and its anterior half removed, both (mj-oc) deflected from each other, right muscles (inferior in Fig.) expanded, left muscles as in situ. (D) spermoviduct, transverse section of middle region. (E) central nervous system (nerve ring), dorsal view. Scales = 1 mm.

Fig. 52. *Neopetraeus lobbi* radulae in SEM. (A) panoramic view, scale= 500 µm. (B) wide view, scale= 200 µm. (C) detail of central region, scale= 100 µm. (D) same, higher magnification, scale= 20 µm. (E) detail of lateral and marginal regions, scale= 50 µm.

Fig. 53. *Neopetraeus lobbi* anatomical drawings. (A) penis, ventral view, longitudinally opened, transverse section artificially done in its middle level, short portion of middle region of epiphallus (eh) also done, some adjacent structures also shown. (B) genital structures, dorsal view, mostly uncoiled. (C) spermoviduct, basal region longitudinally mostly opened, some adjacent structures also shown. (D) genital structures in albumen gland level if it was transparent, ventral view. Scales = 2 mm.

Fig. 54. *Neopetraeus tessellatus* shell characters (MZSP 158044). (A) complete specimen #1 still inside shell, frontal view (L 36.9 mm). (B) same, specimen extracted by means of a hole done in beginning of last whorl. (C), same, dorsal view. (D) same, right view. (E) same, right-slightly ventral view. (F) same, anterior view, showing umbilicus. (G) same, apical view. (H–I) specimen #2, frontal and dorsal views (L 33.1 mm). (J) same, detail of apex, profile-slightly apical view. (K) same, detail of second whorl of protoconch showing sculpture. Scales= 1 mm.

Fig. 55. *Neopetraeus tessellatus* anatomical characters (MZSP 158044) light photos. (A) buccal mass, ventral view, odontophore sectioned along right edge (left in Fig.) and deflected to right, showing dorsal inner surface of oral cavity. (B) foregut, dorsal view, dilated esophageal region opened longitudinally showing its inner surface. (C) intestine, ventral view, region close to pericardial region, opened longitudinally, adjacent structures also shown. (D) reno-pericardial region of pallial roof, ventral view, renal wall sectioned along its left edge and deflected upwards. (E) distal end of penis and epiphallus, opened longitudinally. (F) Jaw in situ, ventral view. Scales= 1 mm. (G–I) radulae in SEM. (G) detail of central region, scale= 30 µm. (H) detail of lateral and marginal regions, scale= 60 µm. (I) detail of central region, scale= 60 µm.

Fig. 56. *Neopetraeus tessellatus* anatomical drawings. (A) extended pallial (pulmonary) cavity, ventral-inner view, inner edge of pneumostome sectioned and deflected upwards. (B) foregut, right extended view. (C) odontophore, dorsal view, superficial layer of muscles and membranes removed, radula removed and deflected downwards, right muscles also deflected. Scales= extended pallial (pulmonary) cavity, ventral-inner view, inner edge of pneumostome sectioned and deflected upwards. Scales= 2 mm.

Fig. 57. *Neopetraeus tessellatus* anatomical drawings of genital structures. (A) whole dorsal view, uncoiled, terminal end of visceral mass also shown. (B) genital structures in albumen gland level if it was transparent, as in situ, ventral view. (C) genital tubes portion preceding pore, dorsal view, 2 of them longitudinally opened. Scales= 2 mm.

Fig. 58. Gallery of studied shells in same scale, frontal views. (A) *Kora corallina* holotype MZSP 103910. (B) *K. nigra* holotype MZSP 106230. (C) *K. rupestris* holotype MZSP 121416. (D) *K. tupan* holotype MZSP 161200. (E) *K. ajar* holotype MZSP 163700. (F) *K. aetheria* holotype MZSP 163400. (G) *K. jimenezi* holotype MZSP 151907. (H) *K. kremerorum* holotype MZSP 151809. (I)

K. uhlei holotype MZSP 165720. (J) *K. vania* holotype MZSP 165500. (K) *K. curumim* holotype MZSP 152077. (L) *Koltrora pyrostoma* holotype MZSP 163500. (M) *Neopetraeus lobbii* MZSP 158045. (N) *Neopetraeus tessellatus* MZSP 158044. Scale= 10 mm.

Fig. 59. *Anctus angiostomus* anatomical features, MZSP 27937, 4 spm. (A) shell (L 28.1 mm), frontal view. (B) same, dorsal view; (C) same, right view; (D) same left view; (E) detail of apex, profile-slightly apical view. (F) nerve ring, ventral view. (G) buccal mass, right view. (H) jaw in situ, ventral view. (I) region of carrefour of reproductive system, removed from albumen gland, ventral view. (J) odontophore, dorsal view, both cartilages deflected, right muscles expanded, left muscles as in situ. (K) dorsal wall of buccal mass, ventral-inner view, odontophore removed. Scales= 1 mm.

Fig. 60. Self-produced maps with countries and states outlined. (top-right) Central and South America, Brazil painted blue. (top-left) Brazil with mark of region enlarged below. (center-below) region of studied samples indicated by black dots, except for *K. rupestris* (red). São Francisco River represented in orange. Abbreviations: BA, Bahia; K, *Kora*; MG, Minas Gerais.

Fig. 61. Single cladogram obtained analyzing the matrix (Appendix 2) of the species assembly reported in Material and Methods (written black), plus the species studied herein (new taxa written red). Branch below is the far outgroup (rooting). Each node has the set of synapomorphies shown with symbols, indicating: black square= non-homoplastic synapomorphy; white square= reversion; black circle= convergence. Above number each symbol is the character, number below is the state in that node. Blue number in italics are branches numeration. Method: parsimony; L= 391; CI= 54; RI= 76.

Appendix 1 –list of characters that base the phylogenetic analysis

1. Color: 0= lacking pigment; 1= mosaic of color (Olympus, Drymaeus, Sanniostracus, Anctus, Neopetraeus1); 2= brown with subsutural pale band (Kora); 3= uniform brown (Rhinus).
2. General form: 0= globose; 1= discoid (Olympus); 2= turriform (Lavajatus); 3= elliptic (remaining).
3. Size rank: 0= ~30 mm (Olympus, Lavajatus, Drymaeus, Bulimulus, Sanniostracus, Rhinus, Anctus, K nigra, K aetheria, Koltrora); 1= ~45 mm (K corallina, K rupestris, K jimenezi, K uhlei, N tesselatus); 2= ~55 mm (K tupan, K itacarambi, N lobbi).
4. Range of length versus width: 0= ~2.0 (Drymaeus, Bulimulus, Sanniostracus, Anctus, K corallina, K rupestris. K tupan, K aetheria, K jimenezi, K uhlei, Koltrora, N lobbi; 1= ~1.5 (Rhinus, K nigra, K itacarambi, N tesselatus); 2= ~1 (Olympus); 3= ~7 (Lavajatus).
5. Dorso-ventral weak flattening: 0= absent; 1= present (K tupan, K aetheria, Koltrora)
6. Sculpture: 0= absent; 1= axial undulations (Kora, Drymaeus, Bulimulus, Anctus).
7. Spiral micro-stripes: 0= absent; 1= present (Rhinus, Kora).
8. Aperture: 0=indeterminate (not deflected); 1= deflected (determinate), but thin (Olympus, Drymaeus, Sanniostracus, Rhinus, Anctus); 2= deflected and thick (Kora) (additive?)
9. Aperture: 0= uniform with growth: 1= weakly dislocated to right (Kora, Koltrora, Neopetraeus).
10. Outer lip with horizontal superior implantation: 0= absent; 1= present (Rhinus, K nigra, K tupan, K itacarambi, K jimenezi,
11. Columellar middle wide fold: 0= absent; 1= present (Kora, Neopetraeus).
12. Umbilicus: 0=shallow; 1=covered ventrally by inner lip (Drymaeus, Kora, Bulimulus, Sanniostracus, Rhinus, Anctus. Koltrora, Neopetraeus)
13. Umbilicus: 0= restricted to peristome; 1= going further along columella (Kora, Neopetraeus).
14. Umbilicus: 0= closed to narrow; 1= widely opened (Anctus, K corallina, K nigra, K itacarambi, K aetheria, K jimenezi, Neopetraeus).
15. Protoconch sculpture: 0= absent; 1= reticulate (Drymaeus, Bulimulus, Sanniostracus, Rhinus); 2= smooth followed by axial cords (Kora).
16. Calcified epiphragm: 0= absent; 1= present (Kora, Koltrora) (? In Neopetraeus).
17. Pair of secondary columellar muscles: 0= absent (Olympus, Lavajatus); 1= present.
18. Secondary pair of columellar muscles with medial pair of bundles: 0= absent; 1= present (Kora).
19. Number of anterior insertions of left secondary pair of columellar muscles: 0= 2; 1= 3-4 (Anctus, K jimenezi, K uhlei, Koltrora, Neopetraeus); 2= 5 (K itacarambi, K aetheria); 3= 7-8 (K nigra, K rupestris, K tupan); 4= +10 (K corallina).
20. Number of anterior insertions of right secondary pair of columellar muscles: 0= 2-4; 1= 6-7 (K corallina, K, nigra, K rupestris, K tupan, K aetheria).
21. Secondary pair of columellar muscles with medial branch differentiated: 0= absent; 1= present (K nigra, K rupestris, K tupan, K itacarambi, K aetheria, K uhlei).
22. Mantle edge: 0= lacking secondary folds; 1= with large, triangular fold (Bulimulus, Sanniostracus, Anctus); 2= with rounded secondary folds (K corallina, K aetheria, K jimenezi, K uhlei, Koltrora); 3= with pointed secondary folds (K nigra, K rupestris, K tupan, K itacarambi).
23. Intercalated pair of vessels at left from pulmonary vessel: 0= absent; 1= present (Kora **except** K jimenezi, Koltrora).

24. Anterior end of pulmonary vessel (cv): 0= simple; 1= branched (K corallina, K tupan, K itacarambi, K aetheria, K uhlei, Neopetraeus).
25. Ureter aperture: 0= 100-30%; 1= ~15% (Drymaeus); 2= 5% (Olympus); 3= 0% (Lavajatus, Kora, Koltrora, Bulimulus, Sanniostracus, Rhinus, Anctus).
26. % of reno-pericardial area in pulmonary cavity: 0= ~1/4; 1= ~1/10 (Kora, Sanniostracus).
27. Kidney: 0= mostly solid; 1= hollow inner space surrounded by tall glandular folds (Kora); 2= hollow but extremely elongated (Olympus).
28. Jaw plate: 0= single plate; 1= strong central notch (Drymaeus, K corallina, K rupestris, K aetheria, Neopetraeus); 2= narrow horseshoe-shaped (Bulimulus, Rhinus, K jimenezi); 3= slightly rectangular (K nigra, K tupan, K itacarambi, K uhlei, Koltrora); 4= absent (Lavajatus).
29. Jaw plate sculpture: 0= fold-like; 1= rib-like (Kora).
30. Radula: clear separation between rachidian and lateral teeth: 0= present; 1= slightly different (Drymaeus, Bulimulus, Sanniostracus, Rhinus, Anctus, Neopetraeus); 2= absent (Kora, Koltrora).
31. Radula: clear separation between lateral and marginal teeth: 0= present; 1= slightly different (Drymaeus, Bulimulus, Sanniostracus, Rhinus, Anctus, Neopetraeus); 2= absent (Kora, Koltrora).
32. Radular teeth: 0= curved inwards; 1= curved outwards (Drymaeus, Bulimulus, Neopetraeus, Kora, Koltrora, Sanniostracus, Rhinus, Anctus).
33. Radular lateral teeth cusps: 0= single, blunt; 1= bifid, asymmetric (Olympus, Lavajatus, Drymaeus, Bulimulus, Sanniostracus, Rhinus, Anctus, Neopetraeus); 2= single, very long (Kora, Koltrora).
34. Radular marginal teeth cusps: 0= similar to lateral teeth; 1= multicuspid (Drymaeus, Sanniostracus, Rhinus).
35. Radular teeth width: 0=relatively wide; 1= very narrow (Kora, Koltrora).
36. Radular sac widely bulging posteriorly in buccal mass: 0= absent; 1= present (Kora).
37. Radular length: 0= slightly longer than buccal mass; 1= about twice buccal mass length (Kora).
38. Pair m1l: 0= absent; 1= present (Lavajatus, K rupestris, K tupan, K aetheria, K jimenezi, K uhlei, Koltrora, Neopetraeus).
39. Pair m1v duplicated: 0= absent; 1= present (Anctus, K corallina, K nigra).
40. M2 insertion: 0= as 2 separated bundles; 1= practically as single bundle (Drymaeus, Kora); 2= in anterior region (Lavajatus).
41. Pair m2a: 0= absent; 1= present (Anctus, K itacarambi, K jimenezi).
42. M3: 0= absent; 1= pair of transverse muscles in ventral region of esophageal origin (Sanniostracus, Rhinus, K. corallina); 2= pair dorso-ventral (Olympus, Anctus, Kora **except** K jimenezi).
43. M3: 0= absent; 1= as latero-lateral muscle in esophageal origin (Rhinus); 2= strong ventral pair (m3p) (Olympus).
44. M5: 0= separated from m4; 1= covering m4 (Bulimulus, Drymaeus, Sanniostracus, Anctus, Koltrora, Kora **except** following); 2= as continuation of m4 (K tupan, K uhlei); 3= as single mass (Olympus, Neopetraeus).
45. M6: 0= present; 1= absent (Lavajatus, Anctus, Kora).
46. M7: 0= absent (Anctus, Neopetraeus); 1= as 2 separated muscles (Olympus, Lavajatus, Bulimulus, Drymaeus, Sanniostracus, Koltrora); 2= as single narrow bundle (K corallina, K nigra, K rupestris, K itacarambi, K aetheria); 3= with 3 bundles (K tupan); 4= broad, thick (K jimenezi); 5= filiform (K uhlei).

47. M8: 0= absent; 1= present (Kora).
48. M10: 0= posterior inserted; 1= anterior inserted (Kora).
49. M10: 0= broad; 1= narrow (Kora **except** K tupan & K aetheria).
50. M11: 0= well-developed; 1= not detected (Kora, Koltrora, Drymaeus).
51. Mj forming ventral muscular platform: 0= absent; 1= present (Kora, Koltrora).
52. % of fusion between odontophore cartilages: 0= 0%; 1= ~20% (Olympus, Sanniostracus, Rhinus); 2= ~50% (Lavajatus, Drymaeus, N lobbii, K nigra); 3= ~80%; (K. corallina, K rupestris, K tupan, K itacarambi, K aetheria, K jimenezi, N tesselatus); 4= ~90% (K uhlei); 5= 100% (Anctus, Koltrora). (add)
53. Salivary gland: 0= small, as 2 separated masses; 1= large, fused in single mass (Drymaeus, Kora).
54. Salivary gland aperture in dorsal wall of buccal cavity: 0= in middle level; 1= in posterior region (Rhinus, Anctus, K tupan, K jimenezi, Koltrora, Neopetraeus).
55. Salivary gland aperture on a papilla: 0= absent; 1= present (K jimenezi, K uhlei).
56. Stomach: 0= bulbed; 1= as simple curve (Drymaeus, Sanniostracus, Rhinus, Kora, Anctus).
57. Duct to anterior lobe of digestive gland: 0= in esophagus (*D. currais)*; 1= in stomach (Drymaeus (except *D. currais*), Sanniostracus, Rhinus, Kora, Anctus).
58. Anterior duct to digestive gland: 0= branched to both sides; 1-= branched only to right side (K nigra, K tupan, K itacarambi, K jimenezi, K uhlei, Neopetraeus).
59. Posterior duct to digestive gland: 0= simple branched tube; 1= tube with right branches only (Kora **except** K nigra).
60. Anus: 0= opening inside lung; 1= opening directly outside (Olympus, Lavajatus, Bulimulus, Drymaeus, Sanniostracus, Rhinus, Kora, Anctus, Neopetraeus, Koltrora).
61. Hermaphrodite duct (hd) insertion: 0= simple; 1= strongly curved (Kora **except** K nigra, N lobbii).
62. Form of seminal receptacle: 0= balloon-like; 1= digitiform-cylindric (Bulimulus, Drymaeus, Sanniostracus, Rhinus, Anctus); 2= digitiform-flattened (Kora), 3= multilobed (Lavajatus).
63. Seminal receptacle: 0= straight; 1= curved (Anctus, K jimenezi).
64. Insertion of hermaphrodite duct in seminal receptacle: 0= in its base; 1= in its tip (Olympus, Lavajatus).
65. Carrefour form: 0= filiform; 1= conic (Drymaeus, Sanniostracus, Rhinus, Anctus, K corallina, K tupan, K itacarambi, K aetheria, K jimenezi, K uhlei, Neopetraeus), 2= entirely narrow (Bulimulus sula, K rupestris, Koltrora); 3= elongated (K nigra).
66. Carrefour duct: 0= absent; 1= narrow-long (Olympus, Lavajatus, Anctus, Drymaeus, Bulimulus, Sanniostracus, K nigra, K rupestris, K tupan, K aetheria; K uhlei, Koltrora, Neopetraeus); 2= narrow-short (K itacarambi, K jimenezi); 3= short-wide (Rhinus, K corallina)
67. Carrefour bulged distal region by side of receptacle: 0= absent; 1= present (Anctus, K tupan, K jimenezi, K uhlei, N lobbii).
68. Insertion of carrefour: 0= in spermoviduct (Olympus, Lavajatus, D. currais, K rupestris, Koltrora); 1= in duct of albumen gland (Anctus, Drymaeus castilhensis, D. micropyrus, K corallina, Neopetraeus); 2= between albumen gland duct and accessory albumen chamber (K nigra, K tupan, K uhlei); 3= in albumen chamber (K itacarambi, K jimenezi); 4= between albumen duct and spermoviduct (K aetheria).
69. Albumen chamber: 0= as separated sac; 1= as simple curve preceding spermoviduct (Anctus, K corallina, K itacarambi, K jimenezi, K uhlei, Neopetraeus); 2= double (Sanniostracus); 3= in intersection of both (Rhinus); 4= absent (Olympus, Lavajatus).

70. Accessory albumen chamber (as): 0= absent; 1= present (Kora, Neopetraeus); 2= a diverticulum (Sanniostracus).
71. % of prostate in spermoviduct: 0= ~50%; 1= ~35% (Drymaeus, K corallina, K nira, K aetheria, Koltrora); 2= ~20% (Neopetraeus).
72. Number of sperm folds in spermoviduct: 0= 1; 1= 2 (K nigra); 2= 0 (Bulimulus, Neopetraeus).
73. Uterus wall: 0= thin; 2= thick glandular (Drymaeus, Sanniostracus).
74. Vas deferens origin: 0= simple hole; 1= in confluence of pair of folds (Kora).
75. Duct of bursa copulatrix: 0= weakly muscular; 1= strongly muscular (Kora **except** K jimenezi).
76. Duct of bursa length: 0= considerable % of spermoviduct; 1= shorter than 25% of spermoviduct (Olympus, Lavajatus).
77. Vas deferens insertion in penis: 0= simple; 1= strongly curved (K nigra, K tupan, K itacarambi, K uhlei).
78. Penis form: 0= stubby-claviform; 1= slightly filiform (Lavajatus, Drymaeus, Sanniostracus, Kora, Anctus).
79. Penis length: 0= ~1/2 of spermoviduct; 1= ~75% of spermoviduct (Drymaeus, Sanniostracus, Anctus, K corallina, K aetheria); 2= 85-90% (K tupan, K itacarambi, N tesselatus); 3= ~100% (K nigra, K jimenezi).
80. Penis shield: 0= absent (Rhinus); 1= only in penis base (Bulimulus, Sanniostracus, Anctus, Kora); 2= ~1/3 pf penis (Drymaeus). (add)
81. Penis muscle insertion: 0= at tip of penis; 1= at tip of epiphallus (Drymaeus, Sanniostracus, Anctus, K corallina, K nigra, K tupan, K aetheria, K jimenezi, Neopetraeus); 2= base of epiphallus (K rupestris, K itacarambi, K uhlei); 3= subterminal (Olympus, Koltrora).
82. Penis distinct muscular basal wall: 0= absent; 1= present (kora **except** K jimenezi).
83. Penis inner organization: 0= single chamber with large fold; 1= 2 chambers separated by transverse fold (Bulimulus, Sanniostracus); 2= elongated chamber with special mosaic of low folds (Drymaeus, Rhinus, Anctus); 3= divided into compartments (Kora); 4= 2 chambers separated by middle narrow duct (Olympus).
84. Pair of longitudinal strong folds in proximal penial region: 0= absent; 1= present (Kora, Koltrora).
85. Type of pair of longitudinal folds: 0= simple (K nigra, K rupestris, K itacarambi, K jimenezi); 1= imbricated (K corallina, K aetheria, K uhlei); 2= forming wing-like folds (K tupan); 3= fused distally (Koltrora) (- in folds-lacking taxa).
86. Pair of longitudinal penial folds – imbricated projections: 0= entire (K corallina); 1= distal region only (K aetheria, K uhlei) (- for lacking).
87. Penis umbrella-like transverse fold: 0= absent; 1= present (K rupestris, K tupan, K itacarambi, K aetheria, K uhlei).
88. Number of rods in umbrella-like transverse fold: 0= 6 (K aetheria); 1= 5 (K itacarambi, K rupestris); 2= 3 (K tupan, K uhlei) (- in folds-lacking taxa).
89. Epiphallus length: 0= ~50% of penis; 1= absent (Olympus, Bulimulus); 2= ~10-15% of penis (Lavajatus, Drymaeus, Sanniostracus, Rhinus, K rupestris); 3= ~35% of penis (Anctus, K corallina, K aetheria, N lobbii); 4- 20-25% (K nigra, K tupan, K itacarambi, K jimenezi, K uhlei, Koltrora, N tesselatus).
90. Epiphallus inner large longitudinal fold: 0= absent; 1= present (Drymaeus, Sanniostracus, Rhinus, K corallina, K tupan, K itacarambi, Neopetraeus).
91. Folds converging to vas deferens aperture: 0= from penis; 1= from epiphallus (Kora **except** K jimenezi, K rupestris).

92. Nerve ring with pleural ganglia close to pedal ganglia: 0= absent; 1= present (Drymaeus, Sanniostracus, Rhinus, Anctus, Kora, Koltrora, Neopetraeus).
93. Pleural commissure: 0= absent; 1= present (Drymaeus, Sanniostracus, Rhinus, Anctus, Kora, Koltrora, Neopetraeus).
94. Cerebral commissure: 0= wide; 1= short (Drymaeus, Sanniostracus, Kora).

Appendix 2 –matrix of characters that base the phylogenetic analysis

Sp\character	1 2 3 4<br>1234567890 1234567890 1234567890 1234567890 12345
Kora corallina	2310011210 1111211141 0211311112 2120111011 01011
Kora nigra	2301011211 1111211131 1310311312 2120111011 02011
Kora rupestris	2310011210 1110211131 1310311112 2120111101 02011
Kora tupan	2320111211 1110211131 1311311312 2120111101 02021
Kora ajar	2321011211 1111211120 1311311312 2120111001 12011
Kora aetheria	2300111210 1111211121 1211311112 2120111101 02011
Kora jimenezi	2310011211 1111211110 0200311212 2120111101 10011
Kora uhlei	2310011210 1110211110 1211311312 2120111101 02021
Koltrora pyrostoma	2300100110 0100011010 0210300302 2120111100 00010
Neopetraeus lobbi	1320000110 11110?1010 0001000101 1110000100 00030
Neopetr. tesselatus	1311000110 11110?1010 0001000101 1110000100 00030
Drymaeus castilhensis	1300010100 0100101000 0000100001 1111000001 00010
Drymaeus micropyrus	1300010100 0100101000 0000100001 1111000001 00010
Drymaeus currais	1300010100 0100101000 0000100001 1111000001 00010
Bulimulus sula	0300010100 0100101000 0100300201 1110000000 00010
Sanniost. carnavalescus	1300000100 0100101000 0100310001 1111000000 01010
Rhinus botocudus	3301001101 0100101000 0000300201 1111000000 01100
Anctus angiostomus	1300010100 0101001010 0100300001 1110000010 12011
Olympus nimbus	1102000100 0000000000 0000202000 0010000000 02230
Lavajatus moroi	0203000000 0000000000 0000300400 0010000102 00001
Strophocheilidae	0000000000 0000000000 0000000000 0000000000 00000

Sp\character	4 5 6 7 8 9<br>67890 1234567890 1234567890 1234567890 1234567890 1234
Kora corallina	21111 1310011011 1200130111 1001100111 1131100-31 1111
Kora nigra	21111 1210011101 0200310201 1101101131 11310-0-40 1111
Kora rupestris	21111 1310011011 1200210001 0001100101 21310-1120 0111
Kora tupan	31101 1311011111 1200111201 0001101121 11312-1241 1111
Kora ajar	21111 1310011111 1200120311 0001101121 21310-1141 1111
Kora aetheria	21101 1310011011 1200110401 1001100111 1131111030 1111
Kora jimenezi	41111 1311111111 1210121311 0001000131 10310-0-40 0111
Kora uhlei	51111 1410111111 1200111211 0001101101 2031111240 1111
Koltrora pyrostoma	10001 1501000001 0000210000 1000000000 30013-0-40 0110
Neopetraeus lobbi	00000 0201000101 1000111111 2200000001 1000--0-31 0110
Neopetr. tesselatus	00000 0301000101 0000110111 2200000021 1000--0-41 0110
Drymaeus castilhensis	10001 0210011001 0100110100 1010000112 1020--0-21 0111
Drymaeus micropyrus	10001 0210011001 0100110100 1010000112 1020--0-21 0111
Drymaeus currais	10001 0210010001 0100110000 1010000112 1020--0-21 0111
Bulimulus sula	10000 0000000001 0100210000 0200000001 0010--0-10 0000
Sanniost. carnavalescus	10000 0100011001 0100110022 0010000111 1010--0-21 0111
Rhinus botocudus	00000 0101011001 0100130030 0000000000 0020--0-21 0110
Anctus angiostomus	00000 0501011001 0110111110 0000000111 1020--0-30 0110
Olympus nimbus	10000 0100000001 0001010040 0000010000 3040--0-10 0000
Lavajatus moroi	10000 0200000001 0301010040 0000010100 0000--0-20 ?000
Strophocheilidae	00000 0000000000 0000000000 0000000000 0000--0-00 0000

Figure 1

Click here to access/download;Figure;Kora 01b corallina.jpg

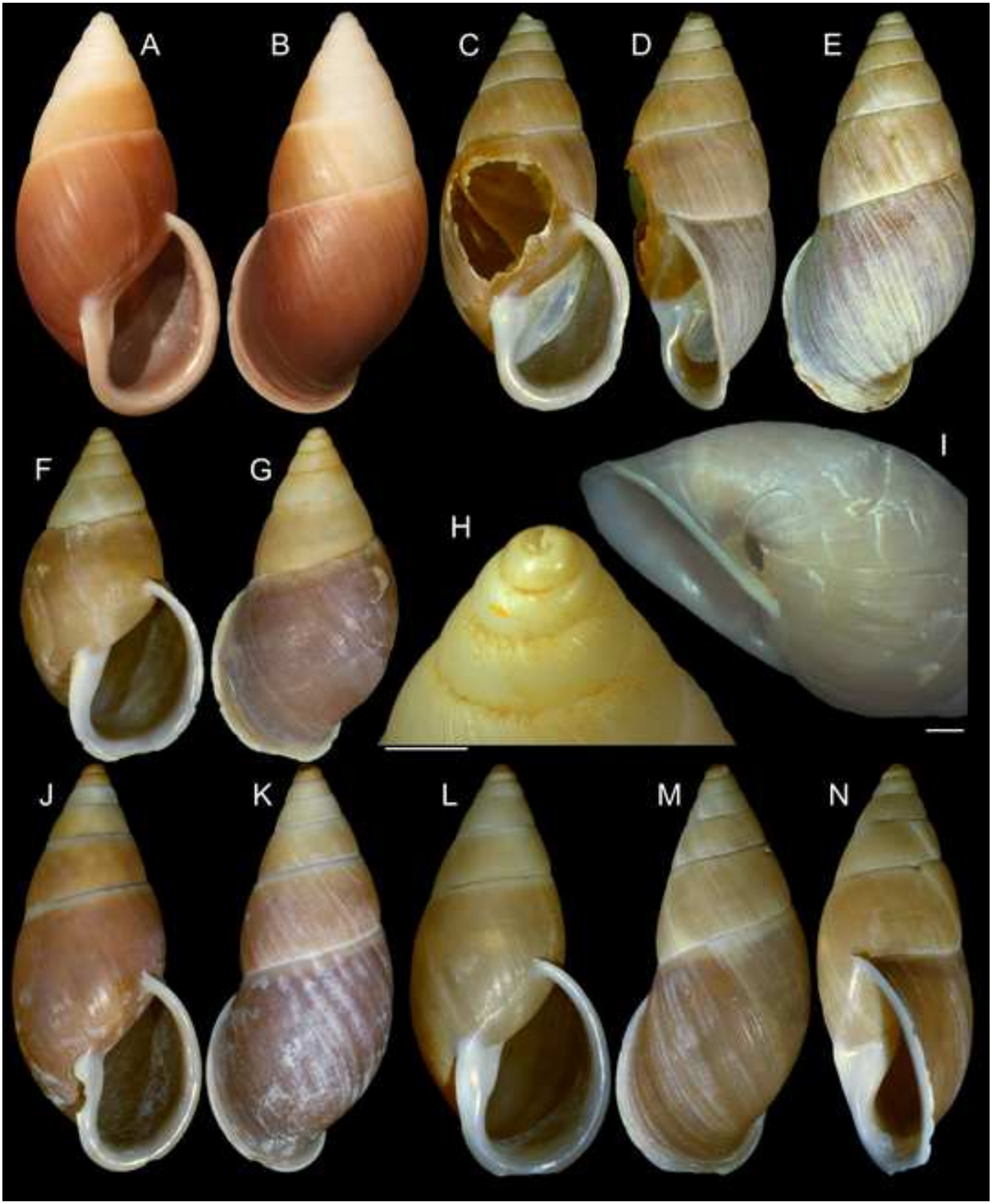

Figure 2

Click here to access/download;Figure;Kora 02b corallina.jpg

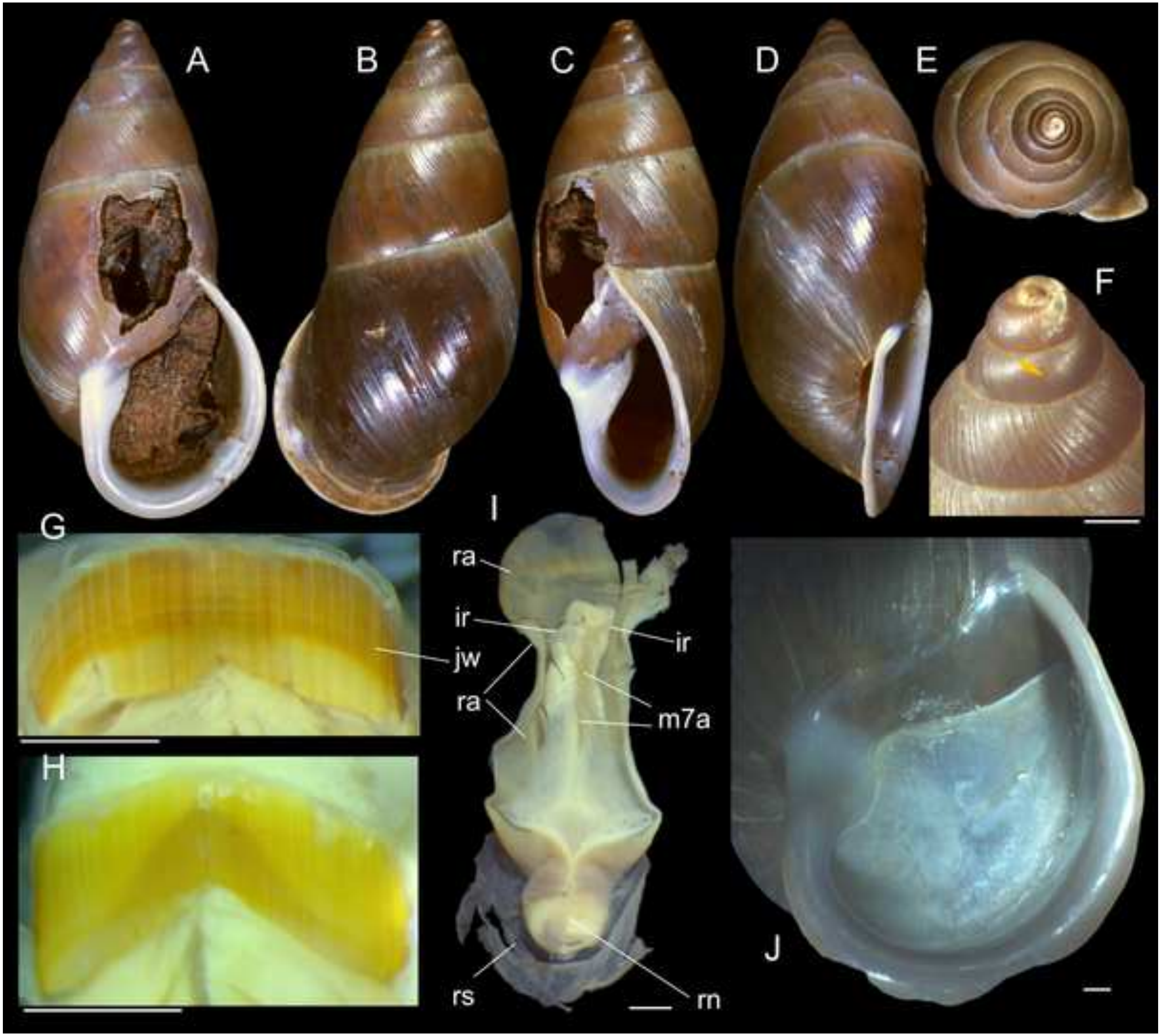

Figure 3

Click here to access/download;Figure;Kora 03b corallina.jpg

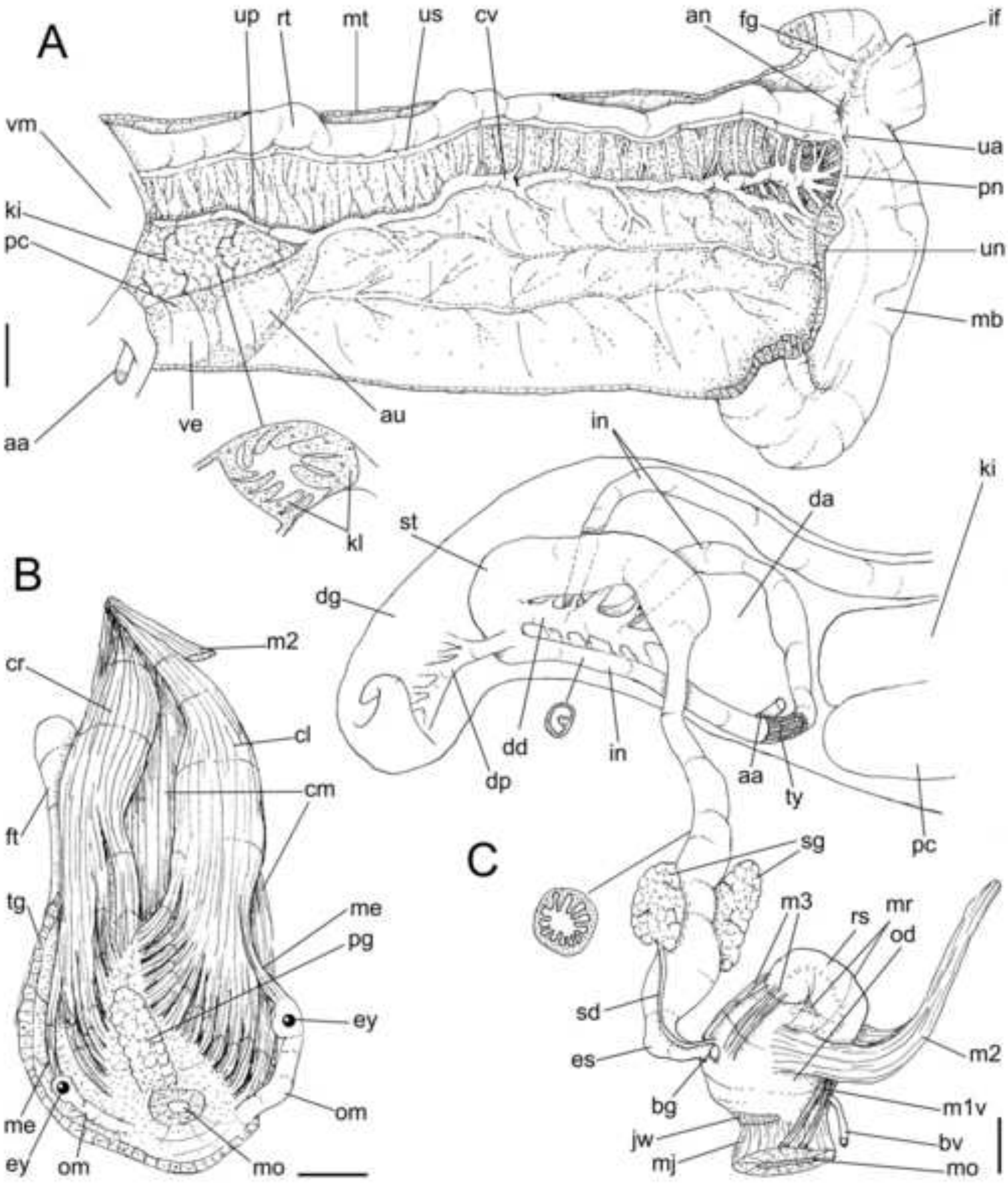

Figure 4

Click here to access/download;Figure;Kora 04b corallina.jpg

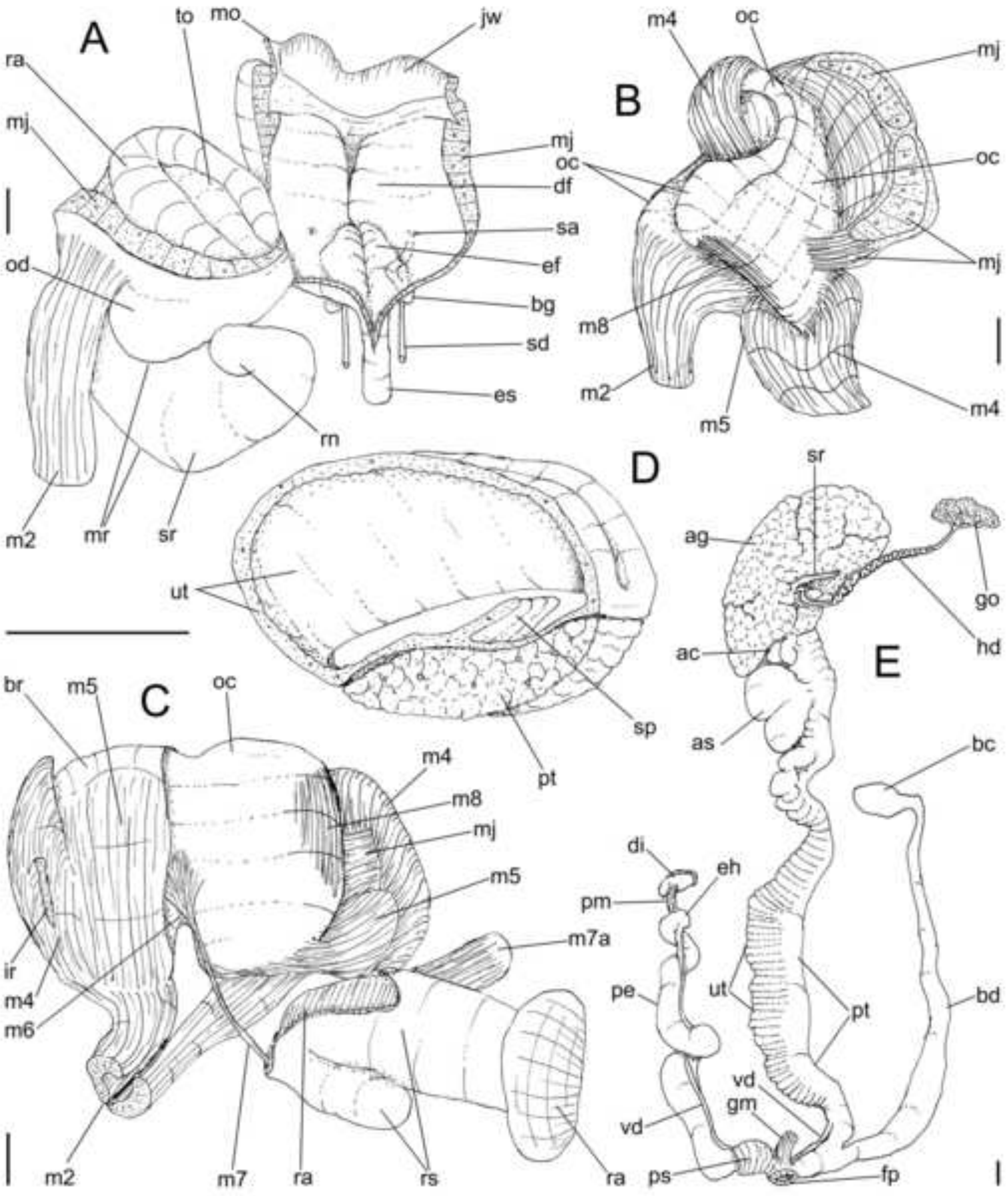

Figure 5

Click here to access/download;Figure;Kora 05b corallina.jpg

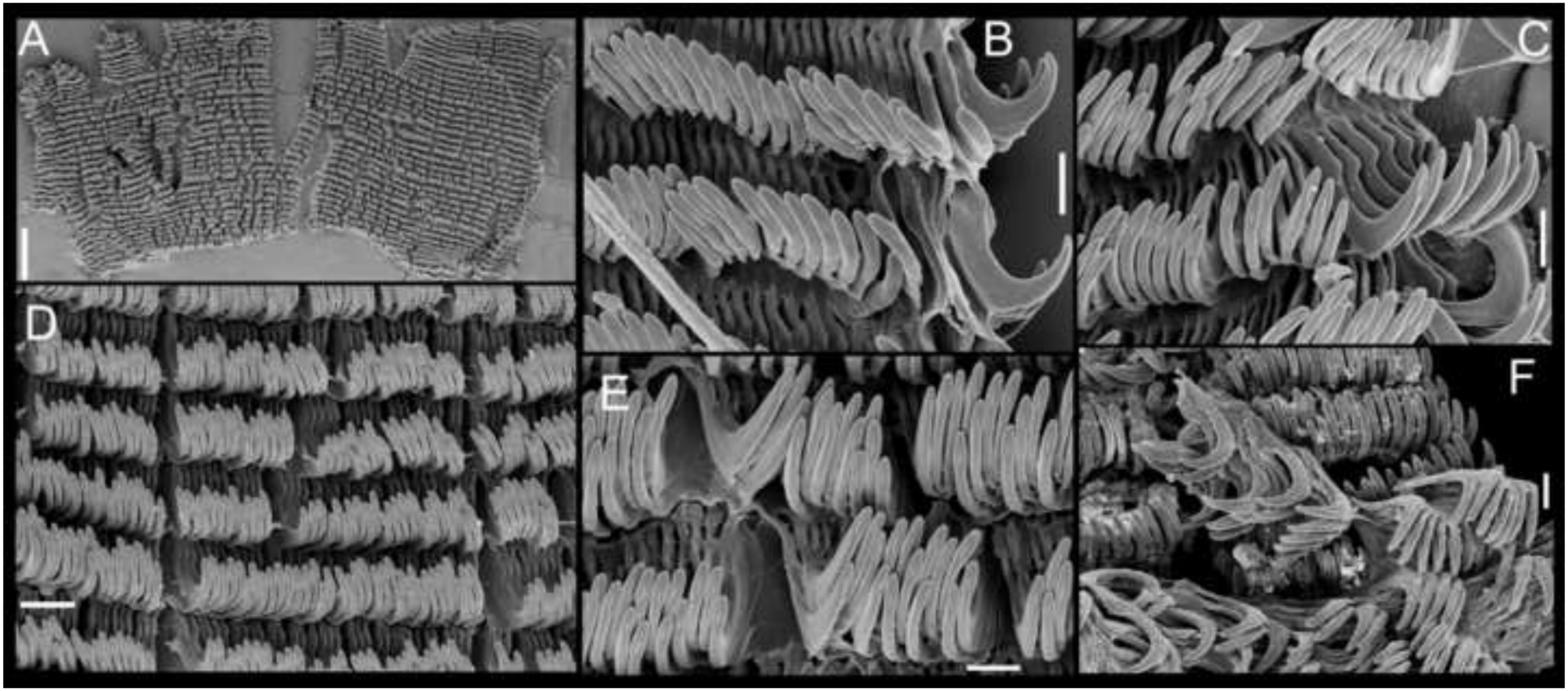

Figure 6

Click here to access/download;Figure;Kora 06b corallina.jpg

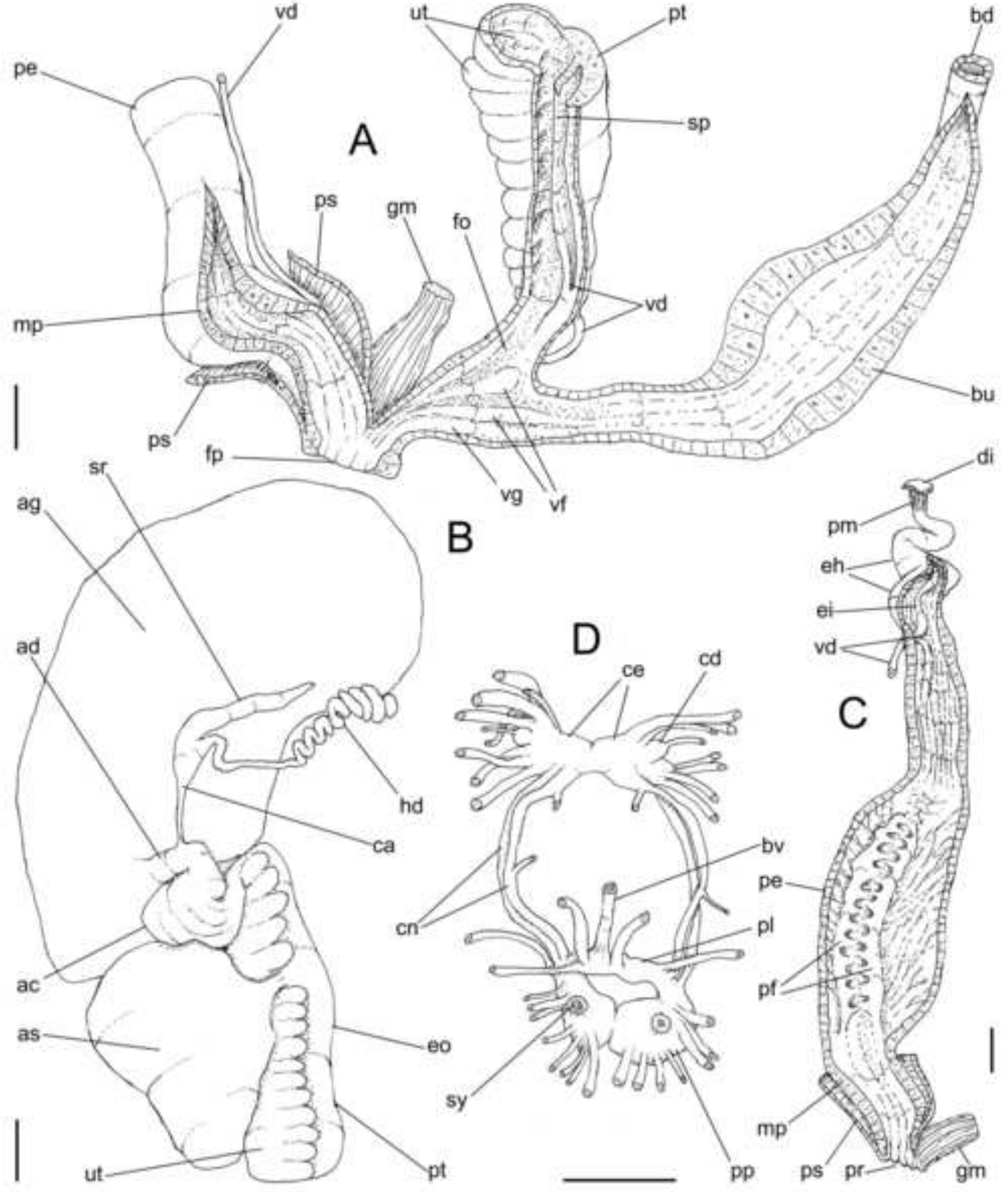

Figure 7 Click here to access/download;Figure;Kora 07b nigra.jpg

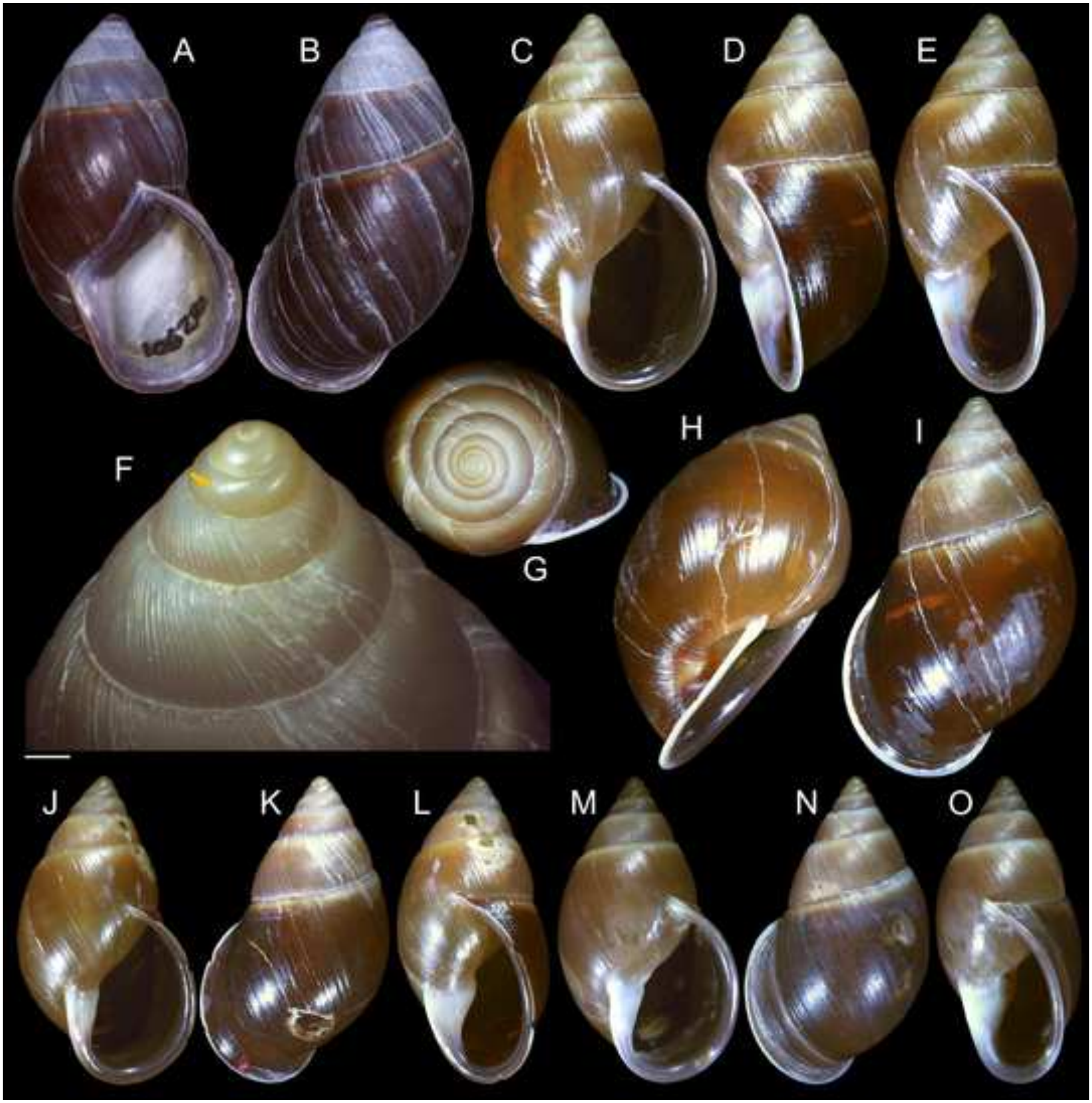

Figure 8

Click here to access/download;Figure;Kora 08b nigra.jpg

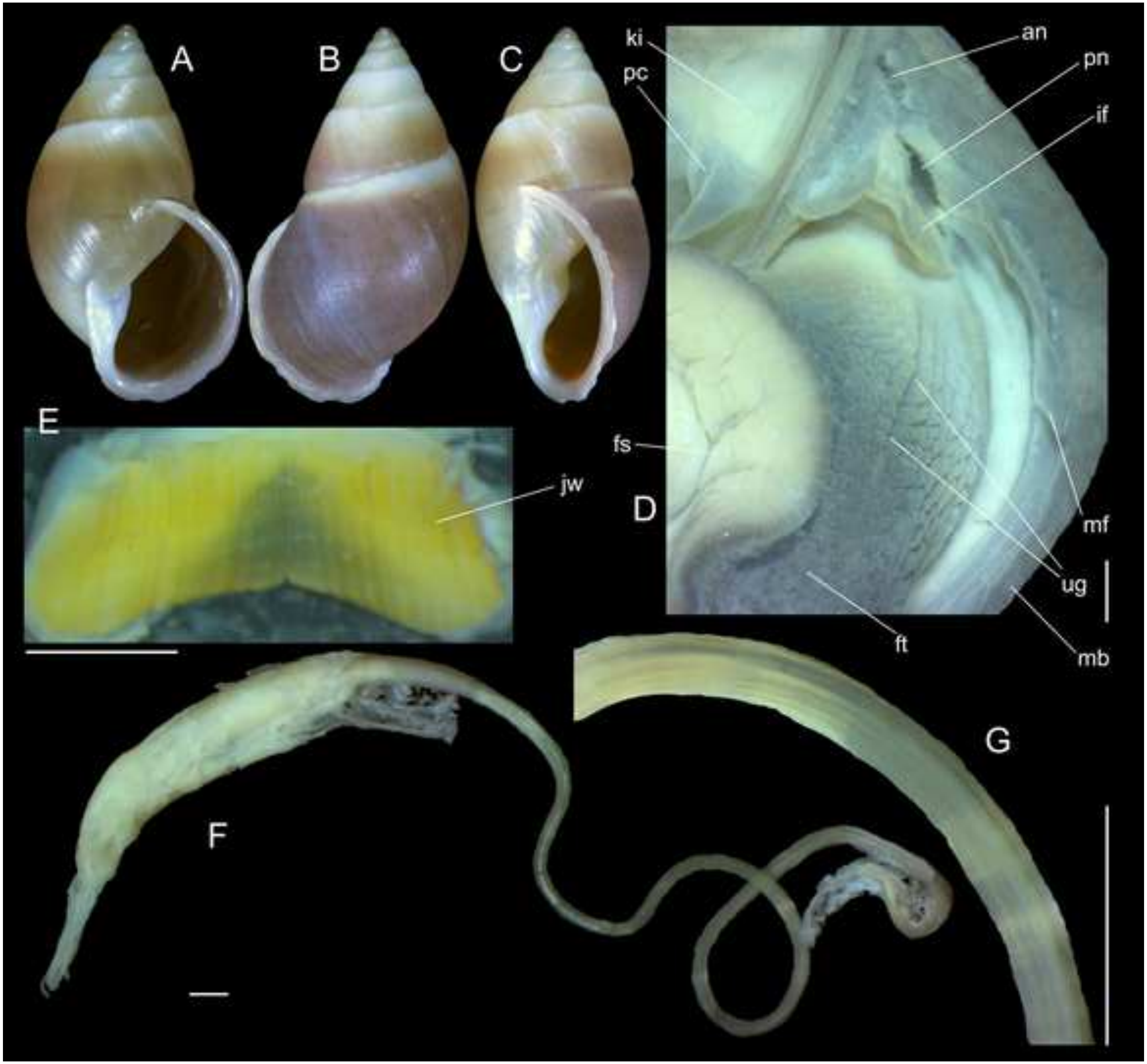

Click here to access/download;Figure;Kora 09b nigra.jpg

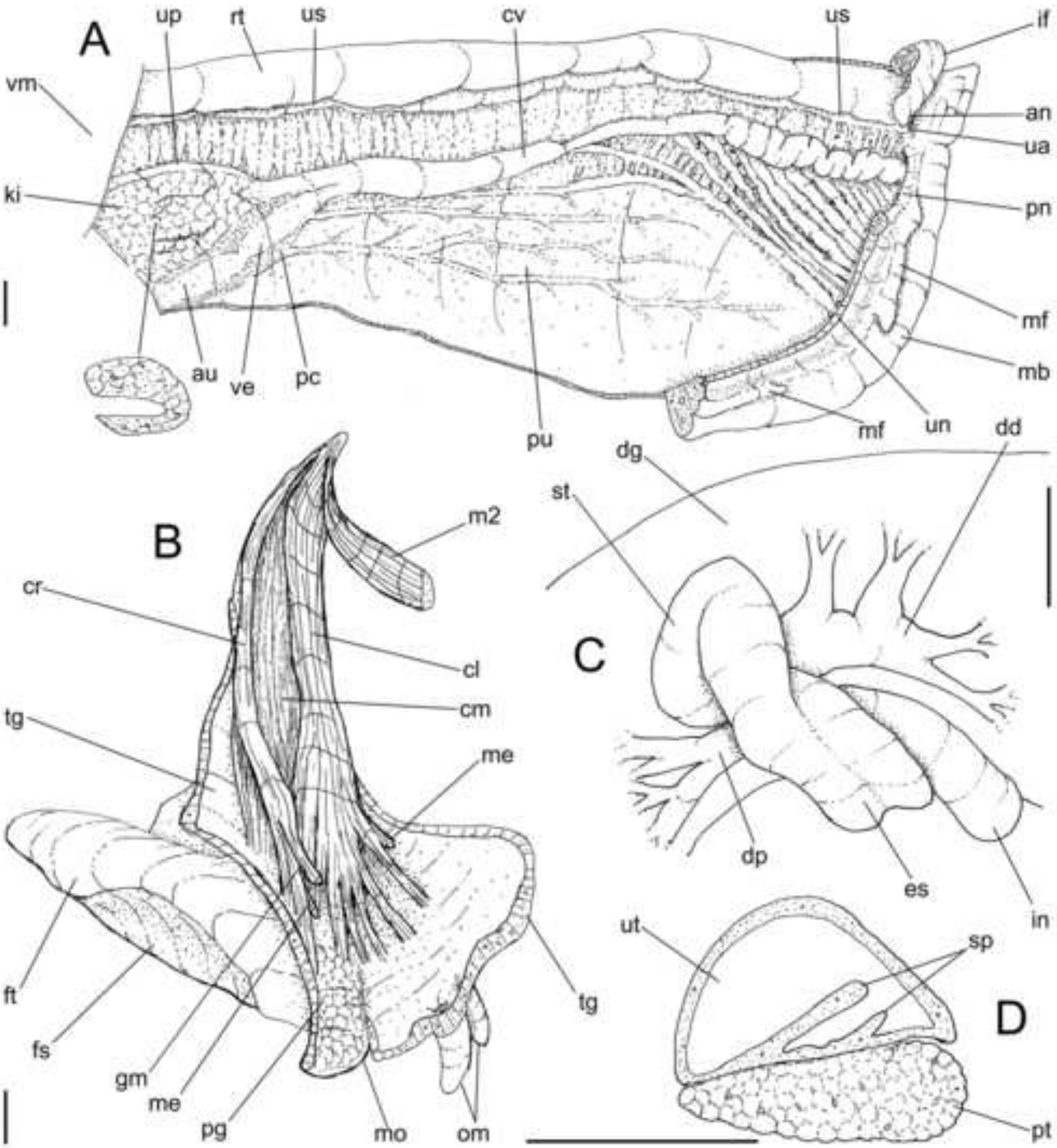

Figure 10 Click here to access/download;Figure;Kora 10b nigra.jpg

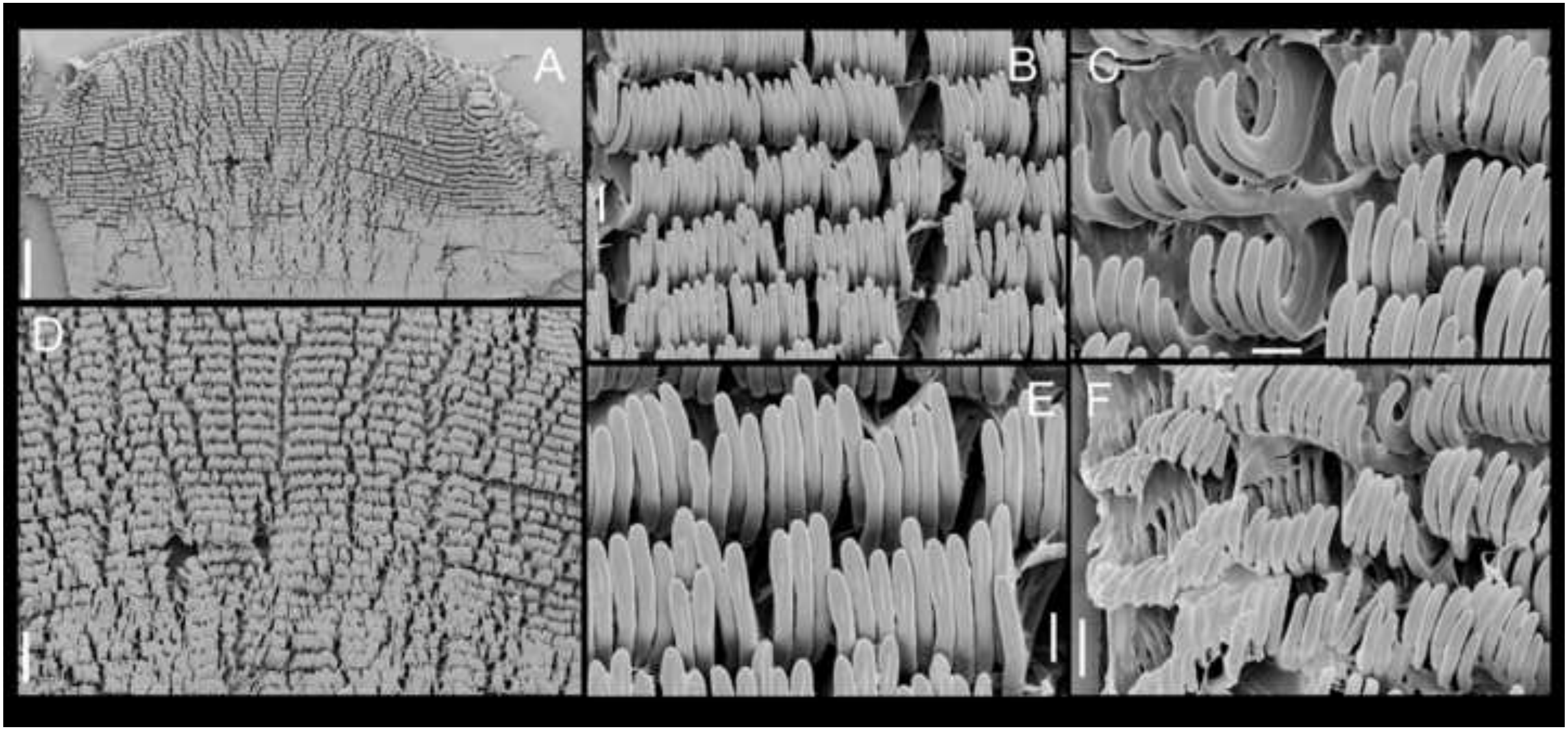

Click here to access/download;Figure;Kora 11b nigra.jpg

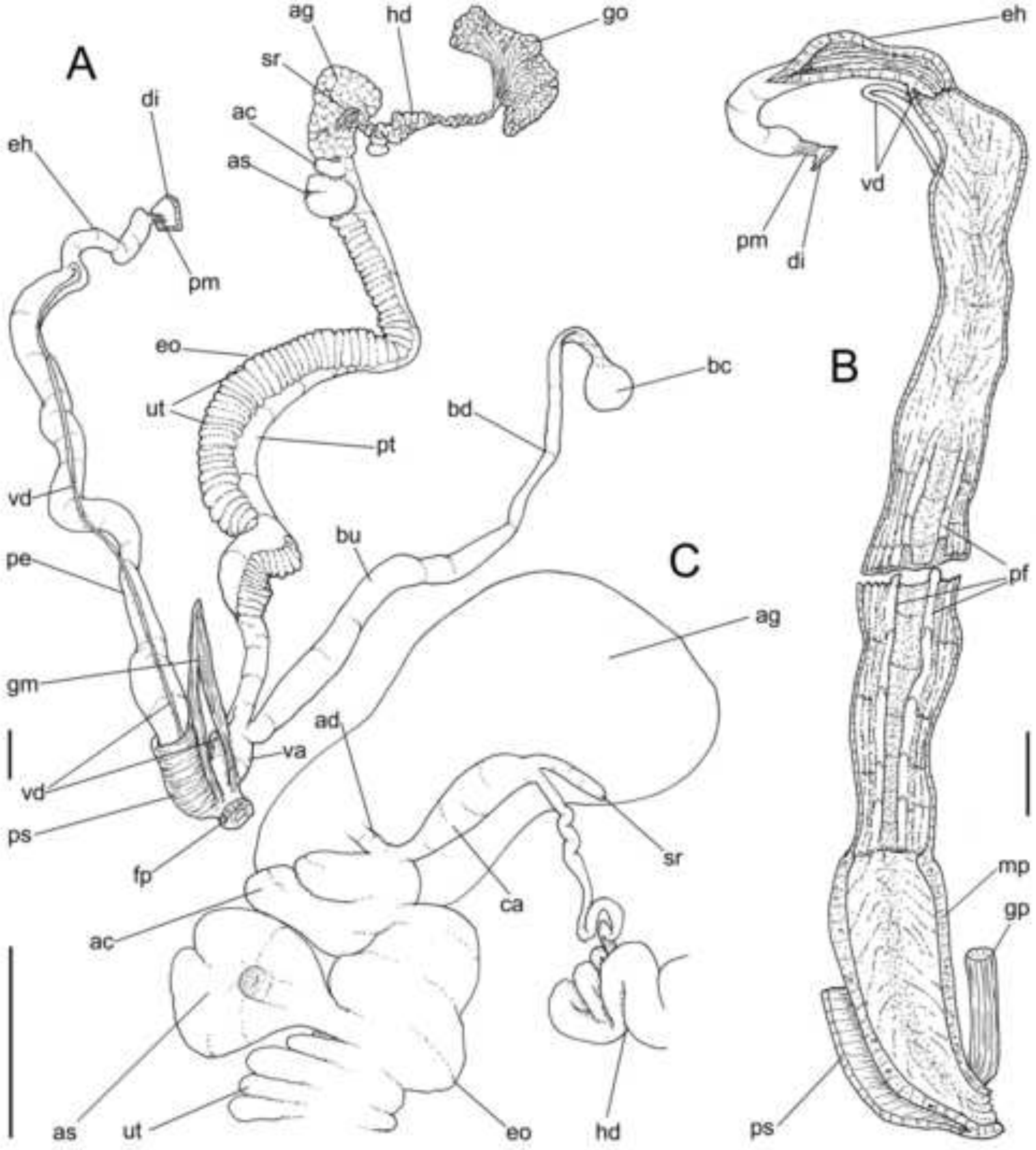

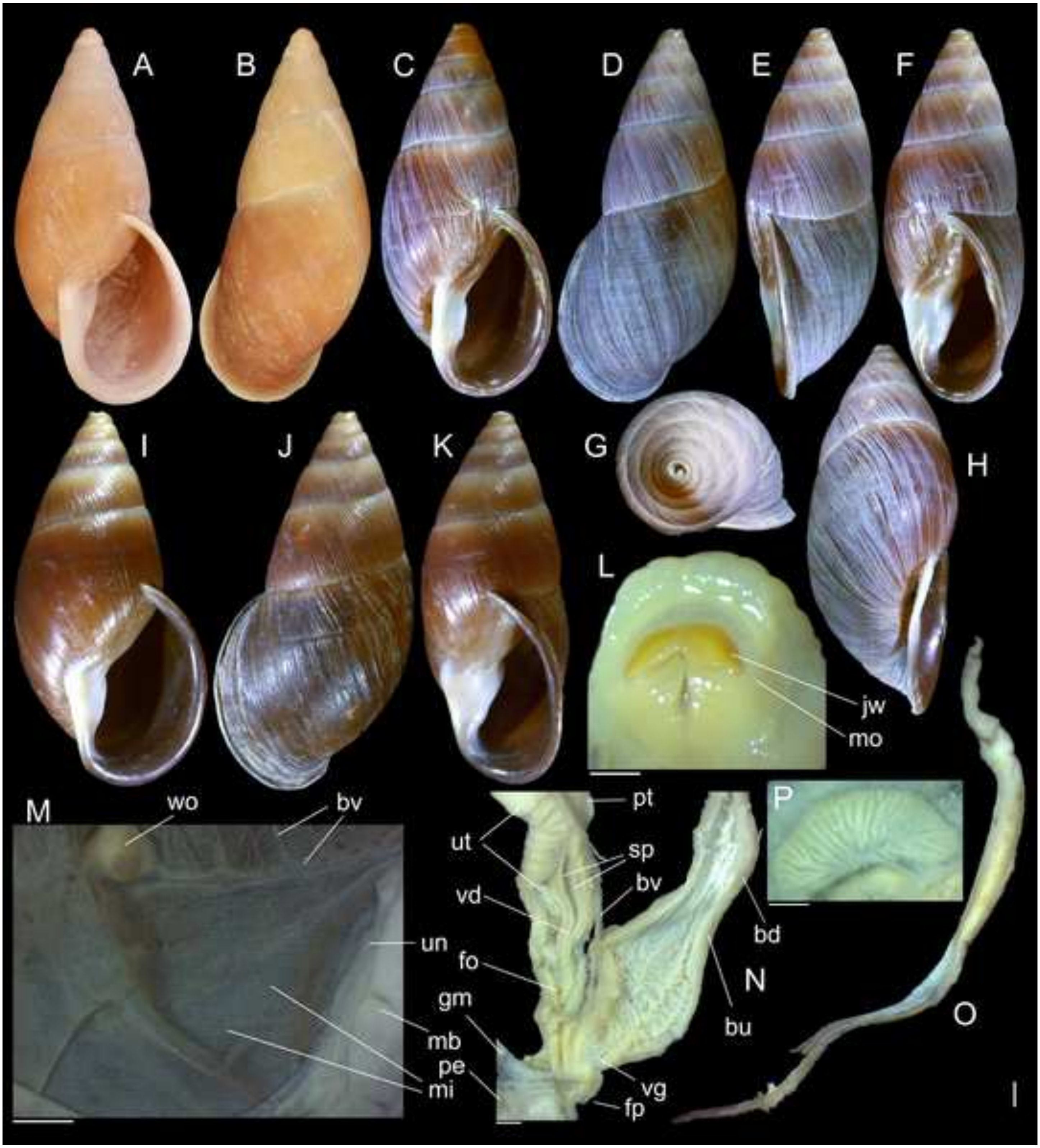
A
B
C
D
E
F
G
H
I
J
K
L
jw
mo
M
wo
bv
un
mb
mi
pt
ut
sp
bv
vd
fo
gm
pe
mi
vg
fp
bd
bu
N
P
O
I

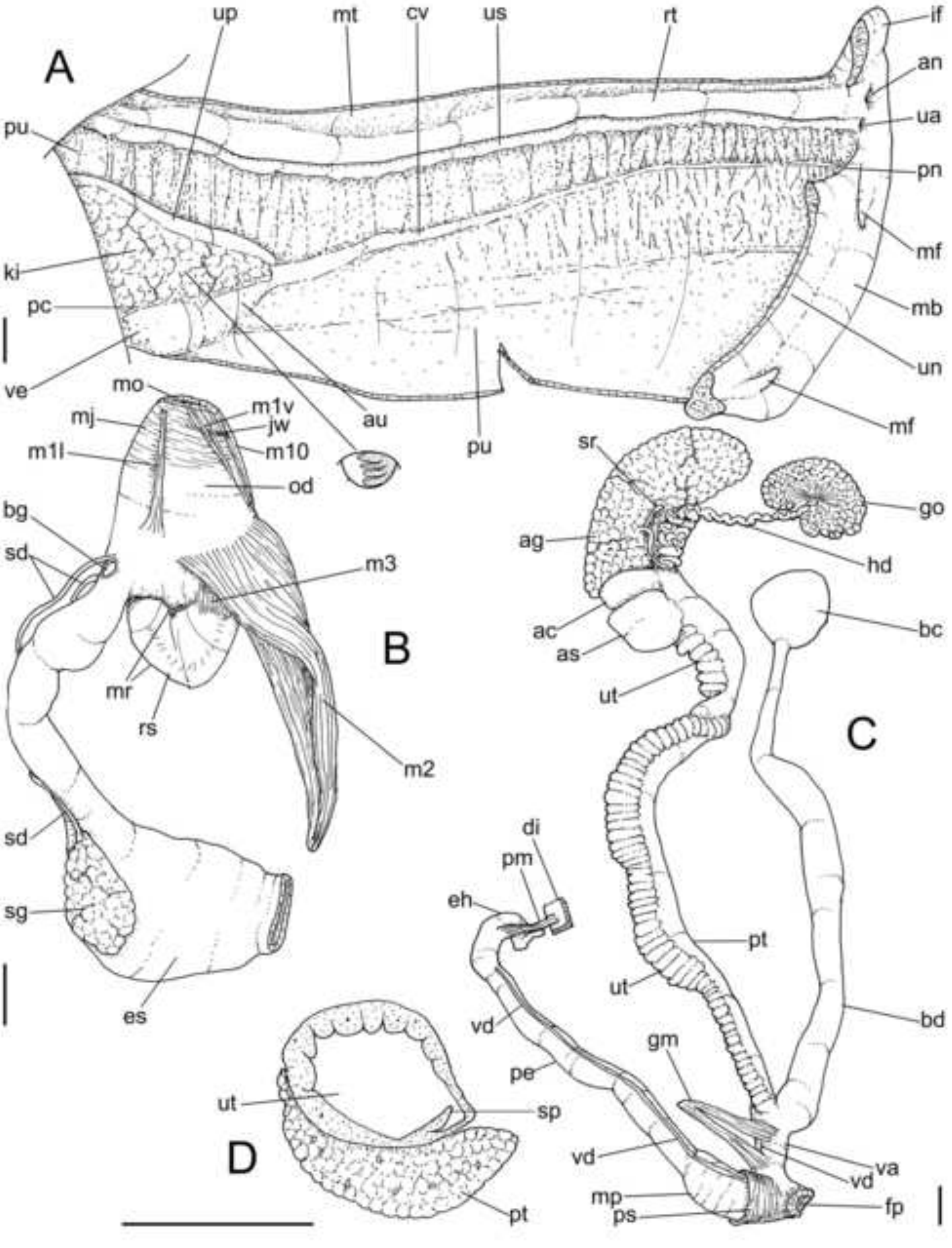
A
up
mt
cv
us
rt
if
an
ua
pn
mf
mb
un
mf
pu
ki
pc
ve
mo
au
pu
B
mj
m1v
jw
m10
m1l
od
bg
sd
m3
mr
rs
m2
sd
sg
es
C
sr
go
ag
hd
ac
as
ut
bc
di
pm
eh
pt
ut
vd
bd
gm
pe
ut
sp
D
vd
va
vd
mp
ps
fp
pt

Figure 14

Click here to access/download;Figure;Kora 14b rupestris.jpg

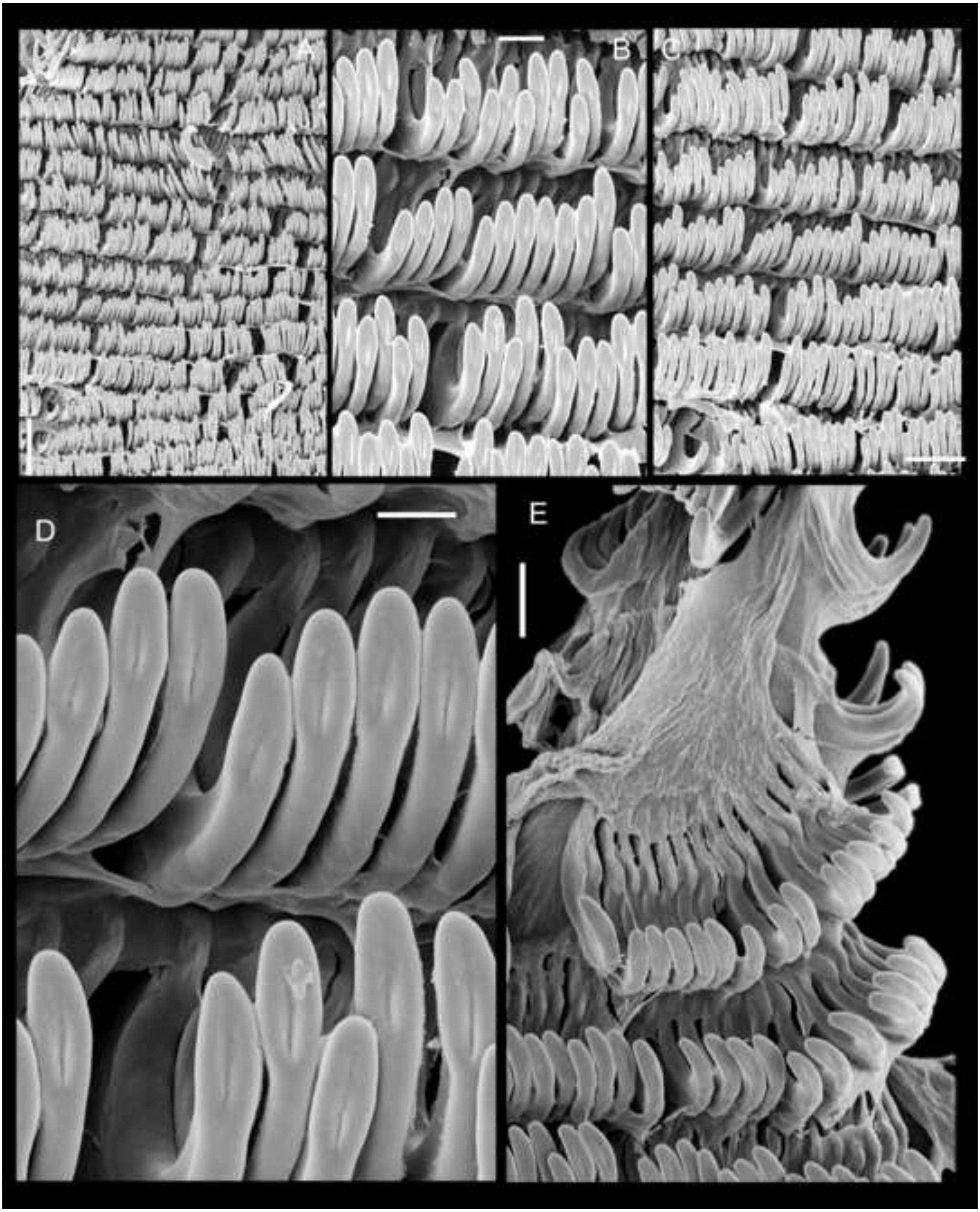

Figure 15

Click here to access/download;Figure;Kora 15b rupestris.jpg

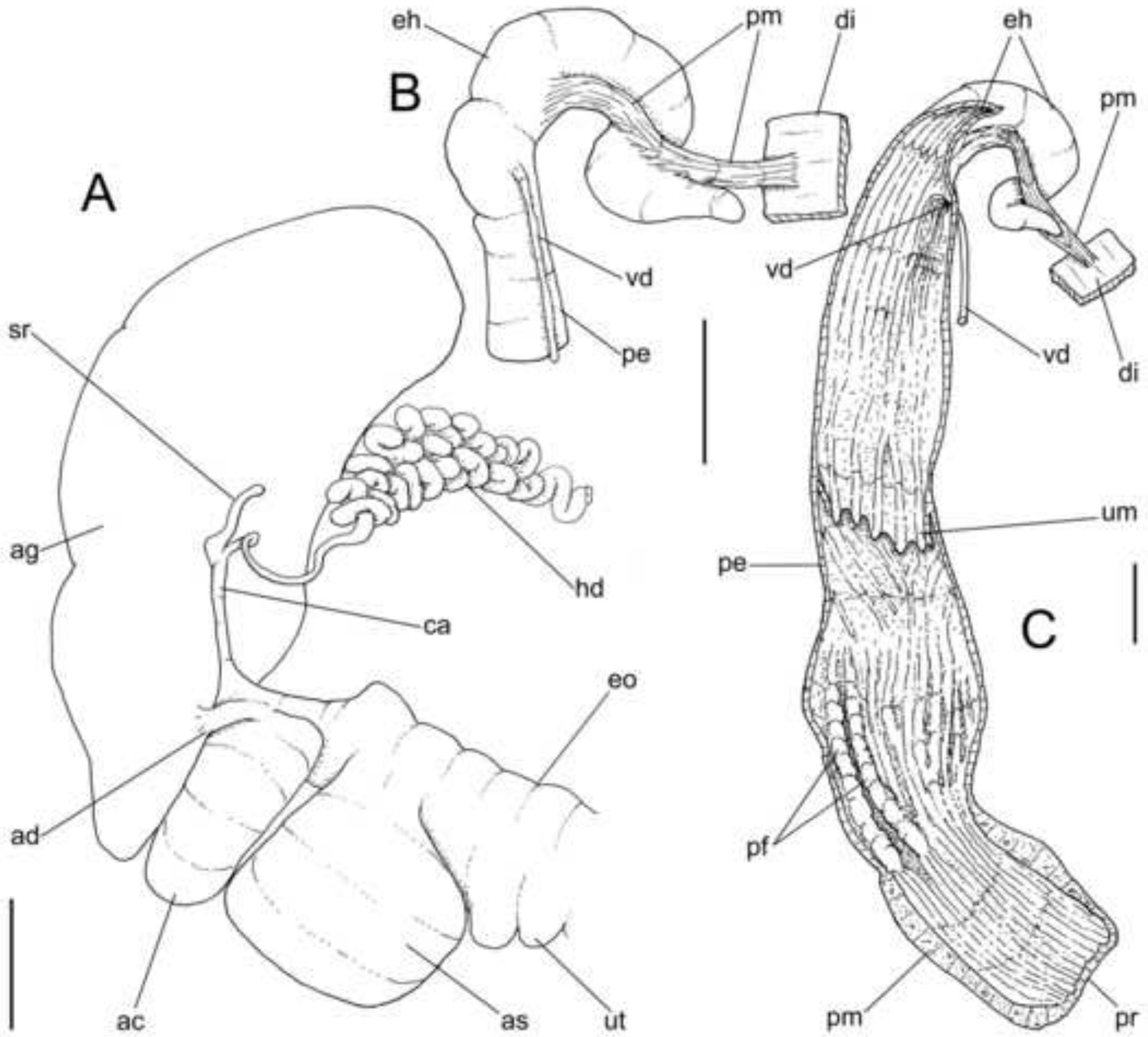

Figure 16

Click here to access/download;Figure;Kora 16b tupan.jpg

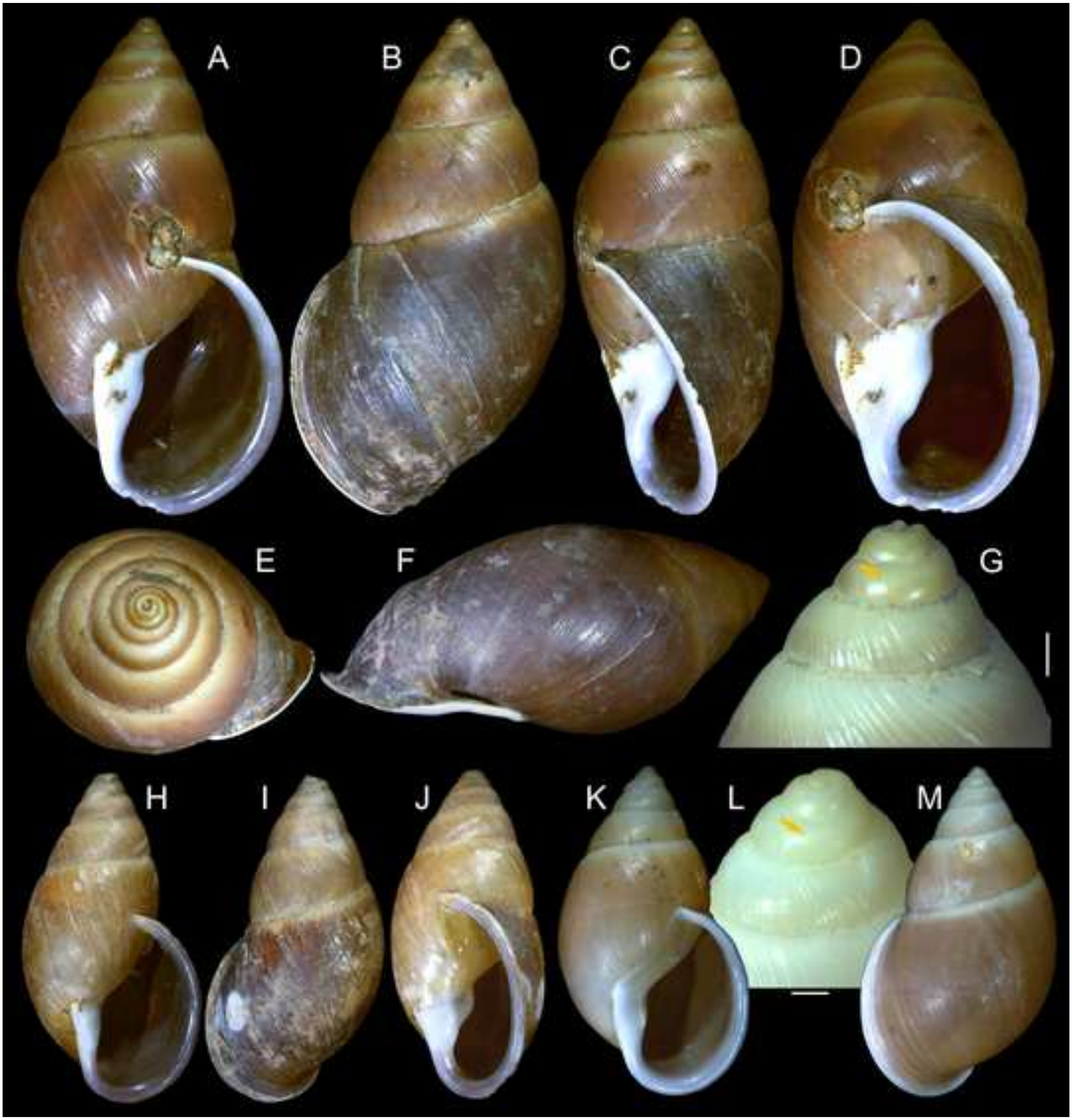

Figure 17

Click here to access/download;Figure;Kora 17b tupan.jpg

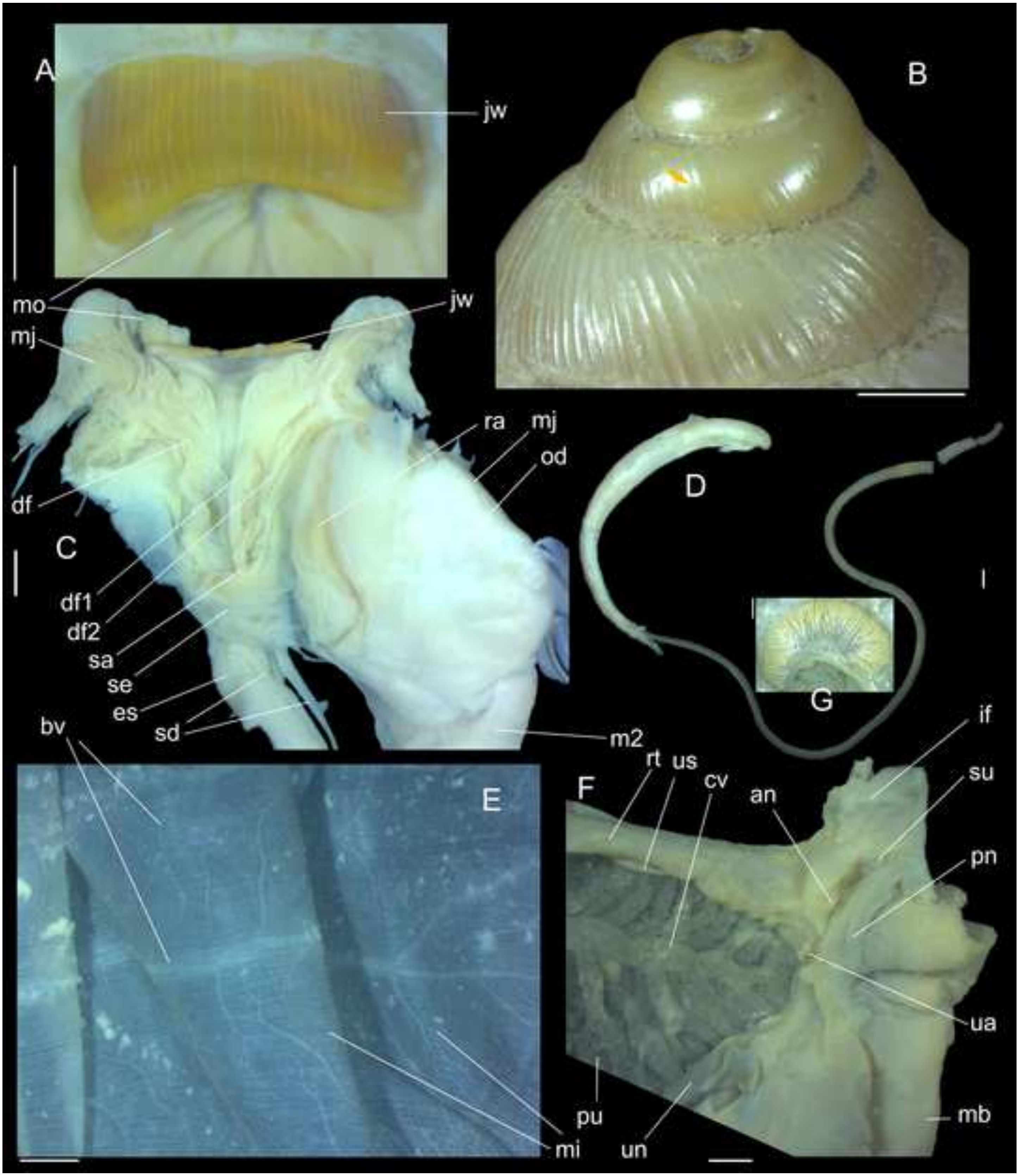

Figure 18

Click here to access/download;Figure;Kora 18b tupan.jpg

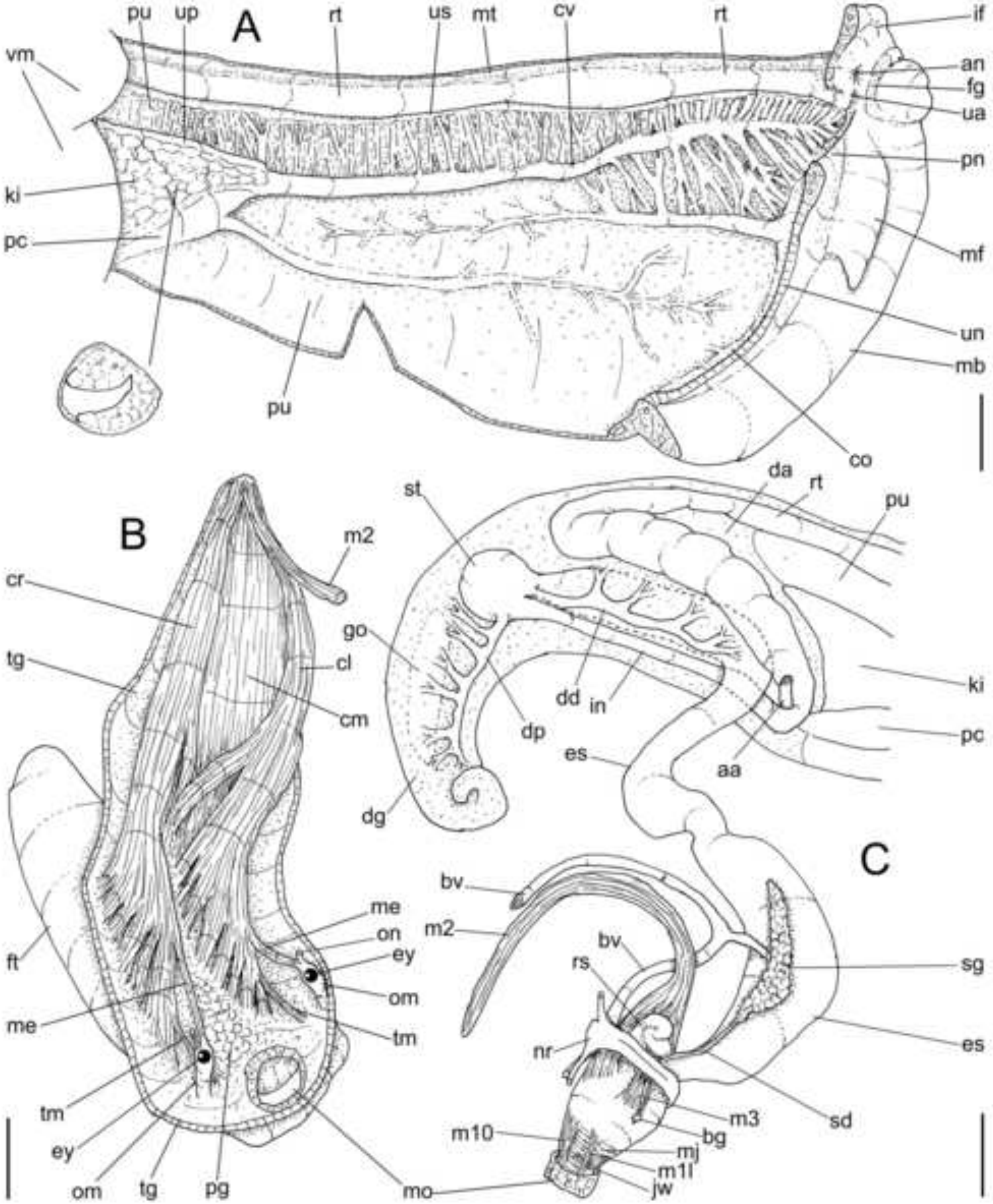

 Click here to access/download;Figure;Kora 19b tupan.jpg

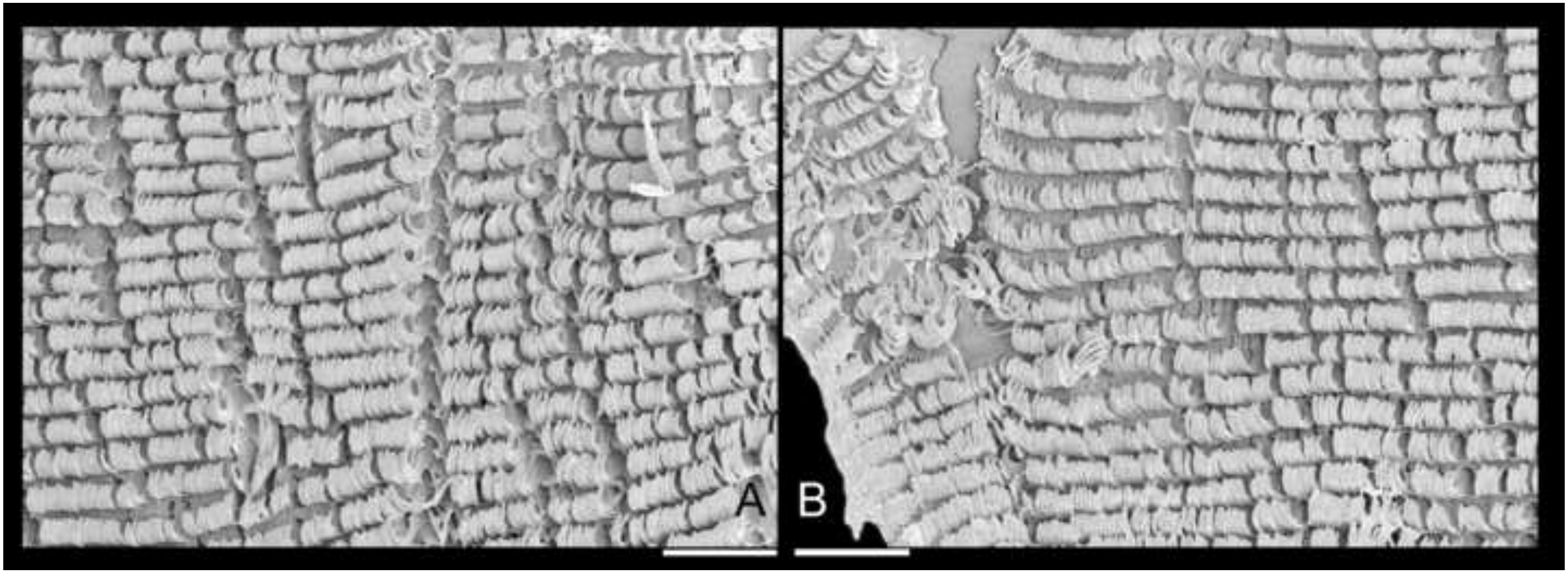

 Click here to access/download;Figure;Kora 20b tupan.jpg

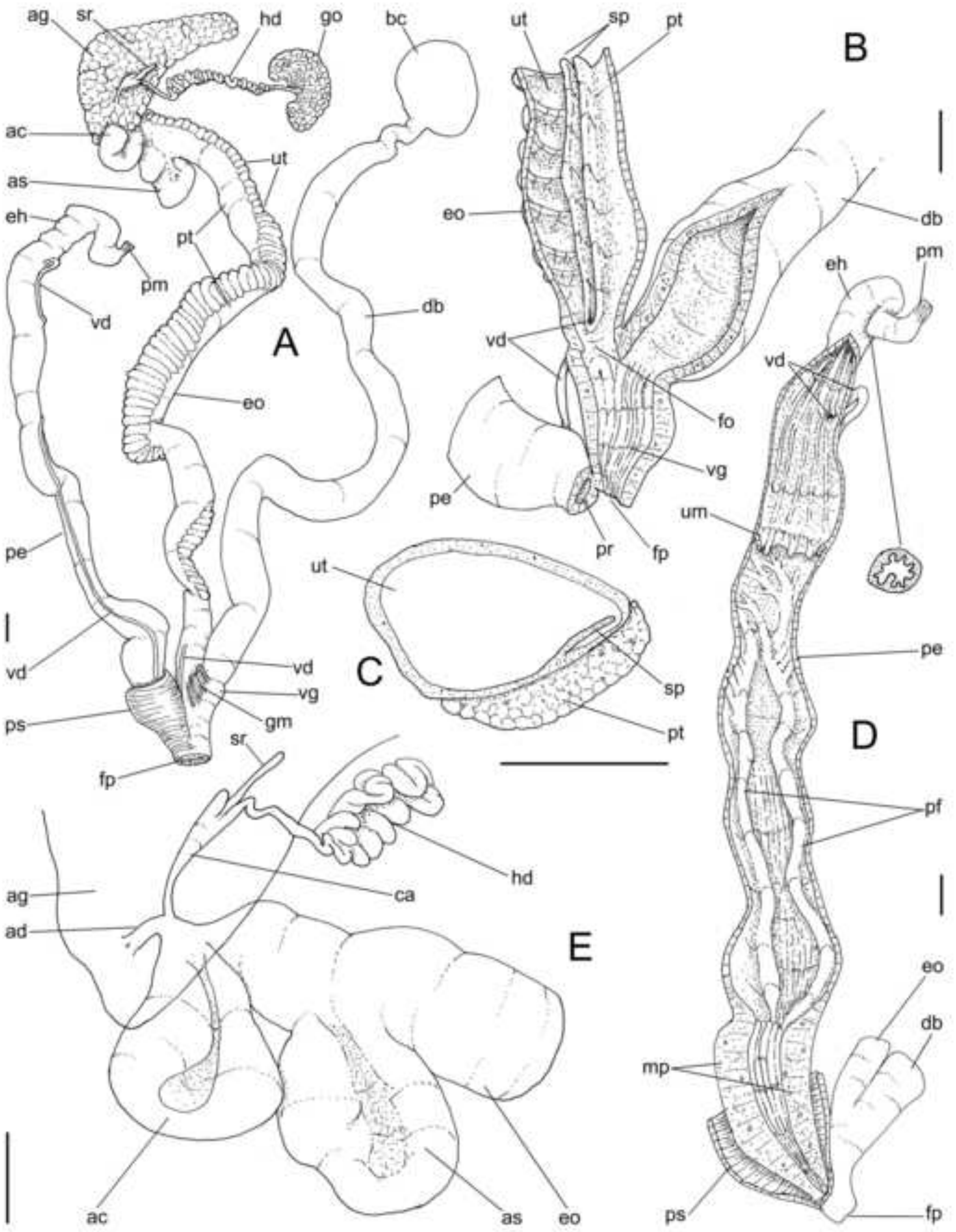

Figure 21 Click here to access/download;Figure;Kora 21b ajar.jpg

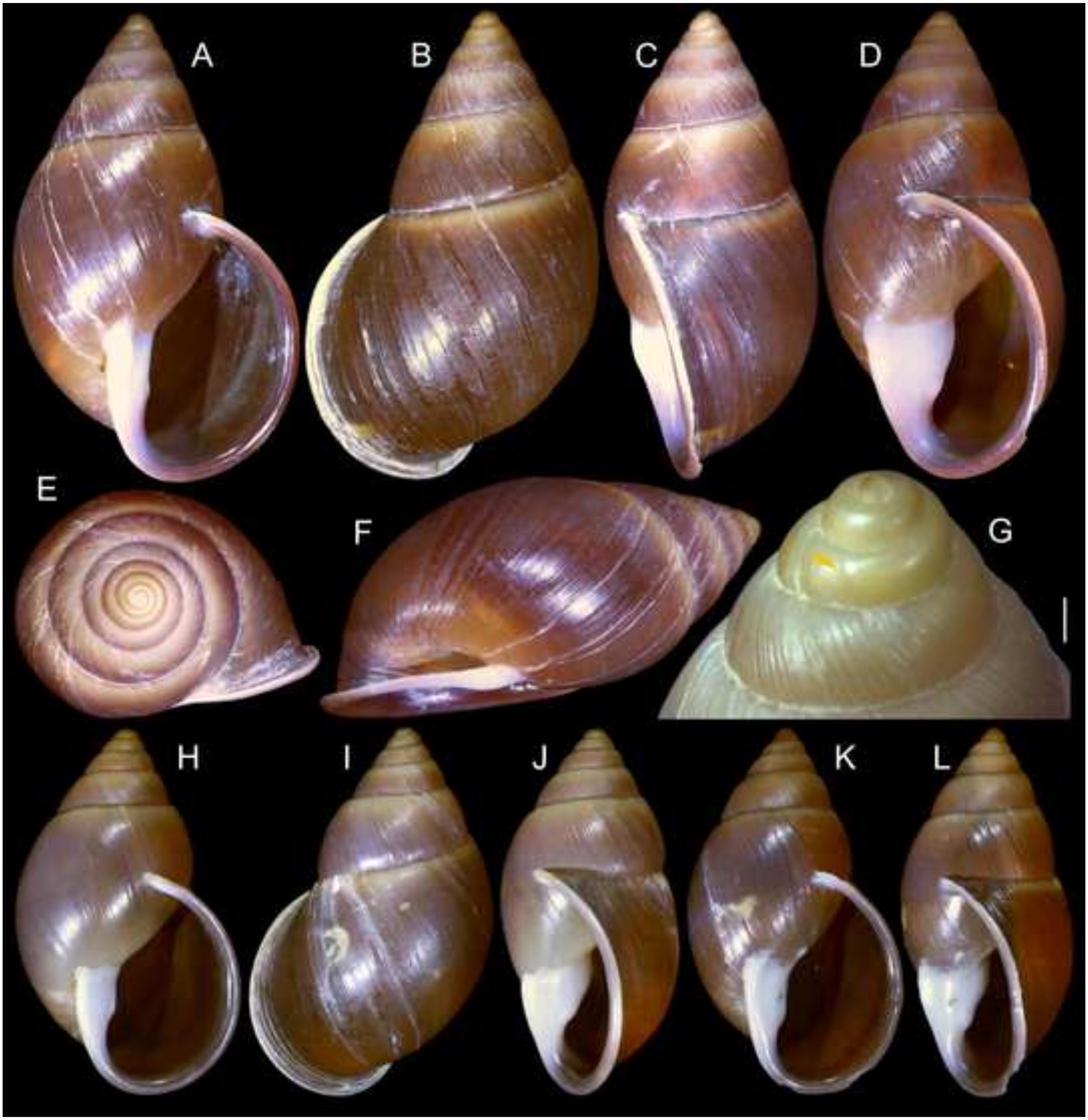

Figure 22

Click here to access/download;Figure;Kora 22b ajar.jpg

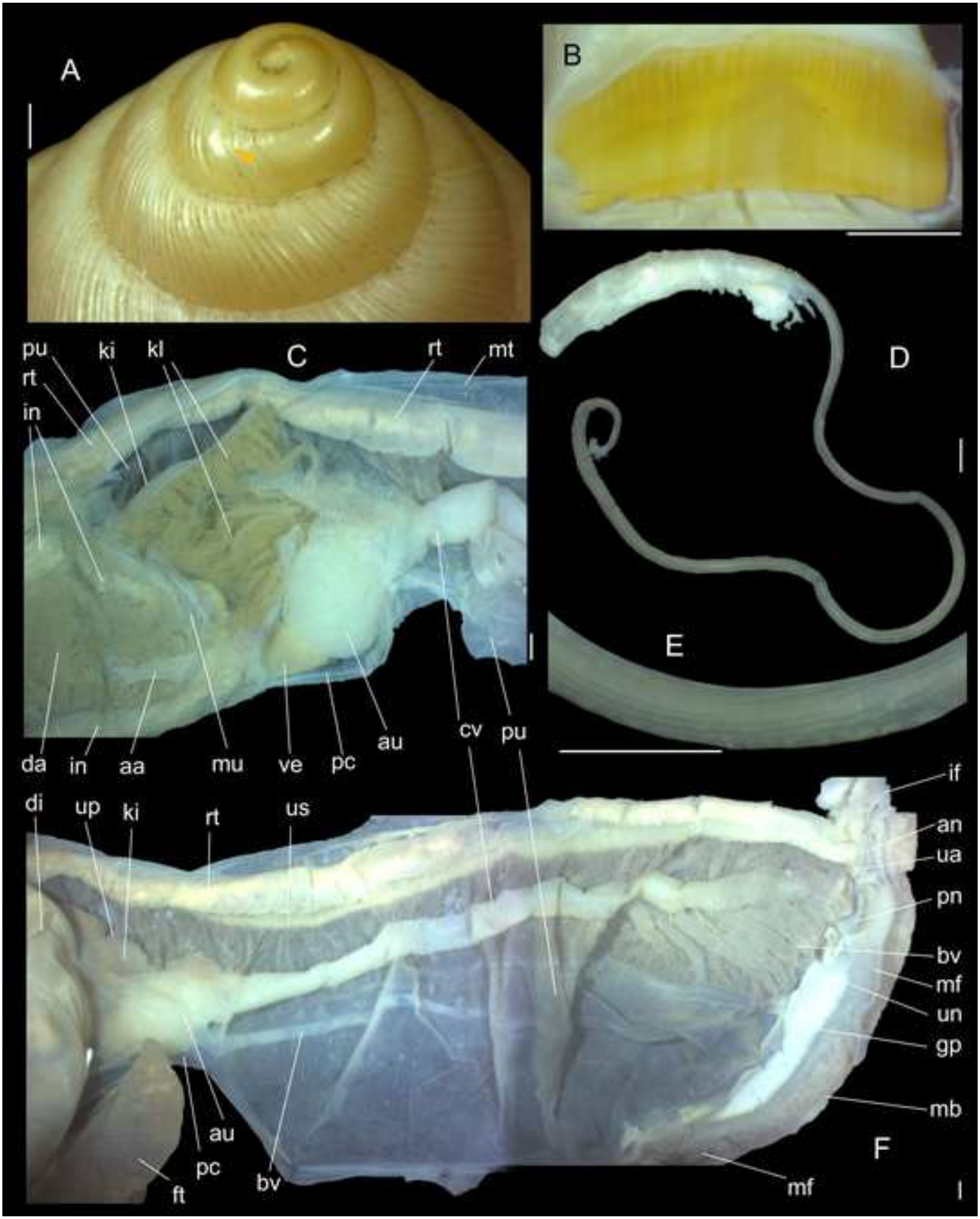

Figure 23

Click here to access/download;Figure;Kora 23b ajar.jpg

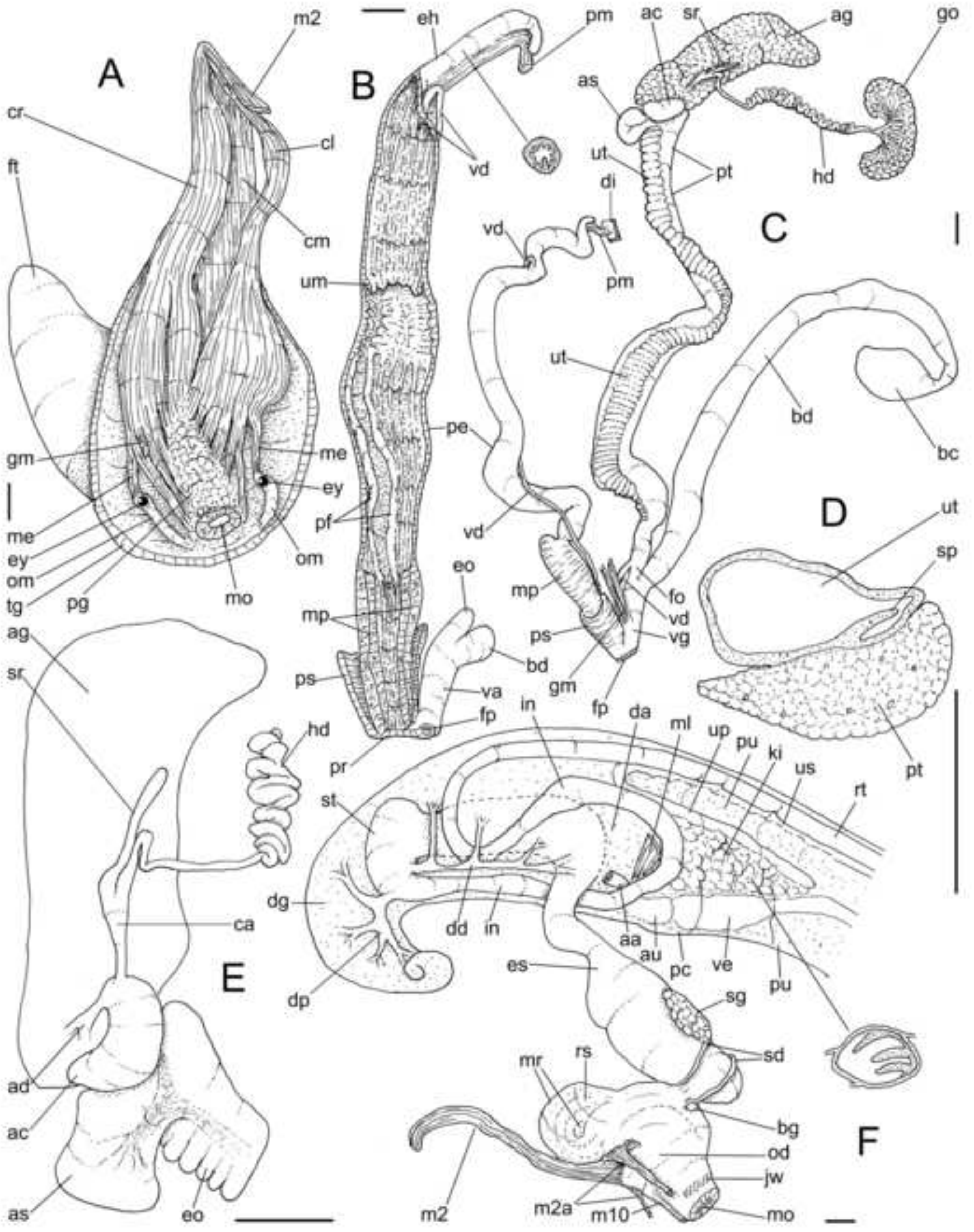

 Click here to access/download;Figure;Kora 24b ajar.jpg

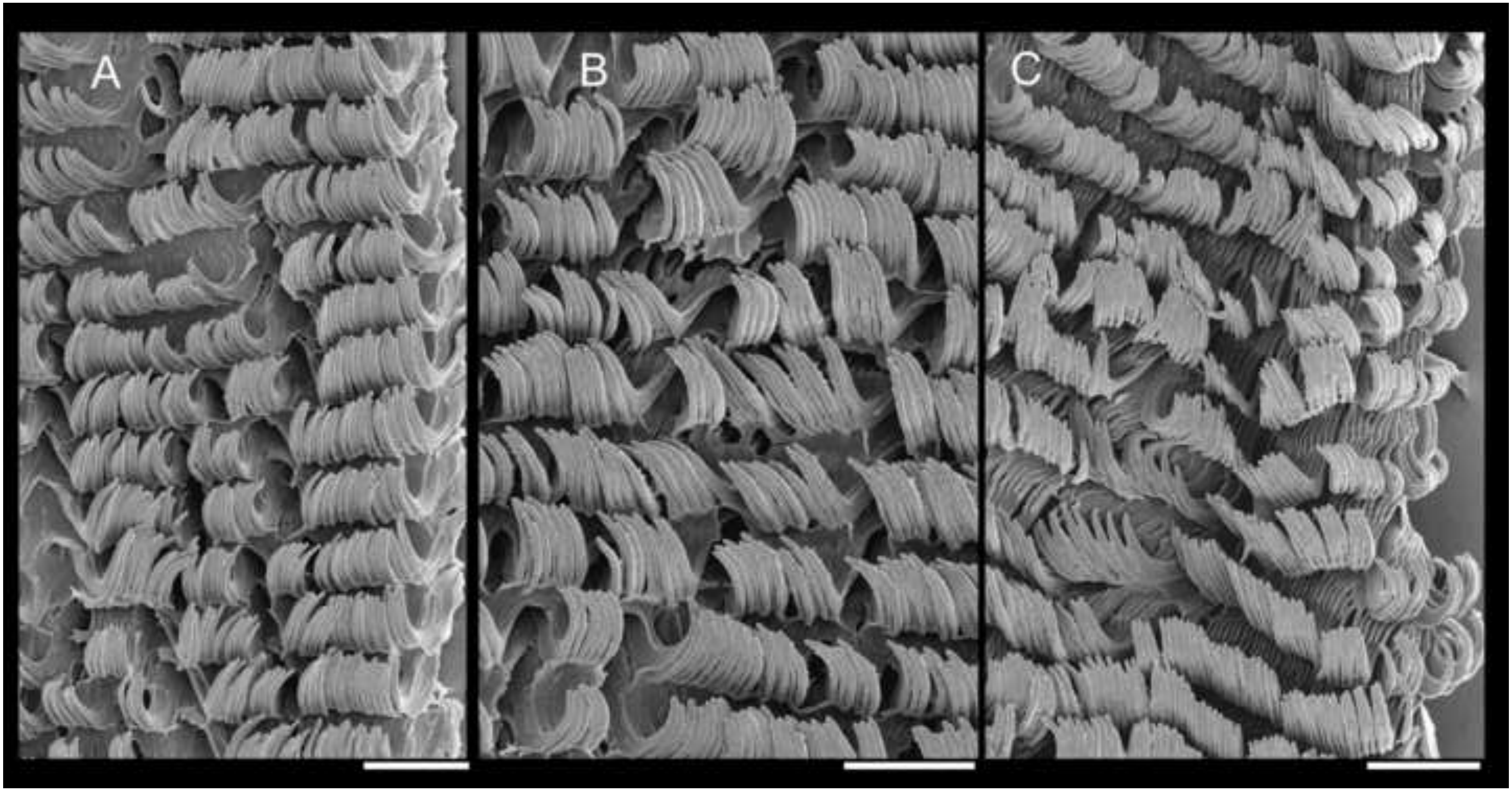

Figure 25

Click here to access/download;Figure;Kora 25b aetheria.jpg

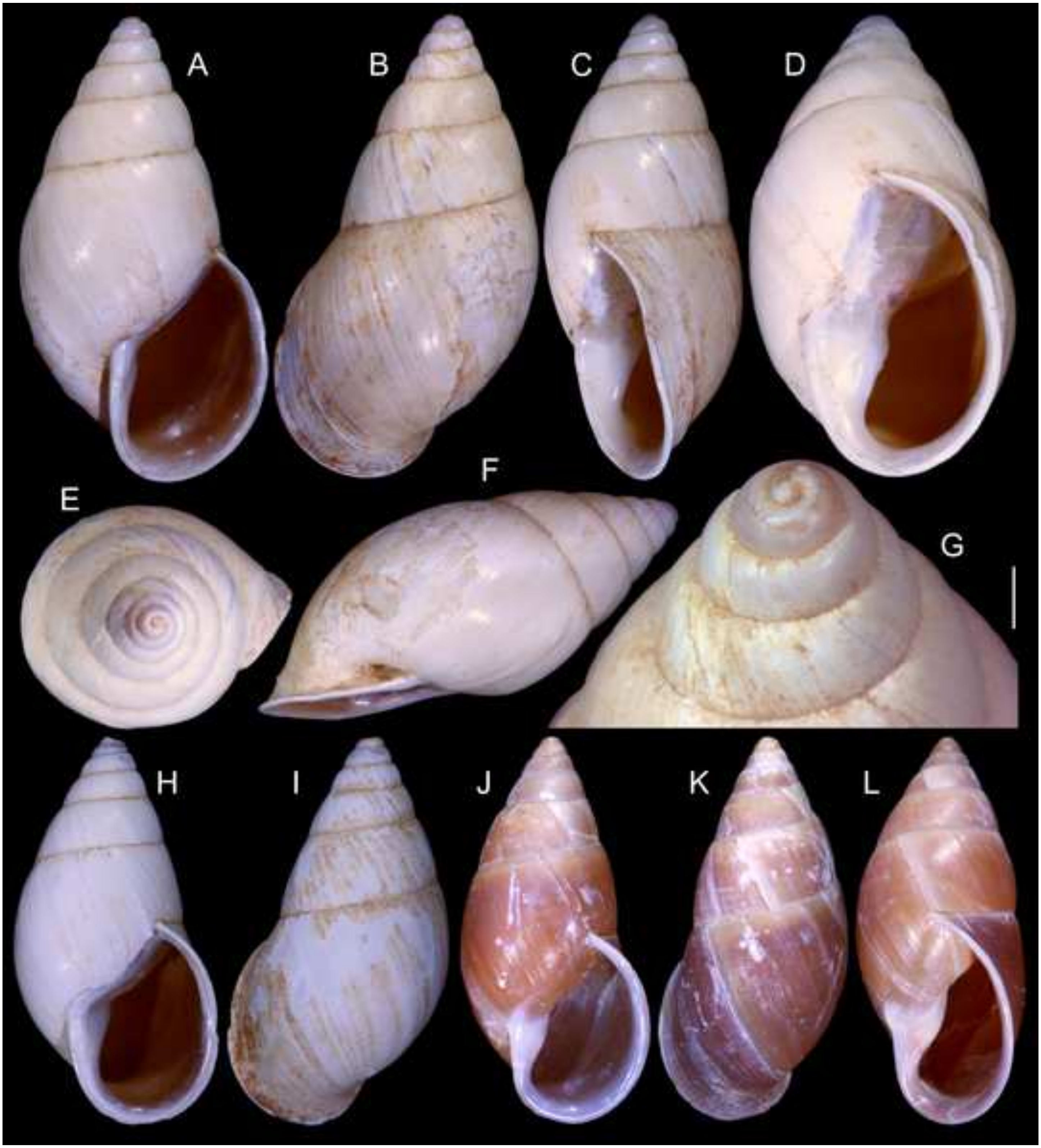

Figure 26

Click here to access/download;Figure;Kora 26b aetheria.jpg

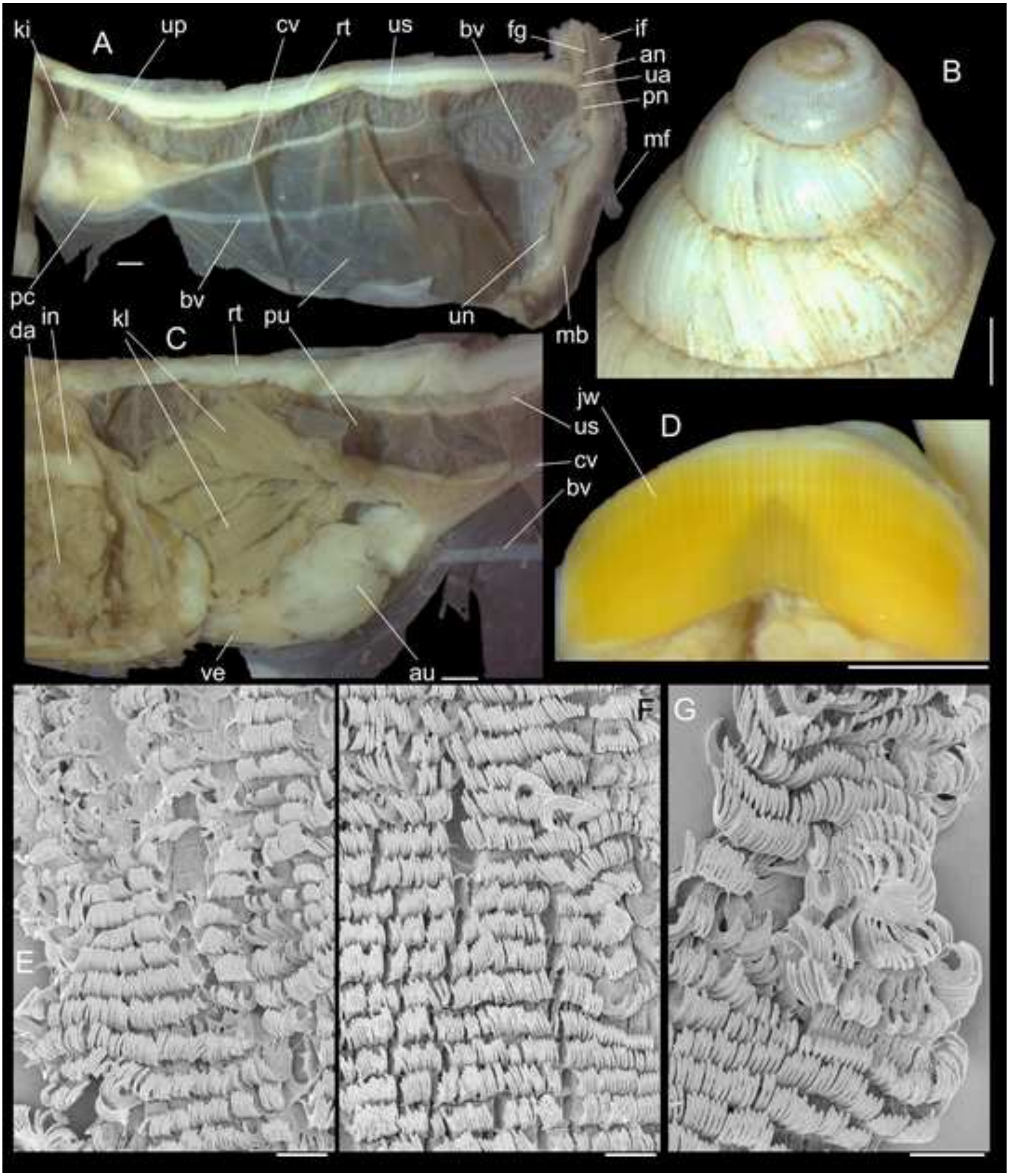

Figure 27 Click here to access/download;Figure;Kora 27b aetheria.jpg

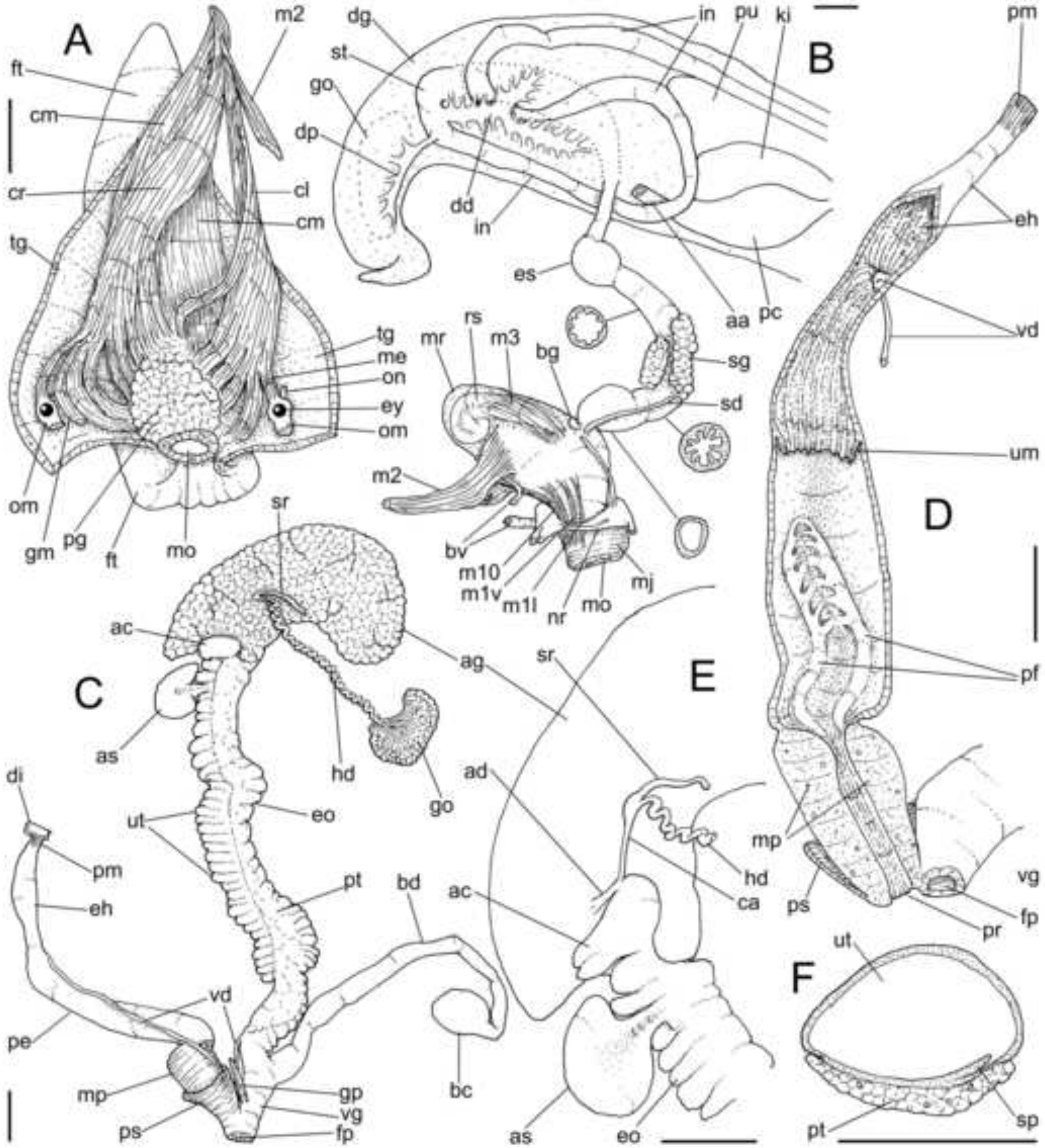

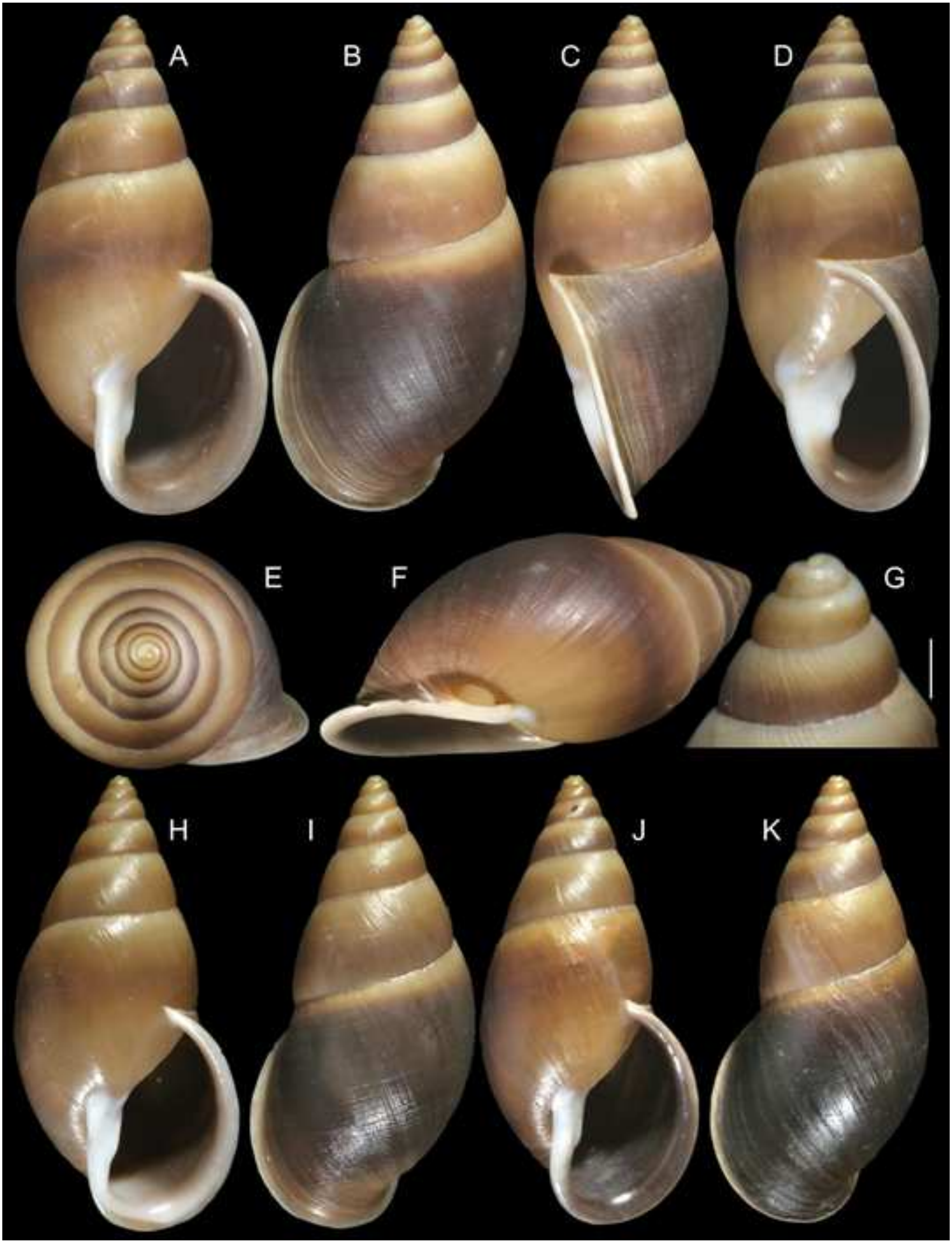
A
B
C
D
E
F
G
H
I
J
K

Figure 29

Click here to access/download;Figure;Kora 29b jimenezi.jpg

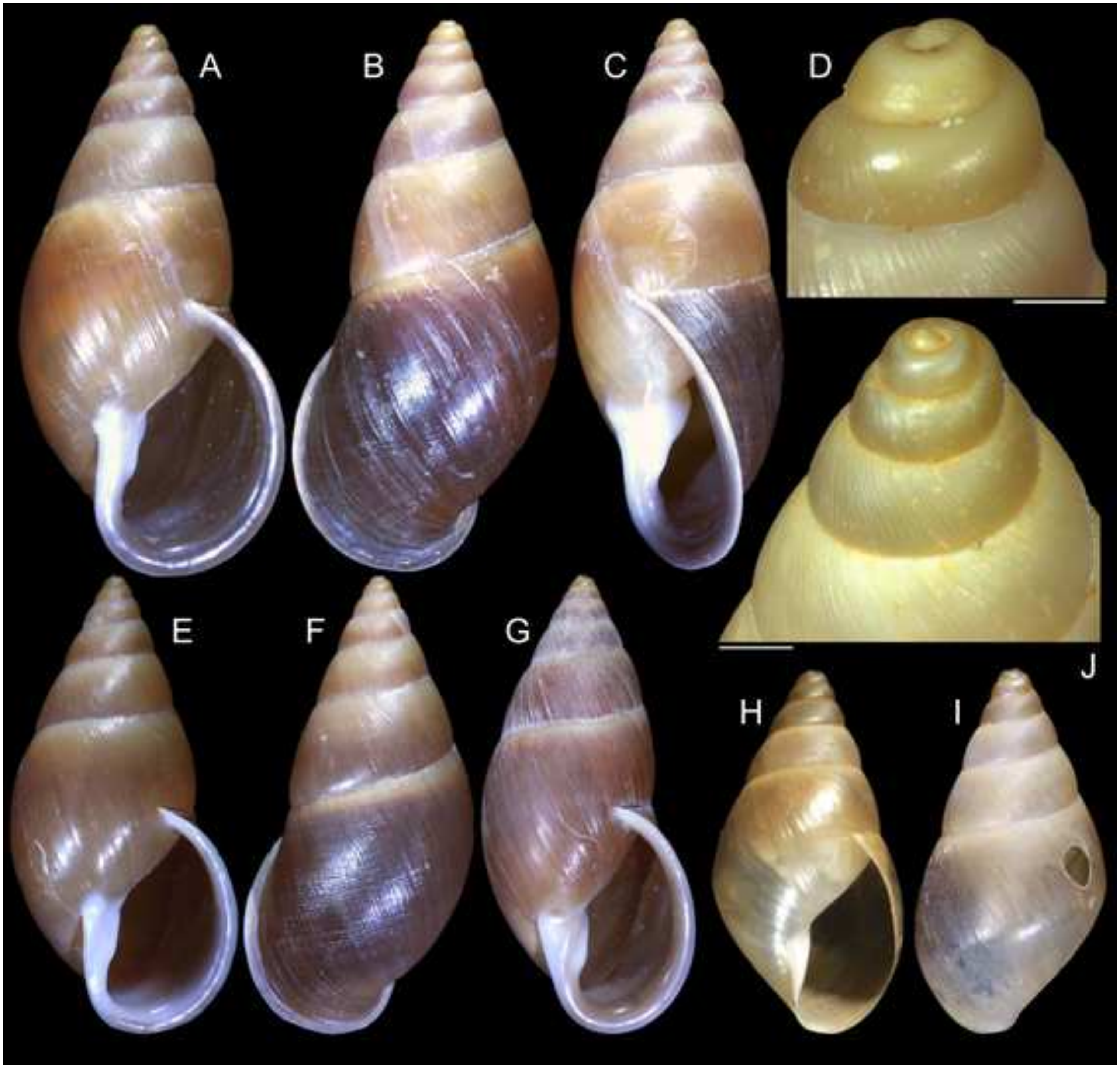

Figure 30

Click here to access/download;Figure;Kora 30b jimenezi.jpg

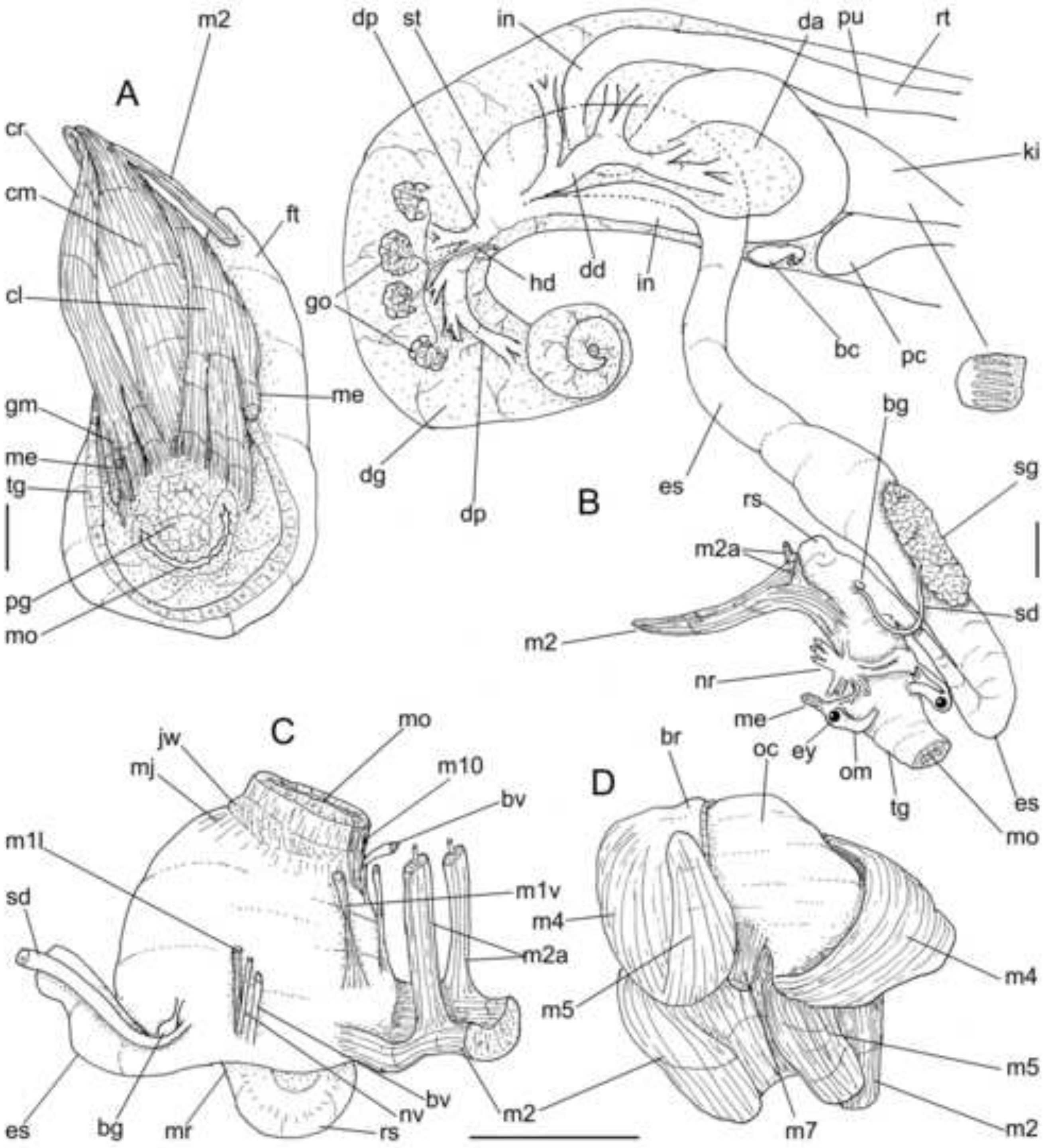

Figure 31 Click here to access/download;Figure;Kora 31b jimenezi.jpg

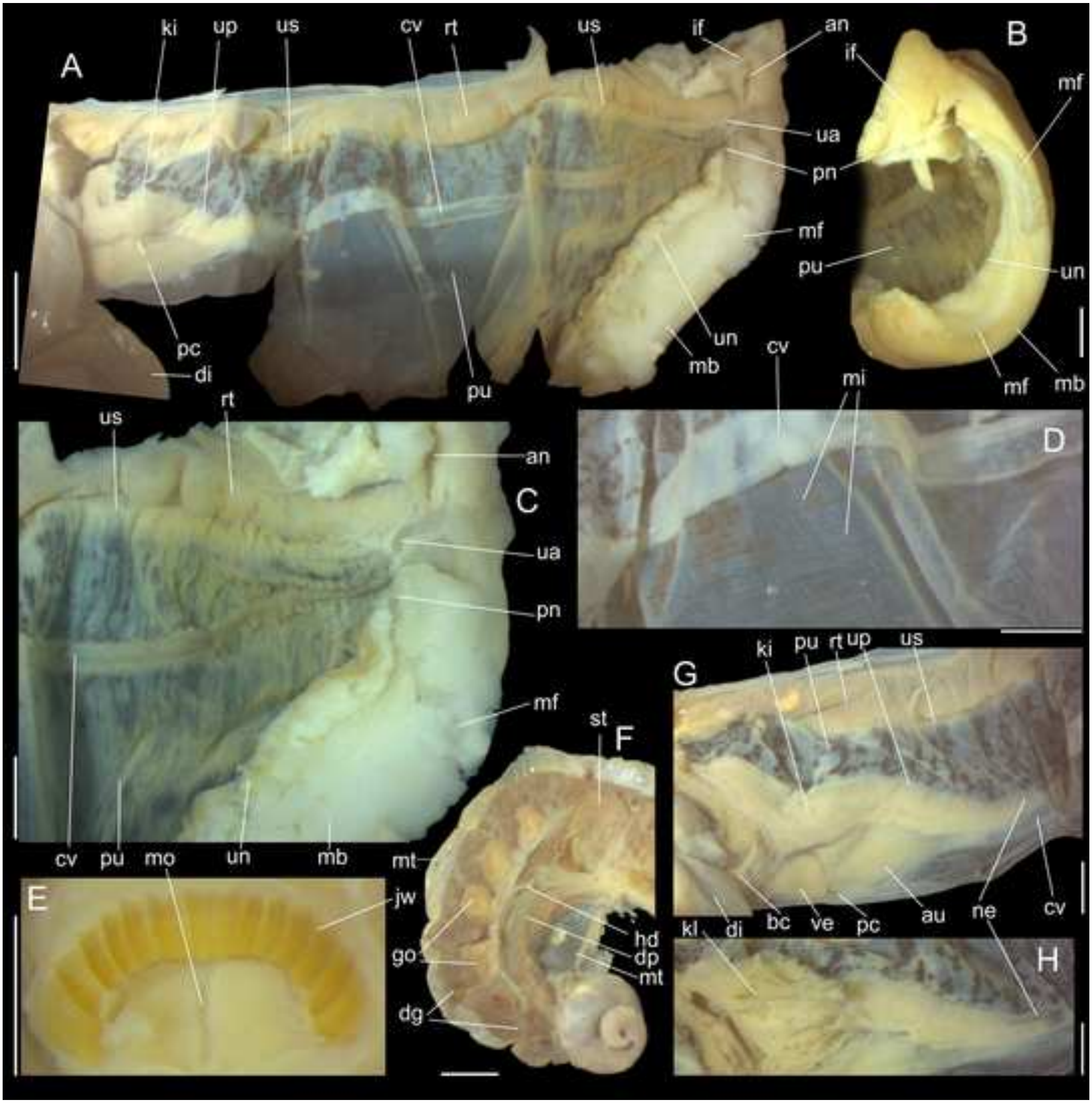

Figure 32 Click here to access/download;Figure;Kora 32b jimenezi.jpg

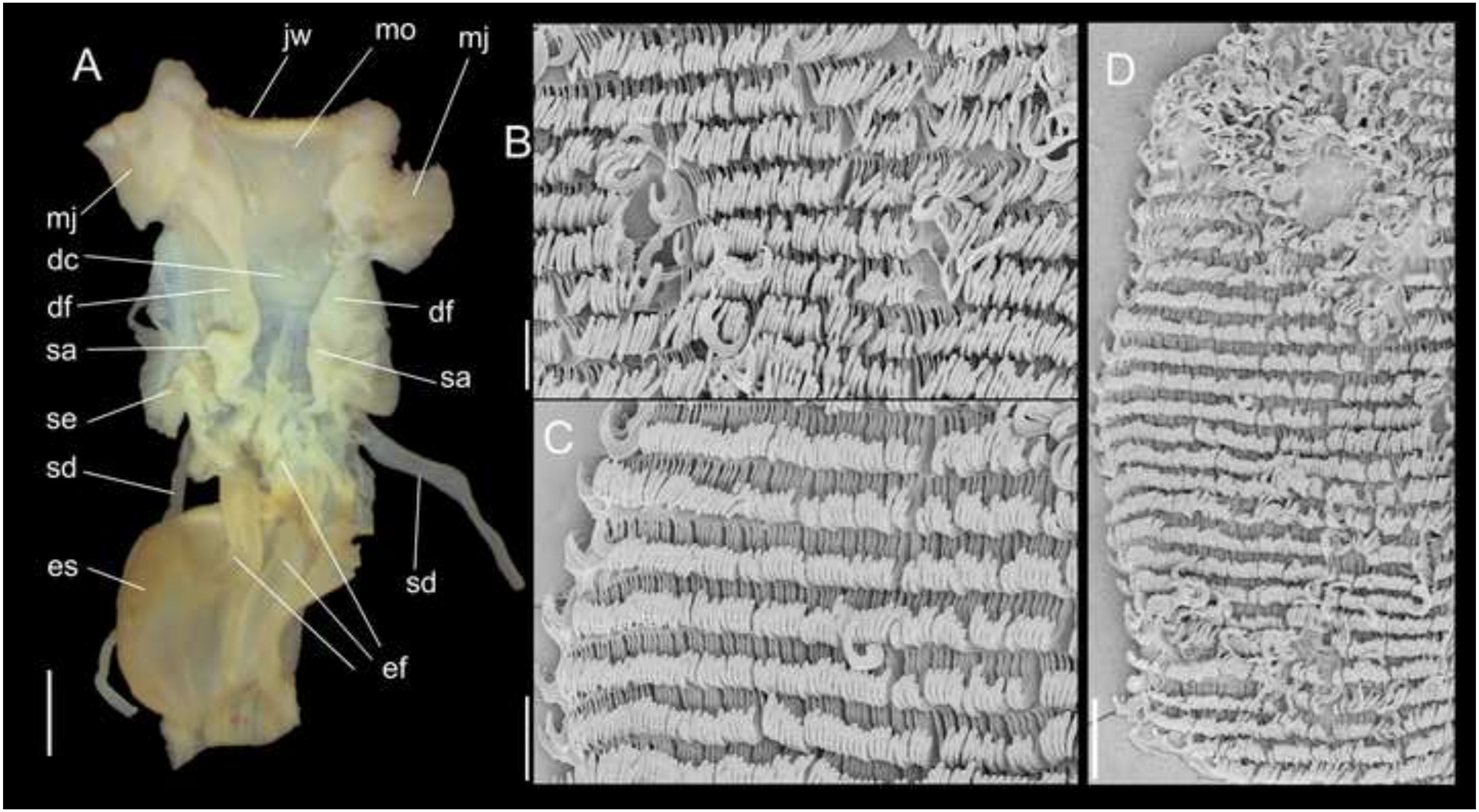

Figure 33

Click here to access/download;Figure;Kora 33b jimenezi.jpg

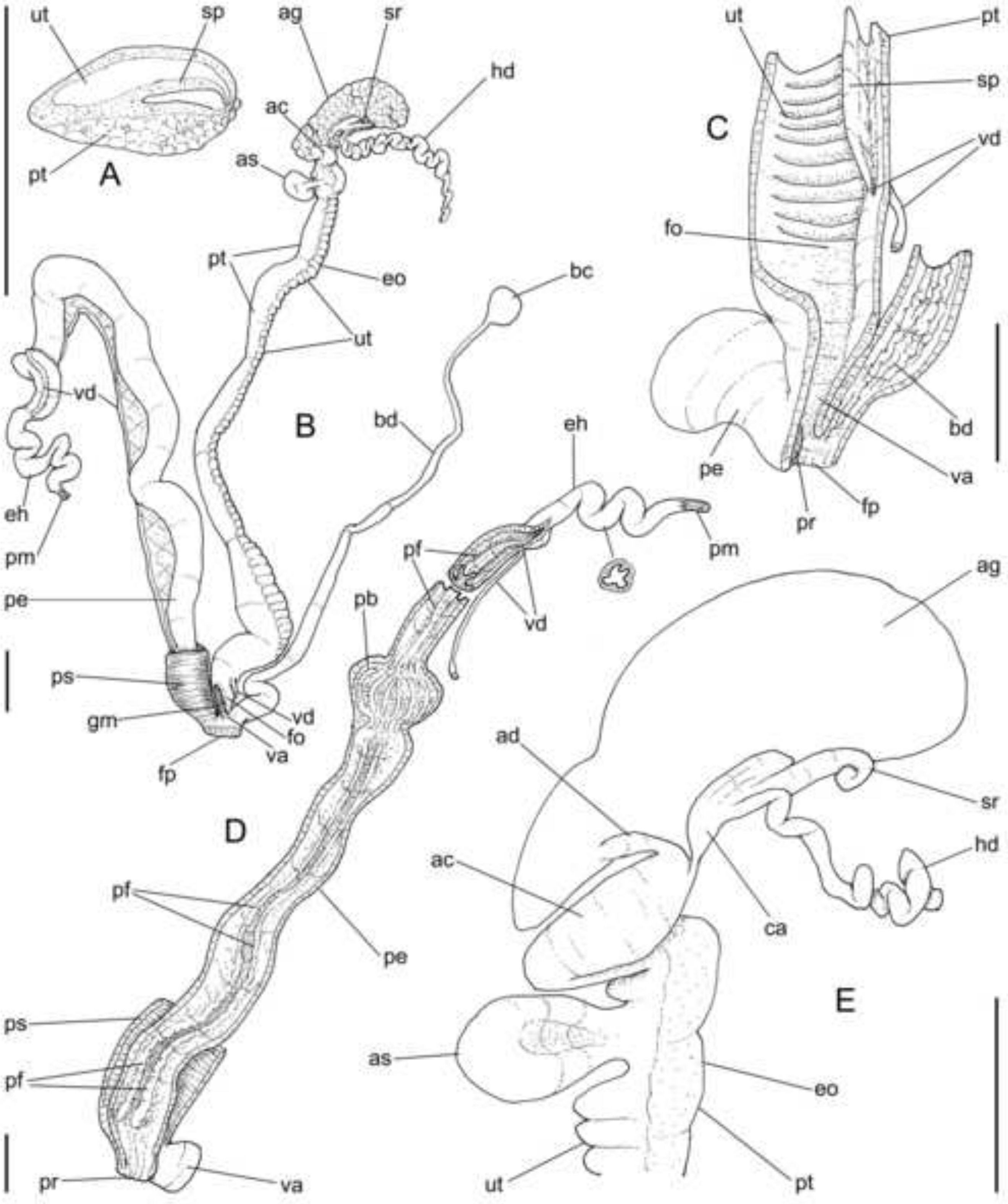

Figure 34

Click here to access/download;Figure;Kora 34b uhlei.jpg

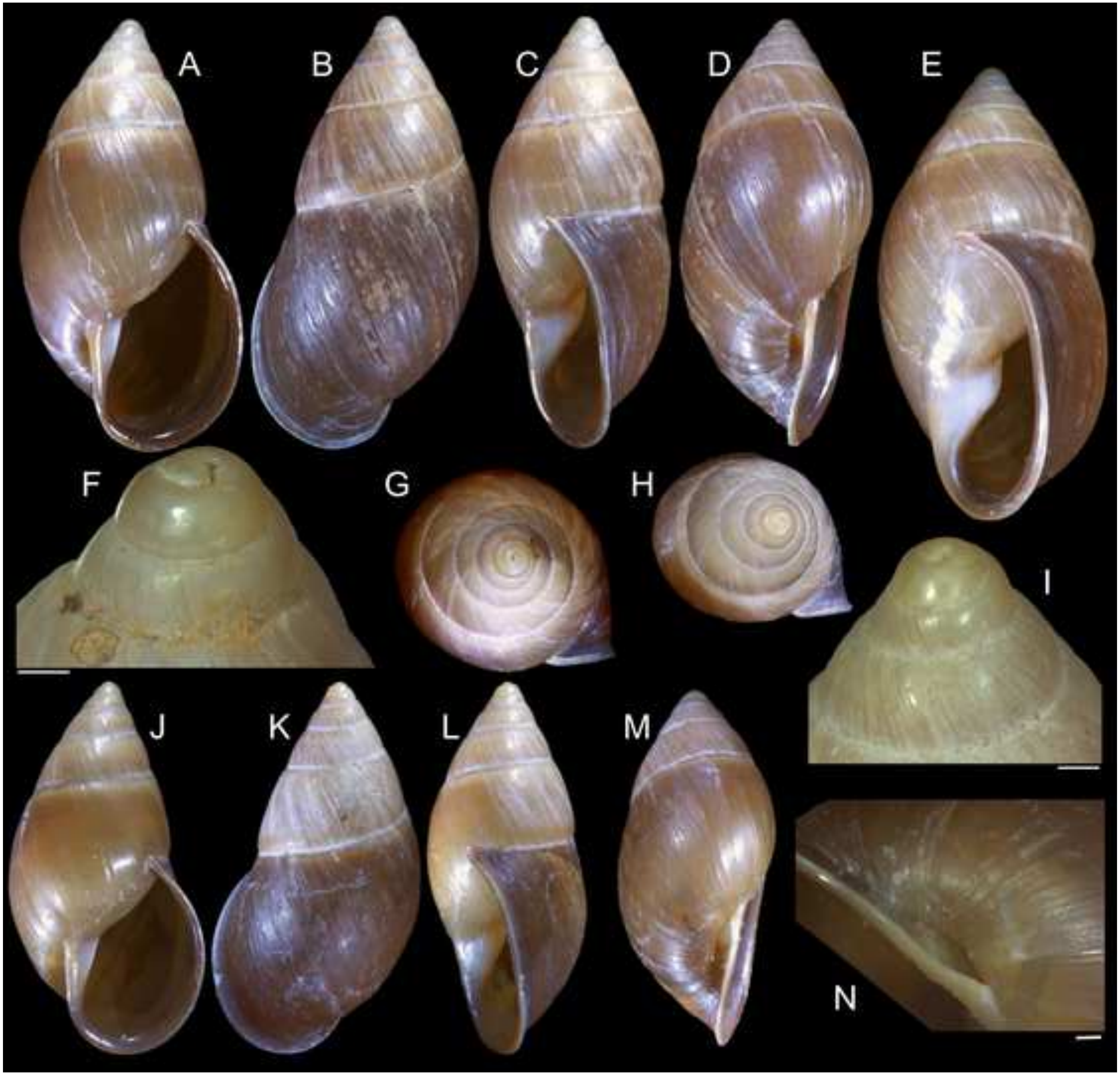

Figure 35

Click here to access/download;Figure;Kora 35b uhlei.jpg

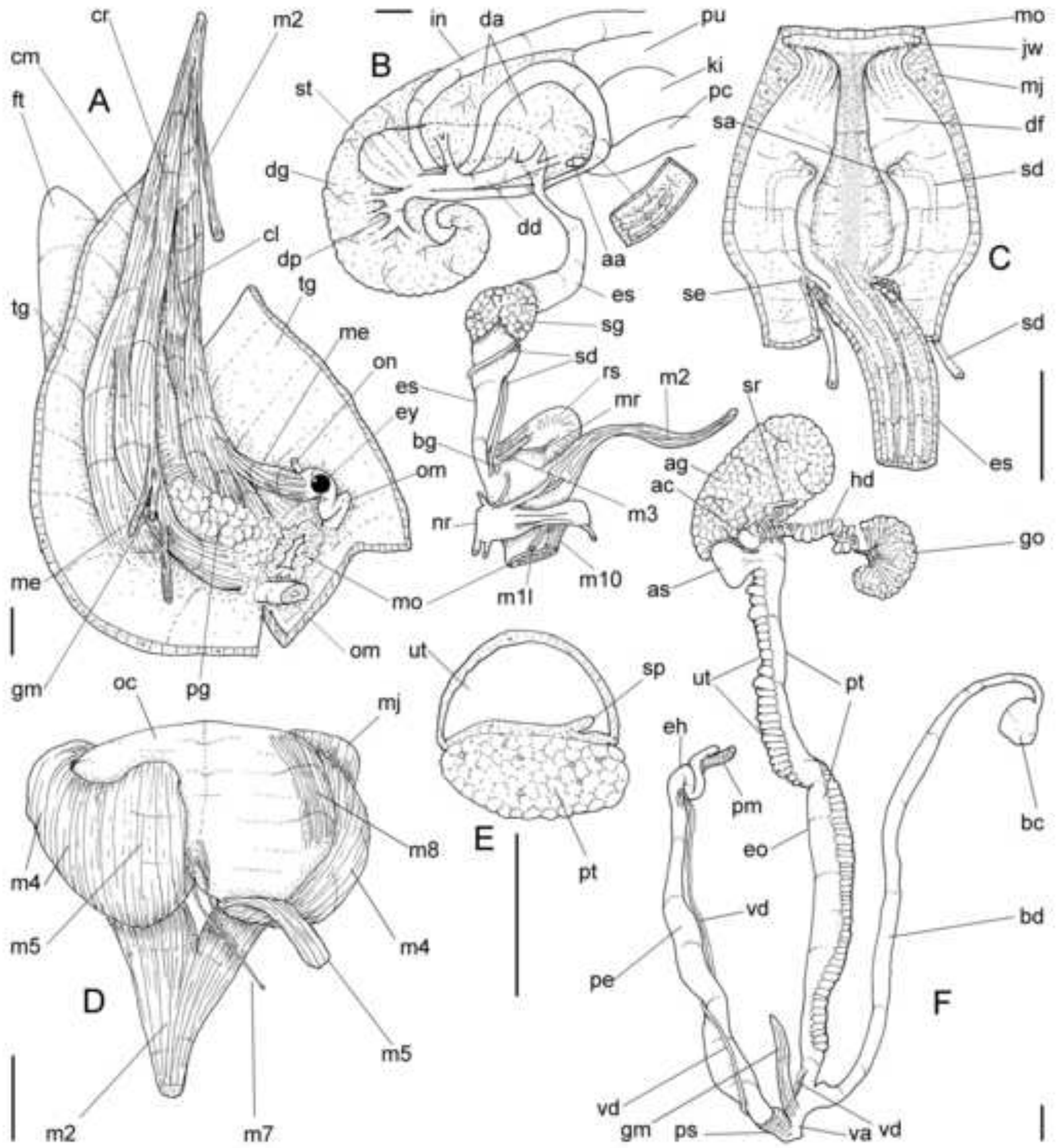

Figure 36

Click here to access/download;Figure;Kora 36b uhlei.jpg

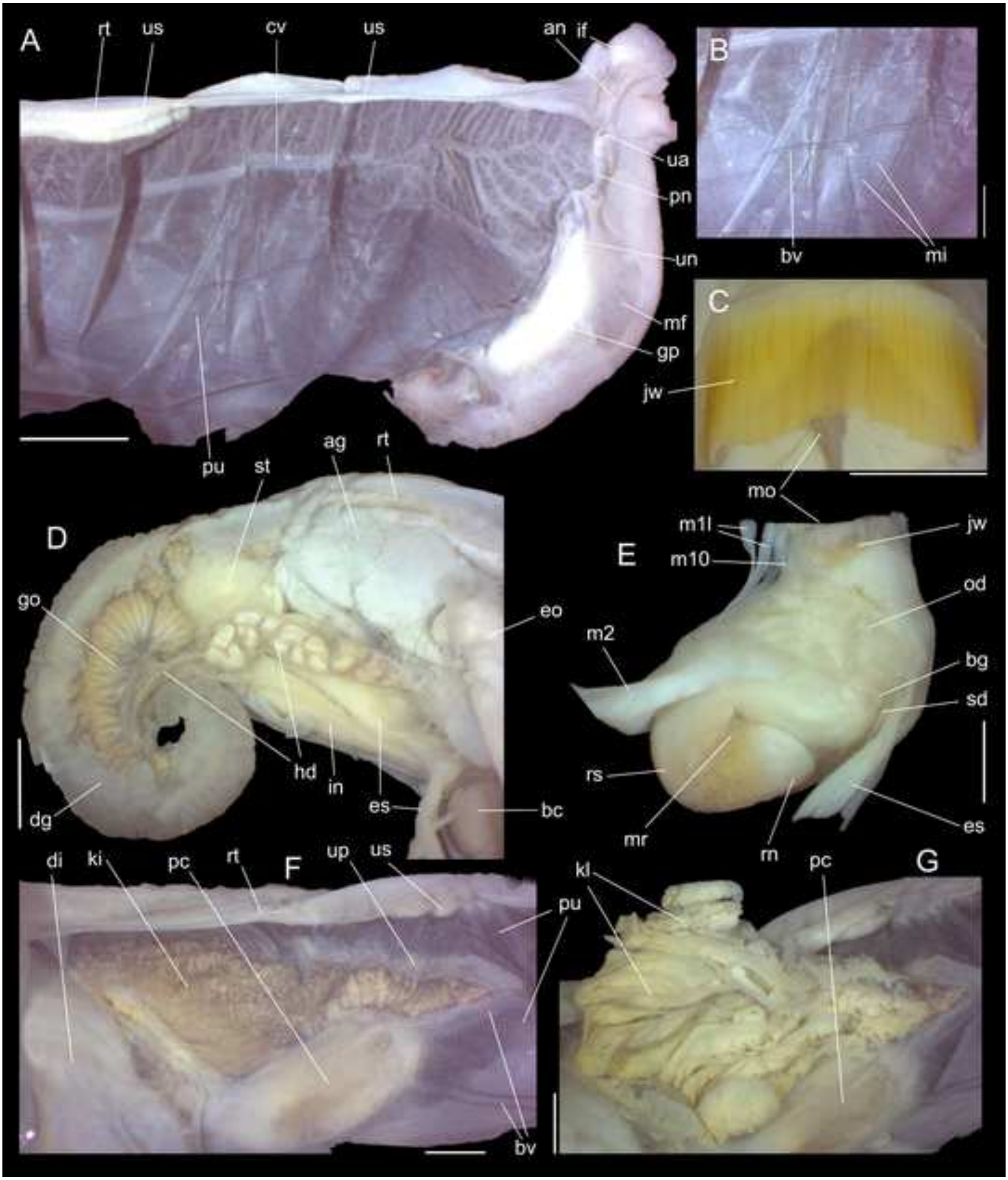

Figure 37

Click here to access/download;Figure;Kora 37b uhlei.jpg

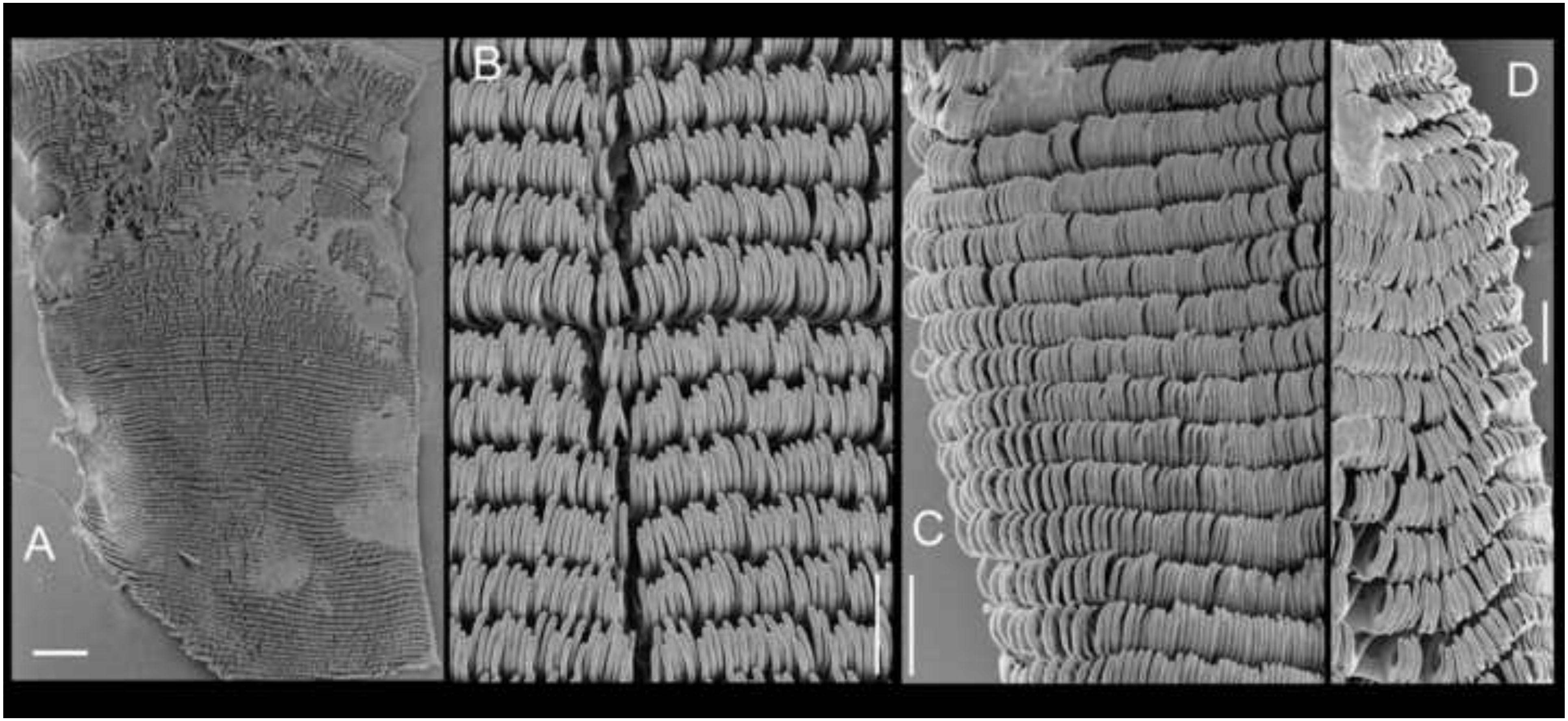

Figure 38

Click here to access/download;Figure;Kora 38b uhlei.jpg

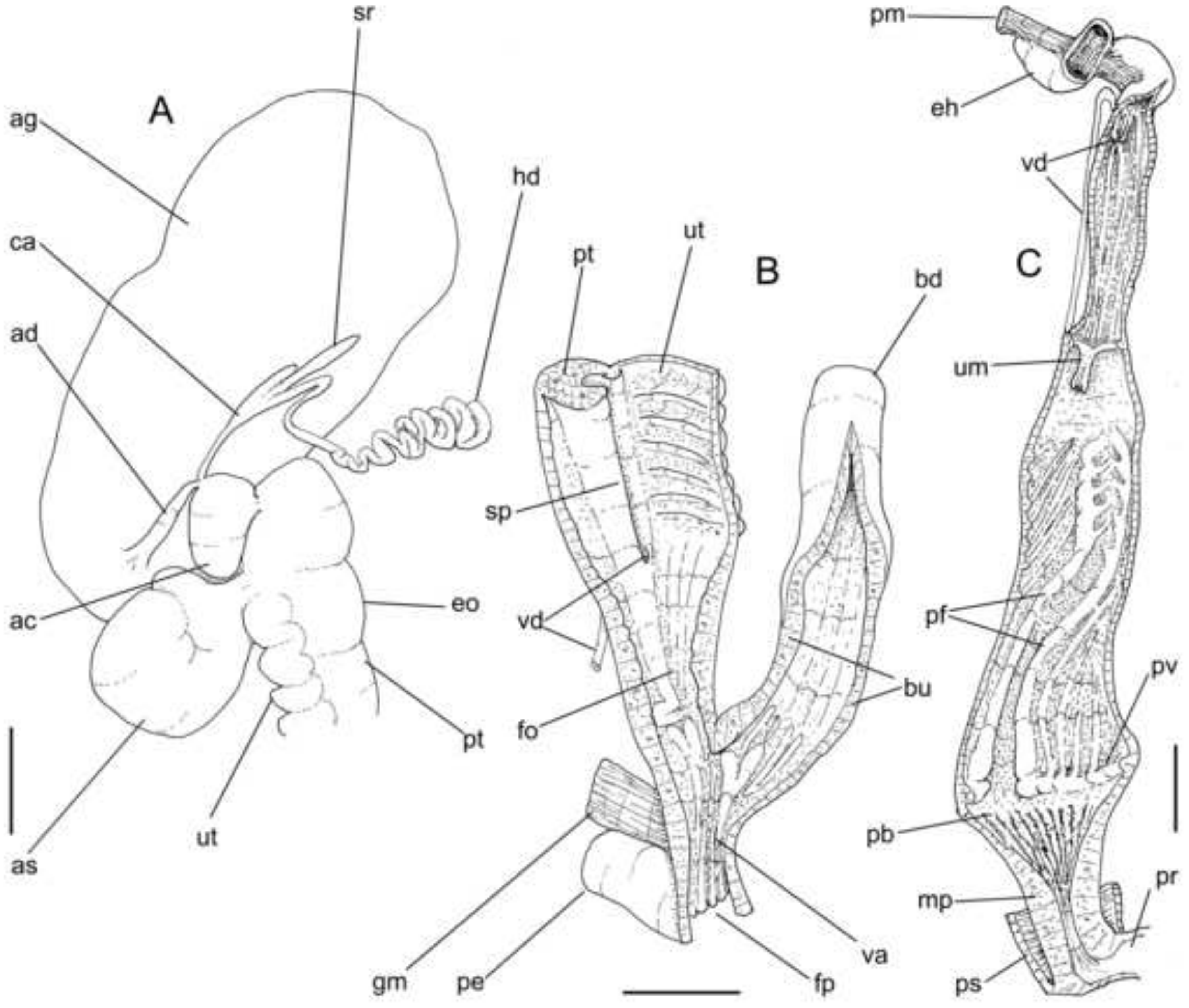

Figure 39

Click here to access/download;Figure;Kora 39b kremerorum.jpg

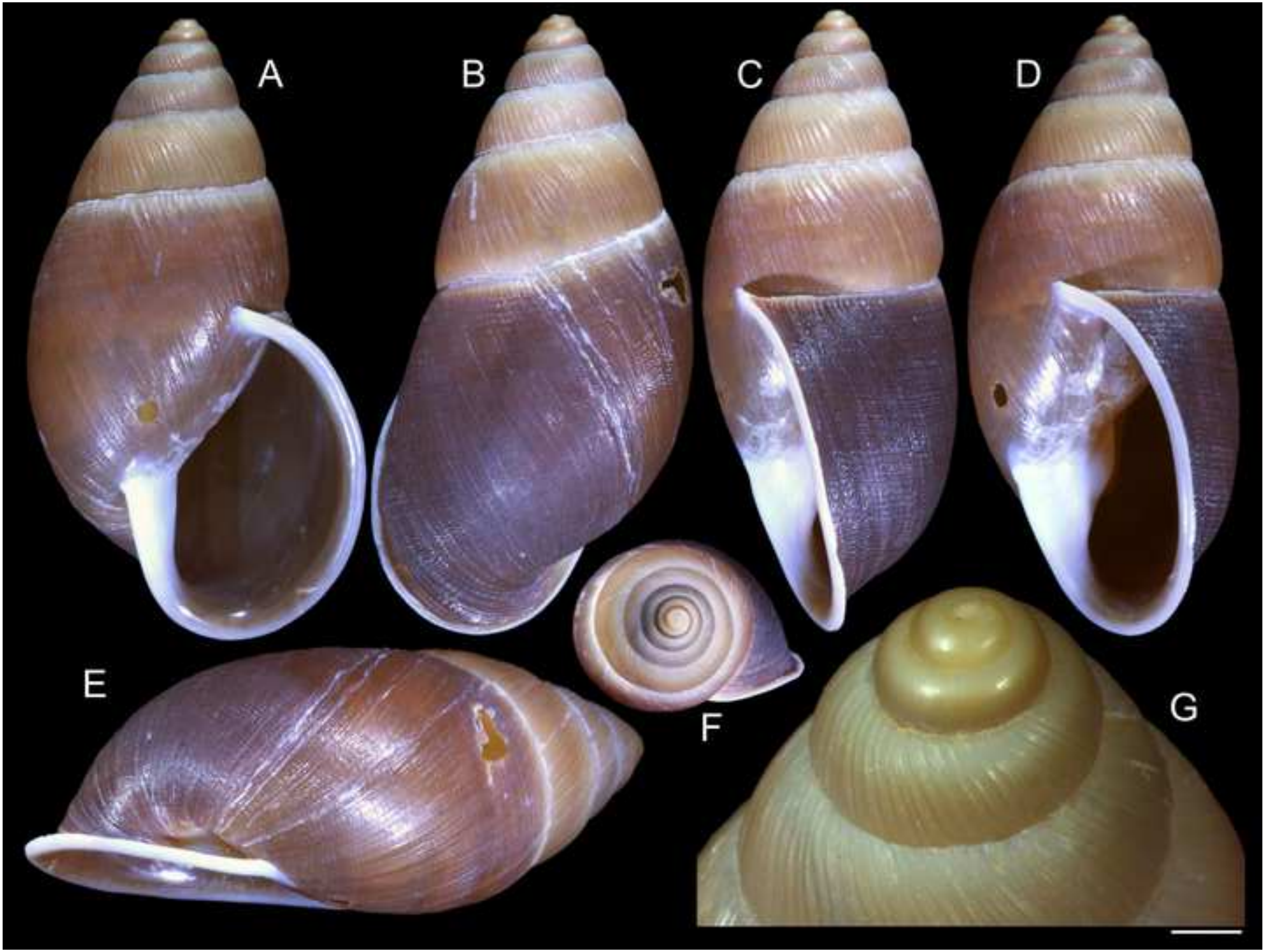

Click here to access/download;Figure;Kora 40b kremerorum.jpg

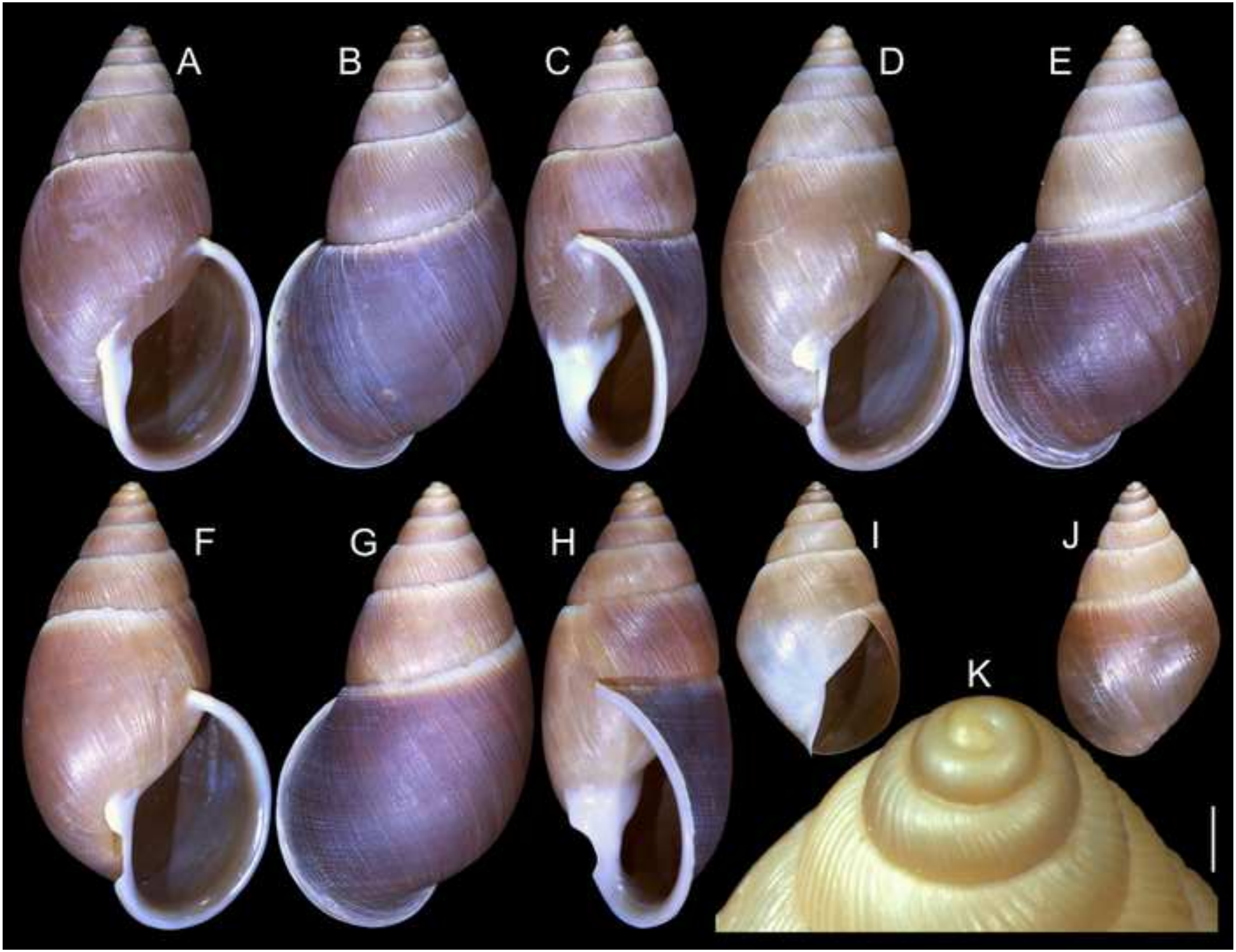

Click here to access/download;Figure;Kora 41b tocantis.jpg

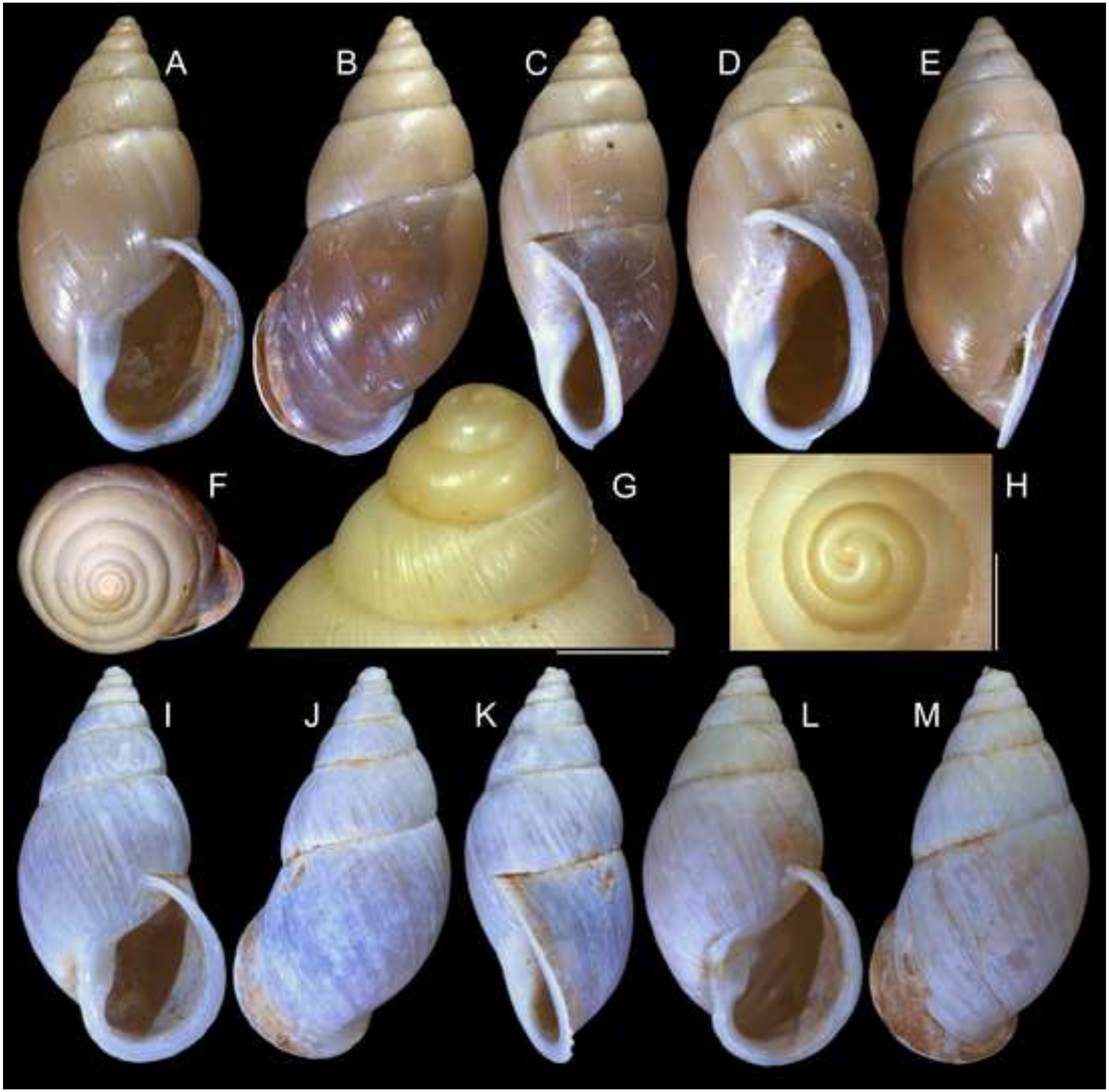

Figure 42

Click here to access/download;Figure;Kora 42b curumim.jpg

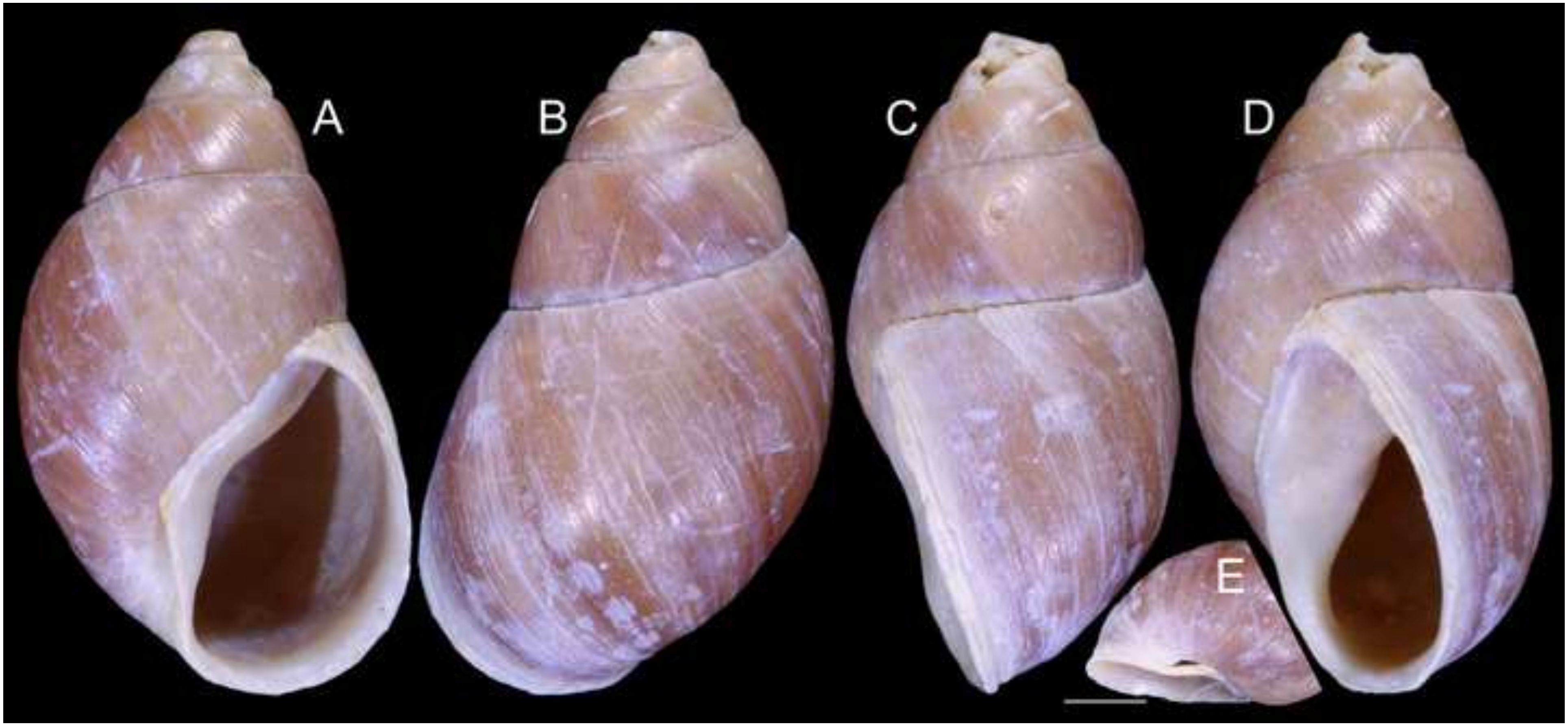

Figure 43

Click here to access/download;Figure;Kora 43b Koltrora.jpg

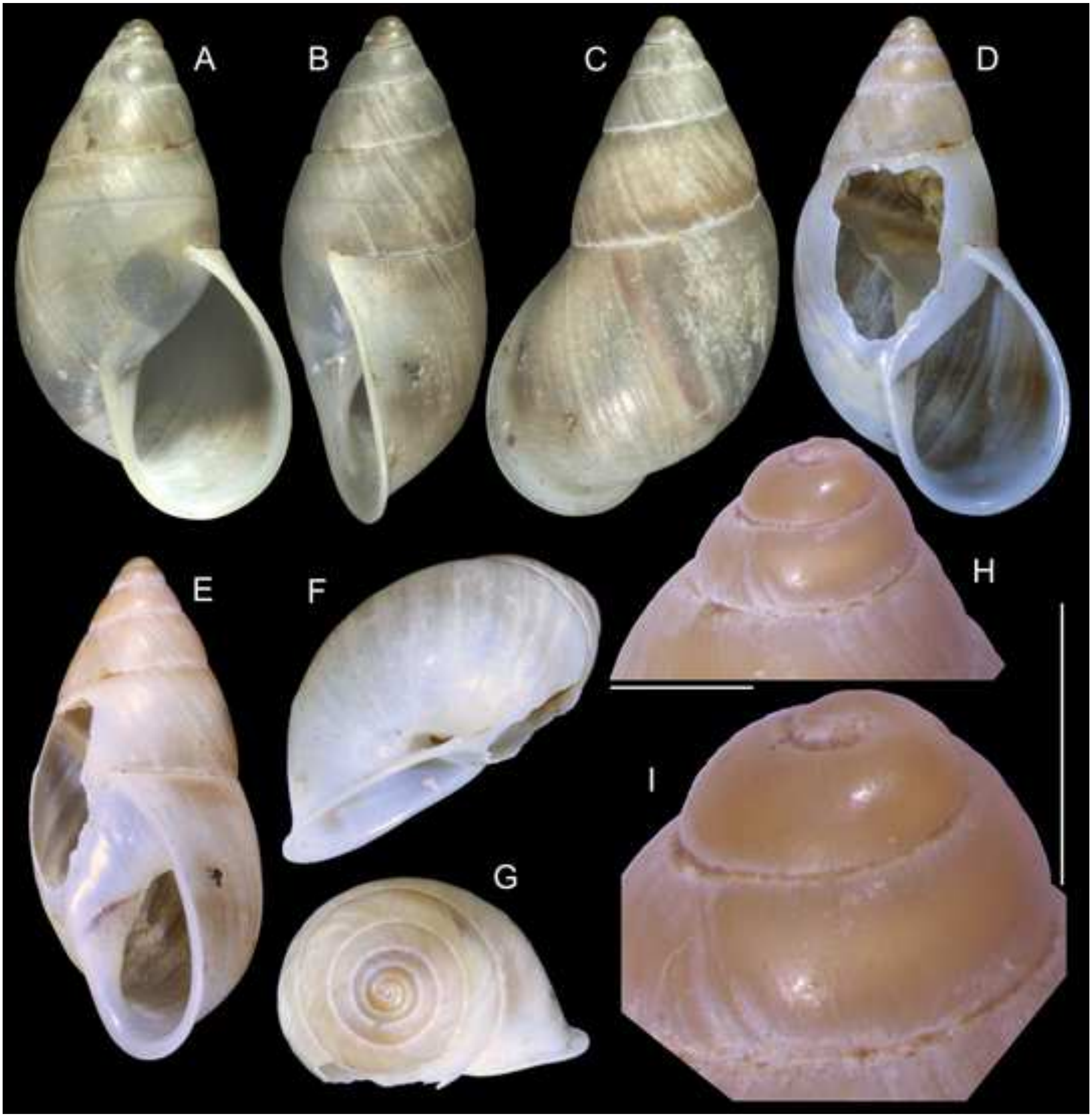

Figure 44 Click here to access/download;Figure;Kora 44b Koltrora.jpg

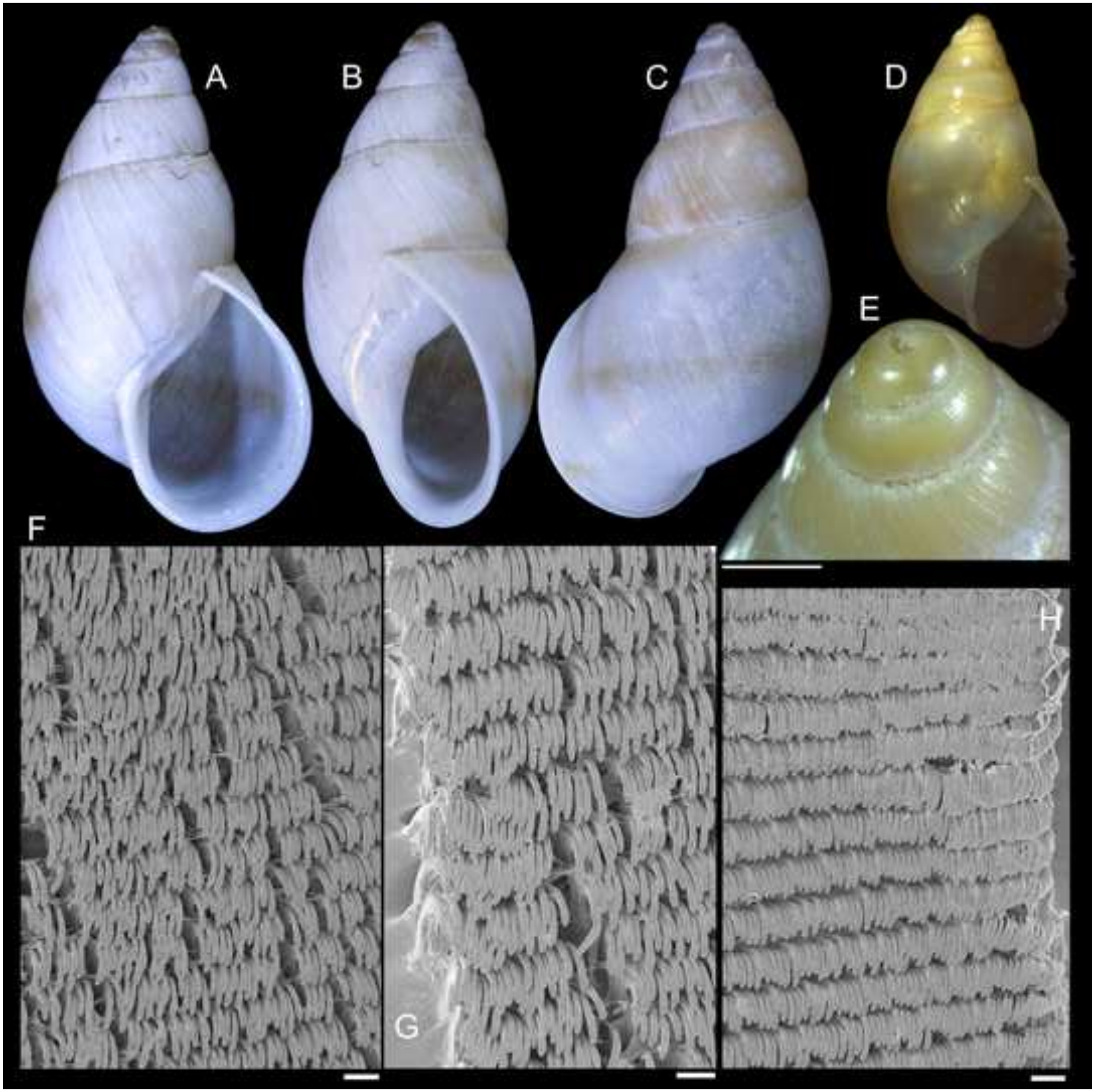

Figure 45

Click here to access/download;Figure;Kora 45b Koltrora.jpg

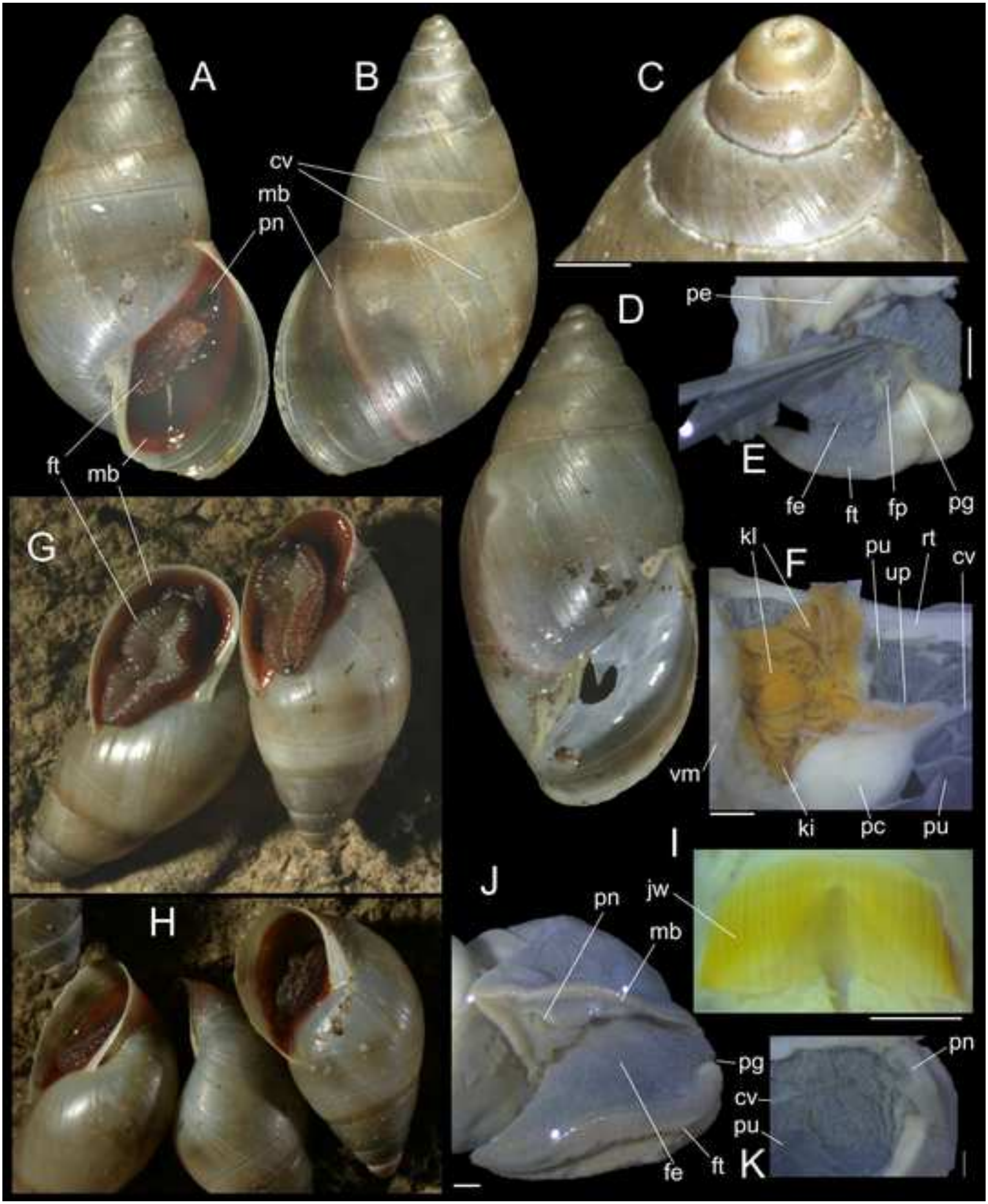

Figure 46

Click here to access/download;Figure;Kora 46b Koltrora.jpg

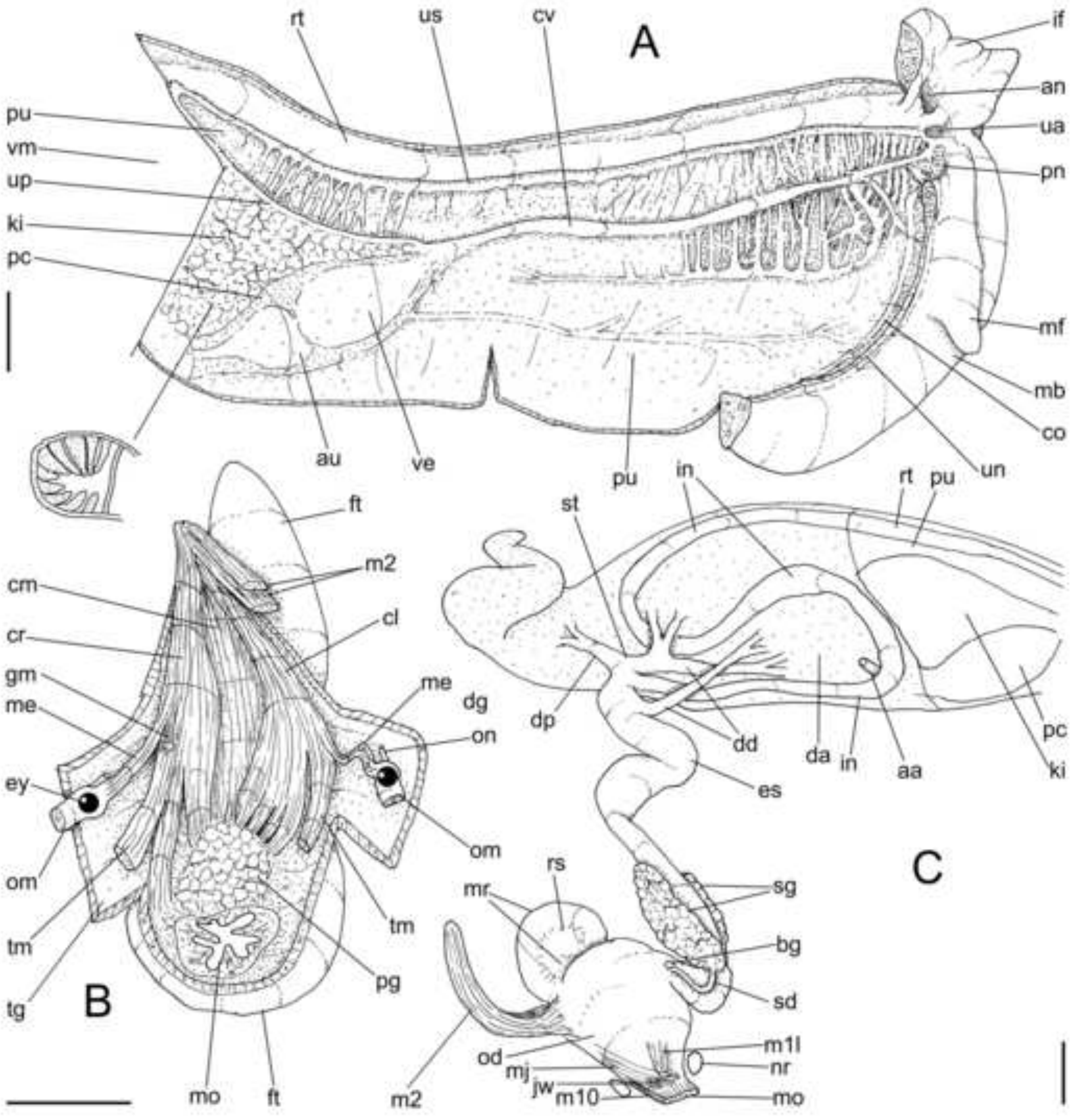

Figure 47

Click here to access/download;Figure;Kora 47b Koltrora.jpg

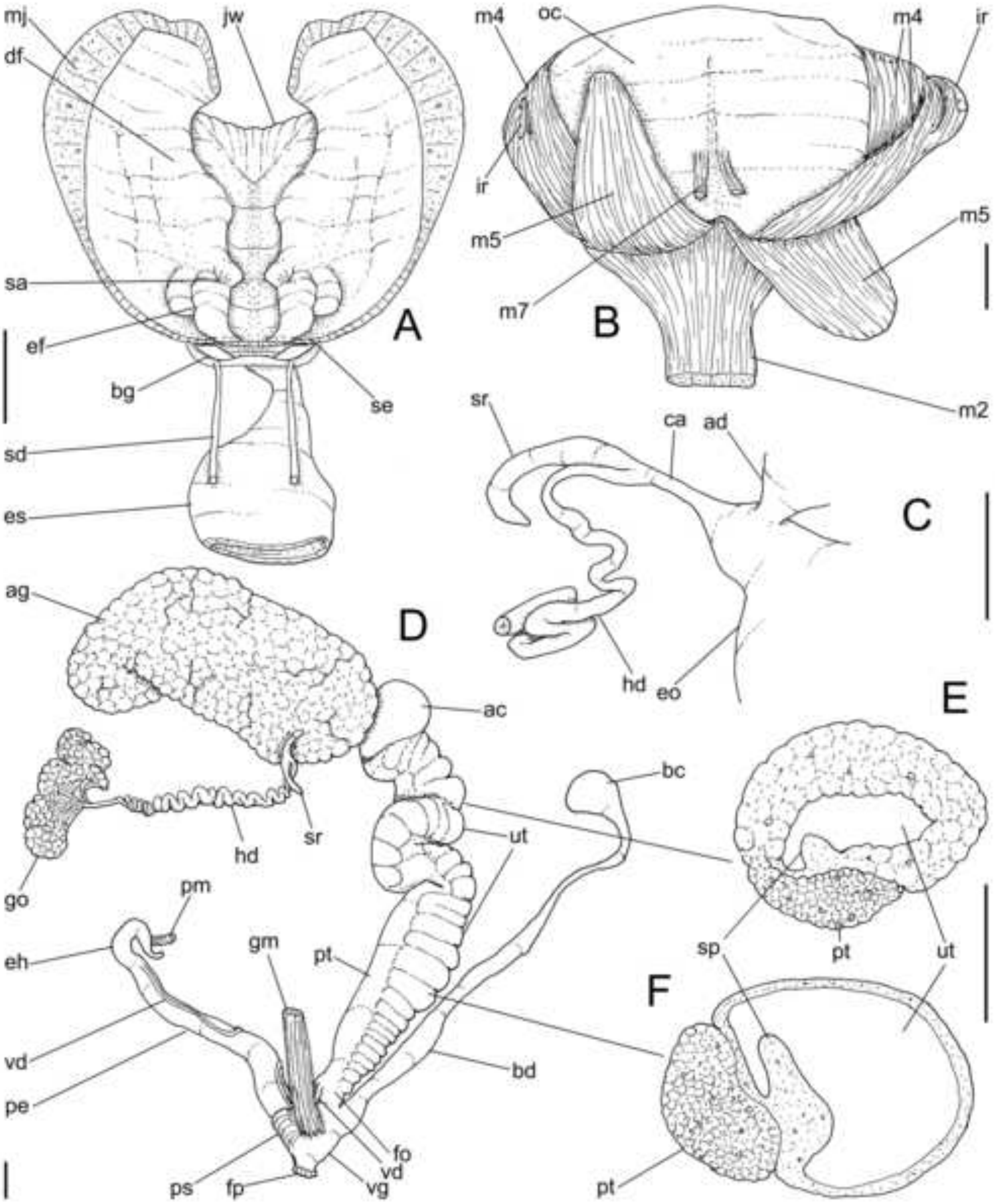

Figure 48

Click here to access/download;Figure;Kora 48b Koltrora.jpg

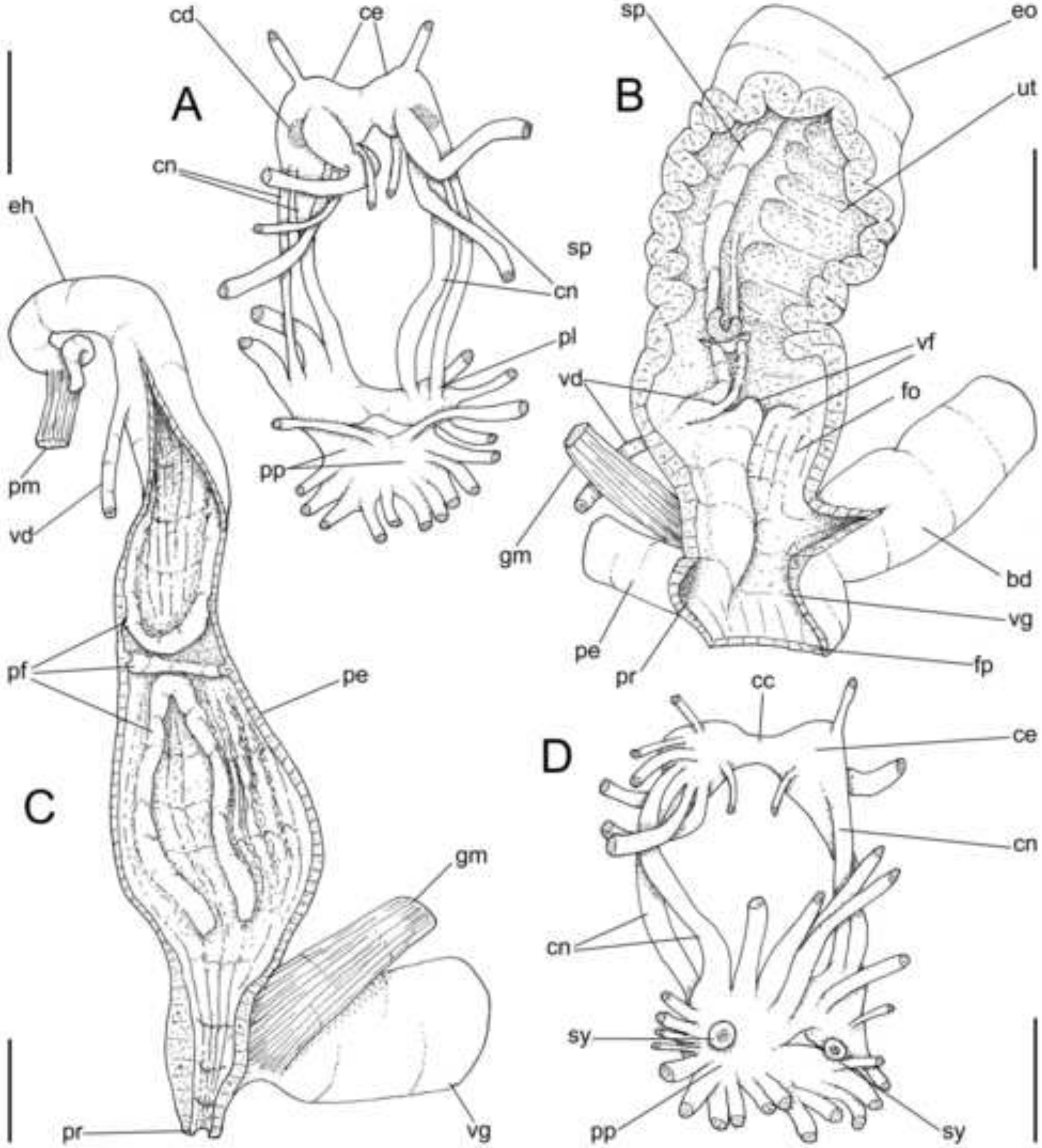

Figure 49

Click here to access/download;Figure;Kora 49b Neolobbi.jpg

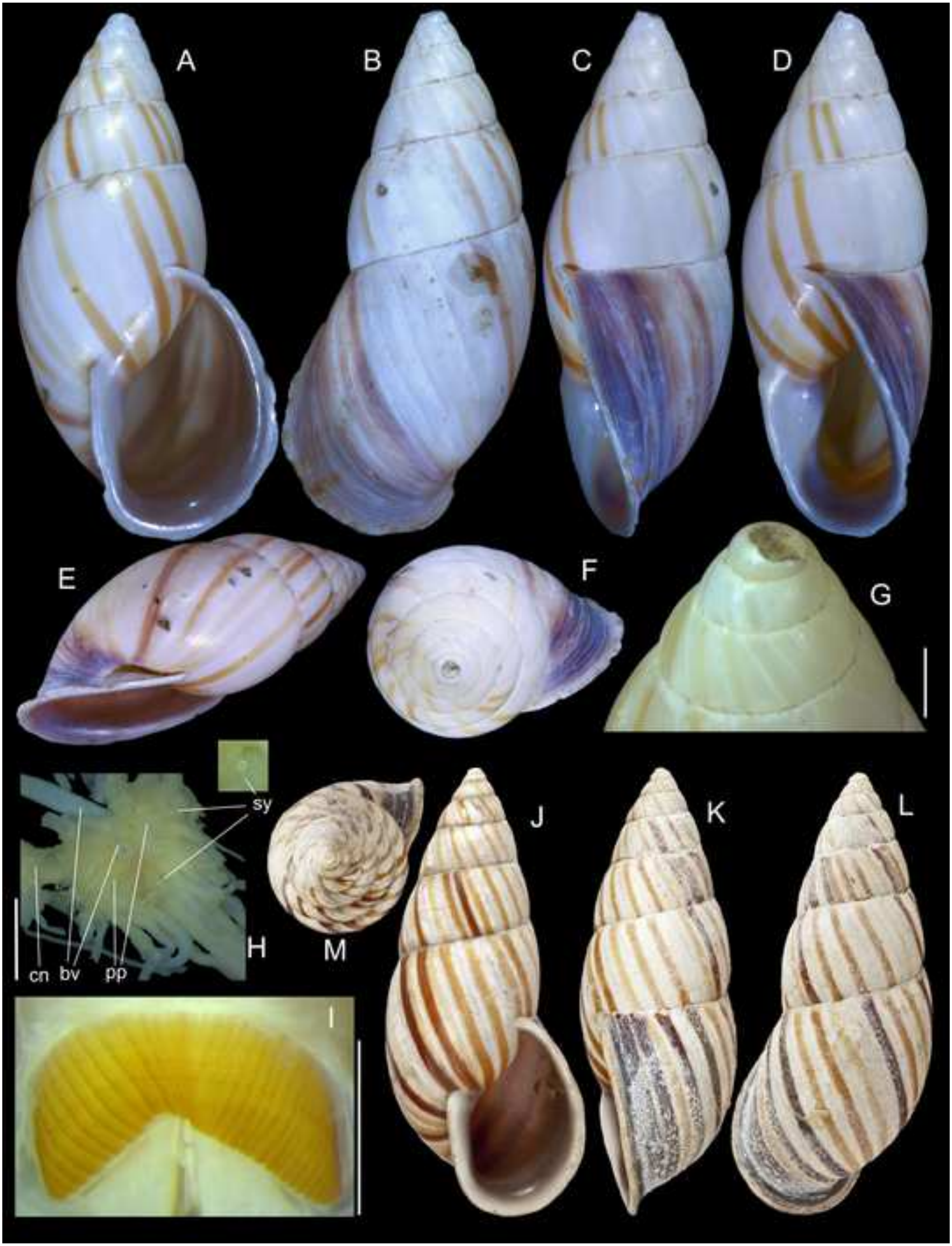

Figure 50

Click here to access/download;Figure;Kora 50b Neolobbi.jpg

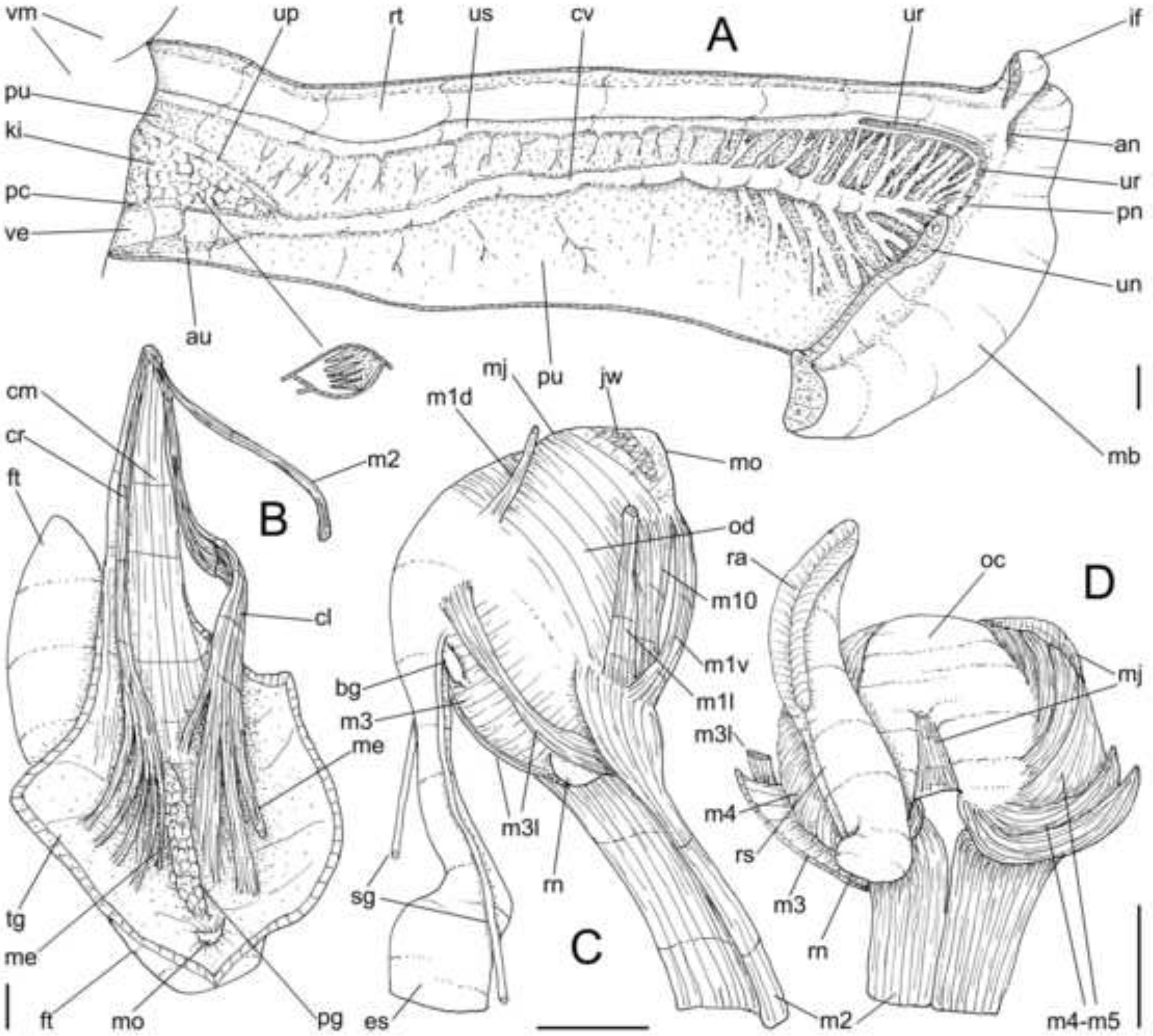

Click here to access/download;Figure;Kora 51b Neolobbi.jpg

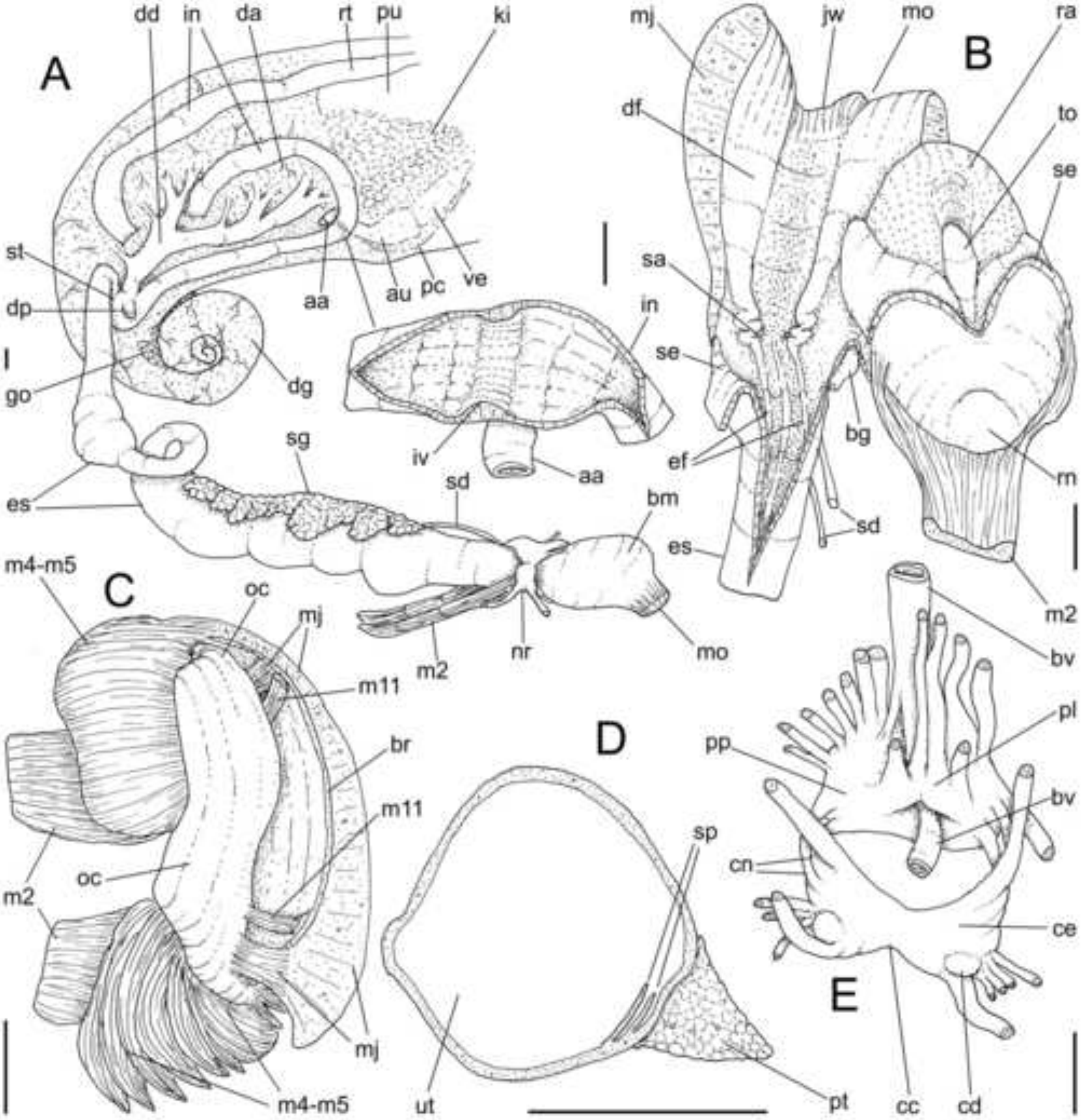

Figure 52

Click here to access/download;Figure;Kora 52b Neolo.jpg

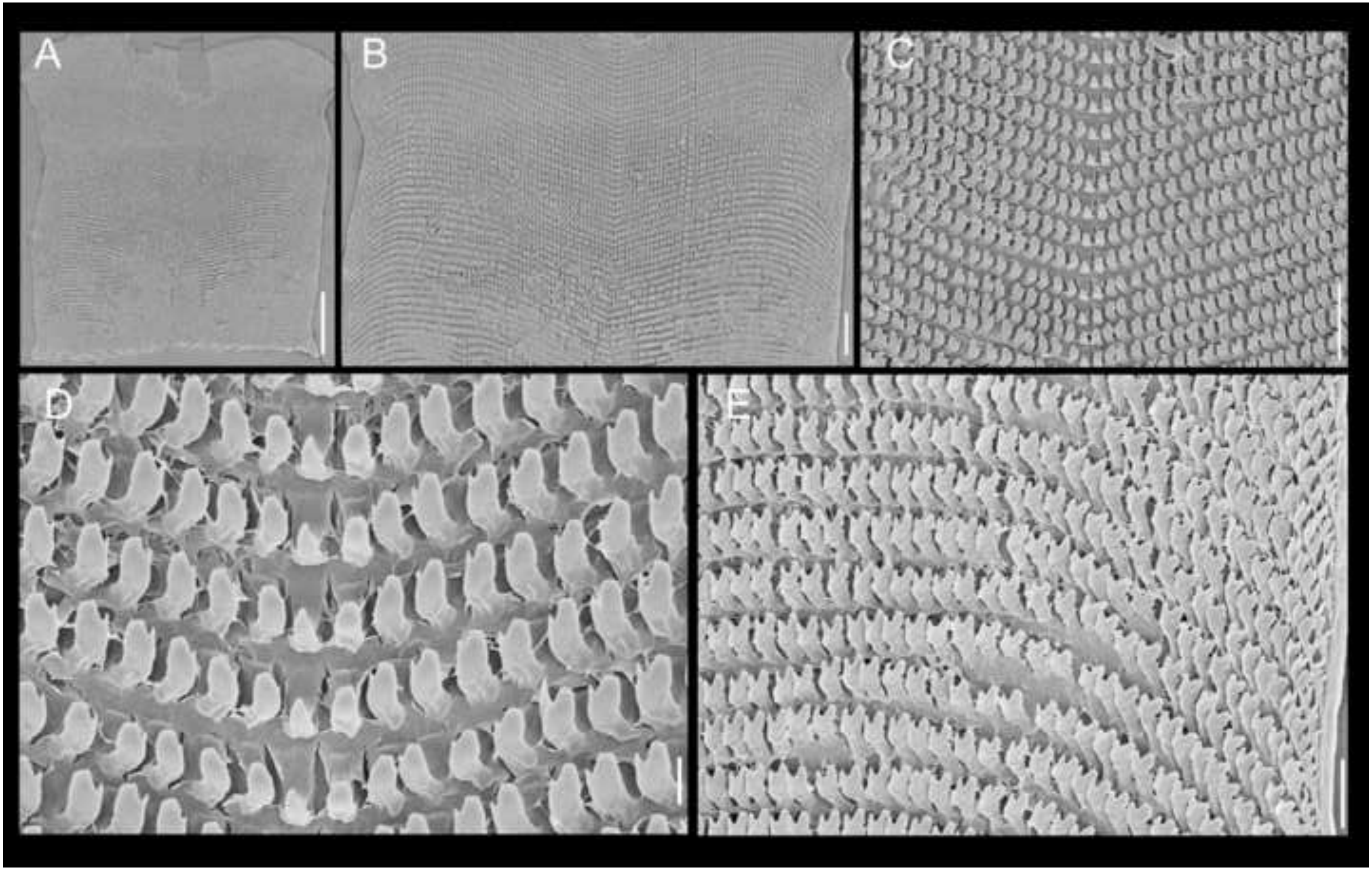

Figure 53 Click here to access/download;Figure;Kora 53b Neolobbi.jpg

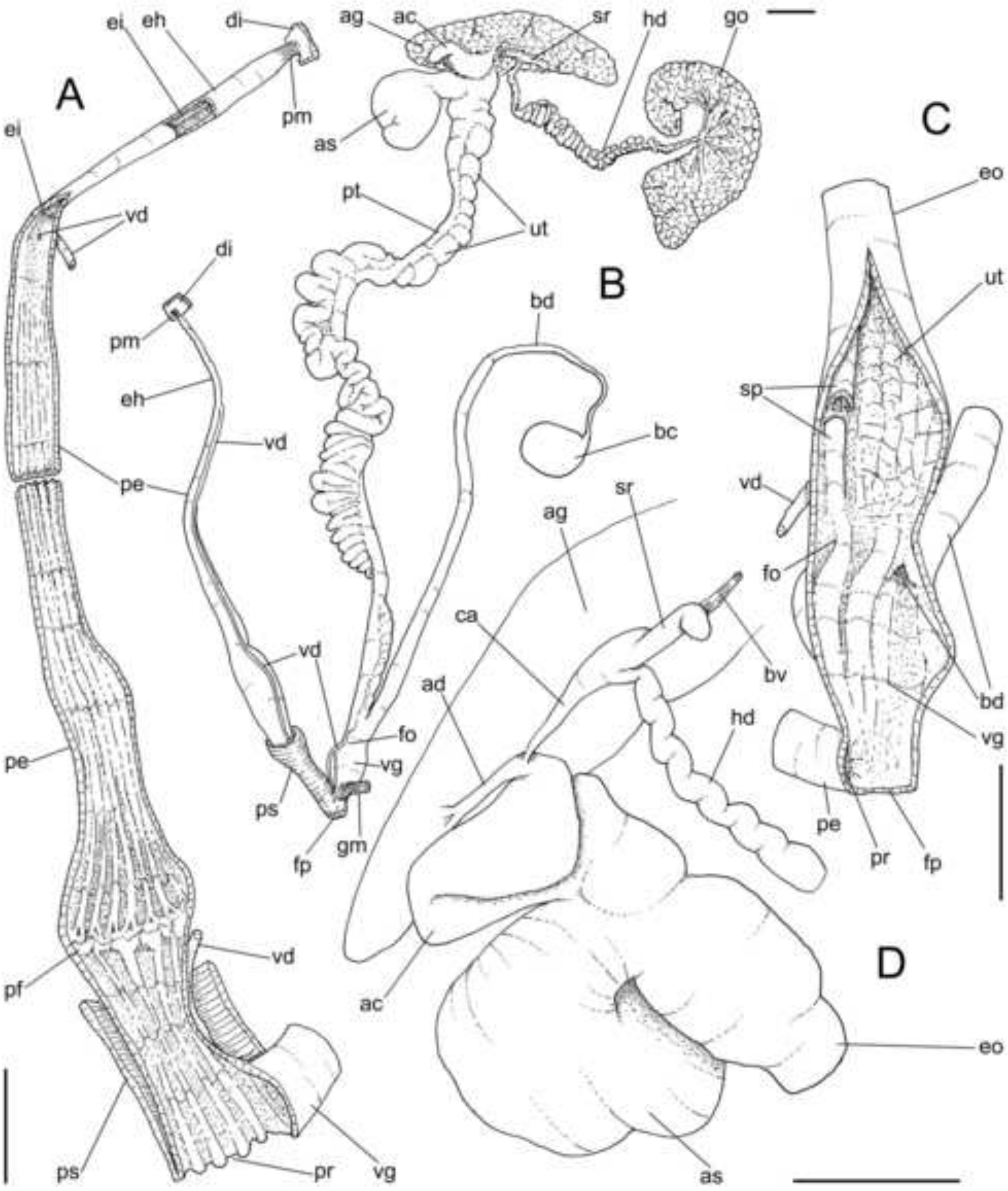

Figure 54

Click here to access/download;Figure;Kora 54b Neocremno.jpg

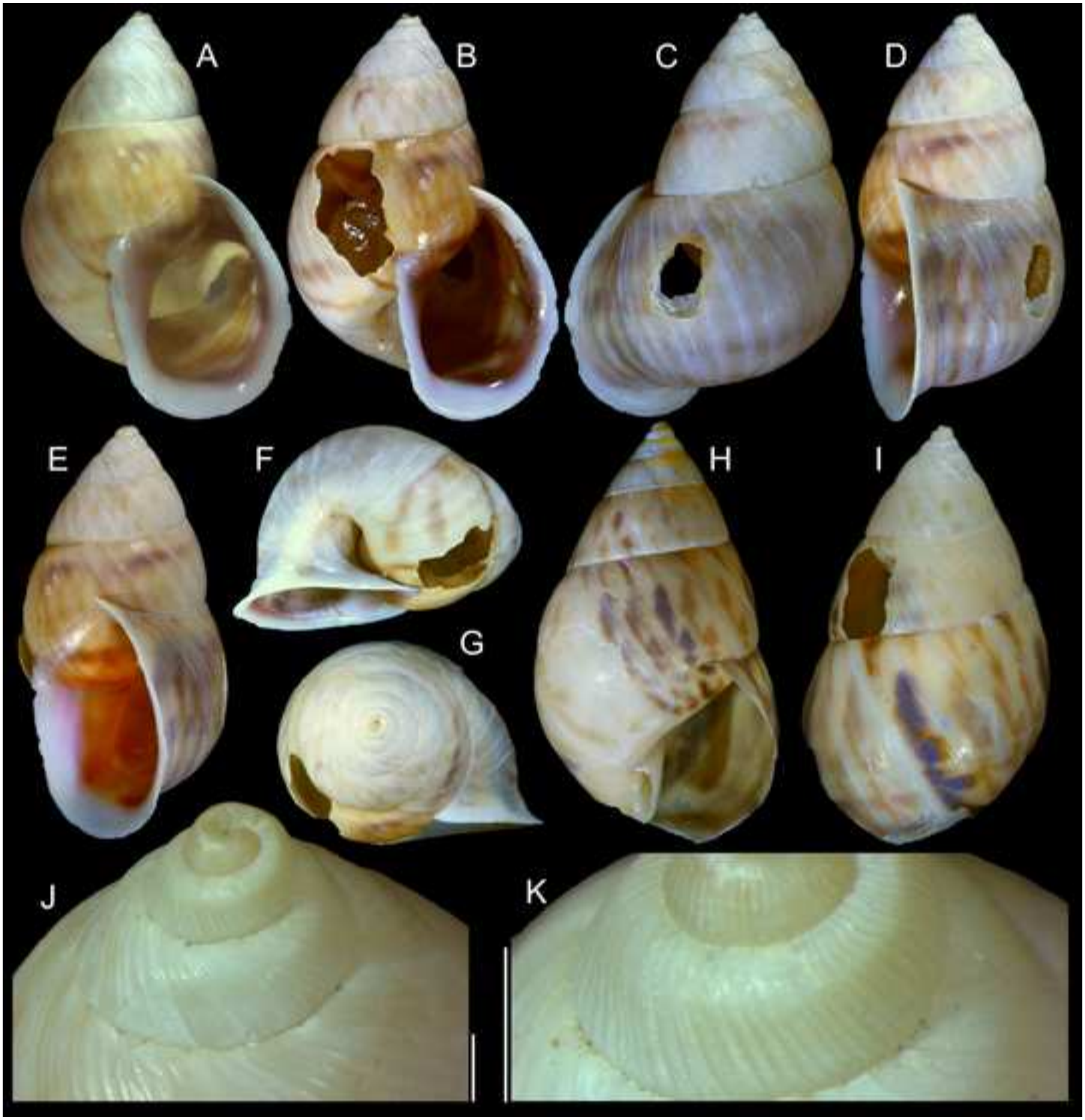

Figure 55

Click here to access/download;Figure;Kora 55b Neocremno.jpg

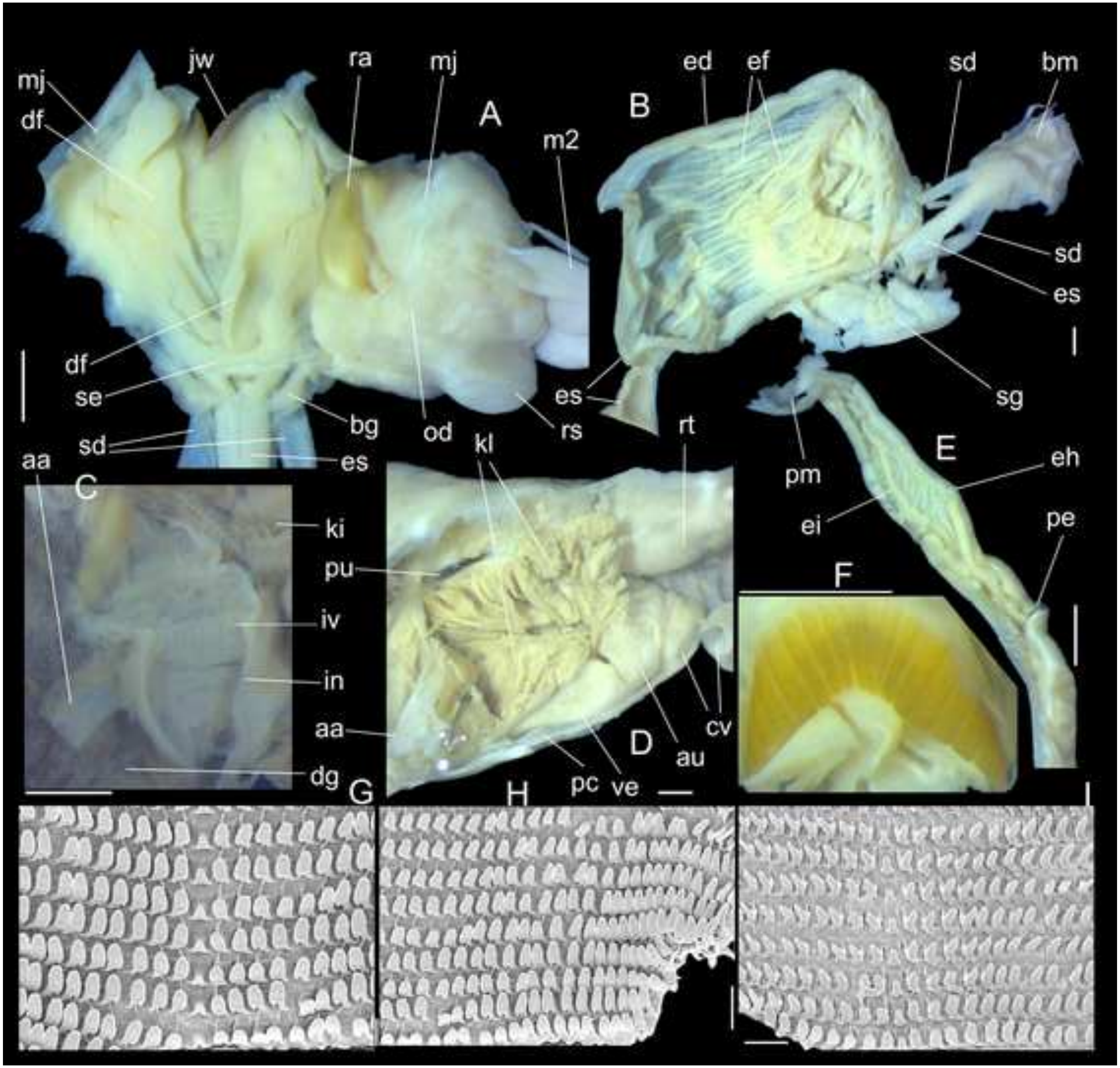

Figure 56 Click here to access/download;Figure;Kora 56b Neocremno.jpg

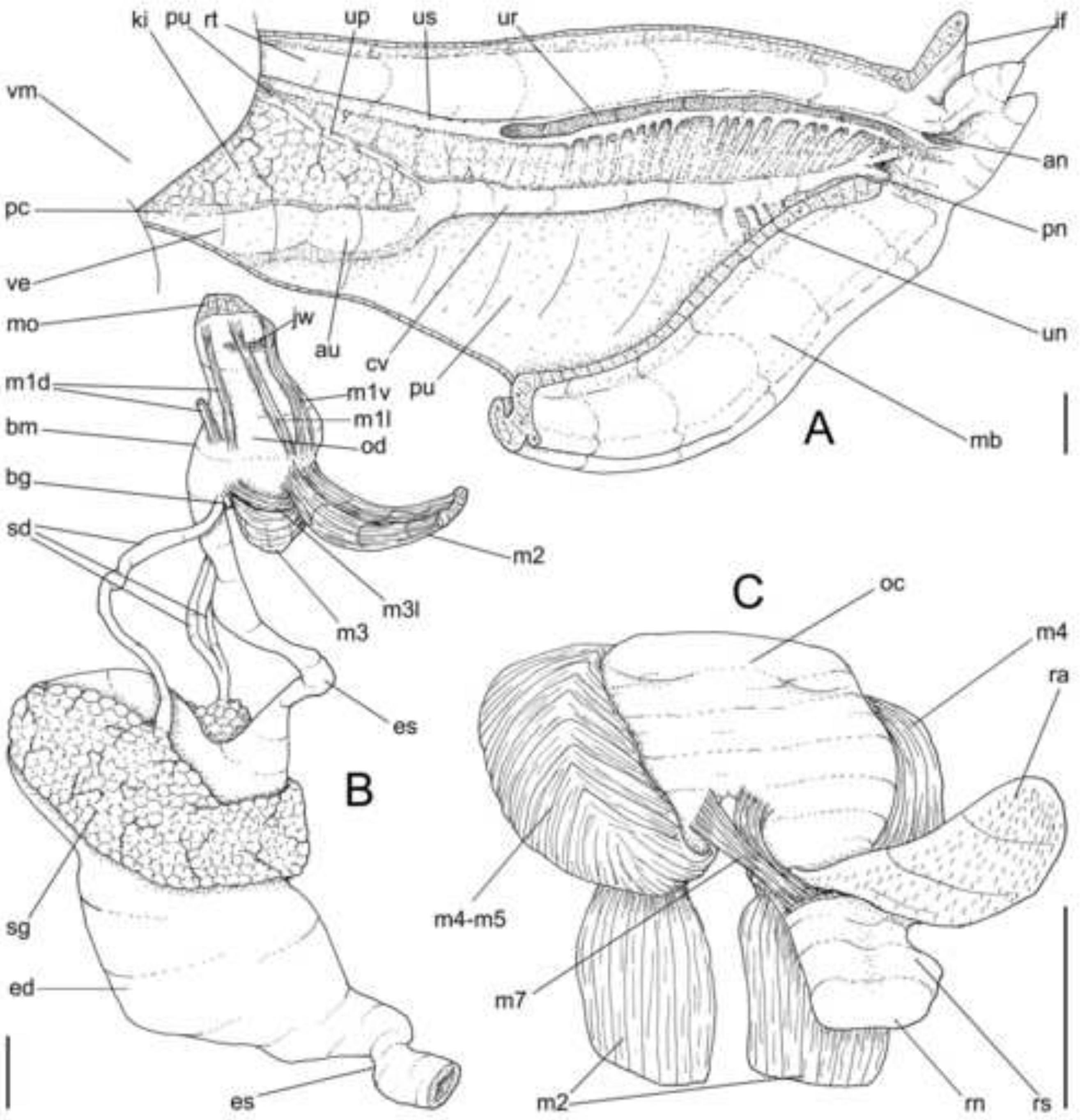

Figure 57

Click here to access/download;Figure;Kora 57b Neocremno.jpg

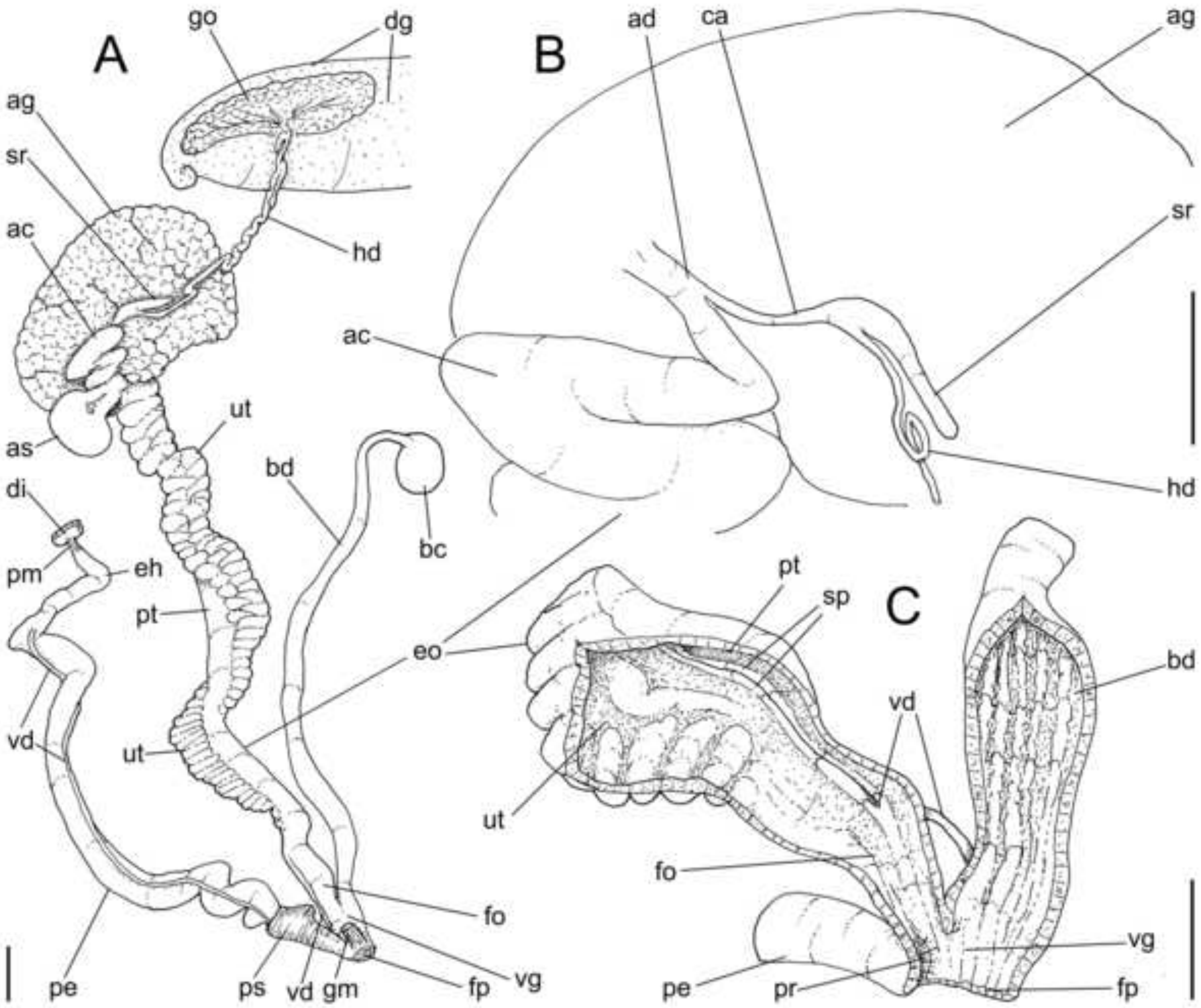

Figure 58

Click here to access/download;Figure;Kora 58b confront.jpg

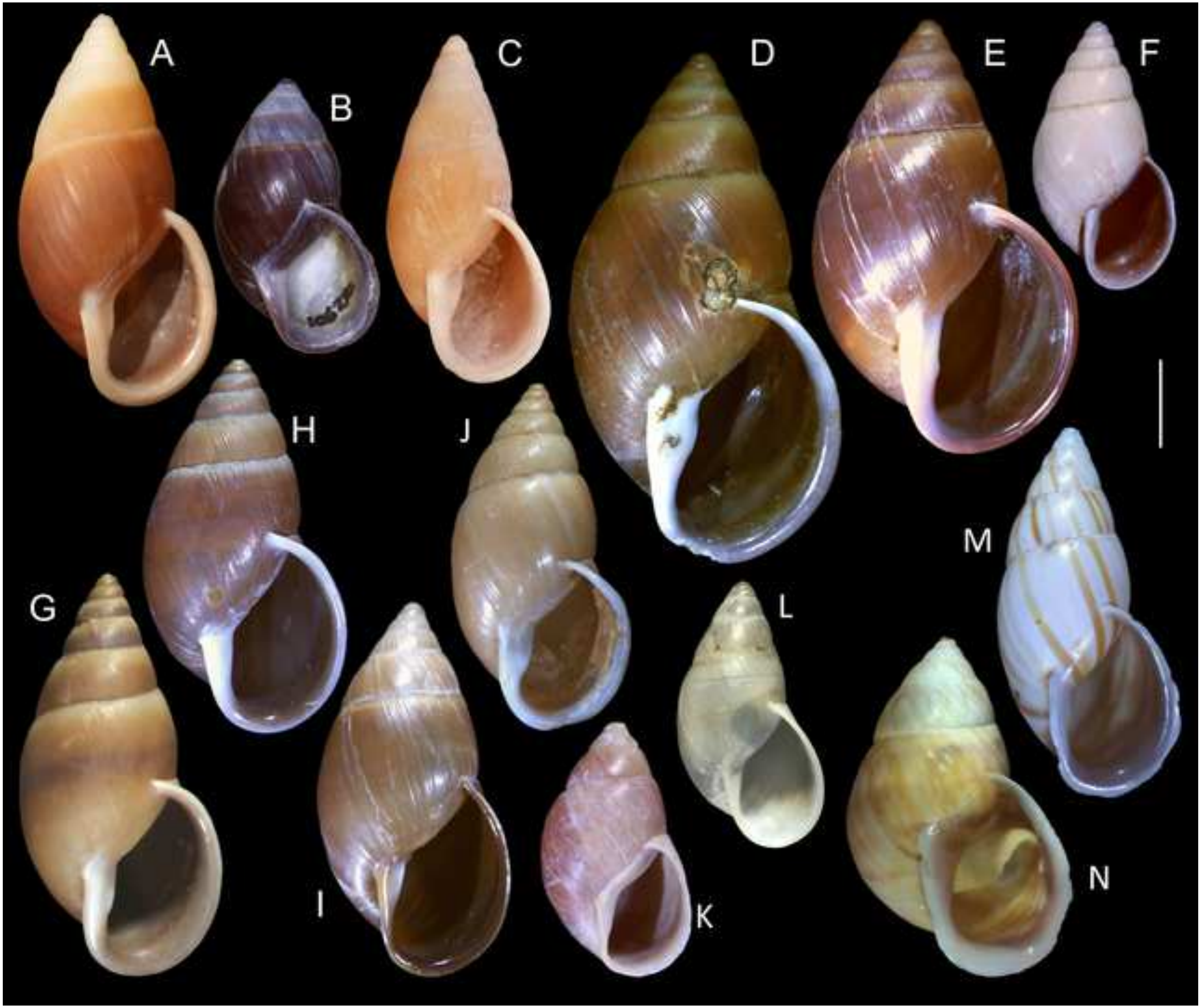

Figure 59

Click here to access/download;Figure;Kora 59b Anctus.jpg

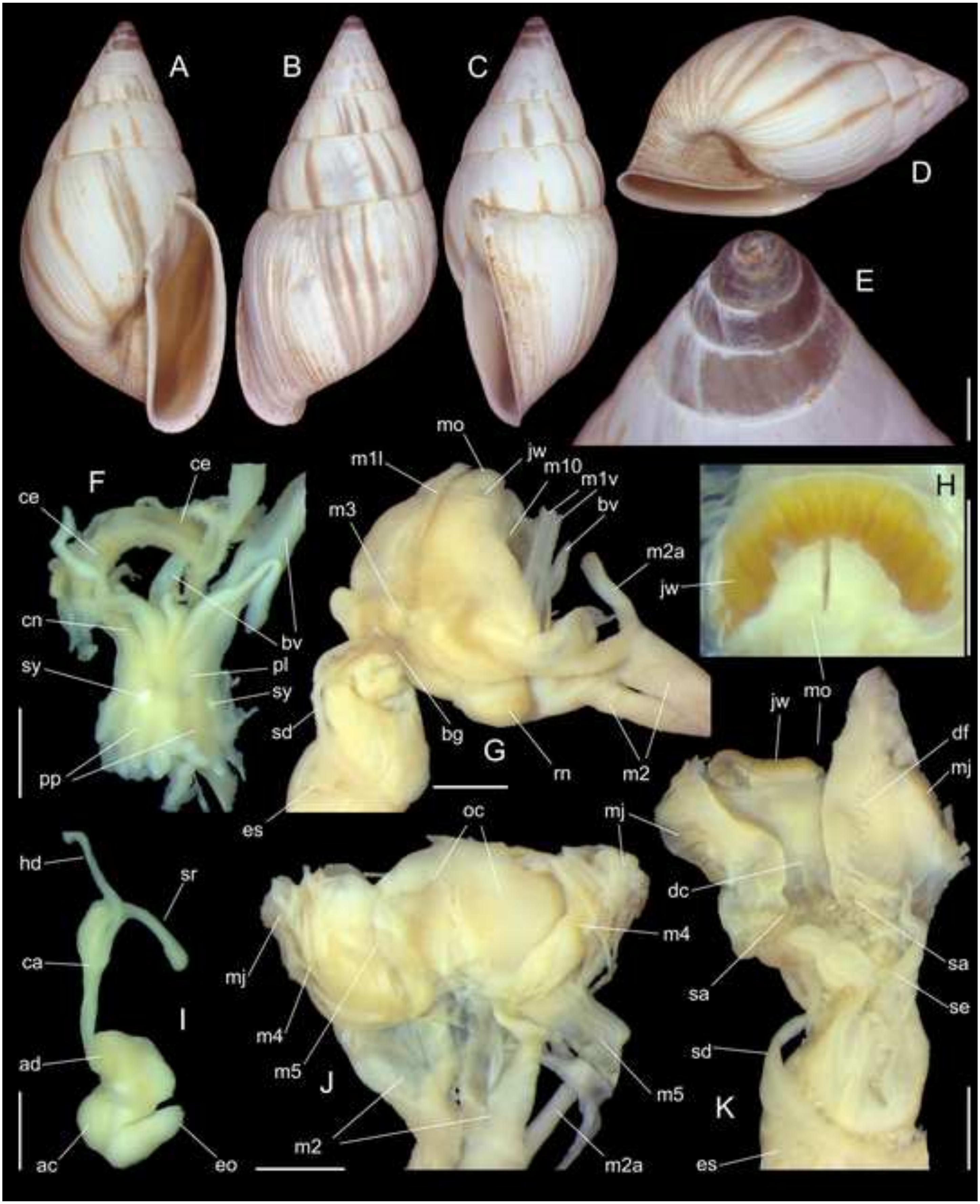

Click here to access/download;Figure;Kora 60b map.jpg

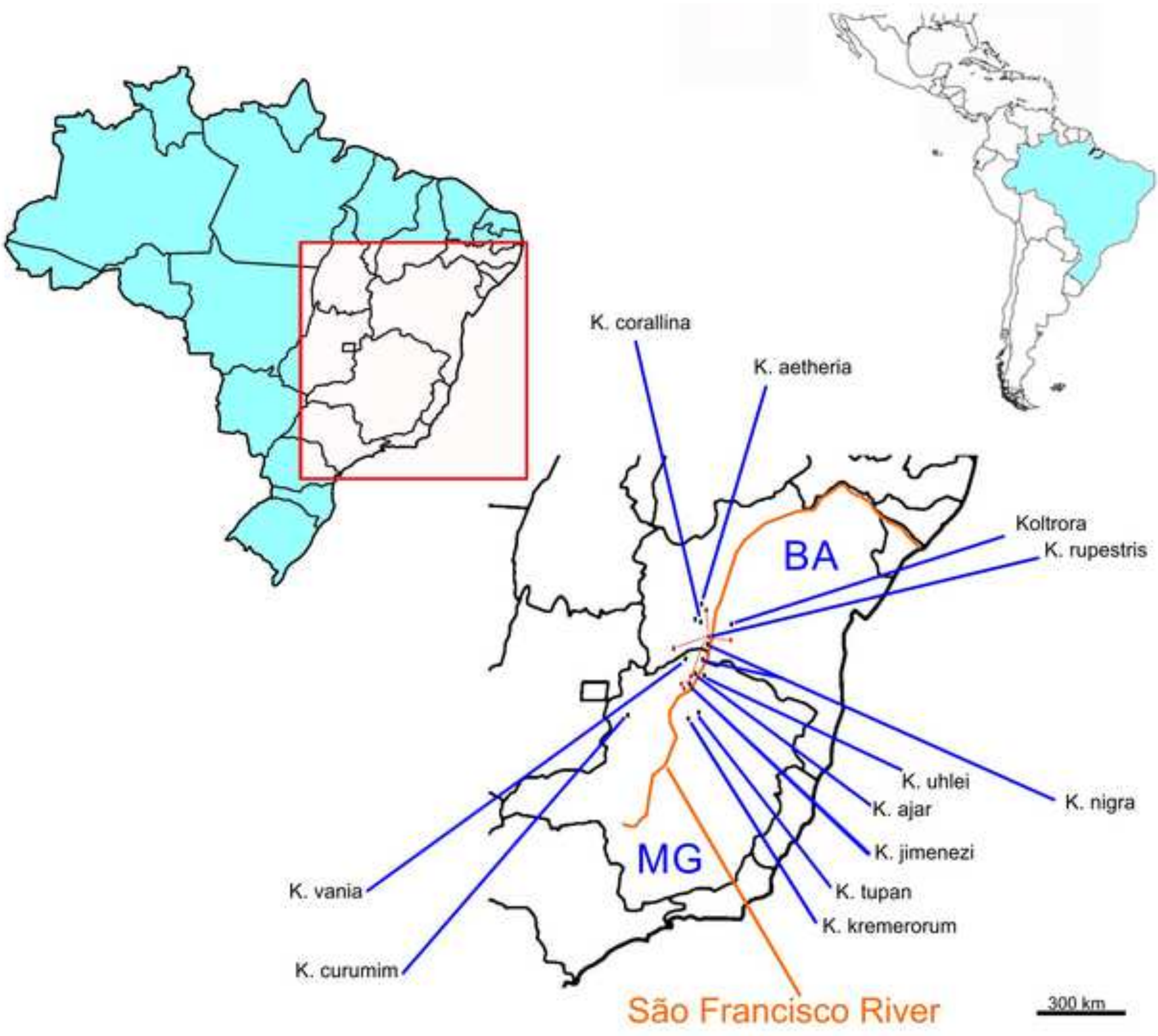

Figure 61

Click here to access/download;Figure;Kora 61b cladogram.jpg

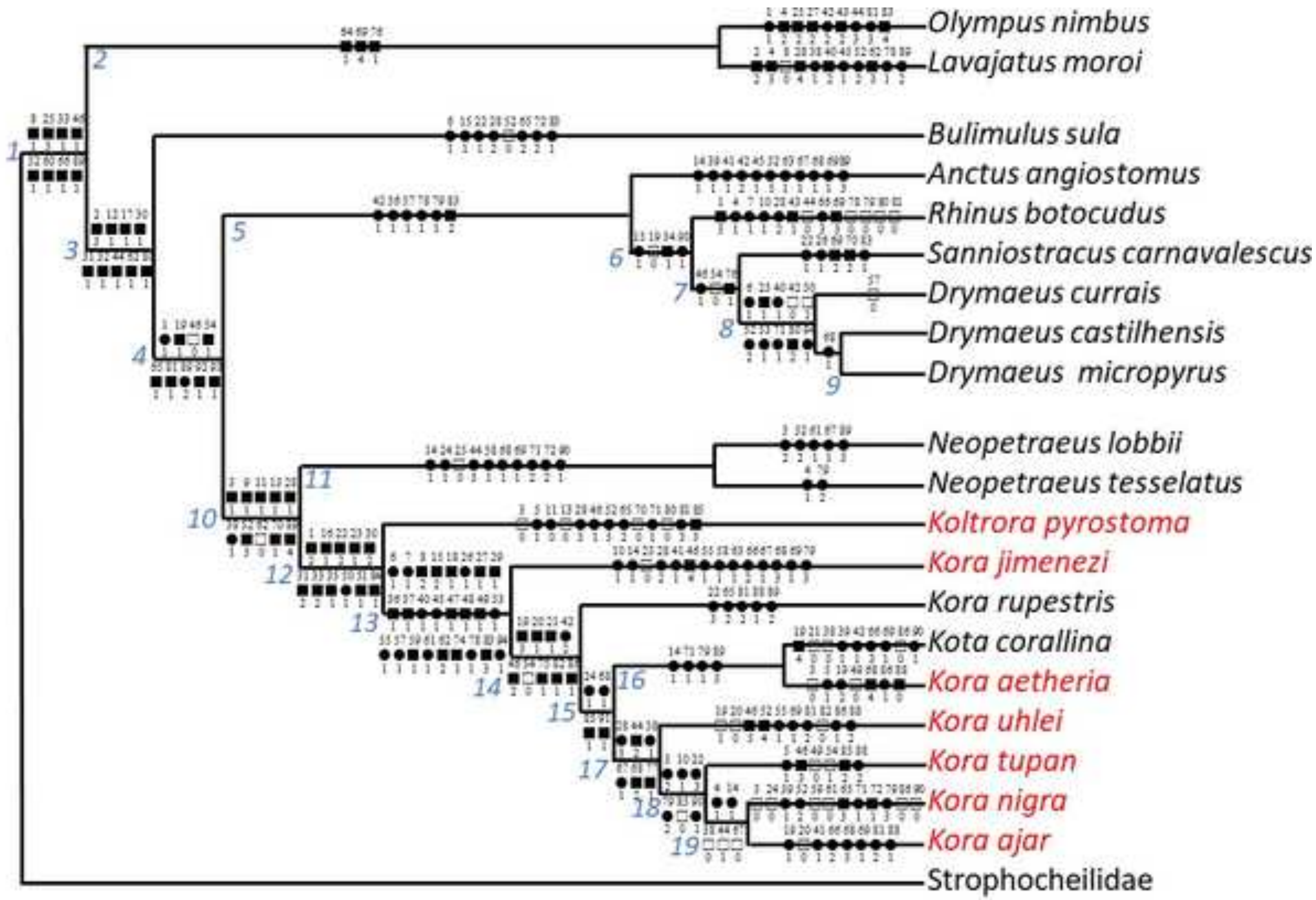

Supplement: Attachment — Submitted filename: PONE-D-23-41760_reviewer(1) Answered.pdf [file pone.0315272.s005.pdf]
