# Supplementary material for: Review of the land snails of the genus Kora from Brazil, with description of eight new species and a new related genus Koltrora, including comparison with two Andean Neopetraeus species (Gastropoda, Eupulmonata, Orthalicoidea)
Source: PLoS One. 2024 Dec 19;19(12):e0315272. doi: 10.1371/journal.pone.0315272 (PMC13052136; doi:10.1371/journal.pone.0315272)
Supplement: Attachment — Submitted filename: Response to Reviewers.docx [file pone.0315272.s006.docx]

I would like to thank Editor Wesley Dondoni Colombo, as well as Reviewer 5 and Reviewer 6, for their thorough analysis of my paper and the previous review process. I appreciate the opportunity for a new round of evaluation and the potential for publication.

I have carefully considered each reviewer’s feedback and incorporated most of their suggestions into the revised manuscript, which I am submitting as an attachment. I believe that this third version of the paper meets all the publication requirements. However, if any issues remain, I am open to making further modifications. In a few instances, I provided additional explanations to address misunderstandings or differences in interpretation. While I have presented my arguments, I am not inflexible and am willing to reconsider any perspective if necessary.

I leave the decision about whether to delete the **taxonomic remarks** following each description up to the Editor. This was requested by Reviewer 3, while Reviewer 6 suggested their removal. I am prepared to comply with whichever decision the Editor makes. The current revised version retains the taxonomic remarks, but they can be easily removed if required.

The responses to the reviewers' comments are highlighted **in red** after each assertion. For any answers that led to modifications in the manuscript, I have included a marker (e.g., [*correction001]). These markers make it easier to identify the changes using the document’s locating tool. Please note that these markers are only present in the **Tracking Mode** version of the manuscript. The **Clean Version** does not contain these markers and is free of any annotations.

Once more I thank you all for this new opportunity.

6. Review Comments to the Author

Please use the space provided to explain your answers to the questions above. You may also include additional comments for the author, including concerns about dual publication, research ethics, or publication ethics. (Please upload your review as an attachment if it exceeds 20,000 characters)

Reviewer #4: The manuscript deals with the revision of a previously created genus of land snails with the addition of a set of new species and the creation of another new genus with a single species. All this information is presented in a cladistic analysis with a particular methodology. This work follows the same scheme and methodology as another contribution by the same author previously published in PLOS ONE. Although this is an indisputable fact, contrary to what the author suggests, criticisms can still be made of the method used and the format employed. The author of the present manuscript is a malacologist renowned for his extensive experience in marine gastropods. On this occasion, the manuscript he wrote deals with species of terrestrial pulmonate gastropods and this is evident in the framework used and the selection of characters for the description of species. It is important to emphasize that I am not opposed to criticism. No scientific procedure is immune to it, and when criticisms are well-intentioned and constructive, they are very welcome. Over more than 40 years, they have contributed significantly to shaping the professional I am today. However, Reviewer 1’s comments fall far from this standard. I won’t reiterate all the arguments supporting this perspective here; please refer to the previous documentation, where I thoroughly countered his claims, including those labeling my work as unscientific.

Furthermore, the above assertion suggests that I am a neophyte in the study of land mollusks, with more extensive experience in marine gastropods. This impression is incorrect. As detailed on my curriculum website (<http://lattes.cnpq.br/5020466945403853>), I have authored 640 published works, including 300 papers in peer-reviewed international journals, many of which are highly regarded, such as *PLOS*, *Zoological Journal*, and *Zootaxa*. I have published extensively across all branches of Mollusca, with marine and terrestrial gastropods comprising the majority of my work in equal measure.

A genuine challenge, as Reviewer 6 correctly points out, lies in the phylogenetic complexities of land snails, most of which are Heterobranchia. Their phylogeny is indeed less developed compared to other gastropod groups. In the current paper, I could not delve as deeply into phylogenetic analysis as I typically do with, for instance, caenogastropods. Nonetheless, we can advance our understanding by doing our best to achieve meaningful results, sufficient to address the taxonomy level discussed in the paper.

As has been stated for some time and only to cite a single author on this subject: Morphology-based cladistics, the birthplace of cladistic methods, has been attacked by molecular systematists as hopelessly ambiguous and inevitably dispensable in an era increasingly awash in molecular data (Sereno, 2009). In complete agreement with Sereno, I consider that morphological studies as well as cladistics based on morphology are enormously useful tool and should not be discredited to achieve the advancement of science. It is a pleasure to hear that. I totally agree with this.

At the same time, this kind of study must be rigorous and repeatable, as well as they have to provide predictable hypotheses. The present manuscript, after having already gone through revisions, has been improved since its initial version. It denotes a detailed study of the species involved, with extensive descriptions of organs not generally used after the publication of Tiller (1989). Tillier studied morphological characters from the nervous, pallial and digestive systems in a comparative way of different Pulmonate gastropod groups. This mentioned work is a classic, but received many criticisms for the phylogenetic method used to address its results, in addition to revealing that these characters are highly homoplasic, so in general, only those of the reproductive system continued to be used in taxonomy and phylogeny because are more informative. Tillier (1989) remains a foundational reference for my work. However, because it is quite general and does not focus extensively on orthalicoideans—a group only weakly represented in that paper—I did not cite it in the present study. The notion that non-genital structures are highly homoplastic and of limited use in phylogenetic analysis is, in my view, a misconception or dogma created to justify the shift towards a purely sequencing-based approach. My research has consistently demonstrated otherwise.

Following there is a list of some points for the author to address:
Abstract:
-“a calcified epiphragm, unique in South American snails.” This is incorrect as there are other genera with calcified epiphragm inhabiting South America, please change the sentence. Okay, it was rephrased[*correction001]. However, neither I, nor any colleagues I have consulted, know of any other group of native South American snails that possess a calcified epiphragm. While several groups, such as the Epiphragmophoridae, do produce an epiphragm, it is, however, typically only minimally calcified or non-calcified. I thank any more precise information.

Results:
All the species descriptions are very detailed, but I found the following problems:
1. The diameter of glandular organs is cited as a distinguishing species characteristic. However, glandular organs undergo significant changes due to seasonality, making this trait unreliable for species comparisons—unless a possibility would be that all specimens were collected and preserved during the same season, which is not specified in this work. I understand that glandular organs and other structures can vary with seasonal changes. However, I cannot omit details about their size and proportions in the descriptions. To address potential issues arising from this variability, I typically describe the average size of these organs across specimens. Furthermore, I either avoid using size and proportions in comparative analyses or use them minimally, and I never rely on these characteristics as diagnostic factors.

2. The Sperm inner longitudinal fold is a character described in different species and shown through a drawing of a transverse section of the spermoviduct. However, since histology has not been carried out I wonder how he managed to see such a detailed aspect in the interior glandular organ. Not only its morphology but also how it is inserted and connected to the albumen gland. The detailed anatomical investigation protocols used in this paper are the same as those I have applied in all my previous studies, as explained in the references cited in the Material and Methods section. Although proper histological analysis was not performed (as that constitutes a different type of study), however, serial sections were used to reveal certain minute and complex structures. In this paper, serial sections were only made for a few specimens, as the specimens were large enough to be thoroughly examined through dissection. With this protocol, it is entirely possible to observe and accurately illustrate these structures.

Regarding the folds in the spermoviduct, they can be identified through detailed dissection, as demonstrated in the attached photo of *Kora nigra* (indicated by arrows), which bears two distinct folds. Serial sections are not required to observe these features; the folds are even visible running along the length of the spermoviduct when it is opened longitudinally. For example, see Fig. 6A (sp) in the paper.

3. In the same way, there is apparently a problem in the identification/delimitation of organs and the terminology used by the author since he describes and equals the “Carrefour” with the fertilization pouch-spermathecal complex organ. The Carrefour is a portion basal to that structure and usually it is embedded into the albumen gland, for which only with histological methods is possible to see how it is connected either to the hermaphroditic duct or to the albumen gland/spermividuct. See Tompa (1984) for a better description of this area. Figure 11C for example shows a fertilization pouch-spermathecal complex term as “Carrefour” by the author with a long basal tube described as “simple, wide, ~twice longer than receptacle and 3-times its width”. It is not clear to me if this an inner duct (embedded into the albumen gland) or if it is external. Is it consider as part of the “carrefour” in the present manuscript? The terminology used for pulmonate snails is not uniform across different schools of thought, resulting in significant variation. I have consistently used the terminology established in my previous papers, which has been influenced by Leme, Scott, Paraense, and others. In this context, "carrefour" refers to the "region of fertilization," “talon,” or "crossing," as described by some authors. It is important to maintain terminological consistency based on my previous work. Additionally, yes, most of the carrefour is located within the albumen gland and requires dissection of it to access it; serial sections or histological analysis are not necessary. The detailed dissection protocols I applied are sufficient to fully expose it, as shown in Fig. 11C. Fig. 11A provides a natural view of the carrefour still immersed in the albumen gland (ag). I have numerous photos documenting these dissections if the reviewer requires further evidence. Annexed, I have included a photo that illustrates a stage of the dissection that produced Fig. 11C, showing the exposed carrefour (arrow) and its connection to the beginning of the spermoviduct.

Above, I presented my rationale for using the term "carrefour" instead of "fertilization pouch-spermathecal complex organ," to maintain consistency with my previous papers. However, I understand that this is a request, and if the reviewer believes it is important to make this terminological change, I am open to it at this stage of the review. I only ask for permission to document this terminological change in the Material and Methods section.

4. Taxonomic remarks are excessively long, often repeating characters already described for each species. They should be more concise highlighting the differences of main characters. I fully agree with this point. The taxonomic remarks were not present in the first two versions of the paper but were introduced in the third version to address the requirements of Reviewer 3. In my opinion, the species comparisons and discussions were already thoroughly addressed, *ad nauseam*, in the Discussion section, as well as in the tables and phylogenetic analyses. Therefore, I believe they can be completely omitted. However, I defer to the Editor and Reviewer on this matter, and I am fine with their deletion.

Therefore, I leave it to the Editor to decide whether I should address Reviewer 3's request and keep the taxonomical remarks or follow Reviewer 6's suggestion and remove them. I prefer the solution proposed by Reviewer 6.

5. Pag. 46: author said: …because it lacks the reticulated sculpturing seen in the protoconchs of Bulimulus. This is incorrect since species of Bulimulus lack of a reticulate Protoconch. Ok, rephrased[*correction002]. ‘Wrinkles’ was introduced as a character of Bulimulus’ protoconch based on Breure (1979).

6. It is not explained why or for what purpose two cladistic softwares such as TNT and PAUP were used or if differences were found between them. Nor is it indicated which commands and strategy were used for the phylogenetic analysis performed. Regardless of establishing that a method already published is used, the author must indicate which particular strategy was used for the analysis of this data. Ok, I introduced an additional paragraph at the end of the item “Phylogenetic analysis” in the Material and Methods to attend this requirement[*correction003].

7. Another interesting feature is the presence of a thick muscular region in the basal portion of the duct of the bursa copulatrix (e.g., Fig. 6A: bu)” A better drawing or photo is needed here because Fig. 6A is not clear in indicating the basal area of the duct. Yes, that character is very interesting, and it appears clearly in Fig. 6A, where the cut basal walls of the bursa duct (indicated by “bu”) are much thicker than those of the preceding section. Additionally, this thick region (bu) is also illustrated in Figs. 11A, 12N (a photograph), 20B, 23C, and 38B.

8. “A second diverticulum-like projection, usually balloon-like, also filled with albumen and interpreted as an accessory albumen chamber (as), is a novelty in the presently studied species, being absent only in Koltrora”. The terminology used is confusing since this area of union between the albumen gland and the spermatoviduct is present in many Pulmonate snails, but does not have a projection shape nor is it a diverticulum. In the region between the albumen gland and the beginning of the spermoviduct, there is typically an **albumen chamber**, a sac where the albumen gland stores albumen. The *Kora* also possesses this structure. The “**accessory albumen chamber**” is an additional feature, located slightly more anteriorly and connected by a different duct. In the figures, the usual albumen chamber, found in most pulmonates, is labeled as ‘ac,’ while the accessory one, a novelty of *Kora*, is labeled ‘as.’ The term “balloon-like” describes its shape, as it usually features a narrow duct leading to a bulged, swollen distal portion that resembles a flying balloon. It is classified as a diverticulum because any blind sac that projects from a tube in anatomy is termed a “diverticulum.” This terminology is commonly used in anatomy, to my knowledge. These terms seem clear to me, but I am happy to make changes if any clarification is requested.

Discussion:
Taxonomic discussion
The first and second paragraphs of the discussion should be compacted because they are repetitive and seem to be more of a justification of what was done in the work. Throughout the text, there are also many repetitions of what was already said in Results and at the same time in the same Discussion they are repeated at least twice. The text should be more concise discussing the results obtained concerning what was previously known. In turn, I find it difficult to follow the excessively detailed comparisons of the musculature of the buccal mass and odontophore whose importance is lost when the information is not hierarchical. These paragraphs were included as responses to previous assertions made by the previous referees. In light of the current criticisms, they have been deleted [*correction004]. Relevant information from these sections has been transferred to the Material and Methods section[*correction005].

The author sustained that “Importantly, no exclusive diagnostic character was obtained, neither at the genus nor at the species levels”, Does it refer to not having identified autapomorphies? How do you interpret the set of diagnostic characters detailed in each case? This is contradictory to the cladistic analysis carried out as well as to the identification of a set of particular characters for each genus. Despite complementary, the taxonomical and phylogenetic scenarios are different in papers. Yes, there are indeed several autapomorphies present among all examined taxa. The translucent shell of *Koltrora* serves as one example. It is indeed a diagnostic character, however, autapomorphies are not utilized in phylogenetic analyses because they do not contribute to resolving evolutionary relationships and are typically excluded in the final stages to prevent index inflation. The only autapomorphies retained—and those are depicted in Fig. 61—are those that turned out to be homoplastic or transformational. The intent of the explanatory statement is to convey that no character remains entirely exclusive; instead, characters show convergence with other orthalicoideans. For example, shell mentioned translucency, while characteristic of *Koltrora*, also appears in the unrelated simpulopsid *Leiostracus perlucidus*.

Even the accessory albumen chamber, which could be considered a synapomorphy for *Kora*, is unexpectedly also present in *Anctus*, as demonstrated in the paper—highlighting a case of convergence. Thus, there is no contradiction. Phylogenetic analyses commonly encounter convergences and reversions, i.e., homoplasies, across all groups. Taxa based on phylogenetic methods are defined by a set of apomorphies. If any of these apomorphies prove to be exclusive, that is beneficial but uncommon, as such traits are typically excluded from the analysis as explained above. Ultimately, taxa are defined by the suite of apomorphies identified, regardless of whether some also define other groups as homoplasies.

To draw a parallel, homeothermy defines Mammalia but is also characteristic of Aves. In the species diagnoses, however, the previously disregarded autapomorphies have been recovered. This led to a rephrasing of that explanation.[*correction006]

Please avoid using the following words as they are totally subjective and only meaningful in a narrative context: Importantly, interesting (used several times), relatively, mostly, extraordinary, impressively, Notably, enigmatic. All these terms were removed from the new manuscript, though in a few instances they may need to be replaced with smoother alternatives. However, I would like to request permission to retain the words “relatively” and “mostly.” These terms are important because they accurately convey degrees of the described features: “relatively” indicates ‘more or less,’ while “mostly” refers to ‘the majority, but not entirely.’ I believe these terms are preferable to their alternatives. Nonetheless, I am open to suggestions if there are better options available. All other terms above have been omitted from the revised manuscript and are not individually marked, as there were too many to highlight.

-“The ureter is totally closed (tubular) in Kora and Koltrora, as apparently is the usual pattern of Orthalicoideans but Neopetraeus has the ureter opened in a larger degree”. This is incorrect since there are other genera classified in Orthalicoidea with this same characteristic. Ok, rephrased[*correction007]

-“Koltrora differs from Kora….by the thick glandular uterine walls (Fig. 47E: ut); by the epiphallus being widely open to the penis”. The first character is inappropriate since a glandular reproductive organ varies seasonally in its diameter/thickness. The second mentioned character is not clear since the relationship of the epiphallus with the penis is not clarified nor is it detectable in the figures. I emphasize that it is essential to report the observations made from the examined samples. These features are evident and noteworthy. Additionally, *Koltrora* specimens were collected in April, the same season as most *Kora* samples, as documented in the "Material Examined" sections. Moreover, the glandular uterine wall (Fig. 47E) appears distinctly different from a weakly glandular one. While all uterine mucosa produce mucus (thus functioning as glands), this distinction is evident in specimens with an empty uterus.

The connection between the penis and epiphallus in *Koltrora* is broadly joined, this looks clearly illustrated in Fig. 48C, which contrasts with the narrower connection observed in the other species. Nevertheless, these two traits are not decisive on their own; they merely help in the support the generic differentiation. There are other, more significant features that form the basis for distinguishing the genera, and, I wonder, not all characters need to carry the same weight.

-In diagnosing Kora, the author includes characters shared with Koltrora and Neopetraeus. Only those characters unique to Kora that justify its difference from the other genera should be mentioned. The characters described are also crucial for distinguishing *Koltrora* and *Kora* from other orthalicoidean genera. If we were to restrict the analysis solely to exclusive characters, the taxa addressed in this paper would be resolved; however, this approach would not adequately differentiate them from other related genera. Therefore, I request permission to keep the current diagnoses as they are, since they are essential for distinguishing these genera from others that are still under study. Diagnoses are supposed to be more widely applied.

-“Concerning the odontophore, all species exhibit fused cartilages along the ventral edge, but the degree of fusion varies. Koltrora has approximately 100%, while K. uhlei has around 90%; K. nigra has the smallest fusion value of approximately 60%, and the remaining species are about 75% fused.” How is the degree of fusion in % of the cartilages of the odontophore measured? The differences in % are very subtle and merit an explanation of how they were obtained. Ok, an explanation was inserted in the M&M[*correction008]

-What does it mean that the m8 muscles are relatively uniform?? Yes, the pair m8 is in the outer (dorsal) edge of the cartilages, and does not show any detected distinction amongst the species. Thus, this feature was not used, only its presence/absence.

-The author sustained that “The carrefour region, for example, has a unique arrangement in each species (Figs 6B, 11C, 15A, 20E, 23E, 27E, 33E, 38A, 47C, 53D, 57B).” However, the mentioned figures show significant similarities in the morphology of the organ between Fig.11C and 15A, between 20E and 33E, also between Fig 23E and 27E making this assertion doubtful or at least these figures are not sufficiently clear to show the difference that the author maintains. What I am emphasizing here is that the carrefour region, often overlooked even in anatomical studies, reveals intriguing features. This does not imply that some species are not occasionally similar to one another. In the evoked examples, in Fig. 11C, the carrefour is swollen and connects to the albumen duct, whereas in Fig. 15A, it is narrow and connects to the beginning of the spermoviduct—showing clear distinctions. Additionally, the also reported Fig. 23E illustrates the carrefour connecting to a curved albumen chamber, while in Fig. 27E, the carrefour connects at the intersection of the albumen duct and the start of the spermoviduct, with the albumen chamber appearing sac-like and bifid. These configurations are also clearly distinct.

The title “Biogeographic analysis” should be changed to “Distribution” since no analysis was performed. Ok. Done.

Cladistic Analysis
The goal in cladistics is not always to find a single tree but to identify the most likely and robust relationships, which may involve accepting that there are multiple equally valid phylogenetic hypotheses. Finding multiple equally parsimonious trees is a common situation in cladistics, and although it can present some challenges, this does not imply that the analysis is flawed or useless. Parsimony is one of the criteria for evaluating phylogeny, and finding multiple parsimonious trees means that there are several equally valid hypotheses based on the current data. It may reflect the reality of the evolutionary data. The key is how to interpret and handle this situation. I completely agree. At no point in the paper do I argue against the use of multiple cladogram analyses. In fact, I have sometimes obtained multiple cladograms in my previously published phylogenies. However, in the present study, I derived a single cladogram, which does not diminish the quality of the analysis. On the contrary. The technique I used to reduce the number of cladograms was biological, rather than statistical (such as using a strict consensus), and it has already been published with favorable reception [<http://www.moluscos.org/trabalhos/Malacopedia/06-042023%20Malacopedia%20less%20trees.pdf>].

The tree obtained in the phylogenetic analysis is described in the Discussion, not in the Results section. This should be migrated to Results and only a discussion of the relationships found in the proposed hypothesis should be made. Ok, both paragraphs reporting the results have been moved to the end of the Results section [*correction009]. Only the discussion points remain in the Discussion section.

Some characters that result in synapomorphies in the analysis carried out are doubtful due to what was previously explained about "carrefour" and its delimitation, for example. Character64. That indeed was wrongly written, the hermaphrodite duct inserts actually in the carrefour, and not in seminal receptacle. That was a mistake, and was corrected, [*correction010] thank you.

The author sustained that in node 12, 11 synapomorphies support its monophyly and then mentioned the “interesting” ones which in fact are the total set of synapomorphies. Why they are “interestings”? It is not informative to indicate that they are interesting, since having resulted in synapomorphic characters they support the monophyly of the clade. If you wanted to say something more about each of them, you should specify it in another way. I am sorry, that was the excitement of a workaholic. All those “interesting’s” were deleted in the new version.

In page 62 the author mentioned that Koltrora pyrostoma has “13 homoplastic autapomorphies, being 4 of them reversions in the present context; two of them are noteworthy: the loss of the accessory albumen chamber (ch. 70) and of the penis
shield (ch. 80). This set of autapomorphies, and the lack of the Kora synapomorphies, are
sufficient reasons for the generic separations of both taxa, despite they share more characters than Neopetraeus. Please explain why the absence of characters are enough to define the monophyly of a genus. The set of remaining autapomorphies for *Koltrora*, after disregarding certain others, could potentially become synapomorphies for the genus if additional congeneric species are discovered. Moreover, the species lack the defining characteristics present in the node supporting *Kora* (node 13). This reasoning is outlined in the paragraph: there are numerous differences from the known genera and an absence of the defining characters of those genera. Therefore, *Koltrora* is justified as a new genus. The argument focuses on this argumentation, not on the mere absence of characters.

Again in the same page, the author says: The Kora branch (node 13) has the extraordinary support of 25 synapomorphies. The more interesting are: characters 6,7,15,18,29, 36,37,45,47,57,59,62,74,78,83,94”. Why is that? Are there synapomorphies? Which criteria follows the author to consider them “interesting” characters? The term “more interesting” was used in this context to spare the reader from a full list of synapomorphies, which can be consulted in the provided character list. The intention was simply to inform that there are 25 (in the example) well-supported synapomorphies. As the author who personally dissected and studied the specimens, selected the characters, reviewed the literature, and has extensive experience in molluscan anatomy across all branches, I highlighted fewer synapomorphies from the list that I considered noteworthy for the reader. This rhetorical approach has been employed in all my previous papers without any prior complaints. However, since this point appeared to be important to the Reviewer, I have removed all instances of “interesting” from the manuscript. I hope this satisfies the Reviewer’s requirement.

Reviewer #5: I commend the author for the work done, the detail in the descriptions, and the composure in responding to each of the reviewers' suggestions. Thank you!
